# Supplementary material for: Metabolomics insights into the polyketide-lactones produced by Diaporthe caliensis sp. nov., an endophyte of the medicinal plant Otoba gracilipes
Source: Microbiol Spectr. 2023 Nov 3;11(6):e02743-23. doi: 10.1128/spectrum.02743-23 (PMC10715209; doi:10.1128/spectrum.02743-23)
Supplement: Supplemental material — Fig. S1 to S23 and Tables S1 to S5. [file spectrum.02743-23-s0001.docx]

Metabolomics insights into the polyketide-lactones produced by *Diaporthe caliensis* sp. nov., an endophyte of the medicinal plant *Otoba gracilipes*

Esteban Charria-Girón^a,b,c^, Yasmina Marin-Felix^a,b,#^, Ulrike Beutling^d^, Raimo Franke^d^, Mark Brönstrup^d^, Aida M. Vasco-Palacios^e,f^, Nelson H. Caicedo^c,g^ and Frank Surup^a,b,#^

^a^Department Microbial Drugs, Helmholtz Centre for Infection Research (HZI), German Centre for Infection Research (DZIF), Partner Site Hannover-Braunschweig, Inhoffenstrasse 7, 38124 Braunschweig, Germany

^b^Institute of Microbiology, Technische Universität Braunschweig, Spielmannstraße 7, 38106 Braunschweig, Germany

^c^Universidad Icesi, Facultad de Ingeniería, Diseño y Ciencias Aplicadas, Departamento de Ingeniería Bioquímica, Calle 18 No. 122 - 135, Cali, Colombia

^d^Department Chemical Biology, Helmholtz Centre for Infection Research GmbH (HZI), Inhoffenstrasse 7, 38124 Braunschweig, Germany

^e^Grupo de Microbiología Ambiental y Grupo BioMicro, Escuela de Microbiología, Universidad de Antioquia UdeA, Medellín, Colombia

^f^Asociación Colombiana de Micología, ASCOLMIC, Colombia

^g^Universidad Icesi, Centro BioInc, Calle 18 No. 122 - 135, Cali, Colombia

Running head: Secondary metabolites of *Diaporthe caliensis* sp. nov.

#Address correspondence to Yasmina Marin-Felix, yasmina.marinfelix@helmholtz-hzi.de, and Frank Surup, frank.surup@helmholtz-hzi.de

**Table S1.** Isolated and reference strains of *Diaporthe* included in this study. # GenBank accession numbers in **bold** were newly generated in this study. The new taxon is indicated in ***bold italic*** font

| **Species** | **Isolates^1^** | **GenBank accession numbers^2^** | | | | | **References** |
| --- | --- | --- | --- | --- | --- | --- | --- |
|  |  | **ITS** | ***tub2*** | ***his3*** | ***tef1*** | ***cal*** |  |
| *Diaporthe acaciarum* | CBS 138862^T^ | KP004460 | KP004509 | KP004504 | - | - | (1) |
| *D. acericola* | MFLUCC 17-0956^T^ | KY964224 | KY964074 | - | KY964180 | KY964137 | (2) |
| *D. alangii* | CFCC 52556^T^ | MH121491 | MH121573 | MH121451 | MH121533 | MH121415 | (3) |
| *D. ambigua* | CBS 114015^T^ | KC343010 | KC343978 | KC343494 | KC343736 | KC343252 | (4) |
| *D. amygdali* | CBS 126679^T^ | KC343022 | KC343990 | KC343506 | KC343748 | KC343264 | (4) |
| *D. angelicae* | CBS 111592^T^ | KC343026 | KC343994 | KC343511 | KC343752 | KC343268 | (4) |
| *D. arctii* | CBS 136.25 | KC343031 | KC343999 | KC343515 | KC343757 | KC343273 | (4) |
| *D. arezzoensis* | MFLU 19-2880^T^ | MT185503 | MT454055 | - | - | - | (5) |
| *D. batatas* | CBS 122.21 | KC343040 | KC344008 | KC343524 | KC343766 | KC343282 | (4) |
| *D. beilharziae* | BRIP 54792^T^ | JX862529 | KF170921 | - | JX862535 | - | (6) |
| *D. biguttulata* | ICMP 20657^T^ | KJ490582 | KJ490403 | KJ490524 | KJ490461 | - | (7) |
| *D. breyniae* | CBS 148910^T^ | ON400846 | ON409186 | ON409187 | ON409188 | ON409189 | (8) |
| ***D. caliensis*** | **CM-UDEA-H27^T^** | **OPS456330** | **OP503173** | **OP503172** | **OP503171** | **-** | **Present study** |
| *D. camporesii* | JZB 320143^T^ | MN533805 | MN561316 | - | - | - | (9) |
| *D. caryae* | CFCC 52563^T^ | MH121498 | MH121580 | MH121458 | MH121540 | MH121422 | (3) |
| *D. celtidis* | NCYU 19-0357^T^ | MW114346 | MW148266 | - | MW192209 | - | (10) |
| *D. cerradensis* | CMRP 4331^T^ | MN173198 | MW751671 | MW751663 | MT311685 | MW751655 | (11) |
|  |  |  |  |  |  |  |  |
| *D. chimonanthi* | SCHM 3614^T^ | AY622993 |  |  |  |  | (12) |
| *D. chinensis* | MFLUCC 19-0101^T^ | MW187324 | MW245013 | - | MW205017 | MW294199 | (13) |
| *D. chromolaenae* | MFLUCC 17-1422^T^ | MH094275 | - | - | - | - | (14) |
| *D. cichorii* | MFLUCC 17-1023^T^ | KY964220 | KY964104 | - | KY964176 | KY964133 | (2) |
| *D. cinnamomi* | CFCC 52569^T^ | MH121504 | MH121586 | MH121464 | MH121546 | - | (3) |
| *D. citriasiana* | CBS 134240^T^ | JQ954645 | KC357459 | MF418282 | JQ954663 | KC357491 | (15) |
| *D. compacta* | LC3083^T^ | KP267854 | KP293434 | KP293508 | KP267928 | - | (16) |
| *D. convolvuli* | CBS 124654 | KC343054 | KC344022 | KC343538 | KC343780 | KC343296 | (4) |
| *D. cucurbitae* | DAOM 42078^T^ | KM453210 | KP118848 | KM453212 | KM453211 | - | (17) |
| *D. cuppatea* | CBS 117499^T^ | AY339322 | JX275420 | KC343541 | AY339354 | JX197414 | (18) |
| *D. discoidispora* | ICMP 20662^T^ | KJ490624 | KJ490445 | KJ490566 | KJ490503 | - | (7) |
| *D. durionigena* | VTCC 930005^T^ | MN453530 | MT276159 | - | MT276157 | - | (19) |
| *D. endophytica* | CBS 133811^T^ | KC343065 | KC344033 | KC343549 | KC343791 | KC343307 | (4) |
| *D. eres* | CBS 138594^T^ | KJ210529 | KJ420799 | KJ420850 | KJ210550 | KJ434999 | (20) |
| *D. fici-septicae* | MFLU 18-2588^T^ | MW114348 | MW148268 | - | MW192211 | - | (10) |
| *D. fructicola* | MAFF 246408^T^ | LC342734 | LC342736 | LC342737 | LC342735 | LC342738 | (21) |
| *D. ganjae* | CBS 180.91^T^ | KC343112 | KC344080 | KC343596 | KC343838 | KC343354 | (4) |
| *D. glabrae* | SCHM 3622^T^ | AY601918 | - | - | - | - | (12) |
| *D. goulteri* | BRIP 55657a^T^ | KJ197290 | KJ197270 | - | KJ197252 | - | (6) |
| *D. guangdongensis* | ZHKUCC20-0014^T^ | MT355684 | MT409292 | - | MT409338 | MT409314 | (22) |
| *D. gulyae* | BRIP 54025^T^ | JF431299 | KJ197271 | - | JN645803 | - | (6) |
| *D. guttulata* | CGMCC 3.20100^T^ | MT385950 | MT424705 | MW022491 | MT424685 | MW022470 | (23) |
| *D. helianthi* | CBS 592.81^T^ | KC343115 | KC344083 | KC343599 | KC343841 | JX197454 | (4) |
| *D. heterostemmatis* | SAUCC 194.85^T^ | MT822613 | MT855810 | MT855581 | MT855925 | MT855692 | (24) |
| *D. hordei* | CBS 481.92 | KC343120 | KC344088 | KC343604 | KC343846 | KC343362 | (4) |
| *D. hubeiensis* | JZB 320123^T^ | MK335809 | MK500148 | - | MK523570 | MK500235 | (25) |
| *D. infecunda* | CBS 133812^T^ | KC343126 | KC344094 | KC343610 | KC343852 | KC343368 | (4) |
| *D. infertilis* | CBS 230.52^T^ | KC343052 | KC344020 | KC343536 | KC343778 | KC343294 | (26) |
| *D. kochmanii* | BRIP 54033^T^ | JF431295 | - | - | JN645809 | - | (19) |
| *D. kongii* | BRIP 54031^T^ | JF431301 | KJ197272 | - | JN645797 | - | (19) |
| *D. leucospermi* | CBS 111980^T^ | JN712460 | KY435673 | KY435653 | KY435632 | KY435663 | (27) |
| *D. longicolla* | FAU 599^T^ | KJ590728 | KJ610883 | KJ659188 | KJ590767 | KJ612124 | (17) |
| *D. longispora* | CBS 194.36^T^ | KC343135 | KC344103 | KC343619 | KC343861 | KC343377 | (4) |
| *D. lusitanicae* | CBS 123212^T^ | KC343136 | KC344104 | KC343620 | KC343862 | KC343378 | (4) |
| *D. machili* | SAUCC 194.111^T^ | MT822639 | MT855836 | MT855606 | MT855951 | MT855718 | (28) |
| *D. manihotia* | CBS 505.76 | KC343138 | KC344106 | KC343622 | KC343864 | KC343380 | (4) |
| *D. masirevicii* | BRIP 57892a^T^ | KJ197277 | KJ197257 | - | KJ197239 | - | (6) |
| *D. mayteni* | CBS 133185^T^ | KC343139 | KC344107 | KC343623 | KC343865 | KC343381 | (4) |
| *D. megalospora* | CBS 143.27 | KC343140 | KC344108 | KC343624 | KC343866 | KC343382 | (4) |
| *D. melonis* | CBS 507.78^T^ | KC343142 | KC344110 | KC343626 | KC343868 | KC343384 | (4) |
| *D. micheliae* | SCHM 3603 | AY620820 | - | - | - | - | (12) |
| *D. middletonii* | BRIP 54884e^T^ | KJ197286 | KJ197266 | - | KJ197248 | - | (6) |
| *D. myracrodruonis* | URM 7972^T^ | MK205289 | MK205291 | - | MK213408 | MK205290 | (29) |
| *D. neoarctii* | CBS 109490 | KC343145 | KC344113 | KC343629 | KC343871 | KC343387 | (4) |
| *D. neoraonikayaporum* | MFLUCC 14-1136^T^ | KU712449 | KU743988 | - | KU749369 | KU749356 | (30) |
| *D. novem* | CBS 127271^T^ | KC343157 | KC344125 | KC343641 | KC343883 | KC343399 | (4) |
| *D. ovalispora* | ICMP 20659^T^ | KJ490628 | KJ490449 | KJ490570 | KJ490507 | - | (7) |
| *D. pachirae* | COAD 2074^T^ | MG559537 | MG559541 | - | MG559539 | MG559535 | (31) |
| *D. passifloricola* | CBS 141329^T^ | KX228292 | KX228387 | KX228367 | - | - | (32) |
| *D. phaseolorum* | CBS 113425 | KC343174 | KC344142 | KC343658 | KC343900 | KC343416 | (4) |
| *D. pseudolongicolla* | CBS 117165^T^ | DQ286285 | - | - | DQ286259 | - | (33) |
| *D. pyracanthae* | CBS142384^T^ | KY435635 | KY435666 | KY435645 | KY435625 | KY435656 | (34) |
| *D. racemosae* | CBS 143770^T^ | MG600223 | MG600227 | MG600221 | MG600225 | MG600219 | (35) |
| *D. raonikayaporum* | CBS 133182^T^ | KC343188 | KC344156 | KC343672 | KC343914 | KC343430 | (4) |
| *D. rosae* | MFLUCC 17-2658^T^ | MG828894 | MG843878 | - | - | MG829273 | (36) |
| *D. rosiphthora* | COAD 2913^T^ | MT311196 | - | - | MT313692 | MT313690 | (37) |
| *D. rossmaniae* | CAA 762^T^ | MK792290 | MK837914 | MK871432 | MK828063 | MK883822 | (38) |
| *D. sackstonii* | BRIP 54669b^T^ | KJ197287 | KJ197267 | - | KJ197249 | - | (6) |
| *D. sambucusii* | CFCC 51986^T^ | KY852495 | KY852511 | KY852503 | KY852507 | KY852499 | (3) |
| *D. schini* | CBS 133181^T^ | KC343191 | KC344159 | KC343675 | KC343917 | KC343433 | (4) |
| *D. schoeni* | MFLU 15-1279^T^ | KY964226 | KY964109 | - | KY964182 | KY964139 | (2) |
| *D. sclerotioides* | CBS 296.67^T^ | KC343193 | KC344161 | KC343677 | KC343919 | KC343435 | (4) |
| *D. serafiniae* | BRIP 55665a^T^ | KJ197274 | KJ197254 | - | KJ197236 | - | (6) |
| *D. siamensis* | MFLUCC 10-0573a | JQ619879 | JX275429 | - | JX275393 | - | (39) |
| *D. sinensis* | CGMCC 3.19521^T^ | MK637451 | MK660447 | - | MK660449 | - | (40) |
| *D. sojae* | CBS 139282^T^ | KJ590719 | KJ610875 | KJ659208 | KJ590762 | KJ612116 | (17) |
| *D. stewartii* | CBS 193.36 | FJ889448 | - | - | GQ250324 | - | (41) |
| *D. subellipicola* | KUMCC 17-0153^T^ | MG746632 | MG746634 | - | MG746633 | - | (42) |
| *D. subordinaria* | CBS 101711 | KC343213 | KC344181 | KC343697 | KC343939 | KC343455 | (4) |
| *D. tecomae* | CBS 100547 | KC343215 | KC344183 | KC343699 | KC343941 | KC343457 | (4) |
| *D. tectonae* | MFLUCC 12-0777^T^ | KU712430 | KU743977 | - | KU749359 | KU749345 | (30) |
| *D. tectonendophytica* | MFLUCC 13-0471^T^ | KU712439 | KU743986 | - | KU749367 | KU749354 | (30) |
| *D. terebinthifolii* | CBS 133180^T^ | KC343216 | KC344184 | KC343700 | KC343942 | KC343458 | (4) |
| *D. thunbergiicola* | MFLUCC 12-0033^T^ | KP715097 | - | - | KP715098 | - | (43) |
| *D. tulliensis* | BRIP 62248a | KR936130 | KR936132 | - | KR936133 | - | (44) |
| *D. ueckeri* | FAU 656 | KJ590726 | KJ610881 | KJ659215 | KJ590747 | KJ612122 | (7) |
|  | BRIP 54736j (type of *D. miriciae*) | KJ197283 | KJ197263 | - | KJ197245 | - | (6) |
| *D. unshiuensis* | CGMCC 3.17569^T^ | KJ490587 | KJ490408 | KJ490529 | KJ490466 | - | (7) |
| *D. vexans* | CBS 127.14 | KC343229 | KC344197 | KC343713 | KC343955 | KC343471 | (4) |
| *D. vitimegaspora* | STE-U 2675 | AF230749 | - | - | - | - | (45) |
| *D. vochysiae* | LGMF 1583^T^ | MG976391 | MK007527 | MK033323 | MK007526 | MK007528 | (46) |
| *D. yunnanensis* | CGMCC 3.18289^T^ | KX986796 | KX999228 | KX999267 | KX999188 | KX999290 | (47) |

BRIP: Queensland Plant Pathology Herbarium, Brisbane, Australia; CBS: Westerdijk Fungal Biodiversity Institute, Utrecht, the Netherlands; CGMCC: Chinese General Microbiological Culture Collection Center, Beijing, China; CM-EM-UDEA: Colección de microorganismos-Escuela de Microbiología, UdeA, Medellin, Colombia; COAD: Culture Collection of Octávio de Almeida Drumond. Universidade Federal de Viçosa, Viçosa, Brasil; FAU: Isolates in culture collection of Systematic Mycology and Microbiology Laboratory; ICMP: International Collection of Micro-organisms from Plants, Auckland, New Zealand; KUMCC: Kumming Institute of Botany, Kumming, China; LGMF, Laboratório de Genética de Microorganismos (LabGeM) culture collection, at the Federal University of Paraná, Brazil; MAFF: Ministry of Agriculture, Forestry and Fisheries, Tokyo, Japan; MFLUCC: Mae Fah Luang University Culture Collection, Chiang Rai, Thailand; SAUCC: Shandong Agricultural University Culture Collection, Shandong, China; STE-U: Department of Plant Pathology, Stellenbosch University, Stellenbosch, South Africa; URM: Culture Collection at the Universidad Federal de Pernambuco, Recife, Brazil; VTCC: Vietnam Type Culture Collection, Center of Biotechnology, Vietnam National University, Hanoi, Vietnam; ZHKUCC: Culture Collection of Zhongkai University of Agriculture and Engineering, Guangzhou, China. ^T^ indicates type material

**GNPS result link:**

Positive electrospray ionization mode: https://gnps.ucsd.edu/ProteoSAFe/status.jsp?task=d6ed92c4b15c44c6bbf564dd43d8d71b

**Table S2.** Dereplicated metabolites from the OFT culture of *Diaporthe caliensis* sp. nov. and isolated metabolites.

| **Compound** | ***m/z*** | **rt** | **Formula** | **Annotation*** | **Database** |
| --- | --- | --- | --- | --- | --- |
| Trichocladinol C | 229.1074 [M+H]^+^ | 3.75 | C_11_H_16_O_5_ | Level 2 | NP Atlas (Genus: *Diaporthe*) |
| Udagawanone B | 177.0547 [M−H2O+H]^+^ | 4.33 | C_10_H_10_O_4_ | Level 2 | NP Atlas (Genus: *Diaporthe*) |
| Multiforisin A | 207.0651 [M−H2O+H]^+^ | 4.51 | C_11_H_12_O_5_ | Level 2 | NP Atlas (Genus: *Diaporthe*) |
| Gulypyrone A | 213.1125 [M+H]^+^ | 4.94 | C_11_H_16_O_4_ | Level 2 | NP Atlas (Genus: *Diaporthe*) |
| Udagawanone A | 211.0968 [M+H]^+^ | 5.25 | C_11_H_14_O_4_ | Level 2 | NP Atlas (Genus: *Diaporthe*) |
| Multiforisin A | 225.0688 [M+H]^+^ | 5.63 | C_11_H_12_O_5_ | Level 2 | NP Atlas (Genus: *Diaporthe*) |
| Pyrenocine L | 267.1226 [M−H2O+H]^+^ | 6.35 | C_14_H_20_O_6_ | Level 2 | NP Atlas (Genus: *Phomopsis*) |
| Caliensolide A (**2**) | 239.1280 [M+H]^+^ | 7.06 | C_13_H_18_O_4_ | Level 0 | Present study |
| Diaportheone B | 203.0702 [M−H2O+H]^+^ | 7.34 | C_12_H_12_O_4_ | Level 2 | NP Atlas (Genus: *Diaporthe*) |
| Caliensolide B (**3**) | 235.0970 [M+H]^+^ | 7.44 | C_13_H_14_O_4_ | Level 0 | Present study |
| Kobifuranone B | 195.1023 [M+H]^+^ | 7.64 | C_11_H_14_O_3_ | Level 2 | NP Atlas (Genus: *Diaporthe*) |
| Chaetolactone | 253.1442 [M+H]^+^ | 7.67 | C_14_H_20_O_4_ | Level 2 | NP Atlas (Genus: *Diaporthe*) |
| Phomol | 395.2435 [M−H_2_O+H]^+^ | 10.04 | C_22_H_36_O_7_ | Level 1 | NP Atlas (Genus: *Phomopsis*) |
| (3*R*,4*R*,7*S*,8*S*,9*R*)-phomol (**1**) | 413.2538 [M+H]^+^ | 12.13 | C_22_H_36_O_7_ | Level 0 | Present study |

*Level 0 corresponds to isolated metabolites; level 1 to annotations by comparison with standards; level 2 to putative annotations by comparison of measured MS/MS spectra and *in silico* predicted MS/MS spectra of compounds reported for *Diaporthe* and *Phomopsis* spp. in NP Atlas. rt = retention time in min.

**
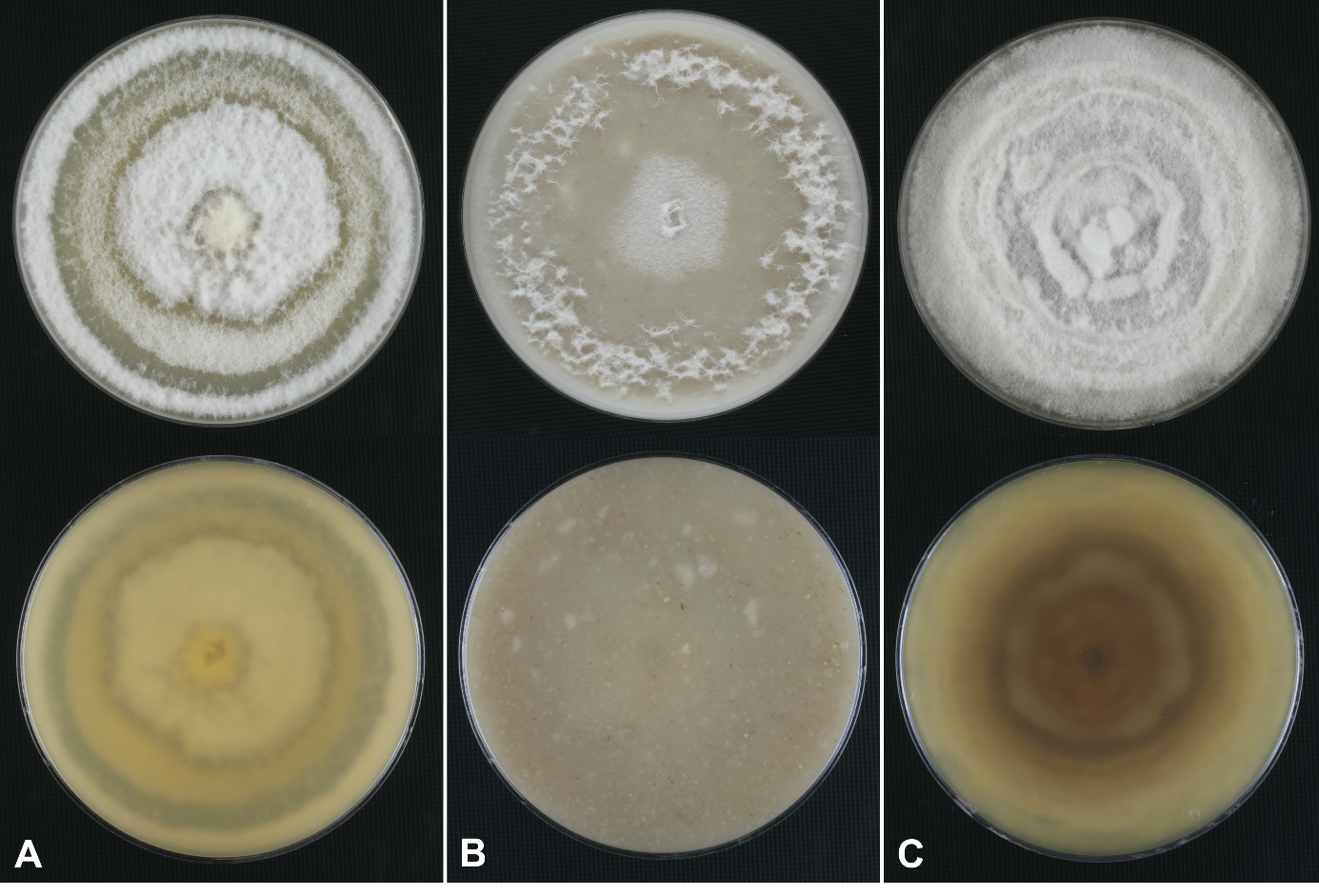
Figure S1**. **A.** Colony in MEA (front and reverse). **B.** Colony in OA (front and reverse). **C.** Colony in PDA (front and reverse).

**Figure S2**. ^1^H NMR spectrum (700 MHz, pyridine-*d*_5_) of phomol (**1**).

**Figure S3**. ^13^C NMR spectrum (175 MHz, pyridine-*d*_5_) of phomol (**1**).

**Figure S4**. ^1^H NMR spectrum (500 MHz, DMSO-*d*_6_) of caliensolide A (**2**).

**Figure S5**. ^13^C NMR spectrum (125 MHz, DMSO-*d*_6_) of caliensolide A (**2**).


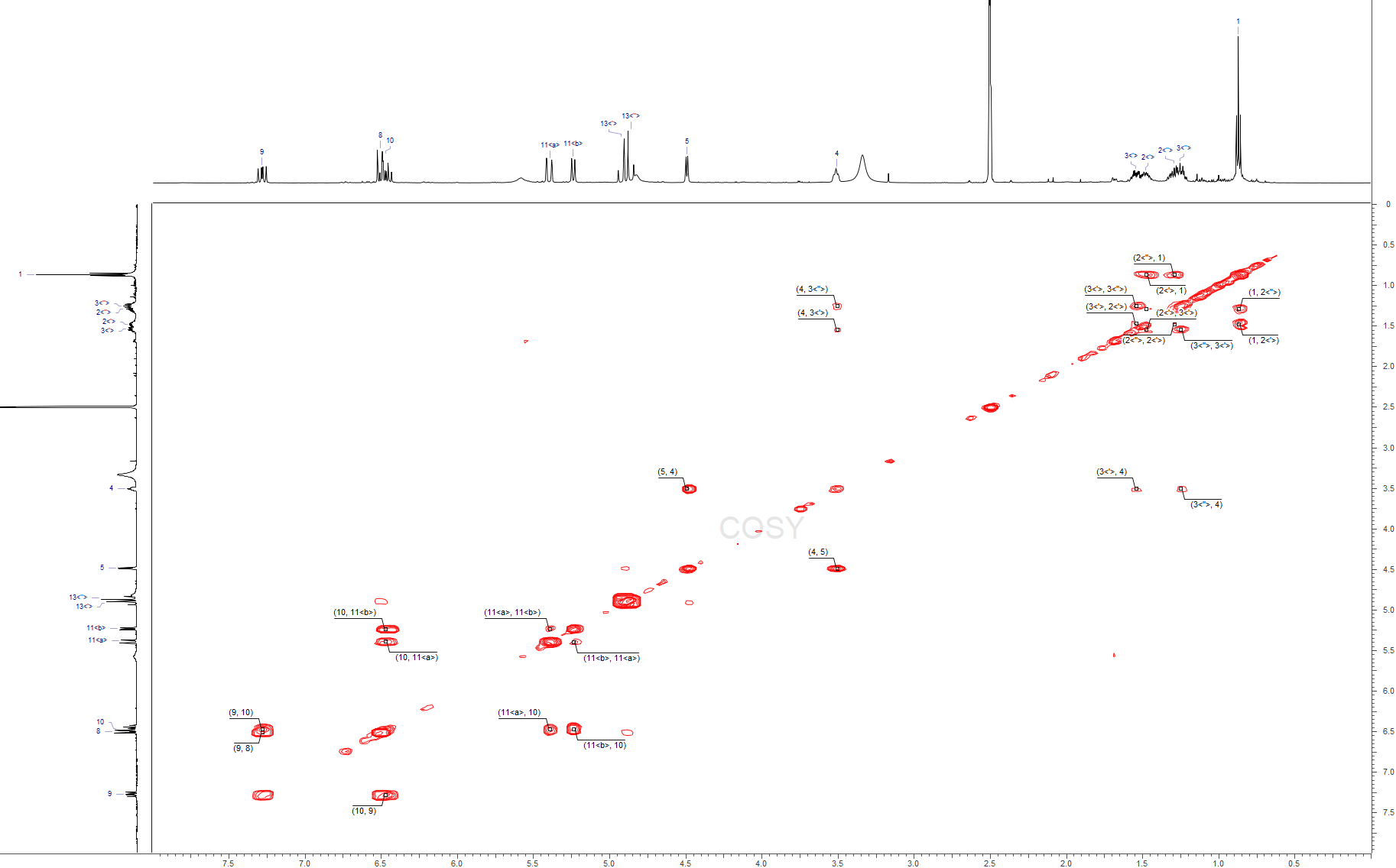


**Figure S6**. COSY NMR spectrum (500 MHz, DMSO-*d*_6_) of caliensolide A (**2**).


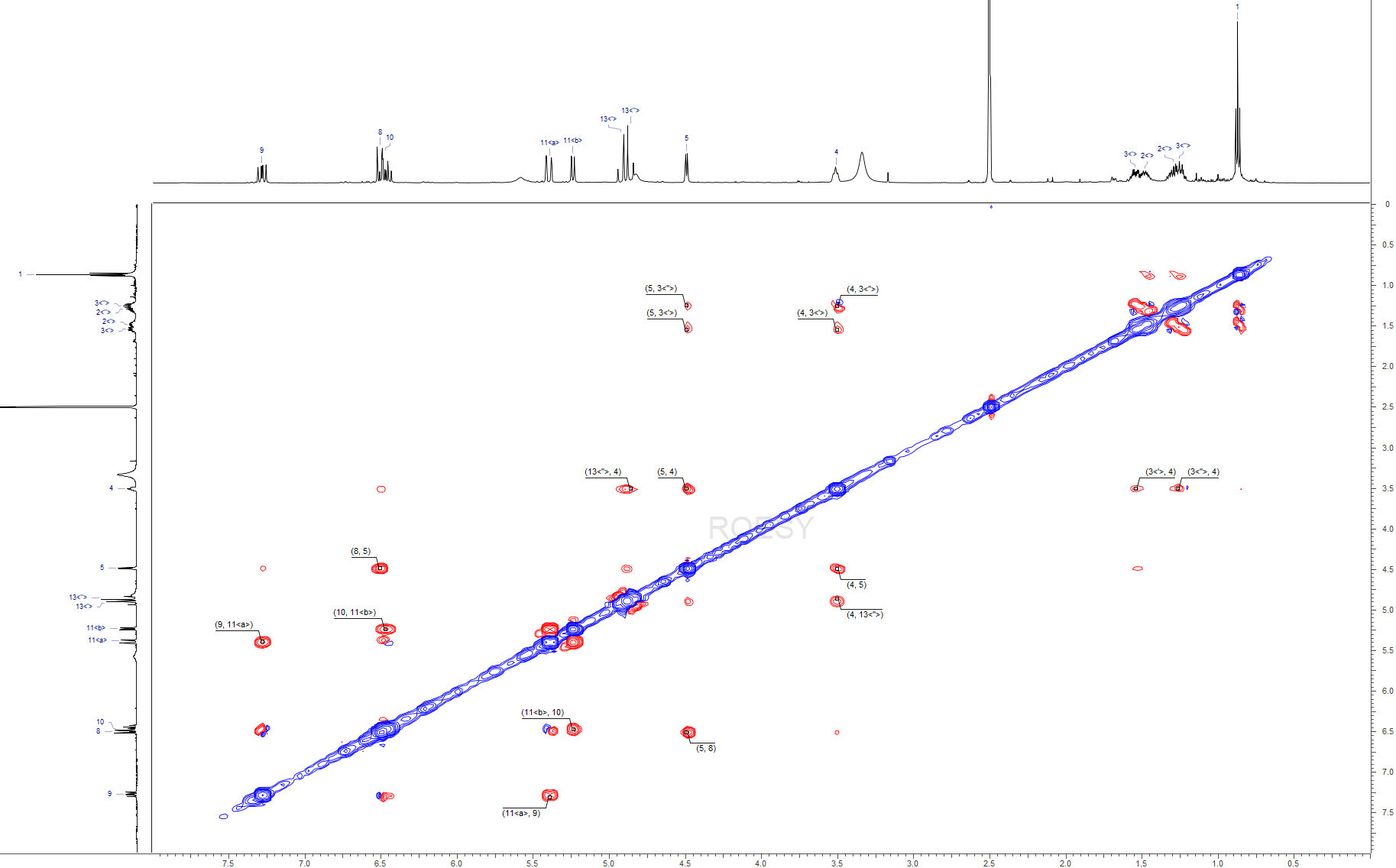


**Figure S7**. COSY NMR spectrum (500 MHz, DMSO-*d*_6_) of caliensolide A (**2**).


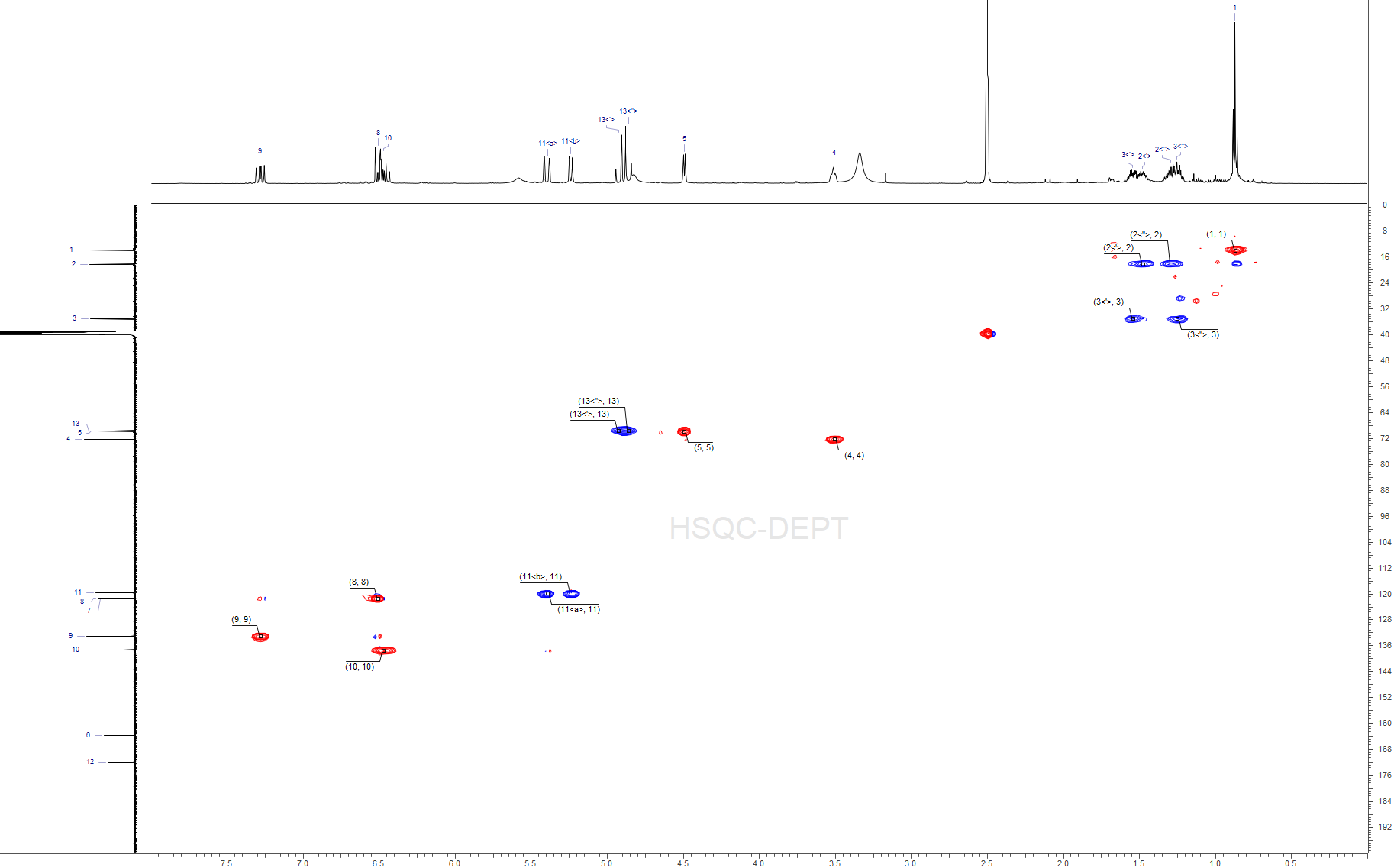


**Figure S8**. HSQC NMR spectrum (500 MHz, DMSO-*d*_6_) of caliensolide A (**2**).


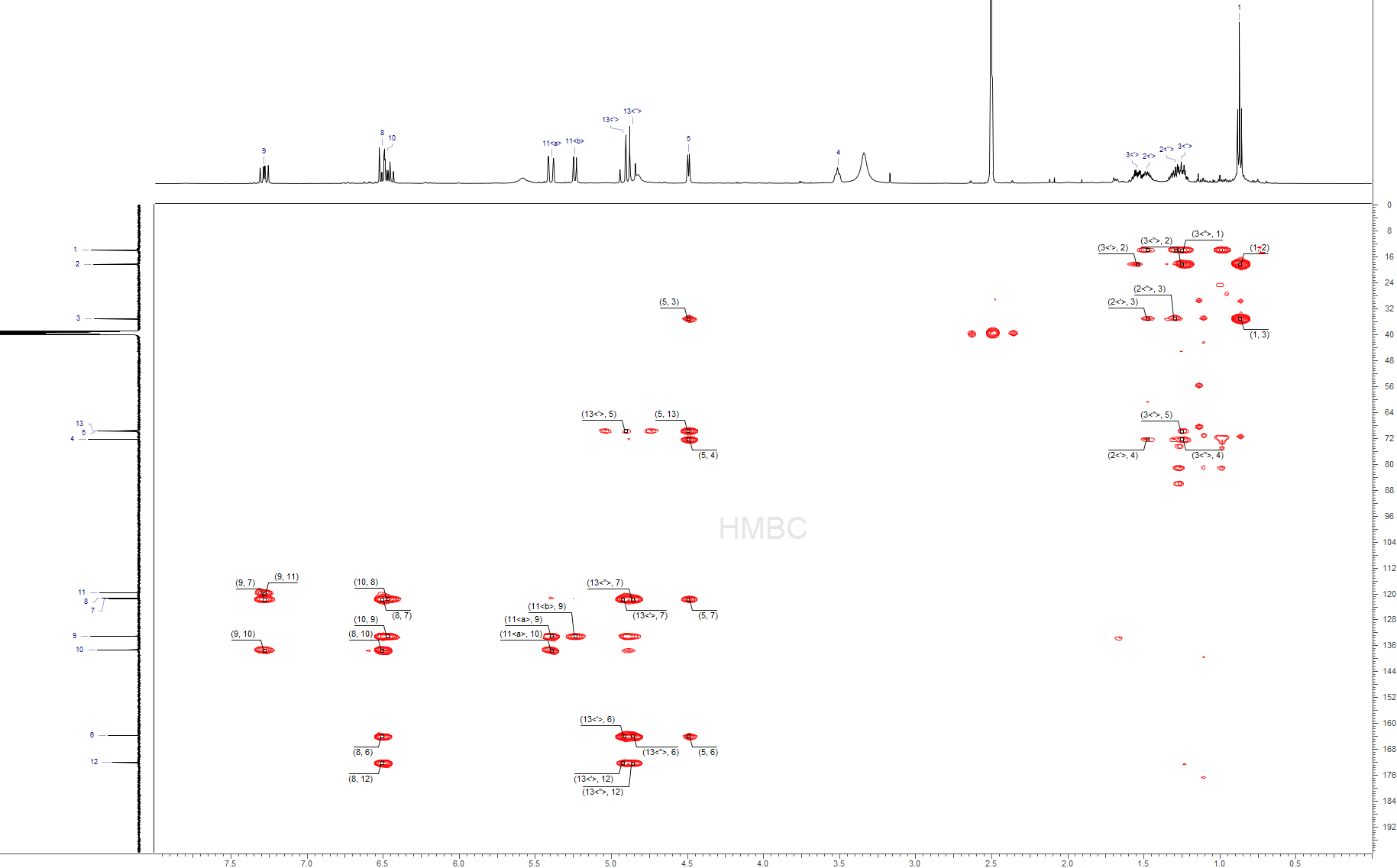


**Figure S9**. HMBC NMR spectrum (500 MHz, DMSO-*d*_6_) of caliensolide A (**2**).


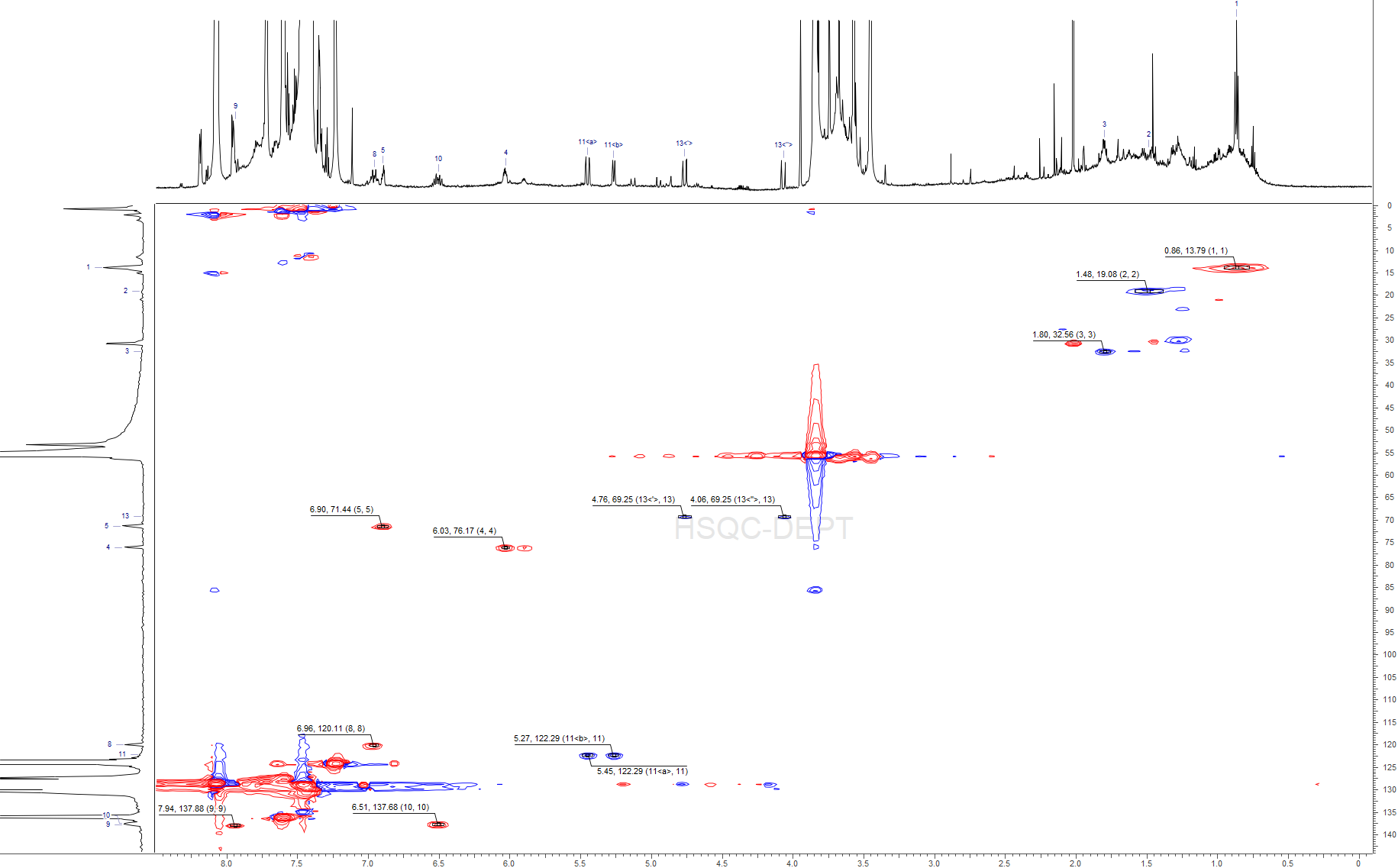


**Figure S10**. HSQC NMR spectrum (700 MHz, pyridin-*d*_5_) of the *S*-MTPA-derivative of caliensolide A (**2**).


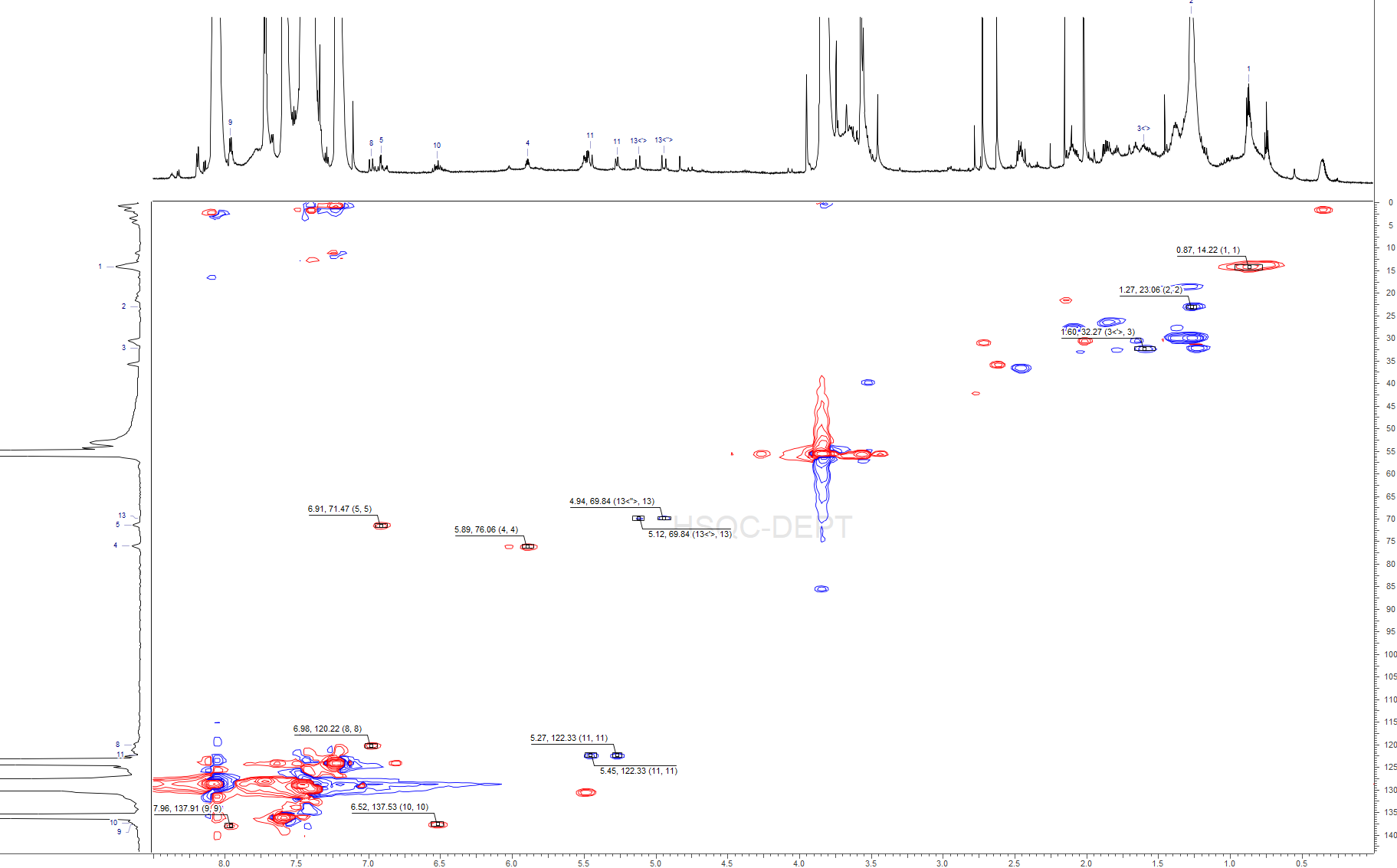


**Figure S11.** HSQC NMR spectrum (700 MHz, pyridin-*d*_5_) of the *R*-MTPA-derivative of caliensolide A (**2**).

**Figure S12**. ^1^H NMR spectrum (500 MHz, DMSO-*d*_6_) of caliensolide B (**3**).

**Figure S13**. ^13^C NMR spectrum (125 MHz, DMSO-*d*_6_) of caliensolide B (**3**).


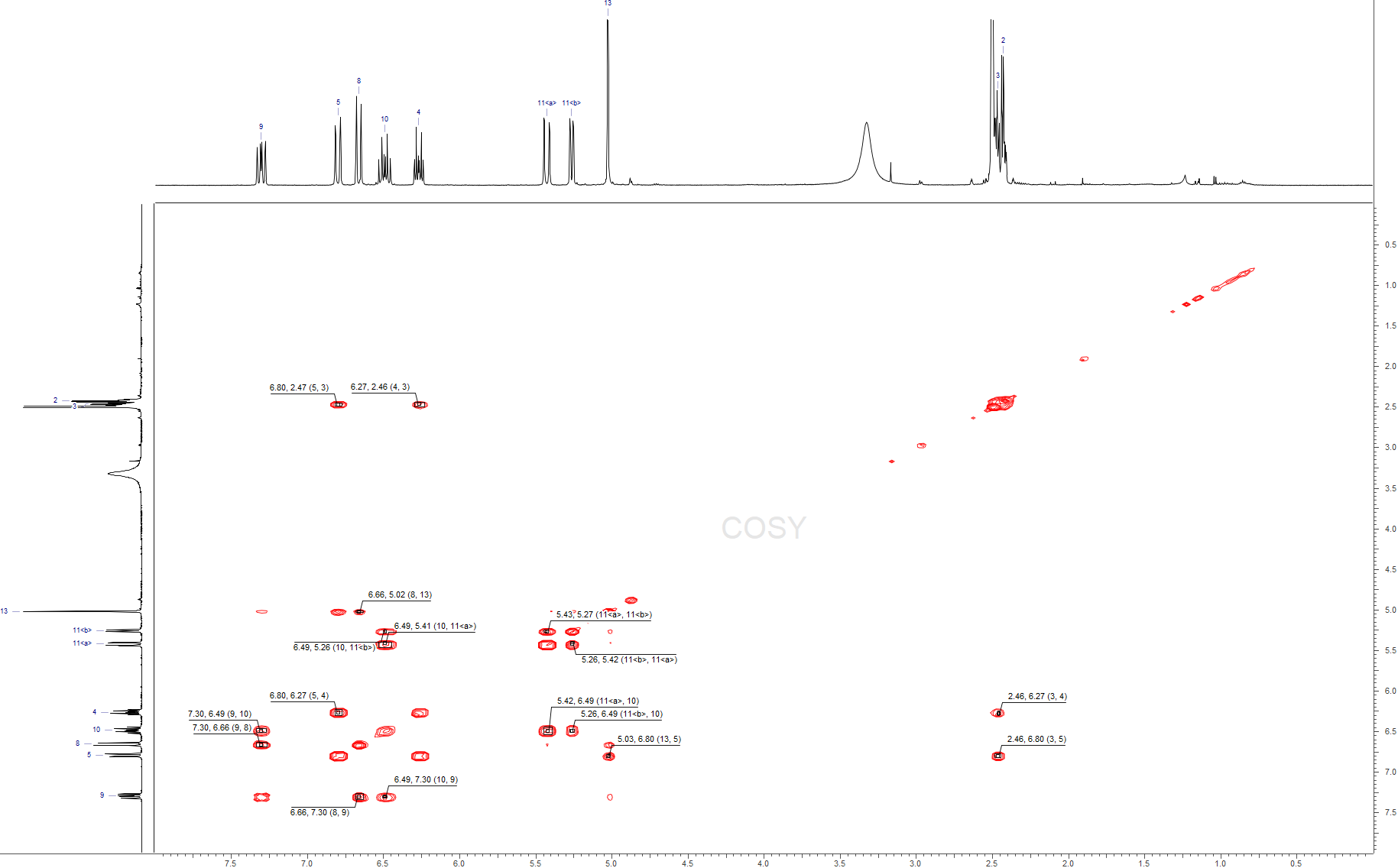


**Figure S14**. COSY NMR spectrum (500 MHz, DMSO-*d*_6_) of caliensolide B (**3**).


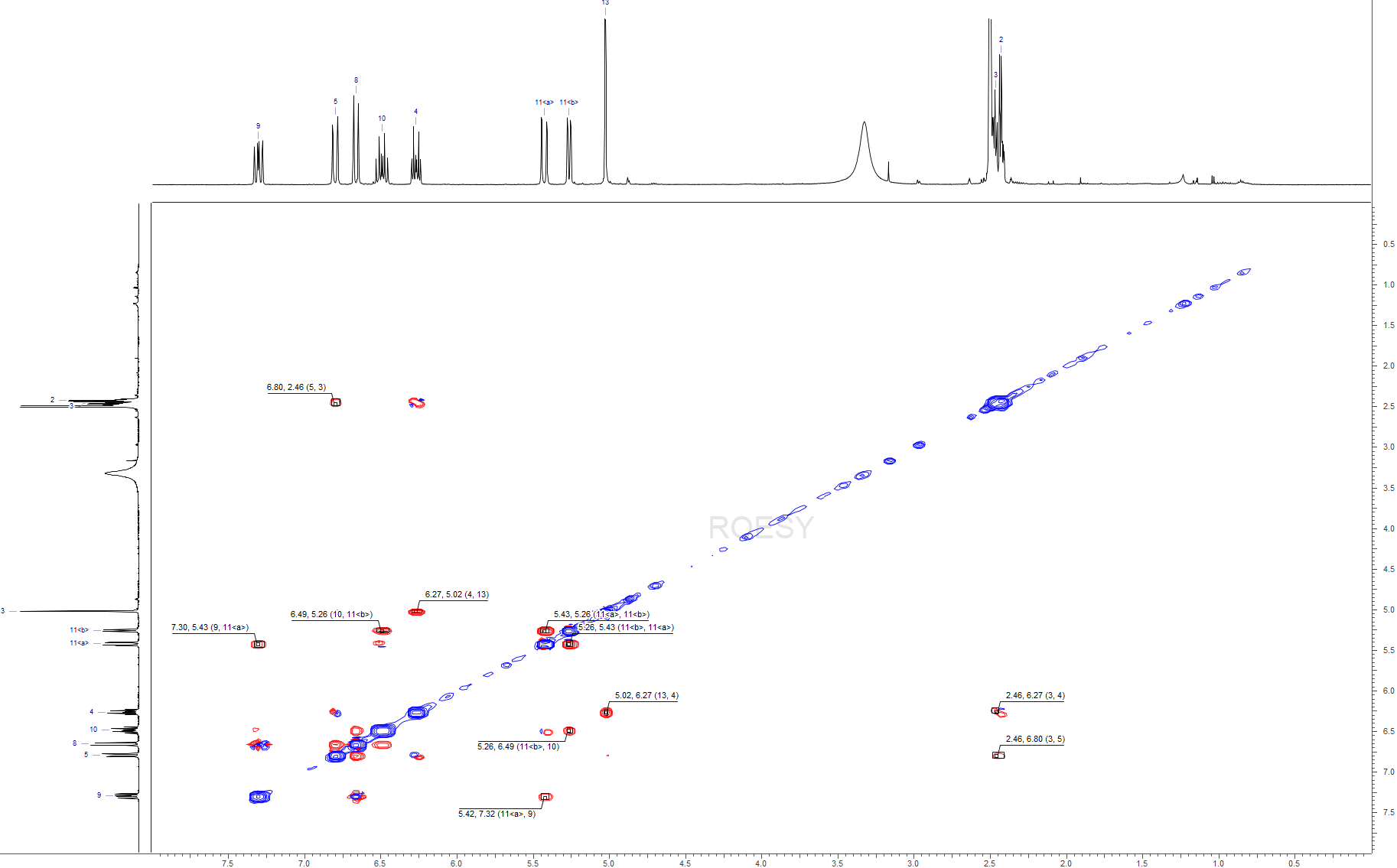


**Figure S15**. ROESY NMR spectrum (500 MHz, DMSO-*d*_6_) of caliensolide B (**3**).


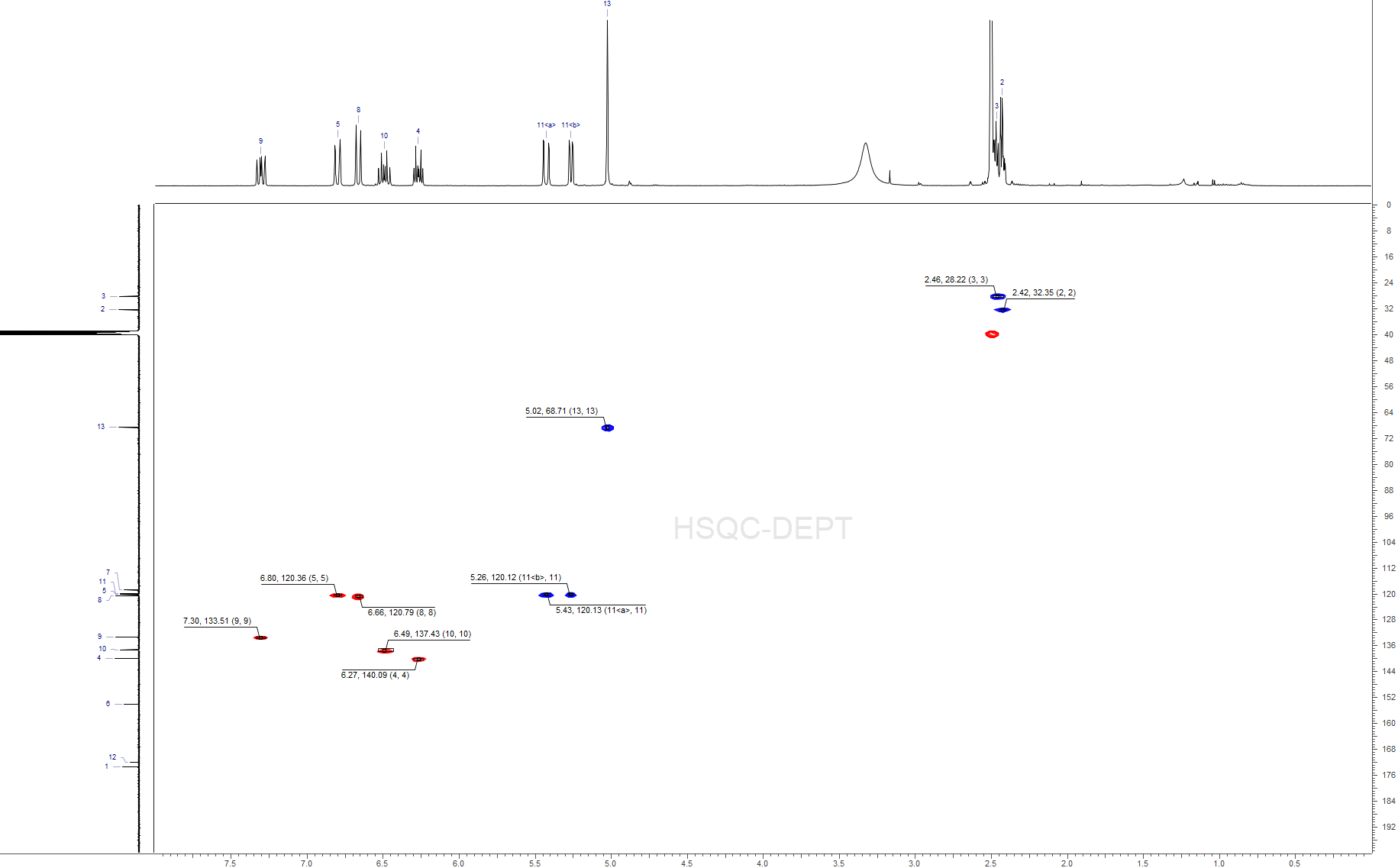


**Figure S16.** HSQC NMR spectrum (500 MHz, DMSO-*d*_6_) of caliensolide B (**3**).


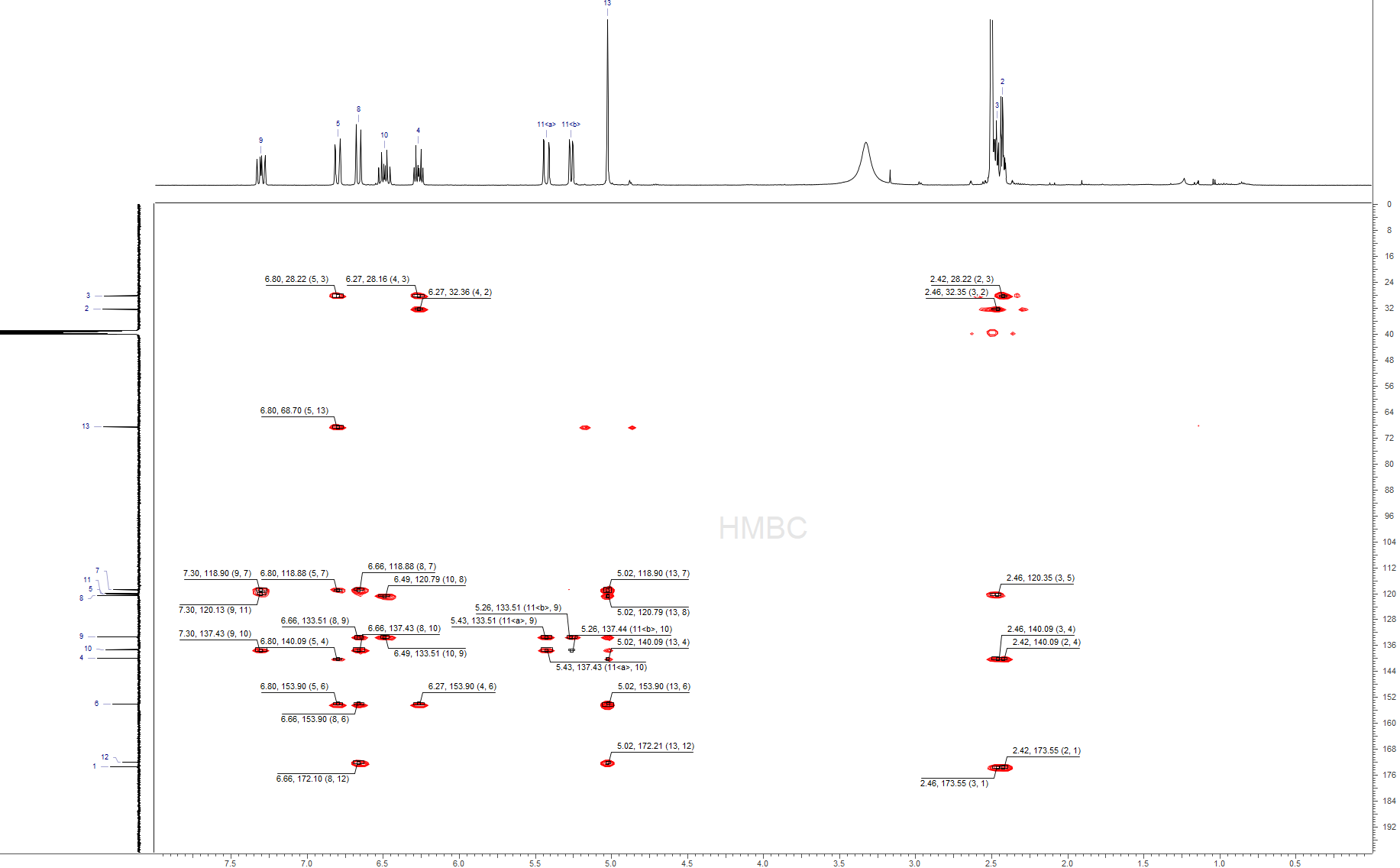


**Figure S17**. HMBC NMR spectrum (500 MHz, DMSO-*d*_6_) of caliensolide B (**3**).


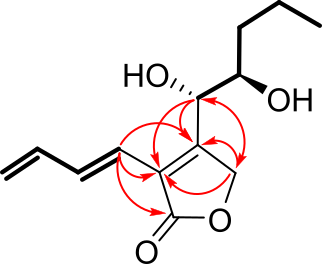


**Figure S18**. Key (―) ^1^H,^1^H COSY and (→) HMBC correlations of caliensolide A (**2**).


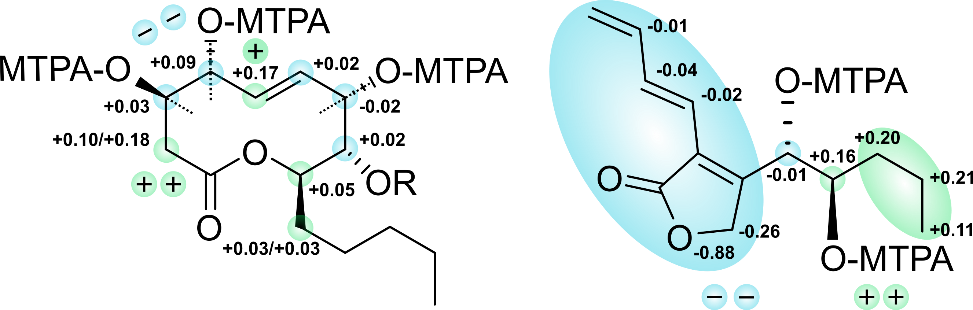


**Figure S19.** Δ*δ*^SR^ values for MPTA esters of phomol (**1**) diagnostic for 3*R*,4*R*,7*S*,8*S*,9*R*, and for caliensolide A (**2**) diagnostic for 4*R*,5*S* configuration.


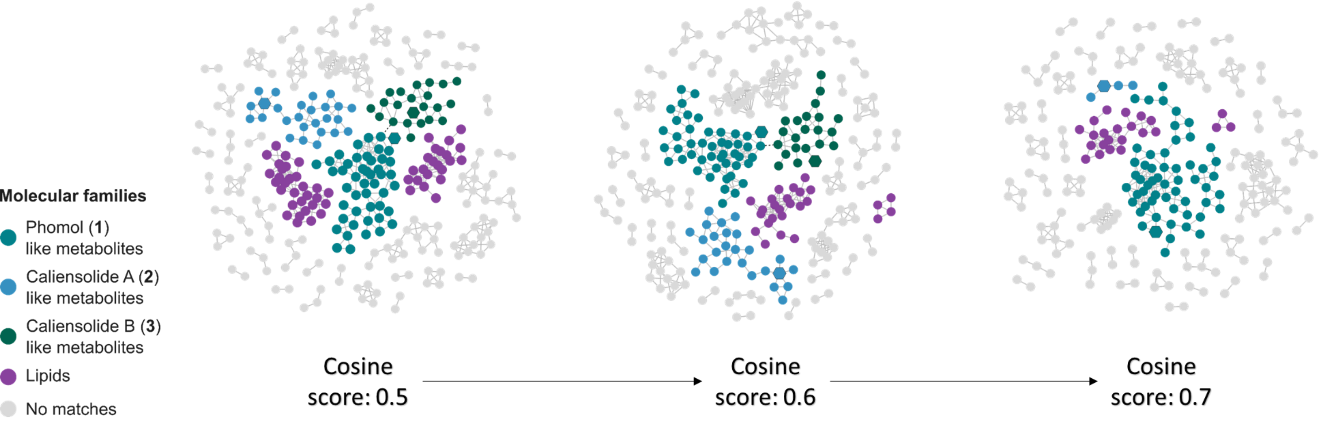


**Figure S20.** Molecular network topology analysis of the metabolites produced by *D. caliensis* sp. nov.


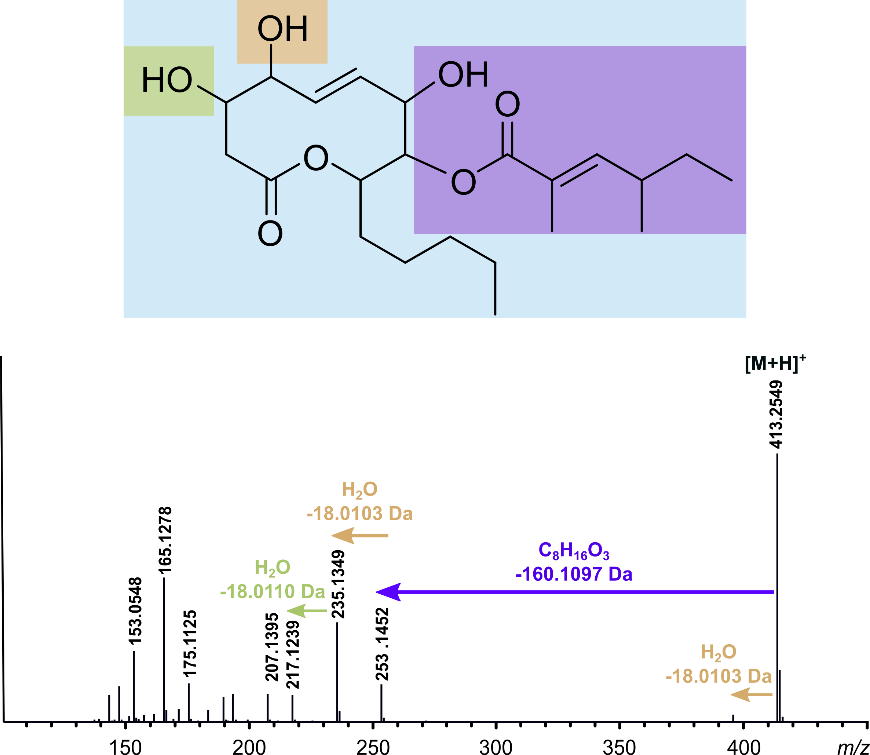


**Figure S21.** Structure of phomol (**1**) with highlighted observed fragments and its corresponding representative MS/MS spectrum.


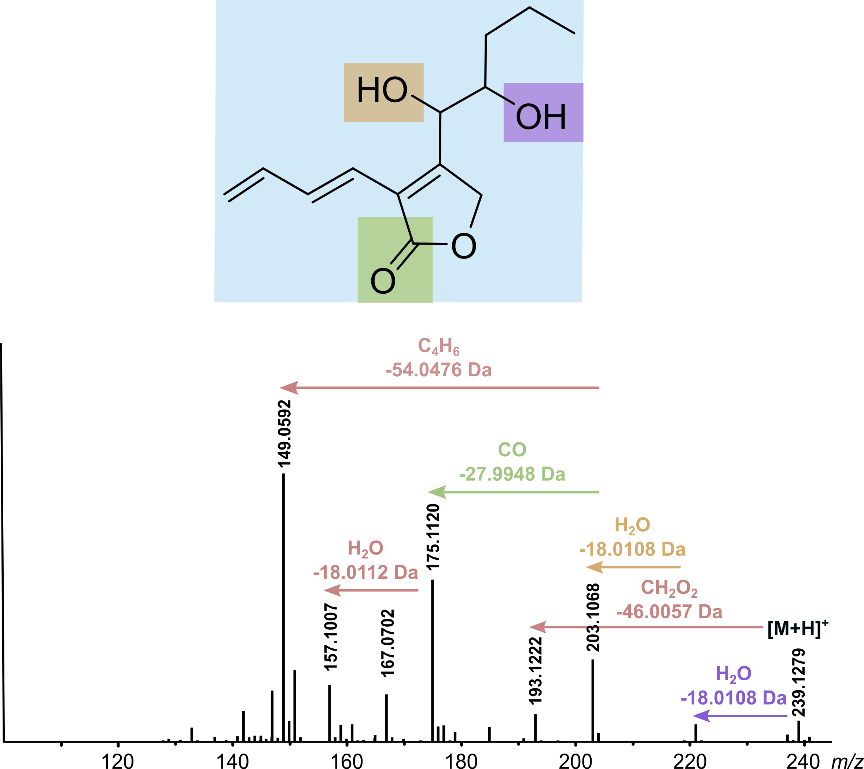


**Figure S22.** Structure of caliensolide A (**2**) with highlighted observed fragments and its corresponding representative MS/MS spectrum. Unassigned neutral losses are shown in red.


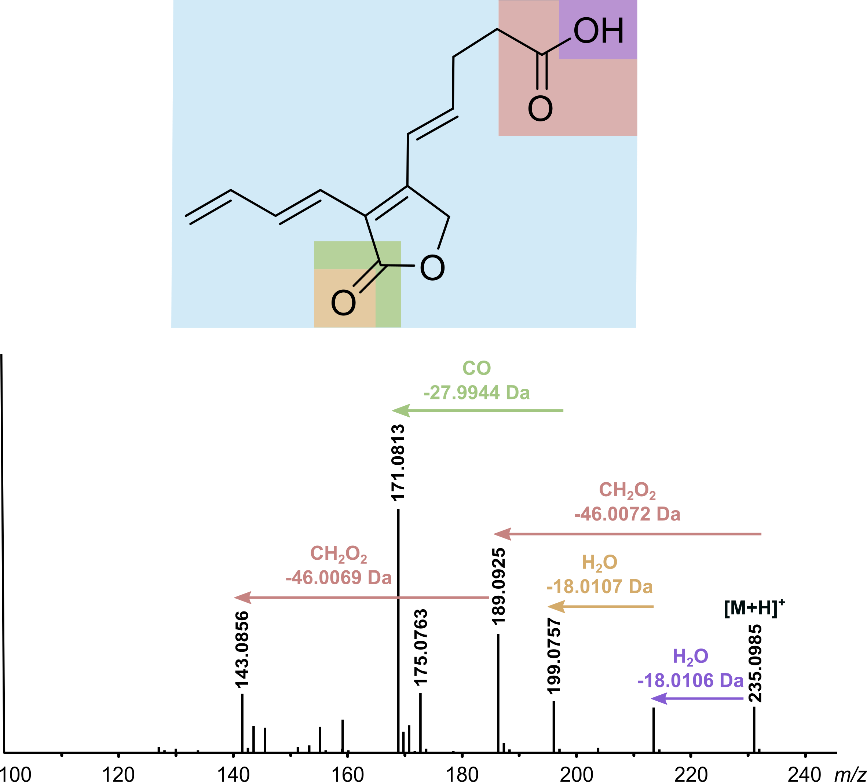


**Figure S23.** Structure of caliensolide B (**3**) with highlighted observed fragments and its corresponding representative MS/MS spectrum. Unassigned neutral losses are shown in red.

**Table S3**. Minimum inhibitory concentration (MIC, µM) of compounds **1**−**3** and the crude extract against bacterial and fungal test organisms. C ciprobay, G gentamicin, K kanamycin, O oxytetracycline, N nystatin, –: no inhibition observed under test conditions.

| **Test organism** | **Strain Number** | **1** | **2** | **3** | **Positive control** |
| --- | --- | --- | --- | --- | --- |
| *Candida albicans* | DSM 1665 | – | – | – | 8.96 ^N^ |
| *Schizosaccharomyces pombe* | DSM 70572 | – | – | – | 4.54 ^N^ |
| *Wickerhamomyces anomala* | DSM 6766 | – | – | – | 8.96 ^N^ |
| *Mucor hiemalis* | DSM 2656 | 80.73 | – | – | 4.54 ^N^ |
| *Mycolicibacterium smegmatis* | ATCC 700084 | 161.45 | – | – | 3.51 ^K^ |
| *Bacillus subtilis* | DSM 10 | 161.45 | – | – | 18.03 ^O^ |
| *Rhodotorula glutinis* | DSM 10134 | 161.45 | – | – | 2.27 ^N^ |
| *Staphylococcus aureus* | DSM 346 | 161.45 | – | – | 0.46 ^O^ |
| *Acinetobacter baumannii* | DSM 30008 | – | – | – | 0.78 ^C^ |
| *Chromobacterium violaceum* | DSM 30191 | – | – | – | 0.91 ^O^ |
| *Escherichia coli* | DSM 1116 | – | – | – | 7.17 ^O^ |
| *Pseudomonas aeruginosa* | DSM 19882 | – | – | – | 0.88 ^G^ |

ATCC: American Type Culture Collection, Manassas, VA, USA; DSM: Leibniz-Institute DSMZ—German Collection of Microorganisms and Cell Cultures GmbH, Braunschweig, Germany. C cibrobay, G gentamicin, K kanamycin, N nystatin, and O oxytetracycline. –: no inhibition observed under test conditions.

**Table S4**. Cytotoxicity of the isolated compounds against mammalian cell lines [half maximal inhibitory concentrations (IC_50_): µM].

| **Cell lines** | **Number ^1^** | **IC_50_ [µM]** | | | |
| --- | --- | --- | --- | --- | --- |
|  |  | **1** | **2** | **3** | **Epothilone B*** |
| HeLa KB 3.1 | ACC 158 | 18 | – | – | 0.000033 |
| Mouse fibroblast L929 | ACC 2 | 22 | – | – | 0.000033 |

–: no inhibition observed under test conditions. ^1^ ACC: Leibniz-Institute DSMZ—German Collection of Microorganisms and Cell Cultures GmbH, Braunschweig, Germany. * positive control (1 mg/mL).

**Table S5.** Alignment of the ITS sequences used in the phylogenetic study

>Diaporthe_acaciarum_CBS_138862

NNNNNNNNNCCGTTGGTGAACCAGCGGAGGGATCATTGCTGGAACGCG-CTTC-GGCGCA-CCCAGAAACCCTTTGTGAACTTATACCT--ACTGTTGCCTCGGCGTCAGGCCGGCCTCC--TC----------ACCGAGGCC-----CCCCG---GAGACGGGGAGCA-GCCCGCCGGCGGCCAA-CTAAACTCTTGTTTCTACAGTGAATCTCTGAGT--AAAAAACATAAATGAATCAAAACTTTCAACAACGGATCTCTTGGTTCTGGCATCGATGAAGAACGCAGCGAAATGCGATAAGTAATGTGAATTGCAGAATTCAGTGAATCATCGAATCTTTGAACGCACATTGCGCCCCCTGGTATTCCGGGGGGCATGCCTGTTCGAGCGTCATTTCAACCCTCAAGCCTGGCTTGG-TGATGGGGCACTACTCCCTC------GCGGGAGTAGGCCCTGAAATTCAGTGGCGAGCT-CGCCAGGAC-CCCGAGCGTAGTAG-TTATA-TCTCGCTTTGG-AAGGCCCTGGCGGTGCCCTGCCGTTAAA-CCCCCAACTTCTGAAAATT

>Diaporthe_acericola_MFLUCC_17_0956

NNNNNNNNNNNNNNNNNNNNNNNNNNNNNNNNNNNNNNNNNNNNNNNNNNNNNNNNNNNNNNNCCAGAACCCTTTGTG-ACTTATACCC--ACTGTTGCCTCGGCG-CAGGCCGGCCTCT--TC----------GCTGAGGCC-----CCCTG---GAAACAGGGAGCA-GCCCGCCGGTGGCCAA-CTAAACTC-TGTTTCTATAGTGAATCTCTGAGT--AAAAAACATAAATGAATCAAAACTTTCAACAACGGATCTCTTGGTTCTGGCATCGATGAAGAACGCAGCGAAATGCGATAAGTAATGTGAATTGCAGAATTCAGTGAATCATCGAATCTTTGAACGCACATTGCGCCCTCTGGTATTCCGGAGGGCATGCCTGTTCGAGCGTCATTTCAACCCTCAAGCCTGGCTTGG-TGATGGGGCACTGCCTGT--------AAAAGGGCAGGCCCTGAAATCTAGTGGCGAGCT-CGCCAGGAC-CCCGAGCGTAGTAG-TTATA-TCTCGCTTTGG-AAGGCCCTGGCGGTGCCCTGCCGTTAAA-CCCCCAACTTCTGAAAATT

>Diaporthe_alangii_CFCC_52556

NNNNNNNNNNNNNNNNNNNNNNNNNNNNNNNNNNNNNNNNNNNNNNNNNNNNNNNNNNNNNNNNNNNNNCCCTTTGTGAACTTATACCTATACTGTTGCCTCGGCG-CTGGCCGGCCTCC--TC----------ACCGAGGCC-----CCCTG---GAGACAGGGAGCA-GCCCGCCGGCGGCCAA-ACAAACTCTTGTTTCT-TAGTGAATCTCTGAGT--AAAAAACATAAATGAATCAAAACTTTCAACAACGGATCTCTTGGTTCTGGCATCGATGAAGAACGCAGCGAAATGCGATAAGTAATGTGAATTGCAGAATTCAGTGAATCATCGAATCTTTGAACGCACATTGCGCCCTCTGGTATTCCGGAGGGCATGCCTGTTCGAGCGTCATTTCAACCCTCAAGCCTGGCTTGG-TGTTGGGGCACCGCCTTTGC------AAAAGGGCGGGCCCTGAAATCTAGTGGCGAGCT-CGCCAGGAC-CCCGAGCGTAGTAG-TTATA-TCTCGTTCTGG-AAGGCCCTGGCGGTGCCCTGCCGTTAAA-CCCCCAACTTCTGAAANNN

>Diaporthe_ambigua_CBS_114015

ACAAGGTCTCCGTTGGTGAACCAGCGGAGGGATCATTGCTGGAACGCG-CCTC-GGCGCA-CCCAGAAACCCTTTGTGAACTTATACCT--ATCGTTGCCTCGGCG-AAGGCCGGCCTCC--CC----------ACCGAGGCC-----CCTTG---GGAACAAGGAGCA-GCCCGCCGGCGGCCAA-CCAAACTCTTGTTTCT-TAGTGAATCTCTGAGTAAAAAAAACATAAATGAATCAAAACTTTCAACAACGGATCTCTTGGTTCTGGCATCGATGAAGAACGCAGCGAAATGCGATAAGTAATGTGAATTGCAGAATTCAGTGAATCATCGAATCTTTGAACGCACATTGCGCCCTCTGGTATTCCGGAGGGCATGCCTGTTCGAGCGTCATTTCAACCCTCAAGCCTGGCTTGG-TGATGGGGCACTGCTTCCGA------GAGGGAGCAGGCCCTGAAATCTAGTGGCGAGCT-CGCCAGGAC-CCCGAGCGTAGTAG-TTATA-TCTCGCTCCGG-AAGGCCCTGGCGGTGCCCTGCCGTTAAA-CCCCCAACTTCTGAAAATT

>Diaporthe_amygdali_CBS_126679

ACAAGGTCTCCGTTGGTGAACCAGCGGAGGGATCATTGCTGGAACGCG-CCTC-GGCGCA-CCCAGAAACCCTTTGTGAACTTATACCT-TACTGTTGCCTCGGCG-CAGGCCGGCTCCC--AT----------CTGGGGGCC-----CCTCGTTTCTGACGAGGAGCAGGCTCGCCGGCGGCCAA-GTTAACTCTTGTTTTTAATTTGAAACTCTGAGA--ATAAAACATAAATGAATCAAAACTTTCAACAACGGATCTCTTGGTTCTGGCATCGATGAAGAACGCAGCGAAATGCGATAAGTAATGTGAATTGCAGAATTCAGTGAATCATCGAATCTTTGAACGCACATTGCGCCCTCTGGTATTCCGGAGGGCATGCCTGTTCGAGCGTCATTTCAACCCTCAAGCCTGGCTTGG-TGATGGGGCACTGCCTTTGTGTAAAAGCGAAGGCAGGCCCTGAAATTCAGTGGCGAGCT-CGCCAGGAC-TCCGAGCGCAGTAG-TTAAACCCTCGCTTTGG--AAGGACTGGCGGTGCCCTGCCGTTAAA-CCCCCAACTCTTGAAAATT

>Diaporthe_angelicae_CBS_111592

ACAAGGTCTCCGTTGGTGAACCAGCGGAGGGATCATTGCTGGAACGCG-CCTC-GGCGCA-CCCAGAAACCCTTTGTGAACTTATACCTATACTGTTGCCTCGGCG-CAGGCCGGCCTTT--CT--------CGGTAAAGGCC-----CCCTG---GAGACAGGGAGCA-GCCCGCCGGCGGCCAG-CCAAACTC-TGTTTCTATAGTGGATCTCTGAGT--AAAAAACATAAATGAATCAAAACTTTCAACAACGGATCTCTTGGTTCTGGCATCGATGAAGAACGCAGCGAAATGCGATAAGTAATGTGAATTGCAGAATTCAGTGAATCATCGAATCTTTGAACGCACATTGCGCCCTCTGGTATTCCGGAGGGCATGCCTGTTCGAGCGTCATTTCAACCCTCAAGCCTGGCTTGG-TGATGGGGCACTGCCTGT--------GAAAGGGCAGGCCCTGAAATCTAGTGGCGAGCT-CGCCAGGAC-CCCGAGCGCAGTAG-TTACA-TCTCGCTCTGG-GAGGCCCTGGCGGTGCCCTGCCGTTAAA-CCCCCAACTTCTGAAAATT

>Diaporthe_arctii_CBS_136_25

ACAAGGTCTCCGTTGGTGAACCAGCGGAGGGATCATTGCTGGAACGCG-CCTC-GGCGCA-CCCAGAAACCCTTTGTGAACTCATACCCATACTGTTGCCTCGGCG-CAGGCCGGCCTTT--TT--------CGACAAAGGCC-----CCCTG---GAGACAGGGAGCA-GCCCGCCGGCGGCCAA-CCAAACTC-TGTTTCTATAGTGGATCTCTGAGT--AAAAAACATAAATGAATCAAAACTTTCAACAACGGATCTCTTGGTTCTGGCATCGATGAAGAACGCAGCGAAATGCGATAAGTAATGTGAATTGCAGAATTCAGTGAATCATCGAATCTTTGAACGCACATTGCGCCCTCTGGTATTCCGGAGGGCATGCCTGTTCGAGCGTCATTTCAACCCTCAAGCCTGGCTTGG-TGATGGGGCACTGCCTGT--------GAAAGGGCAGGCCCTGAAATCTAGTGGCGAGCT-CGCCAGGAC-CCCGAGCGTAGTAG-TTACA-TCTCGCTCTGG-AAGGCCCTGGCGGTGCCCTGCCGTTAAA-CCCCCAACTTCTGAAAATT

>Diaporthe_arezzoensis_MFLU_19_2880

ACAAGGTCTCCGTTGGTGAACCAGCGGAGGGATCATTGCTGGAACGCG-CCCC-GGCGCA-CCCAGAAACCCTTTGTGAACTCATACCTAT-CTGTTGCCTCGGCG-CAGGCCGGCCCCC--TC----------ACAGGGGCC-----CCCCG---GAGACGGGGAGCA-GCCCGCCGGCGGCCAA-CCAAACTCTTGTTTCTACAGTGTATCTCTGAGA-GAAAAAACATAAATGAATCAAAACTTTCAACAACGGATCTCTTGGTTCTGGCATCGATGAAGAACGCAGCGAAATGCGATAAGTAATGTGAATTGCAGAATTCAGTGAATCATCGAATCTTTGAACGCACATTGCGCCCTCTGGTATTCCGGAGGGCATGCCTGTTCGAGCGTCATTTCAACCCTCAAGCCTGGCTTGG-TGATGGGGCACTGCCCGT--------AGAAGGGCAGGCCCTGAAATCTAGTGGCGAGCT-CGCCAGGAC-CCCGAGCGTAGTAG-TTATA-TCTCGCTCCGG-AAGGCCCTGGCGGTGCCCTGCCGTTAAA-CCCCCAACTTCTGAAAATT

>Diaporthe_batatas_CBS_122_21

ACAAGGTCTCCGTTGGTGAACCAGCGGAGGGATCATTGCTGGAACGCG-CCCCTGGCGCA-CCCAGAAACCCTTTGTGAACTTATACC---ACTGTTGCCTCGGCG-CAGGCCGGCCTCT--TA----------GCTGAGGCC-----CCCCG---GAGACGGGGAGCA-GCCCGCCGGCGGCCAA-CCAAACTCTTGTTTCTATAGTGAATCTCTGAGT--AAAAAACATAAATGAATCAAAACTTTCAACAACGGATCTCTTGGTTCTGGCATCGATGAAGAACGCAGCGAAATGCGATAAGTAATGTGAATTGCAGAATTCAGTGAATCATCGAATCTTTGAACGCACATTGCGCCCTCTGGTATTCCGGAGGGCATGCCTGTTCGAGCGTCATTTCAACCCTCAAGCCTGGCTTGG-TGATGGGGCACTGCCTCCCTC-----AGGGGGGCAGGCCCTGAAATCTAGTGGCGAGCT-CGCCAGGAC-CCCGAGCGTAGTAG-TTACA-TCTCGCTCTGG-AAGGCCCTGGCGGTGCCCTGCCGTTAAA-CCCCCAACTTCTGAAAATT

>Diaporthe_beilharziae_BRIP_54792

ACAAGGTCTCCGTTGGTGAACCAGCGGAGGGATCATTGCTGGAACGCG-CCTC-GGCGCA-CCCAGAAACCCTTTGTGAACTTATACCT--ATCGTTGCCTCGGCGTTCGGCCGGCCTCT--TC----------ACTGAGGCC-----CCCTG---GAAACGGGGAGCA-GCCCGCCGGCGGCCAA-CCAAACTCTTGTTTCTATAGTGAATCTCTGAGT--GAAAAACATAAATGAATCAAAACTTTCAACAACGGATCTCTTGGTTCTGGCATCGATGAAGAACGCAGCGAAATGCGATAAGTAATGTGAATTGCAGAATTCAGTGAATCATCGAATCTTTGAACGCACATTGCGCCCCCTGGTATTCCGGGGGGCATGCCTGTTCGAGCGTCATTTCAACCCTCAAGCCTGGCTTGG-TGATGGGGCACTACTTCCTC------ACGGGAGTAGGCCCTGAAATTCAGTGGCGAGCT-CGCCAGGAC-CCCGAGCGTAGTAG-TTACA-TCTCGCTCTGG-AAGGCCCTGGCGGTGCCCTGCCGTTAAA-CCCCCAACTTCTGAAAATT

>Diaporthe_biguttulata_ICMP20657

NNNNNNNNNNNNNNNNNNNNNNNNNNNNNNNNNNNNNNCTGGAACGCG-CCTC-GGCGCA-CCCAGAAACCCTTTGTGAACTTATACCT-TACTGTTGCCTCGGCG-CAGGCCGGCCTCT--TA----------GCTGAGGCC-----CCCCG---GAGACGGGGAGCA-GCCCGCCGGCGGCCAA-CCTAACTCTTGTTTTTACACTGTATCTCTGAGC-AAAAAAACATAAATGAATCAAAACTTTCAACAACGGATCTCTTGGTTCTGGCATCGATGAAGAACGCAGCGAAATGCGATAAGTAATGTGAATTGCAGAATTCAGTGAATCATCGAATCTTTGAACGCACATTGCGCCCTCTGGTATTCCGGAGGGCATGCCTGTTCGAGCGTCATTTCAACCCTCAAGCCTGGCTTGG-TGTTGGGGCACTGCTTCG--------AGAGAAGCAGGCCCTGAAATCTAGTGGCGAGCT-CGCCAGGAC-CCCGAGCGTAGTAG-TTATA-TCTCGTTCTGG-AAGGCCCTGGCGGTGCCCTGCCGTTAAA-CCCCCAACTTCTGAAAATT

>Diaporthe_breyniae

ACAAGGTCTCCGTTGGTGAACCAGCGGAGGGATCATTGCTGGAACGCG-CTTC-GGCGCA-CCCAGAAACCCTTTGTGAACTTATACCTAT--TGTTGCCTCGGCG-TAGGCCGGCCTCT--TC----------ACTGAGGCC-----CCCTG---GAGACAGGGAGCA-GCCCGCCGGCGGCCAA-CTAAACTCTTGTTTCTATAGTGAATCTCTGAGT---AAAAACATAAATGAATCAAAACTTTCAACAACGGATCTCTTGGTTCTGGCATCGATGAAGAACGCAGCGAAATGCGATAAGTAATGTGAATTGCAGAATTCAGTGAATCATCGAATCTTTGAACGCACATTGCGCCCTCTGGTATTCCGGAGGGCATGCCTGTTCGAGCGTCATTTCAACCCTCAAGCCTGGCTTGG-TGATGGGGCACTGCTCTCT------AGCGGGAGCAGGCCCTGAAATCTAGTGGCGAGCT-CGCCAGGAC-CCCGAGCGTAGTAG-TTATA-TCTCGTTCTGG-AAGGCCCTGGCGGTGCCCTGCCGTTAAA-CCCCCAACTTCTGAAAATT

>Diaporthe_camporesii_JZB320143

NNNNNNNNNNNNNNCCCTCGCGACGCGGAGGGACATTGCTGG-ACGCG-CTTC-GGCGCA-CCCAGAAACCCTTTGTGAACTTATACCT--ATTGTTGCCTCGGCG-TAGGCCGGCCTCT--TC----------ACTGAGGCC-----CCCTG---GAAACAGGGAGCA-GCCCGCCGGCGGCCAA-CCAAACTCTTGTTTCTACAGTGAATCTCTGAGT--AAAAAACATAAATGAATCAAAACTTTCAACAACGGATCTCTTGGTTCTGGCATCGATGAAGAACGCAGCGAAATGCGATAAGTAATGTGAATTGCAGAATTCAGTGAATCATCGAATCTTTGAACGCACATTGCGCCCTCTGGTATTCCGGAGGGCATGCCTGTTCGAGCGTCATTTCAACCCTCAAGCCTGGCTTGG-TGATGGGGCACTGCTTTCGTC-----CAGAAAGCAGGCCCTGAAATCTAGTGGCGAGCT-CGCCAGGAC-CCCGAGCGTAGTAG-TTATA-TCTCGCTCCGG-AAGGCCCTGGCGGTGCCCTGCCGTTAAA-CCCCCAACTTCTGAAAATT

>Diaporthe_caryae_CFCC_52563

NNNNNNNNNNNNNNNNNNNNNNNNNNNNNNNNNNNNNNNNNNNNNNNNNNNNNNNNNNNNNNNNNNNNNCCCTTTGTGAACTTATACCT--ACTGTTGCCTCGGCGTCAGGCCGGCCCCT--TC----------ACTGGGGCC-----CCCCG---GAGACGGGGAGCA-GCCCGCCGGCGGCCAA-CTAAACTCTTGTTTCTATAGTGAATCTCTGAGT--AAAAAACATAAATGAATCAAAACTTTCAACAACGGATCTCTTGGTTCTGGCATCGATGAAGAACGCAGCGAAATGCGATAAGTAATGTGAATTGCAGAATTCAGTGAATCATCGAATCTTTGAACGCACATTGCGCCCCCTGGTATTCCGGGGGGCATGCCTGTTCGAGCGTCATTTCAACCCTCAAGCCTGGCTTGG-TGATGGGGCACTGCTTCG--------AGAGGAGCAGGCCCTGAAATTCAGTGGCGAGCT-CGCCAGGAC-CCCGAGCGTAGTAG-TTATA-TCTCGCTTTGG-AAGGCCCTGGCGGTGCCCTGCCGTTAAA-CCCCCAACTTCTGAAANNN

>Diaporthe_celtidis_NCYU_19_0357

NNNNNNNNTCCGTTGGTGAACCAGCGGAGGGA-CATTGCTGGAACGCG-CCCC-GGCGCA-CCCAGAAACCCTTTGTGAACTTATACCTATACCGTTGCCTCGGCG-CTGGCCGGCCCCC--TC----------ACCGGGGCC-----CCCTG---GAGACAGGGAGCA-GCCCGCCGGCGGCCAG-ACAAACTCTTGTTTCT-TAGTGGATCTCTGAGT--AAAAAACAT-AATGAATCAAAACTTTCAACAACGGATCTCTTGGTTCTGGCATCGATGAAGAACGCAGCGAAATGCGATAAGTAATGTGAATTGCAGAATTCAGTGAATCATCGAATCTTTGAACGCACATTGCGCCCCCTGGTATTCCGGGGGGCATGCCTGTTCGAGCGTCATTTCACCCCTCAAGCCTGGCTTGG-TGTTGGGGCACCGCCCGTA--------AAAGGGCGGGCCCTTAAATCCAGCGGCGAGCC-AGAGGAAACGGCCTTGCGTAGTAG-TTCTA-TCTCGCATGTC-CTGCCCTCA--AGCGCCCTGCCGTTAAA-CCCCANNNNNNNNNNNNNN

>Diaporthe_cerradensis_CMRP4331

ACAAGGTCTCCGTTGGTGAACCAGCGGAGGGATCATTGCTGGAACGCG-CTTC-GGCGCA-CCCAGAAACCCTTTGTGAACTTATACCT-TACTGTTGCCTCGGCG-CAGGCCGGCCCCT--CC----------CACGGGGCC-----CCTCC---GGAAGGAGGAGCA-GCCCGCCGGCGGCCAA-CTAAACTCTTGTTTCT-TAGTGAATCTCTGAGT--AAAAAACATAAATGAATCAAAACTTTCAACAACGGATCTCTTGGTTCTGGCATCGATGAAGAACGCAGCGAAATGCGATAAGTAATGTGAATTGCAGAATTCAGTGAATCATCGAATCTTTGAACGCACATTGCGCCCTCTGGTATTCCGGAGGGCATGCCTGTTCGAGCGTCATTTCAACCCTCAAGCACTGCTTGG-TGTTGGGGCACCGCCTGTG--------AAAGGGCGGGCCCTGAAAACTAGTGGCGAGCT-CGCCAGGAC-CCCGAGCGTAGTAGTTTATA-TCTCGTTCTGG-AAGGCCCTGGCGGTGCACTGCCGTTAAA-CCCCCAACTTCTGAAATTT

>Diaporthe_chimonanthi

NNNNNNNNNNNNNNNTGTGACCAGCGGAGGGATCATTGCTGGAACGCG-CTTC-GGCGCA-CCCAGAAACCCTTTGTGAACTTATACCT--ATTGTTGCCTCGGCGTCAGGCCGGCCTCT--TC----------ACTGAGGCC-----CCCCG---GAGACGGGGAGCA-GCCCGCCGGCGGCCAA-CTAAACTCTTGTTTCTATAGTGAATCTCTGAGT--AAAAAACATAAATGAATCAAAACTTTCAACAACGGATCTCTTGGTTCTGGCATCGATGAAGAACGCAGCGAAATGCGATAAGTAATGTGAATTGCAGAATTCAGTGAATCATCGAATCTTTGAACGCACATTGCGCCCCCTGGTATTCCGGGGGGCATGCCTGTTCGAGCGTCATTTCAACCCTCAAGCCTGGCTTGG-TGATGGGGCACTGCTTCG--------AAAGGAGCAGGCCCTGAAATTCAGTGGCGAGCT-CGCCAGGAC-CCCGAGCGTAGTAG-TTATA-TCTCGCTTTGG-AAGGCCCTGGCGGTGCCCTGCCGTTAAA-CCCCCAACTTCTGAAAATT

>Diaporthe_chinensis_MFLUCC_19_0101

NNNNNNNNTCCGTTGGTGAACCAGCGGAGGGATCATTGCTGGAACGCG-CTTC-GGCGCA-CCCAGAAACCCTTTGTGAACTTATACCT-TACTGTTGCCTCGGCG-CAGGCCGGCCTCT--AC---------TGCTGAGGCC-----CCCCG---GAGACGGGGAGCA-GCCCGCCGGCGGCCAA-CCAAACTCTTGTTTCTACAGTGAATCTCTGAGT--AAAAAACATAAATGAATCAAAACTTTCAACAACGGATCTCTTGGTTCTGGCATCGATGAAGAACGCAGCGAAATGCGATAAGTAATGTGAATTGCAGAATTCAGTGAATCATCGAATCTTTGAACGCACATTGCGCCCTCTGGTATTCCGGAGGGCATGCCTGTTCGAGCGTCATTTCAACCCTCAAGCCTGGCTTGG-TGTTGGGGCACTGCCTGT--------AAAAGGGCAGGCCCTGAAATCTAGTGGCGAGCT-CGCTAGGAC-CCCGAGCGTAGTAG-TTATA-TCTCGTTCTGG-AAGGCCCTGGCGGTGCCCTGCCGTTAAA-CCCCCNNNNNNNNNNNNNN

>Diaporthe_chromolaenae_MFLUCC_17_1422

ACAAGGTCTCCGTTGGTGAACCAGCGGAGGGATCATTGCTGGAACGCG-CTTC-GGCGCA-CCCAGAAACCCTTTGTGAACTTATACCTAT--TGTTGCCTCGGCG-CAGGCCGGCCTCT--TC----------ACTGAGGCC-----CCCTG---GAAACAGGGAGCA-GCCCGCCGGCGGCCAA-CCAAACTCTTGTTTCTATAGTGAATCTCTGAGT-AAAAAAACATAAATGAATCAAAACTTTCAACAACGGATCTCTTGGTTCTGGCATCGATGAAGAACGCAGCGAAATGCGATAAGTAATGTGAATTGCAGAATTCAGTGAATCATCGAATCTTTGAACGCACATTGCGCCCTCTGGTATTCCGGAGGGCATGCCTGTTCGAGCGTCATTTCAACCCTCAAGCCTGGCTTGG-TGATGGGGCACTGCCTGTA--------ATAGGGCAGGCCCTGAAATCTAGTGGCGAGCT-CGCCAGGAC-CCCGAGCGTAGTAG-TTATA-TCTCGCTCTGG-AAGGCCCTGGCGGTGCCCTGCCGTTAAA-CCCCCAACTTCTGAAAATT

>Diaporthe_cichorii_MFLUCC_17_1023

NNNNNNNNNNNNNNNNNNNNNNNNNNNNNNNNNNNNNNNNNNNNNNNNNNNNNNNNNNNNNNNCCAGAACCCTTTGTGAACTTATACCCATACTGTTGCCTCGGCG-CAGGCCGGCCCCT--GC---------TGCAGGGGCC-----CCCTG---GGGACAGGGAGCA-GCCCGCCGGCGGCCAA-CCAAACTC-TGTTTCTATAGTGGATCTCTGAGT-AAAAAAACATAAATGAATCAAAACTTTCAACAACGGATCTCTTGGTTCTGGCATCGATGAAGAACGCAGCGAAATGCGATAAGTAATGTGAATTGCAGAATTCAGTGAATCATCGAATCTTTGAACGCACATTGCGCCCTCTGGTATTCCGGAGGGCATGCCTGTTCGAGCGTCATTTCAACCCTCAAGCCTGGCTTGG-TGATGGGGCACTGCCTGT--------GAAAGGGCAGGCCCTGAAATCTAGTGGCGAGCT-CGCCAGGAC-CCCGAGCGTAGTAG-TTACA-TCTCGCTCTGG-AAGGCCCTGGCGGTGCCCTGCCGTTAAA-CCCCCAACTTCTGAAAATT

>Diaporthe_cinnamomi_CFCC_52569

NNNNNNNNNNNNNNNNNNNNNNNNNNNNNNNNNNNNNNNNNNNNNNNNNNNNNNNNNNNNNNNNNNNNNCCCTTTGTGAACTTATACCT-TACTGTTGCCTCGGCG-CAGGCCGGCCTCT--TA----------GCTGAGGCC-----CCCCG---GAGACGGGGAGCA-GCCCGCCGGCGGCCAA-GTTAACTCTTGTTTCTACCCTGAATCTCTGAGT--AAAAAACATAAATGAATCAAAACTTTCAACAACGGATCTCTTGGTTCTGGCATCGATGAAGAACGCAGCGAAATGCGATAAGTAATGTGAATTGCAGAATTCAGTGAATCATCGAATCTTTGAACGCACATTGCGCCCTCTGGTATTCCGGAGGGCATGCCTGTTCGAGCGTCATTTCAACCCTCAAGCCTGGCTTGG-TGCTGGGGCACTGCTTCG--------AGAGAAGCAGGCCCTGAAATCTAGTGGCGAGCT-CGCTAGGAC-CCCGAGCGTAGTAA-TTATA-TCTCGTTCTGG-AAGGCCCTGGCGGTGCCCTGCCGTTAAA-CCCCCAACTTCTGAAANNN

>Diaporthe_citriasiana_CBS_134240

ACAAGGTCTCCGTTGGTGAACCAGCGGAGGGATCATTGCTGGAACGCG-CCCC-GGCGCA-CCCAGAAACCCTTTGTGAACTCATACCT-TACTGTTGCCTCGGCG-CAGGCCGGCCTCT--AC---------TGCTGAGGCC-----CCCCG---GGGACGGGGAGCA-GCCCGCCGGCGGCCAA-GCCAACTCTTGTTTCTACAGTGAATCTCTGAGC--AAAAAACATAAATGAATCAAAACTTTCAACAACGGATCTCTTGGTTCTGGCATCGATGAAGAACGCAGCGAAATGCGATAAGTAATGTGAATTGCAGAATTCAGTGAATCATCGAATCTTTGAACGCACATTGCGCCCTCTGGTATTCCGGAGGGCATGCCTGTTCGAGCGTCATTTCAACCCTCAAGCCTGGCTTGG-TGCTGGGGCACTGCTCCG--------AGAGGAGCAGGCCCTGAAATCTAGTGGCGAGCT-CGCCAGGAC-CCCGAGCGCAGTAG-TTACA-TCTCGTTCTGG-AAGGCCCTGGCGGTGCCCTGCCGTTAAA-CCCCCAACTTCTGAAATTT

>Diaporthe_compacta_LC3083

NNNNNNNNNNNNNNNNNNNNNNNNNNNNGGGATCATTGCTGGAACGCG-CCTC-GGCGCA-CCCAGAAACCCTTTGTGAACTTATACCC--ACTGTTGCCTCGGCG-CAGGCCGGTCTGC---------------CTCAGACC-----CCCTG---GAGACAGGGAGCA-GCCCGCCGGCGGCCAA-CCAAACTC-TGTTTCTATAGTGAATCTCTGAGT--AAAAAACATAAATGAATCAAAACTTTCAACAACGGATCTCTTGGTTCTGGCATCGATGAAGAACGCAGCGAAATGCGATAAGTAATGTGAATTGCAGAATTCAGTGAATCATCGAATCTTTGAACGCACATTGCGCCCTCTGGTATTCCGGAGGGCATGCCTGTTCGAGCGTCATTTCAACCCTCAAGCCTGGCTTGG-TGATGGGGCAGTGCCTTGGA------GACAAGGCACGCCCTGAAATTCAGTGGCGAGCT-CGCCAGGAC-CCCGAGCGTAGTAG-TTACA-TCTCGCTCTGG-AAGGCCCTGGCGGTGCCCTGCCGTTAAA-CCCCCAACTTCTGAAANNN

>Diaporthe_convolvuli_CBS_124654

ACAAGGTCTCCGTTGGTGAACCAGCGGAGGGATCATTGCTGGAACGCG-CCTC-GGCGCA-CCCAGAAACCCTTTGTGAACTTATACCTATACTGTTGCCTCGGCG-CAGGCCGGCCTCC--CC----------ACCGAGGCC-----CCCTG---GAGACAGGGAGCA-GCCCGCCGGCGGCCAA-CCAAACTCTTGTTTCTACAGTGGATCTCTGAGT--AAAAAACATAAATGAATCAAAACTTTCAACAACGGATCTCTTGGTTCTGGCATCGATGAAGAACGCAGCGAAATGCGATAAGTAATGTGAATTGCAGAATTCAGTGAATCATCGAATCTTTGAACGCACATTGCGCCCTCTGGTATTCCGGAGGGCATGCCTGTTCGAGCGTCATTTCAACCCTCAAGCCTGGCTTGG-TGATGGGGCGCTGCCTGTA-------AGACGGGCAGGCCCTGAAATCTAGTGGCGAGCT-CGCCAGGAC-CCCGAGCGTAGTAG-TTATA-TCTCGCTCCGGAAAGGCCCTGGCGGTGCCCTGCCGTTAAA-CCCCCAACTTCTGAAAATT

>Diaporthe_cucurbitae_DAOM_42078

ACAAGGTCTCCGTTGGTGAACCAGCGGAGGGATCATTGCTGGAACGCG-CCCC-GGCGCA-CCCAGAAACCCTTTGTGAACTTATACCCATACTGTTGCCTCGGCG-CAGGCCGGCCTTT--TT--------CGATAGAGGCC-----CCCTG---GAGACAGGGAGCA-GCCCGCCGGCGGCCAA-CCAAACTC-TGTTTCTATAGTGAATCTCTGAGT--AAAAAACATAAATGAATCAAAACTTTCAACAACGGATCTCTTGGTTCTGGCATCGATGAAGAACGCAGCGAAATGCGATAAGTAATGTGAATTGCAGAATTCAGTGAATCATCGAATCTTTGAACGCACATTGCGCCCTCTGGTATTCCGGAGGGCATGCCTGTTCGAGCGTCATTTCAACCCTCAAGCCTGGCTTGG-TGATGGGGCACTGCCTGT--------GAAAGGGCAGGCCCTGAAATCTAGTGGCGAGCT-CGCCAGGAC-CCCGAGCGTAGTAG-TTACA-TCTCGCTCTGG-AAGGCCCTGGCGGTGCCCTGCCGTTAAA-CCCCCAACTTCTGAAAATT

>Diaporthe_cuppatea_CBS_117499

NNNNNNNNNNNNNNNNNNNNNNNNNNNNNGGATCATTGCTGGAACGCG-CTTT-GGCGCA-CCCAGAAACCCTTTGTGAACTTATACCC--ACTGTTGCCTCGGCG-CAGGCCGGCCTTT--GT--------TGCAAAAGGCC-----CCCTG---GAAACAGGGAGCA-GCCCGCCGGCGGCCAA-CCAAACTC-TGTTTCTATAGTGAATCTCTGAGT--AAAAAACATAAATGAATCAAAACTTTCAACAACGGATCTCTTGGTTCTGGCATCGATGAAGAACGCAGCGAAATGCGATAAGTAATGTGAATTGCAGAATTCAGTGAATCATCGAATCTTTGAACGCACATTGCGCCCTCTGGTATTCCGGAGGGCATGCCTGTTCGAGCGTCATTTCAACCCTCAAGCCCGGCTTGG-TGATGGGGCACTGCCTGT--------AAAAGGGCAGGCCCTGAAATCTAGTGGCGAGCT-CGCCAGGAC-CCCGAGCGTAGTAG-TCATA-TCTCGCTCTGG-AAGGCCCTGGCGGTGCCCTGCCGTTAAA-CCCCCAACTTCTGAAAATT

>Diaporthe_discoidispora_ICMP20662

NNNNNNNNNNNNNNNNNNNNNNNNNNNNNNNNNNNNNNCTGGAACGCG-CCTC-GGCGCA-CCCAGAAACCCTTTGTGAACTTATACCT-TACTGTTGCCTCGGCG-CAGGCCGGCCTCT--TA----------GCTGAGGCC-----CCCCG---GAGACGGGGAGCA-GCCCGCCGGCGGCCAA-GTTAACTCTTGTTTCTACCATGAATCTCTGAGT-AAAAAAACATAAATGAATCAAAACTTTCAACAACGGATCTCTTGGTTCTGGCATCGATGAAGAACGCAGCGAAATGCGATAAGTAATGTGAATTGCAGAATTCAGTGAATCATCGAATCTTTGAACGCACATTGCGCCCTCTGGTATTCCGGAGGGCATGCCTGTTCGAGCGTCATTTCAACCCTCAAGCCTGGCTTGG-TGTTGGGGCACTGCTTCG--------AGAGAAGCAGGCCCTGAAATCTAGTGGCGAGCT-CGCTAGGAC-CCCGAGCGTAGTAG-TTATA-TCTCGTTCTGG-AAGGCCCTGGCGGTGCCCTGCCGTTAAA-CCCCCAACTTCTGAAAATT

>Diaporthe_durionigena_VTCC_930005

?????????????????????????????????????????????????????????????????????CCCTTTGTGAACTTATACCTAT-CTGTTGCCTCGGCG-TAGGCCGGCCTCT--TC----------ACTGAGGCC-----CCCTG---GAAACAGGGAGCA-GCCCGCCGGCGGCCAA-CTAAACTCTTGTTTCTATAGTGAATCTCTGAGT---AAAAACATAAATGAATCAAAACTTTCAACAACGGATCTCTTGGTTCTGGCATCGATGAAGAACGCAGCGAAATGCGATAAGTAATGTGAATTGCAGAATTCAGTGAATCATCGAATCTTTGAACGCACATTGCGCCCTCTGGTATTCCGGAGGGCATGCCTGTTCGAGCGTCATTTCAACCCTCAAGCCTGGCTTGG-TGATGGGGCACTGCCTTCT------AGCGAGGGCAGGCCCTGAAATCTAGTGGCGAGCT-CGCTAGGAC-CCCGAGCGTAGTAG-TTATA-TCTCGTTCTGG-AAGGCCCTGGCGGTGCCCTGCCGTTAAA-CCCCCAACTTCTGAAAATT

>Diaporthe_eres_CBS_138594

???????????????????????GCGGAGGGATCATTGCTGGAACGCG-CCCCAGGCGCA-CCCAGAAACCCTTTGTGAACTTATACCT-TACTGTTGCCTCGGCG-CTAGCTGGTCCCT----------------CGGGGCCCCTCACCCTC---GGGTGTTGAGACA-GCCCGTCGGCGGCCAA-CCTAACTCTTGTTTTTACACTGAAACTCTGAGC--ACAAAACATAAATGAATCAAAACTTTCAACAACGGATCTCTTGGTTCTGGCATCGATGAAGAACGCAGCGAAATGCGATAAGTAATGTGAATTGCAGAATTCAGTGAATCATCGAATCTTTGAACGCACATTGCGCCCTCTGGTATTCCGGAGGGCATGCCTGTTCGAGCGTCATTTCAACCCTCAAGCCTGGCTTGG-TGATGGGGCACTGCTTCTTAC----CCAAGAAGCAGGCCCTGAAATTCAGTGGCGAGCT-CGCCAGGAC-CCCGAGCGCAGTAG-TTAAACCCTCGCTCTGG-AAGGCCCTGGCGGTGCCCTGCCGTTAAA-CCCCCAACTTCTGAAAATT

>Diaporthe_fici_septicae_MFLU_18_2588

NNNNNNNNTCCGTTGGTGAACCAGCGGAGGGA-CATTGCTGGAACGCG-CCCC-GGCGCA-CCCAGAAACCCTTTGTGAACTTATACCT-TACTGTTGCCTCGGCG-CAGGCCGGCCTCC--CA----------GCTGAGGCC-----CCCCG---GAGACGGGGAGCA-GCCCGCCGGCGGCCAA-CTAAACTCTTGTTTCTACAGTGGATCTCTGAGTTAAAAAAACATAAATGAATCAAAACTTTCAACAACGGATCTCTTGGTTCTGGCATCGATGAAGAACGCAGCGAAATGCGATAAGTAATGTGAATTGCAGAATTCAGTGAATCATCGAATCTTTGAACGCACATTGCGCCCTCTGGTATTCCGGAGGGCATGCCTGTTCGAGCGTCATTTCAACCCTCAAGCCTGGCTTGG-TGTTGGGGCACTGCCTGT--------AAAAGGGCAGGCCCTGAAATCTAGTGGCGAGCT-CGCTAGGAC-CCCGAGCGTAGTAG-TTATA-TCTCGTTCTGG-AAGGCCCTGGCGGCGCCCTGCCGTTAAA-CCCCCNNNNNNNNNNNNNN

>Diaporthe_fructicola_MAFF_246408

ACAAGGTCTCCGTTGGTGAACCAGCGGAGGGATCATTGCTGGAACGCG-CTTC-GGCGCA-CCCAGAAACCCTTTGTGAACTTATACCTAT--TGTTGCCTCGGCG-TAGGCCGGCCTCT--TC----------ACTGAGGCC-----CCCTG---GAAACAGGGAGCA-GCCCGCCGGCGGCCAA-CCAAACTCTTGTTTCTACAGTGAATCTCTGAGT--AAAAAACATAAATGAATCAAAACTTTCAACAACGGATCTCTTGGTTCTGGCATCGATGAAGAACGCAGCGAAATGCGATAAGTAATGTGAATTGCAGAATTCAGTGAATCATCGAATCTTTGAACGCACATTGCGCCCTCTGGTATTCCGGAGGGCATGCCTGTTCGAGCGTCATTTCAACCCTCAAGCCTGGCTTGG-TGATGGGGCACTGCCTGTA--------AAAGGGCAGGCCCTGAAATCTAGTGGCGAGCT-CGCCAGGAC-CCCGAGCGTAGTAG-TTATA-TCTCGCTCTGG-AAGGCCCTGGCGGTGCCCTGCCGTTAAA-CCCCCAACTTCTGAAAATT

>Diaporthe_ganjae_CBS_180_91

ACAAGGTCTCCGTTGGTGAACCAGCGGAGGGATCATTGCTGGAACGCG-CCCC-GGCGCA-CCCAGAAACCCTTTGTGAACCTATACCC--ACTGTTGCCTCGGCG-CAGGCCGGTCTGT---------------CTCAGACC-----CCCTG---GAGACAGGGAGCA-GCCCGCCGGCGGCCGA-CCAAACTC-CGTTTCTATAGTGAATCTCTGAGT--TAAAAACATAAATGAATCAAAACTTTCAACAACGGATCTCTTGGTTCTGGCATCGATGAAGAACGCAGCGAAATGCGATAAGTAATGTGAATTGCAGAATTCAGTGAATCATCGAATCTTTGAACGCACATTGCGCCCTCTGGTATTCCGGAGGGCATGCCTGTTCGAGCGTCATTTCAACCCTCAAGCCTGGCTTGG-TGATGGGGCAGTGCCTTGGA------GACAAGGCACGCCCTGAAATTCAGTGGCGAGCT-CGCCAGGAC-CCCGAGCGTAGTAG-TTACA-TCTCGCTCCGG-AAGGCCCTGGCGGTGCCCTGCCGTTAAA-CCCCCAACTTCTGAAAATT

>Diaporthe_goulteri_BRIP_55657a

ACAAGGTCTCCGTTGGTGAACCAGCGGAGGGATCATTGCTGGAACGCG-CCCCTGGCGCA-CCCAGAAACCCTTTGTGAACTTATACCT--ACCGTTGCCTCGGCG-CAGGCCGGCCCCC--CT--------CACCGGGGGCC-----CCCCG---GAGACGGGGAGCA-GCCCGCCGGCGGCCAA-CCAAACTCTTGTTTCT-TAGTGAATCTCTGAGT--AAAAATCATAAATGAATCAAAACTTTCAACAACGGATCTCTTGGTTCTGGCATCGATGAAGAACGCAGCGAAATGCGATAAGTAATGTGAATTGCAGAATTCAGTGAATCATCGAATCTTTGAACGCACATTGCGCCCTCTGGTATTCCGGAGGGCATGCCTGTTCGAGCGTCATTTCAACCCTCAAGCCTGGCTTGG-TGATGGGGCACTGCCTTCGTA----ACAGAGGGCAGGCCCTGAAATCTAGTGGCGAGCT-CGCTAGGAC-CCCGAGCGTAGTAGTTTATA-TCTCGTTCTGG-AAGGCCCTGGCGGTGCCCTGCCGTTAAA-CCCCCAACTTCTGAAAATT

>Diaporthe_guangdongensis_ZHKUCC20_0014

????????TCCGTAGGTGAACCTGCGGAGGGATCATTGCTGGAACGCG-CCTC-GGCGCA-CCCAGAAACCCTTTGTGAACTTATACCTA--CTGTTGCCTCGGCG-CAGGCCGGCCTTTGTCA----------AAGAAGGCC-----CCCTG---GGAACAGGGAGCA-GCCCGCCGGCGGCCAA-CTAAACTCTTGTTTCTATAGTGAATCTCTGAGT-AAAAAAACATAAATGAATCAAAACTTTCAACAACGGATCTCTTGGTTCTGGCATCGATGAAGAACGCAGCGAAATGCGATAAGTAATGTGAATTGCAGAATTCAGTGAATCATCGAATCTTTGAACGCACATTGCGCCCTCTGGTATTCCGGAGGGCATGCCTGTTCGAGCGTCATTTCAACCCTCAAGCCTGGCTTGG-TGATGGGGCACTGCTCTCTC------GCGGGAGCAGGCCCTGAAATCTAGTGGCGAGCT-CGCCAGGAC-CCCGAGCGTAGTAG-TTACA-TCTCGCTCTGG-AAGGCCCTGGCGGTGCCCTGCCGTTAAA-CCCCCAACTCCTGAAAATT

>Diaporthe_gulyae_BRIP_54025

NNNNNNNNNNNNNNNNNNNNNNNNNNNAGGGATCATTGCTGGAACGCG-CCTC-GGCGCA-CCCAGAAACCCTTTGTGAACTTATACCCATACTGTTGCCTCGGCG-CAGGCCGGCCTTT--TT--------CGACAAAGGCC-----CCCTG---GAGACAGGGAGCA-GCCCGCCGGCGGCCAA-CCAAACTC-TGTTTCTATAGTGAATCTCTGAGT--AAAAAACATAAATGAATCAAAACTTTCAACAACGGATCTCTTGGTTCTGGCATCGATGAAGAACGCAGCGAAATGCGATAAGTAATGTGAATTGCAGAATTCAGTGAATCATCGAATCTTTGAACGCACATTGCGCCCTCTGGTATTCCGGAGGGCATGCCTGTTCGAGCGTCATTTCAACCCTCAAGCCTGGCTTGG-TGATGGGGCACTGCCTGT--------GAAAGGGCAGGCCCTGAAATCTAGTGGCGAGCT-CGCCAGGAC-CCCGAGCGTAGTAG-TTACA-TCTCGCTCTGG-AAGGCCCTGGCGGTGCCCTGCCGTTAAA-CCCCCAACTTCTGAAAATT

>Diaporthe_guttulata_CGMCC_3_20100

NNNNNNNNNNNNNNNNNNNNNNNNNNNNNNNNNNNNNNNNNNNNNNNNNNNTC-GGCGCA-CCCAGAAACCCTTTGTGAACTTATACCCATACTGTTGCCTCGGCG-CAGGCCGGCCCTT--TT--------CGATAAGGGCC-----CCCTG---GAGACAGGGAGCA-GCCCGCCGGCGGCCAA-CCAAACTC-TGTTTCTATAGTGAATCTCTGAGT--AAAAAACATAAATGAATCAAAACTTTCAACAACGGATCTCTTGGTTCTGGCATCGATGAAGAACGCAGCGAAATGCGATAAGTAATGTGAATTGCAGAATTCAGTGAATCATCGAATCTTTGAACGCACATTGCGCCCTCTGGTATTCCGGAGGGCATGCCTGTTCGAGCGTCATTTCAACCCTCAAGCCTGGCTTGG-TGATGGGGCACTGCCTGT--------GAAAGGGCAGGCCCTGAAATCTAGTGGCGAGCT-CGCCAGGAC-CCCGAGCGTAGTAG-TTACA-TCTCGCTCTGG-AAGGCCCTGGCGGTGCCCTGCCGTTAAA-CCCCCAACTNNNNNNNNNN

>Diaporthe_helianthi_CBS_592_81

ACAAGGTCTCCGTTGGTGAACCAGCGGAGGGATCATTGCTGGAACGCGCCCCC-GGCGCA-CCCAGAAACCCTTTGTGAACTTATACCTAT-CTGTTGCCTCGGCG-CAGGCCGGCCCCC---C----------CTGGGGGCC-----CCCTG---GGAACAGGGAGCA-GCCCGCCGGCGGCCGA-CCAAACTCTTGTTTCTACAGTGGATCTCTGAGT--TAAAAACACAAATGAATCAAAACTTTCAACAACGGATCTCTTGGTTCTGGCATCGATGAAGAACGCAGCGAAATGCGATAAGTAATGTGAATTGCAGAATTCAGTGAATCATCGAATCTTTGAACGCACATTGCGCCCTCTGGTATTCCGGAGGGCATGCCTGTTCGAGCGTCATTTCAACCCTCAAGCCTGGCTTGG-TGATGGGGCACTGCCTGTG--------ACAGGGCAGGCCCTGAAATCCAGCGGCGAGCC-CGCCGGGAC-CCCGAGCGTAGTAG-TAACT-TCTCGCTCCGG-AAGGCCCTGGCGGCGCCCTGCCGTTAAA-CCCCCAACTCCTGAAAATT

>Diaporthe_heterostemmatis_SAUCC194_85

??????TCTCCGTTGGTGAACCAGCGGAGGGATCATTGCTGGAACGCG-CTTC-GGCGCA-CCCAGAAACCCTTTGTGAACTTATACCTAT--TGTTGCCTCGGCG-CAGGCCGGCCTCT--TC----------ACTGAGGCC-----CCCTG---GAAACAGGGAGCA-GCCCGCCGGCGGCCAA-CCAAACTCTTGTTTCTATAGTGAATCTCTGAGT-AAAAAAACATAAATGAATCAAAACTTTCAACAACGGATCTCTTGGTTCTGGCATCGATGAAGAACGCAGCGAAATGCGATAAGTAATGTGAATTGCAGAATTCAGTGAATCATCGAATCTTTGAACGCACATTGCGCCCTCTGGTATTCCGGAGGGCATGCCTGTTCGAGCGTCATTTCAACCCTCAAGCCTGGCTTGG-TGATGGGGCACTGCCTGTA--------ATAGGGCAGGCCCTGAAATCTAGTGGCGAGCT-CGCCAGGAC-CCCGAGCGTAGTAG-TTATA-TCTCGCTCTGG-AAGGCCCTGGCGGTGCCCTGCCGTTAAA-CCCCCAACTTCTGAAAATT

>Diaporthe_hordei_CBS_481_92

ACAAGGTCTCCGTTGGTGAACCAGCGGAGGGATCATTGCTGGAACGCG-CCTC-GGCGCA-CCCAGAAACCCTTTGTGAACTTATACCTAT-CTGTTGCCTCGGCG-CAGGCCGGCCCCC--TC----------ACCGGGGCC-----CCCTG---GAGACAGGGAGCA-GCCCGCCGGCGGCCAA-CCAAACTCTTGTTTCTACAGTGAATCTCTGAGT-AAAAAAACATAAATGAATCAAAACTTTCAACAACGGATCTCTTGGTTCTGGCATCGATGAAGAACGCAGCGAAATGCGATAAGTAATGTGAATTGCAGAATTCAGTGAATCATCGAATCTTTGAACGCACATTGCGCCCTCTGGTATTCCGGAGGGCATGCCTGTTCGAGCGTCATTTCAACCCTCAAGCCTGGCTTGG-TGATGGGGCACTGCCTGTG--------AAAGGGCAGGCCCTGAAATCTAGTGGCGAGCT-CGCCAGGAC-CCCGAGCGTAGTAG-TTATA-TCTCGCTCTGG-AAGGCCCTGGCGGTGCCCTGCCGTTAAA-CCCCCAACTTCTGAAAATT

>Diaporthe_hubeiensis_JZB320123

NNNNNNNNNNNNNNNNNNNNNNNNNNNNNNNNNNNNNNNNNNNACGCG-CTTC-GGCGCA-CCCAGAAACCCTTTGTG-ACTTATACCTATACTGTTGCCTCGGCG-CTGGCCGGCCTCC--TC----------ACCGAGGCC-----CCCTG---GAGACAGGGAGCA-GCCCGCCGGCGGCCAA-ACAAACTCTTGTTTCT-TAGTGAATCTCTGAGT--AAAAAACAT-AATGAATCAAAACTTTCAACAACGGATCTCTTGGTTCTGGCATCGATGAAGAACGCAGCGAAATGCGATAAGTAATGTGAATTGCAGAATTCAGTGAATCATCGAATCTTTGAACGCACATTGCGCCCTCTGGTATTCCGGAGGGCATGCCTGTTCGAGCGTCATTTCAACCCTCAAGCCTGGCTTGG-TGTTGGGGCACCGCCTTTGC------AAAAGGGCGGGCCCTGAAATCTAGTGGCGAGCT-CGCCAGGAC-CCCGAGCGTAGTAG-TTATA-TCTCGTTCTGG-AAGGCCCTGGCGGTGCCCTGCCGTTAAA-CCCCCAACTTCTGAAATTT

>Diaporthe_infecunda_CBS_133812

ACAAGGTCTCCGTTGGTGAACCAGCGGAGGGATCATTGCTGGAACGCG-CTTC-GGCGCA-CCCAGAAACCCTTTGTGAACTTATACCT--ATTGTTGCCTCGGCGTTAGGCCGGCCTCT--TC----------ACCGAGGCC-----CCCTG---GAAACAGGGAGCA-GCCCGCCGGCGGCCAA-CTAAACTCTTGTTTCTATAGTGAATCTCTGAGT--AAAAAACATAAATGAATCAAAACTTTCAACAACGGATCTCTTGGTTCTGGCATCGATGAAGAACGCAGCGAAATGCGATAAGTAATGTGAATTGCAGAATTCAGTGAATCATCGAATCTTTGAACGCACATTGCGCCCCCTGGTATTCCGGGGGGCATGCCTGTTCGAGCGTCATTTCAACCCTCAAGCCTGGCTTGG-TGATGGGGCACTGCTTCG--------AAAGGAGCAGGCCCTGAAATTCAGTGGCGAGCT-CGCCAGGAC-CCCGAGCGTAGTAG-TTATA-TCTCGCTTTGG-AAGGCCCTGGCGGTGCCCTGCCGTTAAA-CCCCCAACTTCTGAAAATT

>Diaporthe_infertilis_CBS_230_52

ACAAGGTCTCCGTTGGTGAACCAGCGGAGGGATCATTGCTGGAACGCG-CTTC-GGCGCA-CCCAGAAACCCTTTGTGAACTTATACCTA--CTGTTGCCTCGGCG-CAGGCCGGCCTTTTGTG----------ACAAAGGCC-----CCCTG---GAGACAGGGAGCA-GCCCGCCGGCGGCCAA-CTAAACTCTTGTTTCTATAGTGAATCTCTGAGT---AAAAACATAAATGAATCAAAACTTTCAACAACGGATCTCTTGGTTCTGGCATCGATGAAGAACGCAGCGAAATGCGATAAGTAATGTGAATTGCAGAATTCAGTGAATCATCGAATCTTTGAACGCACATTGCGCCCTCTGGTATTCCGGAGGGCATGCCTGTTCGAGCGTCATTTCAACCCTCAAGCCTGGCTTGG-TGATGGGGCACTGCCTGTT--------ACAGGGCAGGCCCTGAAATCTAGTGGCGAGCT-CGCCAGGAC-CCCGAGCGTAGTAG-TTATA-TCTCGCTTTGG-AAGGCCCTGGCGGTGCCCTGCCGTTAAA-CCCCCAACTTCTGAAAATT

>Diaporthe_kochmanii_BRIP_54033

???????????????????????????AGGGATCATTGCTGGAACGCG-CTTC-GGCGCA-CCCAGAAACCCTTTGTGAACTTATACCTAT--TGTTGCCTCGGCG-TAGGCCGGCCTCT--TC----------ACTGAGGCC-----CCCTG---GAAACAGGGAGCA-GCCCGCCGGCGGCCAA-CCAAACTCTTGTTTCTACAGTGAATCTCTGAGT--AAAAAACATAAATGAATCAAAACTTTCAACAACGGATCTCTTGGTTCTGGCATCGATGAAGAACGCAGCGAAATGCGATAAGTAATGTGAATTGCAGAATTCAGTGAATCATCGAATCTTTGAACGCACATTGCGCCCTCTGGTATTCCGGAGGGCATGCCTGTTCGAGCGTCATTTCAACCCTCAAGCCTGGCTTGG-TGATGGGGCACTGCTTTCGTC-----CAGAAAGCAGGCCCTGAAATCTAGTGGCGAGCT-CGCCAGGAC-CCCGAGCGTAGTAG-TTATA-TCTCGCTCTGG-AAGGCCCTGGCGGTGCCCTGCCGTTAAA-CCCCCAACTTCTGAAAATT

>Diaporthe_kongii_BRIP_54031

???????????????????????????AGGGATCATTGCTGGAACGCG-CTTC-GGCGCA-CCCAGAAACCCTTTGTGAACTTATACCTAT--TGTTGCCTCGGCG-TAGGCCGGCTTTT--TC-----------TAAAAGCC-----CCCTG---GAAACAGGGAGAA-GCCCGCCGGCGGCCAA-CCAAACTCTTGTTTCTACAGTGAATCTCTGAGT--AAAAAACATAAATGAATCAAAACTTTCAACAACGGATCTCTTGGTTCTGGCATCGATGAAGAACGCAGCGAAATGCGATAAGTAATGTGAATTGCAGAATTCAGTGAATCATCGAATCTTTGAACGCACATTGCGCCCTCTGGTATTCCGGAGGGCATGCCTGTTCGAGCGTCATTTCAACCCTCAAGCCTGGCTTGG-TGATGGGGCACTGCCTGTA--------AAAGGGCAGGCCCTGAAATCTAGTGGCGAGCT-CGCCAGGAC-CCCGAGCGTAGTAG-TTATA-TCTCGCTCTGG-AAGGCCCTGGCGGTGCCCTGCCGTTAAA-CCCCCAACTTCTGAAAATT

>Diaporthe_leucospermi_CBS_111980

ACAAGGTCTCCGTTGGTGAACCAGCGGAGGGATCATTGCTGGAACGCG-CTTC-GGCGCA-CCCAGAAACCCTTTGTGAACTTATACCT--ACTGTTGCCTCGGCG-TAAGCTGGCTTTG--AA----------ATATAAGCC-----CCCTG---GAAACGGGGAGCA-GCCCGCCGGCGGCCAA-CTAAACTCTTGTTTCTATAGTGAATCTCTGAGT--AAAAAACATAAATGAATCAAAACTTTCAACAACGGATCTCTTGGTTCTGGCATCGATGAAGAACGCAGCGAAATGCGATAAGTAATGTGAATTGCAGAATTCAGTGAATCATCGAATCTTTGAACGCACATTGCGCCCCCTGGTATTCCGGGGGGCATGCCTGTTCGAGCGTCATTTCAACCCTCAAGCCTGGCTTGG-TGATGGGGCACTGCTTCG--------AAAGGAGCAGGCCCTGAAATTCAGTGGCGAGCT-CGCCAGGAC-CCCGAGCGTAGTAG-TTATA-TCTCGCTTTGG-AAGGCCCTGGCGGTGCCCTGCCGTTAAA-CCCCCAACTTCTGAAAATT

>Diaporthe_longicolla_FAU_599

???????????????????????GCGGAGGGATCATTGCTGGAACGCG-CTTC-GGCGCA-CCCAGAAACCCTTTGTGAACTTATACCTA--CTGTTGCCTCGGCG-CAGGCCGGCCTTTTGTG----------ACAAAGGCC-----CCCTG---GAGACAGGGAGCA-GCCCGCCGGCGGCCAA-CCAAACTCTTGTTTCTACAGTGAATCTCTGAGT--ACAAAACATAAATGAATCAAAACTTTCAACAACGGATCTCTTGGTTCTGGCATCGATGAAGAACGCAGCGAAATGCGATAAGTAATGTGAATTGCAGAATTCAGTGAATCATCGAATCTTTGAACGCACATTGCGCCCTCTGGTATTCCGGAGGGCATGCCTGTTCGAGCGTCATTTCAACCCTCAAGCCTGGCTTGG-TGATGGGGCACTGCTCTCT------GACGGGAGCAGGCCCTGAAATCTAGTGGCGAGCT-CGCTAGGAC-CCCGAGCGTAGTAG-TTATA-TCTCGTTCTGG-AAGGCCCTGGCGGTGCCCTGCCGTTAAA-CCCCCAACTTCTGAAAATT

>Diaporthe_longispora_CBS_194_36

ACAAGGTCTCCGTTGGTGAACCAGCGGAGGGATCATTGCTGGAACGCG-CTTC-GGCGCA-CCCAGAAACCCTTTGTGAACTCATACCT-TACTGTTGCCTCGGCG-CAGGCCGGCCCCC---------------CTGGGGCC-----CCTCG---TTCCCGAGGAGCA-GCCCGCCGGCGGCCAA-CCAAACTCTTGTTTCT-TAGTGAGTCTCTGAGT--AAAAAACAAAAATAAATCAAAACTTTCAACAACGGATCTCTTGGTTCTGGCATCGATGAAGAACGCAGCGAAATGCGATAAGTAATGTGAATTGCAGAATTCAGTGAATCATCGAATCTTTGAACGCACATTGCGCCCTCTGGTATTCCGGAGGGCATGCCTGTTCGAGCGTCATTTCAACCCTCAAGCTCTGCTTGG-TGATGGGGCACCGCCCGTA--------AGAGGGCGGGCCCTGAAATCTAGTGGCGAGCT-CGCCAGGAC-CCCGAGCGTAGTAG-TTATA-TCTCGCCCTGG-AAGGCCCTGGCGGTGCCCTGCCGTTAAA-CCCCCAACTTCTGAAATTT

>Diaporthe_lusitanicae_CBS_123212

ACAAGGTCTCCGTTGGTGAACCAGCGGAGGGATCATTGCTGGAACGCG-CTTC-GGCGCA-CCCAGAAACCCTTTGTGAACTTATACCC--ACTGTTGCCTCGGCG-CAGGCCGGCCTCT--TC----------ACTGAGGCC-----CCCCC---GAAAGGGGGAGCA-GCCCGCCGGCGGCCAA-CTAAACTC-TGTTTCTATAGTGAATCTCTGAGT-AAAAAAACATAAATGAATCAAAACTTTCAACAACGGATCTCTTGGTTCTGGCATCGATGAAGAACGCAGCGAAATGCGATAAGTAATGTGAATTGCAGAATTCAGTGAATCATCGAATCTTTGAACGCACATTGCGCCCTCTGGTATTCCGGAGGGCATGCCTGTTCGAGCGTCATTTCAACCCTCAAGCCTGGCTTGG-TGATGGGGCACTGCCTGT--------AAAAGGGCAGGCCCTGAAATCTAGTGGCGAGCT-CGCCAGGAC-CCCGAGCGTAGTAG-TTACA-TCTCGCTCTGG-AAGGCCCTGGCGGTGCCCTGCCGTTAAA-CCCCCAACTTCTGAAAATT

>Diaporthe_machili_SAUCC194_111

NNNNNNTCTCCGTTGGTGAACCAGCGGAGGGATCATTGCTGGAACGCG-CTTC-GGCGCA-CCCAGAAACCCTTTGTGAACTTATACCT--ACTGTTGCCTCGGCGTCAGGCCGGCCTCC--TC----------ACCGAGGCC-----CCCCG---GAGACGGGGAGCA-GCCCGCCGGCGGCCAA-CTAAACTCTTGTTTCTATAGTGAATCTCTGAGT--AAAAAACATAAATGAATCAAAACTTTCAACAACGGATCTCTTGGTTCTGGCATCGATGAAGAACGCAGCGAAATGCGATAAGTAATGTGAATTGCAGAATTCAGTGAATCATCGAATCTTTGAACGCACATTGCGCCCCCTGGTATTCCGGGGGGCATGCCTGTTCGAGCGTCATTTCAACCCTCAAGCCTGGCTTGG-TGATGGGGCACTGCTTCG--------AGAGGAGCAGGCCCTGAAATTCAGTGGCGAGCT-CGCCAGGAC-CCCGAGCGTAGTAG-TTATA-TCTCGCTCTGG-AAGGCCCTGGCGGTGCCCTGCCGTTAAA-CCCCCAACTTCTGAAAATT

>Diaporthe_manihotia_CBS_505_76

NNNNNNNNNNNNNNNNNNNNNNNGCGGAGGGATCATTGCTGGAACGCG-CTTC-GGCGCA-CCCAGAAACCCTTTGTGAACTTATACCT--ACTGTTGCCTCGGCG-CAGGCCGGTCTGT---------------CTCAGACC-----CCCTG---GAAACAGGGAGCA-GCCCGCCGGCGGCCAA-CTAAACTC-TGTTTCTATAGTGAATCTCTGAGT--AAAAAACATAAATGAATCAAAACTTTCAACAACGGATCTCTTGGTTCTGGCATCGATGAAGAACGCAGCGAAATGCGATAAGTAATGTGAATTGCAGAATTCAGTGAATCATCGAATCTTTGAACGCACATTGCGCCCTCTGGTATTCCGGAGGGCATGCCTGTTCGAGCGTCATTTCAACCCTCAAGCCTGGCTTGG-TGATGGGGCAGTGCTCTGGA------GACAGAGCACGCCCTGAAATTCAGTGGCGAGCT-CGCCAGGAC-CCCGAGCGTAGTAG-TTATA-TCTCGCTCTGG-AAGGCCCTGGCGGTGCCCTGCCGTTAAA-CCCCCAACTTCTGAAAATT

>Diaporthe_mayteni_CBS_133185

ACAAGGTCTCCGTTGGTGAACCAGCGGAGGGATCATTGCTGGAACGCG-CCCC-GGCGCA-CCCAGAAACCCTTTGTGAACCTATACCT-TACTGTTGCCTCGGCG-CAGGCCGGCCTCC--------------CGTGAGGCC-----CCTCG---GAGACGAGGAGCA-GCCCGCCGGCGGCCAA-GCAAACTCTTGTTTCT-TAGTGGATCTCTGAGT--AAAAAACACAAATGAATCAAAACTTTCAACAACGGATCTCTTGGTTCTGGCATCGATGAAGAACGCAGCGAAATGCGATAAGTAATGTGAATTGCAGAATTCAGTGAATCATCGAATCTTTGAACGCACATTGCGCCCCCTGGTATTCCGGGGGGCATGCCTGTTCGAGCGTCATTTCACCCCTCAAGCCTGGCTTGG-TGCTGGGGCACTACTCCCTC------GCCGGAGTAGGCCCTGAAATCCAGCGGCGAGCC-CTATGGGTA-ACCGAGTGCAGTAG-T--AT-TCTCGCTCTGG-GATGCCCTGGCGGCGC---GCCGTTAAA-CCCCCAACTCCTGAGATTT

>Diaporthe_megalospora_CBS_143_27

ACAAGGTCTCCGTTGGTGAACCAGCGGAGGGATCATTGCTGGAACGCG-CTTC-GGCGCA-CCCAGAAACCCTTTGTGAACTTATACCTA--CTGTTGCCTCGGCG-CAGGCCGGCCTCC--CT----------TCCGAGGCC-----CCCTG---GGAACAGGGAGCA-GCCCGCCGGCGGCCGA-CCAGACTCTTGTTTCTGTAGTGGATCTCTGAGT-ACAAAAACACAAATGAATCAAAACTTTCAACAACGGATCTCTTGGTTCTGGCATCGATGAAGAACGCAGCGAAATGCGATAAGTAATGTGAATTGCAGAATTCAGTGAATCATCGAATCTTTGAACGCACATTGCGCCCTCTGGTATTCCGGAGGGCATGCCTGTTCGAGCGTCATTTCAACCCTCAAGCCTGGCTTGG-TGATGGGGCACTGCCCGTA--------ACAGGGCAGGCCCTGAAATCCAGTGGCGAGCT-CGCCAGGAC-CCCGAGCGTAGTAG-TTACA-TCTCGCTCCGG-AAGGCCCTGGCGGTGCCCTGCCGTTAAA-CCCCCAACTCCTGAAAATT

>Diaporthe_melonis_CBS_507_78

ACAAGGTCTCCGTTGGTGAACCAGCGGAGGGATCATTGCTGGAACGCG-CCTC-GGCGCA-CCCAGAAACCCTTTGTGAACTTATACCTA--CTGTTGCCTCGGCG-CAGGCCGGCCTTTGTCA----------AAGAAGGCC-----CCCTG---GAGACAGGGAGCA-GCCCGCCGGCGGCCAA-CTAAACTCTTGTTTCTATAGTGAATCTCTGAGT---AAAAACATAAATGAATCAAAACTTTCAACAACGGATCTCTTGGTTCTGGCATCGATGAAGAACGCAGCGAAATGCGATAAGTAATGTGAATTGCAGAATTCAGTGAATCATCGAATCTTTGAACGCACATTGCGCCCTCTGGTATTCCGGAGGGCATGCCTGTTCGAGCGTCATTTCAACCCTCAAGCCTGGCTTGG-TGATGGGGCACTGCTCTCTC------GCGGGAGCAGGCCCTGAAATCTAGTGGCGAGCT-CGCCAGGAC-CCCGAGCGTAGTAG-TTACA-TCTCGCTCTGG-AAGGCCCTGGCGGTGCCCTGCCGTTAAA-CCCCCAACTTCTGAAAATT

>Diaporthe_middletonii_BRIP_54884e

ACAAGGTCTCCGTTGGTGAACCAGCGGAGGGATCATTGCTGGAACGCG-CTTC-GGCGCA-CCCAGAAACCCTTTGTGAACTTATACCT--ATTGTTGCCTCGGCGTTAGGCCGGCCTCC--TC----------ACTGAGGCC-----CCCTG---GAAACAGGGAGCA-GCCCGCCGGCGGCCAA-CTAAACTCTTGTTTCTATAGTGAATCTCTGAGT--AAAAAACATAAATGAATCAAAACTTTCAACAACGGATCTCTTGGTTCTGGCATCGATGAAGAACGCAGCGAAATGCGATAAGTAATGTGAATTGCAGAATTCAGTGAATCATCGAATCTTTGAACGCACATTGCGCCCCCTGGTATTCCGGGGGGCATGCCTGTTCGAGCGTCATTTCAACCCTCAAGCCTGGCTTGG-TGATGGGGCACTACTTCCTC------ACGGGAGTAGGCCCTGAAATTCAGTGGCGAGCT-CGCCAGGAC-CCCGAGCGTAGTAG-TTATA-TCTCGCTTTGG-AAGGCCCTGGCGGTGCCCTGCCGTTAAA-ACCCCAACTTCTGAAAATT

>Diaporthe_miriciae_BRIP_54736j

ACAAGGTCTCCGTTGGTGAACCAGCGGAGGGATCATTGCTGGAACGCG-CTTC-GGCGCA-CCCAGAAACCCTTTGTGAACTTATACCTAT-TTGTTGCCTCGGCG-TAGGCCGGCCTCT--TC----------ACTGAGGCC-----CCCTG---GAGACAGGGAGCA-GCCCGCCGGCGGCCAA-CTAAACTCTTGTTTCTATAGTGAATCTCTGAGT--AAAAAACATAAATGAATCAAAACTTTCAACAACGGATCTCTTGGTTCTGGCATCGATGAAGAACGCAGCGAAATGCGATAAGTAATGTGAATTGCAGAATTCAGTGAATCATCGAATCTTTGAACGCACATTGCGCCCTCTGGTATTCCGGAGGGCATGCCTGTTCGAGCGTCATTTCAACCCTCAAGCCTGGCTTGG-TGATGGGGCACTGCCTTCT------AGCGAGGGCAGGCCCTGAAATCTAGTGGCGAGCT-CGCTAGGAC-CCCGAGCGTAGTAG-TTATA-TCTCGTTCTGG-AAGGCCCTGGCGGTGCACTGCCGTTAAA-CCCCCAACTTCTGAAAATT

>Diaporthe_myracrodruonis_URM7972

NNNNNNNNNNNNNNNNNNNACCTGCGGAGGGATCATTGCTGGAACGCG-CCCC-GGCGCA-CCCAGAAACCCTTTGTGAACTTATACGTA--CTGTTGCCTCGGCG-CAGGCCGGCTCTG--AG----------ATACGAGCC-----CCCCG---GAGACGGGGAGAA-GCCCGCCGGCGGCCAA-GCAAACTCTTGTTTCTACAGTGGATCTCTGAGT--AAAAAACATAAATGAATCAAAACTTTCAACAACGGATCTCTTGGTTCTGGCATCGATGAAGAACGCAGCGAAATGCGATAAGTAATGTGAATTGCAGAATTCAGTGAATCATCGAATCTTTGAACGCACATTGCGCCCCCTGGTATTCCGGGGGGCATGCCTGTTCGAGCGTCATTTCACCCCTCAAGCCTGGCTTGG-TGATGGGGCACTGCCCGTA--------ACAAGGCAGGCCCTGAAATCCAGCGGCGCACCACTGTGGAAA-ACCGAGCGTAGTAG-TTATG-TCTCGCCCGGT-ACGCCCGCAGCGGT--CCTGCCGTTAAA-CCCCCAACTCCTGAAAATT

>Diaporthe_neoarctii_CBS_109490

ACAAGGTCTCCGTTGGTGAACCAGCGGAGGGATCATTGCTGGAACGCG-CCTC-GGCGCA-CCCAGAAACCCTTTGTGAACTTATACCCAAACTGTTGCCTCGGCG-CAGGCCGGCCCCT--CT--------CGTTAGGGGCC-----CCCTG---GAGACAGGGAGCA-GCCCGCCGGCGGCCGA-CCAAACTC-TGTTTCTATAGTGAATCTCTGAGT--AAAAAACATAAATGAATCAAAACTTTCAACAACGGATCTCTTGGTTCTGGCATCGATGAAGAACGCAGCGAAATGCGATAAGTAATGTGAATTGCAGAATTCAGTGAATCATCGAATCTTTGAACGCACATTGCGCCCTCTGGTATTCCGGAGGGCATGCCTGTTCGAGCGTCATTTCAACCCTCAAGCCTGGCTTGG-TGATGGGGCACTGCCTGT--------TAAAGGGCAGGCCCTGAAATCTAGTGGCGAGCT-CGCCAGGAC-CCCGAGCGTAGTAG-TTACA-TCTCGCTCTGG-AAGGCCCTGGCGGTGCCCTGCCGTTAAA-CCCCCAACTTCTGAAAATT

>Diaporthe_neoraonikayaporum_MFLUCC_14_1136

ACAAGGTCTCCGTTGGTGAACCAGCGGAGGGATCATTGCTGGAACGCG-CTTC-GGCGCA-CCCAGAAACCCTTTGTGAACTTATACCT-TACTGTTGCCTCGGCG-CAGGCCGGCCTCT--C------------CTGAGGCC-----CCTCC---GGAAGGAGGAGCA-GCCCGCCGGCGGCCAG-CCAAACTCTTGTTTCT-TAGTGAATCTCTGAGT--AAACAACACAAATGAATCAAAACTTTCAACAACGGATCTCTTGGTTCTGGCATCGATGAAGAACGCAGCGAAATGCGATAAGTAATGTGAATTGCAGAATTCAGTGAATCATCGAATCTTTGAACGCACATTGCGCCCTTTGGTATTCCGAAGGGCATGCCTGTTCGAGCGTCATTTCAACCCTCAAGCCCGGCTTGG-TGTTGGGGCACTACTCCGA-------AGAGGAGTAGGCCCTGAAATCTAGTGGCGAGCT-CGCCAGGAC-CCCGAGCGTAGTAGTTTATA-TCTCGCTCTGG-AAGGCCCTGGCGGTGCCCTGCCGTTAAA-CCCCCAACTTCTGAAAATT

>Diaporthe_novem_CBS_127271

ACAAGGTCTCCGTTGGTGAACCAGCGGAGGGATCATTGCTGGAACGCG-CTTC-GGCGCA-CCCAGAAACCCTTTGTGAACTTATACCC--ACTGTTGCCTCGGCG-CAGGCCGGCCTCT--TC----------ACTGAGGCC-----CCCTG---GAAACAGGGAGCA-GCCCGCCGGTGGCCAA-CTAAACTC-TGTTTCTATAGTGAATCTCTGAGT--AAAAAACATAAATGAATCAAAACTTTCAACAACGGATCTCTTGGTTCTGGCATCGATGAAGAACGCAGCGAAATGCGATAAGTAATGTGAATTGCAGAATTCAGTGAATCATCGAATCTTTGAACGCACATTGCGCCCTCTGGTATTCCGGAGGGCATGCCTGTTCGAGCGTCATTTCAACCCTCAAGCCTGGCTTGG-TGATGGGGCACTGCCTGT--------AAAAGGGCAGGCCCTGAAATCTAGTGGCGAGCT-CGCCAGGAC-CCCGAGCGTAGTAG-TTATA-TCTCGCTCTGG-AAGGCCCTGGCGGTGCCCTGCCGTTAAA-CCCCCAACTTCTGAAAATT

>Diaporthe_ovalispora_ICMP20659

??????????????????????????????????????CTGGAACGCG-CCTC-GGCGCA-CCCAGAAACCCTTTGTGAACTTATACCTA--CTGTTGCCTCGGCG-CAGGCCGGCTTTTT-TT----------CTAAAAGCC-----CCCTG---GAAACAGGGAGCA-GCCCGCCGGCGGCCGA-CCAAACTCTTGTTTCTACAGTGGATCTCTGAGA--AAAAAACATAAATGAATCAAAACTTTCAACAACGGATCTCTTGGTTCTGGCATCGATGAAGAACGCAGCGAAATGCGATAAGTAATGTGAATTGCAGAATTCAGTGAATCATCGAATCTTTGAACGCACATTGCGCCCTCTGGTATTCCGGAGGGCATGCCTGTTCGAGCGTCATTTCAACCCTCAAGCCTGGCTTGG-TGATGGGGCACTGCCTTCGCC-----CAGGAGGCAGGCCCTGAAATCTAGTGGCGAGCT-CGCCAGGAC-CCCGAGCGCAGTAG-TCATA-TCTCGCTCTGG-AAGGCCCTGGCGGTGCCCTGCCGTTAAA-CCCCCAACTTCTGAAAATT

>Diaporthe_pachirae_CDA_728

NNNNNNNNNNNNNNNNNNNNNNNNCGGAGGGATCATTGCTGGAACGCG-CTTC-GGCGCA-CCCAGAAACCCTTTGTGAACTTATACCT--ATTGTTGCCTCGGCGTCAGGCCGGCCTCT--TC----------ACTGAGGCC-----CCCTG---GAGACAGGGAGCA-GCCCGCCGGCGGCCAA-CTAAACTCTTGTTTCTATAGTGAATCTCTGAGT--AAAAAACATAAATGAATCAAAACTTTCAACAACGGATCTCTTGGTTCTGGCATCGATGAAGAACGCAGCGAAATGCGATAAGTAATGTGAATTGCAGAATTCAGTGAATCATCGAATCTTTGAACGCACATTGCGCCCCCTGGTATTCCGGGGGGCATGCCTGTTCGAGCGTCATTTCAACCCTCAAGCCTGGCTTGG-TGATGGGGCACTACTTCCTC------ACGGGAGTAGGCCCTGAAATTCAGTGGCGAGCT-CGCCAGGAC-CCCGAGCGTAGTAG-TTATA-TCTCGCTTTGG-AAGGCCCTGGCGGTGCCCTGCCGTTAAA-CCCCCAACTTCTGAAAATT

>Diaporthe_passifloricola_CBS_141329

ACAAGGTCTCCGTTGGTGAACCAGCGGAGGGATCATTGCTGGAACGCG-CCTC-GGCGCA-CCCAGAAACCCTTTGTGAACTTATACCTAT-TTGTTGCCTCGGCG-CCGGCCGGCCTTTTGTG----------ACAAAGGCC-----CCCTG---GAGACAGGGAGCA-GCCCGCCGGCGGCCAA-CTAAACTCTTGTTTCTATAGTGAATCTCTGAGT---AAAAACATAAATGAATCAAAACTTTCAACAACGGATCTCTTGGTTCTGGCATCGATGAAGAACGCAGCGAAATGCGATAAGTAATGTGAATTGCAGAATTCAGTGAATCATCGAATCTTTGAACGCACATTGCGCCCTCTGGTATTCCGGAGGGCATGCCTGTTCGAGCGTCATTTCAACCCTCAAGCCTGGCTTGG-TGATGGGGCACTGCCTTCT------AGCGAGGGCAGGCCCTGAAATCTAGTGGCGAGCT-CGCTAGGAC-CCCGAGCGTAGTAG-TTATA-TCTCGTTCTGG-AAGGCCCTGGCGGTGCCCTGCCGTTAAA-CCCCCAACTTCTGAAAATT

>Diaporthe_pseudolongicolla_CBS_117165

NNNNNNNNNNNNNNNNNNNNNNNNNNNNNNNNTCATTGCTGGAACGCG-CTTC-GGCGCA-CCCAGAAACCCTTTGTGAACTTATACCC--ACTGTTGCCTCGGCG-CAGGCCGGCCTCT--TC----------ACTGAGGCC-----CCCTG---GAAACAGGGAGCA-GCCCGCCGGTGGCCAA-CTAAACTC-TGTTTCTATAGTGAATCTCTGAGT--AAAAAACATAAATGAATCAAAACTTTCAACAACGGATCTCTTGGTTCTGGCATCGATGAAGAACGCAGCGAAATGCGATAAGTAATGTGAATTGCAGAATTCAGTGAATCATCGAATCTTTGAACGCACATTGCGCCCTCTGGTATTCCGGAGGGCATGCCTGTTCGAGCGTCATTTCAACCCTCAAGCCTGGCTTGG-TGATGGGGCACTGCCTGT--------AAAAGGGCAGGCCCTGAAATCTAGTGGCGAGCT-CGCCAGGAC-CCCGAGCGTAGTAG-TTATA-TCTCGCTCTGG-AAGGCCCTGGCGGTGCCCTGCCGTTAAA-CCCCCAACTTCTGAAAATT

>Diaporthe_pyracanthae_CBS142384

NNNNNNNNNNNNNNNNNNNNNNNNNNNAGGGATCATTGCTGGAACGCG-CTTC-GGCGCA-CCCAGAAACCCTTTGTGAACTTATACCT--ATTGTTGCCTCGGCG-TAAGCTGGCTTTG--AA----------ATATAAGCC-----CCCTG---GAAACGGGGAGCA-GCCCGCCGGCGGCCAA-CTAAACTCTTGTTTCTATAGTGAATCTCTGAGT--AAAAAACATAAATGAATCAAAACTTTCAACAACGGATCTCTTGGTTCTGGCATCGATGAAGAACGCAGCGAAATGCGATAAGTAATGTGAATTGCAGAATTCAGTGAATCATCGAATCTTTGAACGCACATTGCGCCCCCTGGTATTCCGGGGGGCATGCCTGTTCGAGCGTCATTTCAACCCTCAAGCCTGGCTTGG-TGATGGGGCACTGCTTCG--------AAAGGAGCAGGCCCTGAAATTCAGTGGCGAGCT-CGCCAGGAC-CCCGAGCGTAGTAG-TTATA-TCTCGCTCTGG-AAGGCCCTGGCGGCGCCCTGCCGTTAAA-CCCCCAACTTCTGAAAATT

>Diaporthe_racemosae_CBS_143770

ACAAGGTCTCCGTTGGTGAACCAGCGGAGGGATCATTGCTGGAACGCG-CTTC-GGCGCA-CCCAGAAACCCTTTGTGAACTTATACCTAT-CTGTTGCCTCGGCG-CAGGCCGGCTTTTTGTG----------ACAAAAGCC-----CCCTG---GAGACAGGGAGCA-GCCCGCCGGCGGCCAA-CTAAACTCTTGTTTCTATAGTGAATCTCTGAGT--AAAAAACATAAATGAATTAAAACTTTCAACAACGGATCTCTTGGTTCTGGCATCGATGAAGAACGCAGCGAAATGCGATAAGTAATGTGAATTGCAGAATTCAGTGAATCATCGAATCTTTGAACGCACATTGCGCCCTCTGGTATTCCGGAGGGCATGCCTGTTCGAGCGTCATTTCAACCCTCAAGCCTGGCTTGG-TGATGGGGCACTGCCTGTA--------AAAGGGCAGGCCCTGAAATCTAGTGGCGAGCT-CGCCAGGAC-CCCGAGCGTAGTAG-TTATA-TCTCGCTTTGG-AAGGCCCTGGCGGTGCCCTGCCGTTAAA-CCCCCAACTCTTGAAAATT

>Diaporthe_raonikayaporum_CBS_133182

ACAAGGTCTCCGTTGGTGAACCAGCGGAGGGATCATTGCTGGAACGCG-CTTC-GGCGCA-CCCAGAAACCCTTTGTGAACTTATACCT-TACTGTTGCCTCGGCG-CAGGCCGGCCTCT--CT----------TCTGAGGCC-----CCTCC---GGAAGGAGGAGCA-GCCCGCCGGCGGCCAG-CTAAACTCTTGTTTCT-TAGTGAATCTCTGAGT--AAAAAACACAAATGAATCAAAACTTTCAACAACGGATCTCTTGGTTCTGGCATCGATGAAGAACGCAGCGAAATGCGATAAGTAATGTGAATTGCAGAATTCAGTGAATCATCGAATCTTTGAACGCACATTGCGCCCTTTGGTATTCCGAAGGGCATGCCTGTTCGAGCGTCATTTCAACCCTCAAGCCTGGCTTGG-TGTTGGGGCACTGCTCCGA-------AGAGGAGTAGGCCCTGAAATCTAGTGGCGAGCT-CGCCAGGAC-CCCGAGCGTAGTAG-TTATA-TCTCGTTCTGG-AAGGCCCTGGCGGTGCCCTGCCGTTAAA-ACCCCAAATTCTGAAAATT

>Diaporthe_rosae_MFLUCC_17_2658

ACAAGGTCTCTGTTGGTGAACCAGCGGAGGGATCATTGCTGGAACGCG-CTTC-GGCGCA-CCCAGAAACCCTTTGTGAACTTATACCTAT-CTGTTGCCTCGGCG-CAGGCCGGCCTCT--TC----------ACTGAGGCC-----CCCTG---GAAACAGGGAGCA-GCCCGCCGGCGGCCAA-CTAAACTCTTGTTTCTATAGTGAATCTCTGAGT--AAAAAACATAAATGAATCAAAACTTTCAACAACGGATCTCTTGGTTCTGGCATCGATGAAGAACGCAGCGAAATGCGATAAGTAATGTGAATTGCAGAATTCAGTGAATCATCGAATCTTTGAACGCACATTGCGCCCTCTGGTATTCCGGAGGGCATGCCTGTTCGAGCGTCATTTCAACCCTCAAGCCTGGCTTGG-TGATGGGGCACTGCCTTCT------AACGAGGGCAGGCCCTGAAATCTAGTGGCGAGCT-CGCTAGGAC-CCCGAGCGTAGTAG-TTATA-TCTCGTTCTGG-AAGGCCCTGGCGGTGCCCTGCCGTTAAA-CCCCCAACTTCTGAAAATT

>Diaporthe_rosiphthora_COAD_2913

??????????????GGTGAACCAGCGGAGGGATCATTGCTGGAACGCG-CTTC-GGCGCA-CCCAGAAACCCTTTGTGAACTTATACCTAT-CTGTTGCCTCGGCG-CAGGCCGGCTTCT--TC----------ACTGAAGCC-----CCCTG---GAGACAGGGAGCA-GCCCGCCGGCGGCCAA-CTAAACTCTTGTTTCTACAGTGAATCTCTGAGT--AAAAAACATAAATGAATCAAAACTTTCAACAACGGATCTCTTGGTTCTGGCATCGATGAAGAACGCAGCGAAATGCGATAAGTAATGTGAATTGCAGAATTCAGTGAATCATCGAATCTTTGAACGCACATTGCGCCCTCTGGTATTCCGGAGGGCATGCCTGTTCGAGCGTCATTTCAACCCTCAAGCCTGGCTTGG-TGATGGGGCACTACTTCCT------TACGGGAGTAGGCCCTGAAATTCAGTGGCGAGCT-CGCCAGGAC-CCCGAGCGTAGTAG-TTATA-TCTCGCTTTGG-AAGGCCCTGGCGGTGCCCTGCCGTTAAA-CCCCCAACTTCTGAAAATT

>Diaporthe_rossmaniae_CAA762

NNNNNNNNNNNNNNNNNNNNNNNNNNNAGGGATCATTGCTGGAACGCG-CTTC-GGCGCA-CCCAGAAACCCTTTGTGAACTTATACCT--ATTGTTGCCTCGGCG-TAAGCTGGCTTTG--AA----------ATATAAGCC-----CCCTG---GAAACGGGGAGCA-GCCCGCCGGCGGCCAA-CTAAACTCTTGTTTCTATAGTGAATCTCTGAGT--AAAAAACATAAATGAATCAAAACTTTCAACAACGGATCTCTTGGTTCTGGCATCGATGAAGAACGCAGCGAAATGCGATAAGTAATGTGAATTGCAGAATTCAGTGAATCATCGAATCTTTGAACGCACATTGCGCCCCCTGGTATTCCGGGGGGCATGCCTGTTCGAGCGTCATTTCAACCCTCAAGCCTGGCTTGG-TGATGGGGCACTGCTTCG--------AAAGGAGCAGGCCCTGAAATTCAGTGGCGAGCT-CGCCAGGAC-CCCGAGCGTAGTAG-TTATA-TCTCGCTCTGG-AAGGCCCTGGCGGCGCCCTGCCGTTAAA-CCCCCAACTTCTGAAAATT

>Diaporthe_sackstonii_BRIP_54669b

ACAAGGTCTCCGTTGGTGAACCAGCGGAGGGATCATTGCTGGAACGCG-CTTC-GGCGCA-CCCAGAAACCCTTTGTGAACTTATACCT--ATTGTTGCCTCGGCGTCAGGCCGGCCTCT--TC----------ACTGAGGCC-----CCCCG---GAGACGGGGAGCA-GCCCGCCGGCGGCCAA-CTAAACTCTTGTTTCTATAGTGAATCTCTGAGT--AAAAAACATAAATGAATCAAAACTTTCAACAACGGATCTCTTGGTTCTGGCATCGATGAAGAACGCAGCGAAATGCGATAAGTAATGTGAATTGCAGAATTCAGTGAATCATCGAATCTTTGAACGCACATTGCGCCCCCTGGTATTCCGGGGGGCATGCCTGTTCGAGCGTCATTTCAACCCTCAAGCCTGGCTTGG-TGATGGGGCACTGCTTCG--------AGAGGAGCAGGCCCTGAAATTCAGTGGCGAGCT-CGCCAGGAC-CCCGAGCGTAGTAG-TTATA-TCTCGCTCTGG-AAGGCCCTGGCGGTGCCCTGCCGTTAAA-CCCCCAACTTCTGAAAATT

>Diaporthe_schini_CBS_133181

ACAAGGTCTCCGTTGGTGAACCAGCGGAGGGATCATTGCTGGAACGCG-CTTC-GGCGCA-CCCAGAAACCCTTTGTGAACTTATACCTAT-CTGTTGCCTCGGCG-CAGGCCGGCTTCT--TC----------ACTGAAGCC-----CCCTG---GAGACAGGGAGCA-GCCCGCCGGCGGCCAA-CTAAACTCTTGTTTCTATAGTGAATCTCTGAGT--AAAAAACATAAATGAATCAAAACTTTCAACAACGGATCTCTTGGTTCTGGCATCGATGAAGAACGCAGCGAAATGCGATAAGTAATGTGAATTGCAGAATTCAGTGAATCATCGAATCTTTGAACGCACATTGCGCCCTCTGGTATTCCGGAGGGCATGCCTGTTCGAGCGTCATTTCAACCCTCAAGCCTGGCTTGG-TGATGGGGCACTGCCTGTA--------AAAGGGCAGGCCCTGAAATTCAGTGGCGAGCT-CGCCAGGAC-CCCGAGCGTAGTAG-TTATA-TCTCGCTTTGG-AAGGCCCTGGCGGTGCCCTGCCGTTAAA-CCCCCAACTTCTGAAAATT

>Diaporthe_schoeni_MFLU_15_1279

NNNNNNNNNNNNNNNNNNNNNNNNNNNNNNNNNNNNNNNNNNNNNNNNNNNNNNNNNNNNNNCCAG-AACCCTTTGTG-ACTTATACCCATACTGTTGCCTCGGCG-CAGGCCGGCCTTT--TT--------CGATAAGGGCC-----CCCTG---GAGACAGGGAGCA-GCCCGCCGGCGGCCAA-CCCAAACTCTGTTTCTATAGTGAATCTCTGAGT--AAAAAACATAAATGAATCAAAACTTTCAACAACGGATCTCTTGGTTCTGGCATCGATGAAGAACGCAGCGAAATGCGATAAGTAATGTGAATTGCAGAATTCAGTGAATCATCGAATCTTTGAACGCACATTGCGCCCTCTGGTATTCCGGAGGGCATGCCTGTTCGAGCGTCATTTCAACCCTCAAGCCTGGCTTGG-TGATGGGGCACTGCCTGT--------AAAAGGGCAGGCCCTGAAATCTAGTGGCGAGCT-CGCCAGGAC-CCCGAGCGTAGTAG-TTACA-TCTCGCTCTGG-AAGGCCCTGGCGGTGCCCTGCCGTTAAA-CCCCCAACTTCTGAAAATT

>Diaporthe_sclerotioides_CBS_296_67

ACAAGGTCTCCGTTGGTGAACCAGCGGAGGGATCATTGCTGGAACGCG-CTTC-GGCGCA-CCCAGAAACCCTTTGTGAACTTATACCT-TACTGTTGCCTCGGCG-CAGGCCGGCCTC---------------ACCGAGGCC-----CCTCG---GAAACGAGGAGCA-GCCCGCCGGCGGCCGA-CCAAACTCTTGTTTCT-CAGTGGATCTCTGAGT--AAAAAA-AAAAATGAATCAAAACTTTCAACAACGGATCTCTTGGTTCTGGCATCGATGAAGAACGCAGCGAAATGCGATAAGTAATGTGAATTGCAGAATTCAGTGAATCATCGAATCTTTGAACGCACATTGCGCCCTCTGGTATTCCGGAGGGCATGCCTGTTCGAGCGTCATTTCAACCCTCAAGCACTGCTTGG-TGTTGGGGCACCGCCTGTA--------AAAGGGCGGGCCCTGAAATCTAGTGGCGAGCT-CGCCGGGAC-CCCGAGCGTAGTAAATTATA-TTTCGTTCTGG-AAGGCCCCGGCGGTGCCCTGCCGTTAAA-CCCCCAACTCCTGAAAATT

>Diaporthe_serafiniae_BRIP_55665a

ACAAGGTCTCCGTTGGTGAACCAGCGGAGGGATCATTGCTGGAACGCG-CTTC-GGCGCA-CCCAGAAACCCTTTGTGAACTTATACCT--ATTGTTGCCTCGGCGTTAGGCCGGCCTCT--TC----------ACTGAGGCC-----CCCTG---GAAACGGGGAGCA-GCCCGCCGGCGGCCAA-CTAAACTCTTGTTTCTATAGTGAATCTCTGAGT--AAAAAACATAAATGAATCAAAACTTTCAACAACGGATCTCTTGGTTCTGGCATCGATGAAGAACGCAGCGAAATGCGATAAGTAATGTGAATTGCAGAATTCAGTGAATCATCGAATCTTTGAACGCACATTGCGCCCCCTGGTATTCCGGGGGGCATGCCTGTTCGAGCGTCATTTCAACCCTCAAGCCTGGCTTGG-TGATGGGGCACTGCTTCG--------AGAGGAGCAGGCCCTGAAATTCAGTGGCGAGCT-CGCCAGGAC-CCCGAGCGTAGTAG-TTATA-TCTCGCT-TAA-AGGGCCCTGGCGGTGCCCTGCCGTTAAA-CCCCCAACTNNNNNNNNNN

>Diaporthe_siamensis_MFLUCC_10_0573a

NNNNNNNNNNNNNNNNNNNNNNNNNNNNNNNNNNNNNNNNNNNNNNNNNNNNNNNNNNNNNNNNNNNNNCCCTTTGTGAACTTATACCT-TACTGTTGCCTCGGCG-CAGGCCGGCCTCT--TA----------GCTGAGGCC-----CCCCG---GAGACGGGGAGCA-GCCCGCCGGCGGCCAA-CCAAACTCTTGTTTCTACAGTGAATCTCTGAGT--AAAAAACATAAATGAATCAAAACTTTCAACAACGGATCTCTTGGTTCTGGCATCGATGAAGAACGCAGCGAAATGCGATAAGTAATGTGAATTGCAGAATTCAGTGAATCATCGAATCTTTGAACGCACATTGCGCCCTCTGGTATTCCGGAGGGCATGCCTGTTCGAGCGTCATTTCAACCCTCAAGCCTGGCTTGG-TGTTGGGGCACTGCTTCG--------AGAGAAGCAGGCCCTGAAATCTAGTGGCGAGCT-CGCTAGGAC-CCCGAGCGTAGTAG-TTATA-TCTCGTTCTGG-AAGGCCCTGGCGGTGCCCTGCCGTTAAA-CCCCCAACTTCTGAAATTT

>Diaporthe_sinensis_ZJUP0033_4

NNNNNNNNNNNNNNNNNNNNNNNNNNNNNNNNNNNNNNNNNNNNNNNNNNNNNNNNNNNNNNNNNNNNNNNNNNNNNNNNNNNNNNNNNNNNNNNNNNNNNNNNNNNNNNNNNNNNNNNNNNNNNNNNNNNNNNNNNCCCCCC-----CCCCC---CGGGGGGGGAGCA-GCCCGCCGGCGGCCAACCCAAACTCCTGATTCTGCAGTGGATCTCTGAGC-AAAAAAACACAAATGAATCAAAACTTTCAACAACGGATCTCTTGGTTCTGGCATCGATGAAGAACGCAGCGAAATGCGATAAGTAATGTGAATTGCAGAATTCAGTGAATCATCGAATCTTTGAACGCACATTGCGCCCTCCGGCATTCCGGAGGGCATGCCTGTTCGAGCGTCATTTCAACCCTCAAGCCTGGCTTGG-TGATGGGGCGCTGCCCGTA--------GAAGGGCAGGCCCTGAAATCTAGTGGCGGGCC-CGCCGGGAC-CCCGAGCGTAGTAGCCTACA-CCTCGCTCCGG-GAGGCCCCGGCGGTGCCCTGCCGTTAAA-CCCCCAACACCCGAAATCT

>Diaporthe_stewartii_CBS_193_36

NNNNNNNNNNNNNNNNNNNNNNNNNNNAGGGATCATTGCTGGAACGCG-CCCC-GGCGCA-CCCAGAAACCCTTTGTGAACTTATACCCATACTGTTGCCTCGGCG-CAGGCCGGCCTTT--TT--------CGATAAGGGCC-----CCCTG---GAGACAGGGAGCA-GCCCGCCGGCGGCCAA-CCAAACTC-TGTTTCTATAGTGAATCTCTGAGT--AAAAAACATAAATGAATCAAAACTTTCAACAACGGATCTCTTGGTTCTGGCATCGATGAAGAACGCAGCGAAATGCGATAAGTAATGTGAATTGCAGAATTCAGTGAATCATCGAATCTTTGAACGCACATTGCGCCCTCTGGTATTCCGGAGGGCATGCCTGTTCGAGCGTCATTTCAACCCTCAAGCCTGGCTTGG-TGATGGGGCACTGCCTGT--------GAAAGGGCAGGCCCTGAAATCTAGTGGCGAGCT-CGCCAGGAC-CCCGAGCGTAGTAG-TTACA-TCTCGCTCTGG-AAGGCCCTGGCGGTGCCCTGCCGTTAAA-CCCCCAACTTCTGAAAATT

>Diaporthe_subordinaria_CBS_101711

ACAAGGTCTCCGTTGGTGAACCAGCGGAGGGATCATTGCTGGAACGCG-CCTC-GGCGCA-CCCAGAAACCCTTTGTGAACTTATACCCATACTGTTGCCTCGGCG-CAGGCCGGCCTTT--CT--------CGGTAAAGGCC-----CCCTG---GAGACAGGGAGCA-GCCCGCCGGCGGCCAG-CCAAACTC-TGTTTCTATAGTGGATCTCTGAGT--AAAAAACATAAATGAATCAAAACTTTCAACAACGGATCTCTTGGTTCTGGCATCGATGAAGAACGCAGCGAAATGCGATAAGTAATGTGAATTGCAGAATTCAGTGAATCATCGAATCTTTGAACGCACATTGCGCCCTCTGGTATTCCGGAGGGCATGCCTGTTCGAGCGTCATTTCAACCCTCAAGCCTGGCTTGG-TGATGGGGCACTGCCTGT--------GAAAGGGCAGGCCCTGAAATCTAGTGGCGAGCT-CGCCAGGAC-CCCGAGCGTAGTAG-TTACA-TCTCGCTCTGG-GAGGCCCTGGCGGTGCCCTGCCGTTAAA-CCCCCAACTTCTGAAAATT

>Diaporthe_tecomae_CBS_100547

ACAAGGTCTCCGTTGGTGAACCAGCGGAGGGATCATTGCTGGAACGCG-CCTC-GGCGCA-CCCAGAAACCCTTTGTGAACGTATACCTAC-CTGTTGCCTCGGCGTTAGGCCGGCCTTTTGTG----------ACAAAGGCC-----CCCTG---GAGACAGGGAGCA-GCCCGCCGGCGGCCAA-CTAAACTCTTGTTTCTATAGTGAATCTCTGAG---AAAAAACATAAATGAATCAAAACTTTCAACAACGGATCTCTTGGTTCTGGCATCGATGAAGAACGCAGCGAAATGCGATAAGTAATGTGAATTGCAGAATTCAGTGAATCATCGAATCTTTGAACGCACATTGCGCCCTCTGGTATTCCGGAGGGCATGCCTGTTCGAGCGTCATTTCAACCCTCAAGCCTGGCTTGG-TGATGGGGCACTGCTTTTGTT-----ATAAAAGCAGGCCCTGAAATTCAGTGGCGAGCT-CGCCAGGAC-CCCGAGCGTAGTAG-TTATA-TCTCGCTTTTG-GAGGCCCTGGCGGTGCCCTGCCGTTAAA-CCCCCAACTTCTGAAAATT

>Diaporthe_tectonae_MFLUCC_12_0777

ACAAGGTCTCCGTTGGTGAACCAGCGGAGGGATCATTGCTGGAACGCG-CTTC-GGCGCA-CCCAGAAACCCTTTGTGAACTTATACCTATACTGTTGCCTCGGCG-CTGGCCGGCCTCC--TC----------ACCGAGGCC-----CCCTG---GAGACAGGGAGCA-GCCCGCCGGCGGCCAA-ACAAACTCTTGTTTCT-TAGTGAATCTCTGAGT--AAAAAACAT-AATGAATCAAAACTTTCAACAACGGATCTCTTGGTTCTGGCATCGATGAAGAACGCAGCGAAATGCGATAAGTAATGTGAATTGCAGAATTCAGTGAATCATCGAATCTTTGAACGCACATTGCGCCCTCTGGTATTCCGGAGGGCATGCCTGTTCGAGCGTCATTTCAACCCTCAAGCCTGGCTTGG-TGTTGGGGCACCGCCTTTGC------AAAAGGGCGGGCCCTGAAATCTAGTGGCGAGCT-CGCCAGGAC-CCCGAGCGTAGTAG-TTATA-TCTCGTTCTGG-AAGGCCCTGGCGGTGCCCTGCCGTTAAA-CCCCCAACTTCTGAAA-TT

>Diaporthe_tectonendophytica_MFLUCC_13_0471

ACAAGGTCTCCGTTGGTGAACCAGCGGAGGGATCATTGCTGGAACGCG-CTTC-GGCGCA-CCCAGAAACCCTTTGTGAACTTATACCTA--CTGTTGCCTCGGCG-CAGGCCGGCCTCT--TC----------GCTGAGGCC-----CCCTG---GAGACAGGGAGCA-GCCCGCCGGCGGCCAA-CTAAACTCTTGTTTCTTTAGTGAATCTCTGAGT--AAAAAACATAAATGAATCAAAACTTTCAACAACGGATCTCTTGGTTCTGGCATCGATGAAGAACGCAGCGAAATGCGATAAGTAATGTGAATTGCAGAATTCAGTGAATCATCGAATCTTTGAACGCACATTGCGCCCTCTGGTATTCCGGAGGGCATGCCTGTTCGAGCGTCATTTCAACCCTCAAGCCTGGCTTGG-TGATGGGGCACTGCTCTCT------AGCGGGAGCAGGCCCTGAAATCTAGTGGCGAGCT-CGCCAGGAC-CCCGAGCGTAGTAG-TTATA-TCTCGTTCTGG-AAGGCCCTGGCGGTGCCCTGCCGTTAAA-CCCCCAACTTCTGAAAATT

>Diaporthe_terebinthifolii_CBS_133180

ACAAGGTCTCCGTTGGTGAACCAGCGGAGGGATCATTGCTGGAACGCG-CTTC-GGCGCA-CCCAGAAACCCTTTGTGAACTTATACCTAT-CTGTTGCCTCGGCGTCAGGCCGGCCTCT--TC----------ACTGAGGCC-----CCCTG---GAAACAGGGAGCA-GCCCGCCGGCGGCCAA-CTAAACTCTTGTTTCTATAGTGAATCTCTGAGT--AAAAAACATAAATGAATCAAAACTTTCAACAACGGATCTCTTGGTTCTGGCATCGATGAAGAACGCAGCGAAATGCGATAAGTAATGTGAATTGCAGAATTCAGTGAATCATCGAATCTTTGAACGCACATTGCGCCCTCTGGTATTCCGGAGGGCATGCCTGTTCGAGCGTCATTTCAACCCTCAAGCCTGGCTTGG-TGATGGGGCACTGCCTGTA--------AAAGGGCAGGCCCTGAAATCTAGTGGCGAGCT-CGCCAGGAC-CCCGAGCGTAGTAG-TTATA-TCTCGCTTTGG-AAGGCCCTGGCGGTGCCCTGCCGTTAAA-CCCCCAACTTCTGAAAATT

>Diaporthe_thunbergiicola_MFLUCC_12_0033

ACAAGGTCTCCGTTGGTGAACCAGCGGAGGAATCATTGCGAGCGCGCA-TTCC--GCGCA-CCGAGAAACCCTTTGTGAACTTATACCT-TACTGTTGCCTCGGCG-CAGGCCGGCCTCT--------------GCTGAGGCC-----CCCCG---GAGACGGGGAGCA-GCCCGCCGGCGGCCAA-CCAAACTCTTGTTTCTACAGTGAATCTCTGAGT--AAAAAACATAAATGAATCAAAACTTTCAACAACGGATCTCTTGGTTCTGGCATCGATGAAGAACGCAGCGAAATGCGATAAGTAATGTGAATTGCAGAATTCAGTGAATCATCGAATCTTTGAACGCACATTGCGCCCTCTGGTATTCCGGAGGGCATGCCTGTTCGAGCGTCATTTCAACCCTCAAGCCTGGCTTGG-TGTTGGGGCACTGCTCCGA--------GAGGAGCAGGCCCTGAAATCTAGTGGCGAGCT-CGCTAGGAC-CCCGAGCGTAGTAG-TTATA-TCTCGTTCTGG-AAGGCCCTGGCGGTGCCCTGCCGTTAAA-CCCCCAACTTCTGAAATTT

>Diaporthe_tulliensis_BRIP_62248a

ACAAGGTCTCCGTTGGTGAACCAGCGGAGGGATCATTGCTGGAACGCG-CTTC-GGCGCA-CCCAGAAACCCTTTGTGAACTTATACCTATACTGTTGCCTCGGCG-CTGGCCGGCCTCC--TC----------ACCGAGGCC-----CCCTG---GAGACAGGGAGCA-GTCCGCCGGCGGCCAA-CCAAACTCTTGTTTCT-TAGTGAATCTCTGAGT--AAAAAACAT-AATGAATCAAAACTTTCAACAACGGATCTCTTGGTTCTGGCATCGATGAAGAACGCAGCGAAATGCGATAAGTAATGTGAATTGCAGAATTCAGTGAATCATCGAATCTTTGAACGCACATTGCGCCCTCTGGTATTCCGGAGGGCATGCCTGTTCGAGCGTCATTTCAACCCTCAAGCCTAGCTTGGTTGTTGGGGCACCGCCTTCGC------AAGAGGGCGGGCCCTGAAATCTAGTGGCGAGCT-CGCCAGGAC-CCCGAGCGTAGTAG-TTATA-TCTCGTTCTGG-AAGGCCCTGGCGGTGCCCTGCCGTTAAA-CCCCCAACTTCTGAAATTT

>Diaporthe_ueckerae_FAU_656

???????????????????????GCGGAGGGATCATTGCTGGAACGCG-CTTC-GGCGCA-CCCAGAAACCCTTTGTGAACTTATACCTAT-TTGTTGCCTCGGCC-TAGGCCGGCCTCT--TC----------ACTGAGGCC-----CCCTG---GAGACAGGGAGCA-GCCCGCCGGCGGCCAA-CTAAACTCTTGTTTCTATAGTGAATCTCTGAGT--AAAAAACATAAATGAATCAAAACTTTCAACAACGGATCTCTTGGTTCTGGCATCGATGAAGAACGCAGCGAAATGCGATAAGTAATGTGAATTGCAGAATTCAGTGAATCATCGAATCTTTGAACGCACATTGCGCCCTCTGGTATTCCGGAGGGCATGCCTGTTCGAGCGTCATTTCAACCCTCAAGCCTGGCTTGG-TGATGGGGCACTGCCTTCT------AGCGAGGGCAGGCCCTGAAATCTAGTGGCGAGCT-CGCTAGGAC-CCCGAGCGTAGTAG-TTATA-TCTCGTTCTGG-AAGGCCCTGGCGGTGCACTGCCGTTAAA-CCCCCAACTTCTGAAAATT

>Diaporthe_unshiuensis_CGMCC3_17569

??????????????????????????????????????CTGGAACGCG-CTTC-GGCGCA-CCCAGAAACCCTTTGTGAACTTATACCTAT--TGTTGCCTCGGCG-CAGGCCGGCCTCT--TC----------ACTGAGGCC-----CCCTG---GAGACAGGGAGCA-GCCCGCCGGCGGCCAA-CCAAACTCTTGTTTCTACAGTGAATCTCTGAGT--ACAAAACATAAATGAATCAAAACTTTCAACAACGGATCTCTTGGTTCTGGCATCGATGAAGAACGCAGCGAAATGCGATAAGTAATGTGAATTGCAGAATTCAGTGAATCATCGAATCTTTGAACGCACATTGCGCCCTCTGGTATTCCGGAGGGCATGCCTGTTCGAGCGTCATTTCAACCCTCAAGCCTGGCTTGG-TGATGGGGCACTGCTCTCT------GACGAGAGCAGGCCCTGAAATCTAGTGGCGAGCT-CGCTAGGAC-CCCGAGCGTAGTAG-TTATA-TCTCGTTCTGG-AAGGCCCTGGCGGTGCCCTGCCGTTAAA-CCCCCAACTTCTGAAAATT

>Diaporthe_vexans_CBS_127_14

ACAAGGTCTCCGTTGGTGAACCAGCGGAGGGATCATTGCTGGAACGCG-CCTC-GGCGCA-CCCAGAAACCCTTTGTGAACTTATACCTAT--TGTTGCCTCGGCG-CAGGCCGGCCTCTCCTG----------GCAGAGGCC-----CCCTG---GAGACAGGGAGCA-GCCCGCCGGCGGCCAG-CTAAACTCTTGTTTCTACAGTGAATCTCTGAGT---AAAAACATAAATGAATCAAAACTTTCAACAACGGATCTCTTGGTTCTGGCATCGATGAAGAACGCAGCGAAATGCGATAAGTAATGTGAATTGCAGAATTCAGTGAATCATCGAATCTTTGAACGCACATTGCGCCCTCTGGTATTCCGGAGGGCATGCCTGTTCGAGCGTCATTTCAACCCTCAAGCCTGGCTTGG-TGATGGGGCACTGCCTGTG--------AAAGGGCAGGCCTTGAAATCTAGTGGCGAGCT-CGCCAGGAC-CCCGAGCGTAGTAG-TATTA-TCTCGCCCTGG-AAGGCCCTGGCGGTGCCCTGCCGTTAAACCCCCCAACTCCTGAAAATT

>Diaporthe_yunnanensis_CGMCC_3_18289

NNNNNNNNNNNNNNNNNNNNNNNGCGGAGGGATCATTGCTGGAACGCG-CTTC-GGCGCA-CCCAGAAACCCTTTGTGAACTTATACCT--ATTGTTGCCTCGGCG-CAGGCCGGCCTCT--TC----------ACTGAGGCC-----CCCTG---GAAACAGGGAGCA-GCCCGCCGGCGGCCAA-CCAAACTCTTGTTTCTATAGTGAATCTCTGAGT-AAAAAAACATAAATGAATCAAAACTTTCAACAACGGATCTCTTGGTTCTGGCATCGATGAAGAACGCAGCGAAATGCGATAAGTAATGTGAATTGCAGAATTCAGTGAATCATCGAATCTTTGAACGCACATTGCGCCCTCTGGTATTCCGGAGGGCATGCCTGTTCGAGCGTCATTTCAACCCTCAAGCCTGGCTTGG-TGATGGGGCACTGCCTGT--------AATAGGGCAGGCCCTGAAATCTAGTGGCGAGCT-CGCCAGGAC-CCCGAGCGTAGTAG-TTATA-TCTCGCTCTGG-AAGGCCCTGGCGGTGCCCTGCCGTTAAA-CCCCCAACTTCTGAAAATT

>Phomopsis_glabrae_SCHM_3622

NNNNNNNNNNNNNNNNNNNNCCAGCGGAGGGATCATTGCTGGAACGCG-CTTC-GGCGCA-CCCAGAAACCCTTTGTGAACTTATACCTATACTGTTGCCTCGGCG-CTGGCCGGCCTCC--TC----------ACCGAGGCC-----CCCTG---GAGACAGGGAGCA-GCCCGCCGGCGGCCAA-ACAAACTCTTGTTTCT-TAGTGAATCTCTGAGT--AAAAAACATAAATGAATCAAAACTTTCAACAACGGATCTCTTGGTTCTGGCATCGATGAAGAACGCAGCGAAATGCGATAAGTAATGTGAATTGCAGAATTCAGTGAATCATCGAATCTTTGAACGCACATTGCGCCCTCTGGTATTCCGGAGGGCATGCCTGTTCGAGCGTCATTTCAACCCTCAAGCCTGGCTTGG-TGTTGGGGCACCGCCTTTGC------AAAAGGGCGGGCCCTGAAATCTAGTGGCGAGCT-CGCCAGGAC-CCCGAGCGTAGTAG-TTATA-TCTCGTTCTGG-AAGGCCCTGGCGGTGCCCTGCCGTTAAA-CCCCCAACTTCTGAAATTT

>Phomopsis_micheliae

NNNNNNNNNNNNNNNTGTGACCAGCGGAGGGATCATTGCTGGAACGCG-CTTC-GGCGCA-CCCAGAAACCCTTTGTGAACTTATACCT--ATTGTTGCCTCGGCGTCAGGCCGGCCTCT--TC----------ACTGAGGCC-----CCCCG---GAGACGGGGAGCA-GCCCGCCGGCGGCCAA-CTAAACTCTTGTTTCTATAGTGAATCTCTGAGT--AAAAAACATAAATGAATCAAAACTTTCAACAACGGATCTCTTGGTTCTGGCATCGATGAAGAACGCAGCGAAATGCGATAAGTAATGTGAATTGCAGAATTCAGTGAATCATCGAATCTTTGAACGCACATTGCGCCCCCTGGTATTCCGGGGGGCATGCCTGTTCGAGCGTCATTTCAACCCTCAAGCCTGGCTTGG-TGATGGGGCACTGCTTCG--------AAAGGAGCAGGCCCTGAAATTCAGTGGCGAGCT-CGCCAGGAC-CCCGAGCGTAGTAG-TTATA-TCTCGCTTTGG-AAGGCCCTGGCGGTGCCCTGCCGTTAAA-CCCCCAACTTCTGAAAATT

>Phomopsis_vitimegaspora_STE_U2675

NNNNNNNNNNNNNNNNNNNNNNNNNNNNNNNNNNNNNNNNNNNNNNNNNNNNNNNNNNNNNNNNNNNAACCCTTTGTGAACTCATACCT-TACTGTTGCCTCGGCG-CAGGCCGGCCTCT--CA----------GCTGAGGCC-----CCCCG---GAGACGGGGAGCA-GCCCGCCGGCGGCCAG-CCAAACTCTTGTTTCTACAGTGAATCTCTGAGT-AAGAAAACATAAATGAATCAAAACTTTCAACAACGGATCTCTTGGTTCTGGCATCGATGAAGAACGCAGCGAAATGCGATAAGTAATGTGAATTGCAGAATTCAGTGAATCATCGAATCTTTGAACGCACATTGCGCCCTCTGGTATTCCGGAGGGCATGCCTGTTCGAGCGTCATTTCAACCCTCAAGCCTGGCTTGG-TGTTGGGGCACTGCCTGT--------GAGAGGGCAGGCCCTGAAATCTAGTGGCGAGCT-CGCCAGGAC-CCCGAGCGTAGTAG-TTATA-TCTCGTTCTGG-AAGGCCCTGGCGGCGCCCTGCCGTTAAA-CCCCCAACTTCTGAAATTT

>Diaporthe_subellipicola_KUMCC_17_0153

ACAAGGTCTCCGTTGGTGAACCAGCGGAGGGATCATTGCTGGAACGCG-CTTC-GGCGCA-CCCAGAAACCCTTTGTGAACTTATACCTAT--TGTTGCCTCGGCG-CAGGCCGGCCTCT--TC----------ACTGAGGCC-----CCCTG---GAAACAGGGAGCA-GCCCGCCGGCGGCCAA-CCAAACTCTTGTTTCTATAGTGAATCTCTGAGT-AAAAAAACATAAATGAATCAAAACTTTCAACAACGGATCTCTTGGTTCTGGCATCGATGAAGAACGCAGCGAAATGCGATAAGTAATGTGAATTGCAGAATTCAGTGAATCATCGAATCTTTGAACGCACATTGCGCCCTCTGGTATTCCGGAGGGCATGCCTGTTCGAGCGTCATTTCAACCCTCAAGCCTGGCTTGG-TGATGGGGCACTGCCTGTA--------ATAGGGCAGGCCCTGAAATCTAGTGGCGAGCT-CGCCAGGAC-CCCGAGCGTAGTAG-TTATA-TCTCGCTCTGG-AAGGCCCTGGCGGTGCCCTGCCGTTAAA-CCCCCAACTTCTGAAAATT

>Diaporthe_masirevicii_BRIP_57892a

ACAAGGTCTCCGTTGGTGAACCAGCGGAGGGATCATTGCTGGAACGCG-CTTC-GGCGCA-CCCAGAAACCCTTTGTGAACTTATACCTAT--TGTTGCCTCGGCG-TAGGCCGGCCTCT--TC----------ACTGAGGCC-----CCCTG---GAAACAGGGAGCA-GCCCGCCGGCGGCCAA-CCAAACTCTTGTTTCTATAGTGAATCTCTGAGT--AAAAAACATAAATGAATCAAAACTTTCAACAACGGATCTCTTGGTTCTGGCATCGATGAAGAACGCAGCGAAATGCGATAAGTAATGTGAATTGCAGAATTCAGTGAATCATCGAATCTTTGAACGCACATTGCGCCCTCTGGTATTCCGGAGGGCATGCCTGTTCGAGCGTCATTTCAACCCTCAAGCCTGGCTTGG-TGATGGGGCACTGCCTGTA--------AGAGGGCAGGCCCTGAAATCTAGTGGCGAGCT-CGCCAGGAC-CCCGAGCGTAGTAA-TTATA-TCTCGCTCTGG-AAGGCCCTGGCGGTGCCCTGCCGTTAAA-CCCCCAACTTCTGAAAATT

>Diaporthe_sojae_CBS_139282

???????????????????????GCGGAGGGATCATTGCTGGAACGCG-CTTC-GGCGCACCCCAGAAACCCTTTGTGAACTTATACCTAT--TGTTGCCTCGGCG-TAGGCCGGCCTCT--TC----------ACTGAGGCC-----CCCTG---GAAACAGGGAGCA-GCCCGCCGGCGGCCAA-CCAAACTCTTGTTTCTACAGTGAATCTCTGAGT--AAAAAACATAAATGAATCAAAACTTTCAACAACGGATCTCTTGGTTCTGGCATCGATGAAGAACGCAGCGAAATGCGATAAGTAATGTGAATTGCAGAATTCAGTGAATCATCGAATCTTTGAACGCACATTGCGCCCTCTGGTATTCCGGAGGGCATGCCTGTTCGAGCGTCATTTCAACCCTCAAGCCTGGCTTGG-TGATGGGGCACTGCTTTCGTC-----CAGAAAGCAGGCCCTGAAATCTAGTGGCGAGCT-CGCCAGGAC-CCCGAGCGTAGTAG-TCATA-TCTCGCTCTGG-AAGGCCCTGGCGGTGCCCTGCCGTTAAA-CCCCCAACTTCTGAAAATT

>Diaporthe_sambucusii_CFCC_51986

NNNNNNNNNNNNNNNNNNNNNNNNNNNNNNNNNNNNNNNNNNNNNNNNNNNNNNNNNNNNNNNNNNNNNCCCTTTGTGAACTTATACCC--ACTGTTGCCTCGGCG-CAGGCCGGTCTGT---------------CTCAGACC-----CCCTG---GAAACAGGGAGCA-GCCCGCCGGCGGCCAA-CCAAACTC-TGTTTCTATAGTGAATCTCTGAGT--AAAAAACATAAATGAATCAAAACTTTCAACAACGGATCTCTTGGTTCTGGCATCGATGAAGAACGCAGCGAAATGCGATAAGTAATGTGAATTGCAGAATTCAGTGAATCATCGAATCTTTGAACGCACATTGCGCCCTCTGGTATTCCGGAGGGCATGCCTGTTCGAGCGTCATTTCAACCCTCAAGCCTGGCTTGG-TGATGGGGCAGTGCCTTGGA------GACAAGGCACGCCCTGAAATTCAGTGGCGAGCT-CGCCAGGAC-CCCGAGCGTAGTAG-TTACA-TCTCGCTCTGG-AAGGCCCTGGCGGTGCCCTGCCGTTAAA-CCCCCAACTTCTGAAANNN

>Diaporthe_vochysiae_LGMF1583

ACAAGGTCTCCGTTGGTGAACCAGCGGAGGGATCATTGCTGGAACGCG-CTTC-GGCGCA-CCCAGAAACCCTTTGTGAACTTATACCTAT-CTGTTGCCTCGGCG-CAGGCCGGCCTTTTGTG----------ACAAAGGCC-----CCCTG---GAGACAGGGAGCA-GCCCGCCGGCGGCCAA-CCAAACTCTTGTTTCTGTAGTGAATCTCTGAGT--AAAAAACATAAATGAATCAAAACTTTCAACAACGGATCTCTTGGTTCTGGCATCGATGAAGAACGCAGCGAAATGCGATAAGTAATGTGAATTGCAGAATTCAGTGAATCATCGAATCTTTGAACGCACATTGCGCCCTCTGGTATTCCGGAGGGCATGCCTGTTCGAGCGTCATTTCAACCCTCAAGCCTGGCTTGG-TGATGGGGCACTGCCTTCT------AACGAGGGCAGGCCCTGAAATCTAGTGGCGAGCT-CGCTAGGAC-CCCGAGCGTAGTAG-TTATA-TCTCGTTCTGG-AAGGCCCTGGCGGTGCCCTGCCGTTAAA-CCCCCAACTTCTGAAAATT

>Diaporthe_endophytica_CBS_133811

ACAAGGTCTCCGTTGGTGAACCAGCGGAGGGATCATTGCTGGAACGCG-CTTC-GGCGCA-CCCAGAAACCCTTTGTGAACTTATACCTAT--TGTTGCCTCGGCG-TAGGCCGGCCTCT--TC----------ACTGAGGCC-----CCCTG---GAAACAGGGAGCA-GCCCGCCGGCGGCCAA-CCAAACTCTTGTTTCTACAGTGAATCTCTGAGT--AAAAAACATAAATGAATCAAAACTTTCAACAACGGATCTCTTGGTTCTGGCATCGATGAAGAACGCAGCGAAATGCGATAAGTAATGTGAATTGCAGAATTCAGTGAATCATCGAATCTTTGAACGCACATTGCGCCCTCTGGTATTCCGGAGGGCATGCCTGTTCGAGCGTCATTTCAACCCTCAAGCCTGGCTTGG-TGATGGGGCACTGCCTGTA--------AAAGGGCAGGCCCTGAAATCTAGTGGCGAGCT-CGCCAGGAC-CCCGAGCGTAGTAG-TTATA-TCTCGCTCTGG-AAGGCCCTGGCGGTGCCCTGCCGTTAAA-CCCCCAACTTCTGAAAATT

>Diaporthe_phaseolorum_CBS_113425

ACAAGGTCTCCGTTGGTGAACCAGCGGAGGGATCATTGCTGGAACGCG-CTTC-GGCGCA-CCCAGAAACCCTTTGTGAACTTATACCTAT--TGTTGCCTCGGCG-TAGGCCGGCCTCT--TC----------ACTGAGGCC-----CCCTG---GAAACAGGGAGCA-GCCCGCCGGCGGCCAA-CCAAACTCTTGTTTCTACAGTGAATCTCTGAGT--AAAAAACATAAATGAATCAAAACTTTCAACAACGGATCTCTTGGTTCTGGCATCGATGAAGAACGCAGCGAAATGCGATAAGTAATGTGAATTGCAGAATTCAGTGAATCATCGAATCTTTGAACGCACATTGCGCCCTCTGGTATTCCGGAGGGCATGCCTGTTCGAGCGTCATTTCAACCCTCAAGCCTGGCTTGG-TGATGGGGCACTGCTTTCGTC-----CAGAAAGCAGGCCCTGAAATCTAGTGGCGAGCT-CGCCAGGAC-CCCGAGCGTAGTAG-TTATA-TCTCGCTCTGG-AAGGCCCTGGCGGTGCCCTGCCGTTAAA-CCCCCAACTTCTGAAAATT

>Diaporthe_caliensis_STMA_22040

ACAAGGTCTCCGTTGGTGAACCAGCGGAGGGATCATTGCTGGAACGCG-CTTC-GGCGCA-CCCAGAAACCCTTTGTGAACTTATACCTAT--TGTTGCCTCGGCG-TAGGCCGGCCTCT--TC----------ACTGAGGCC-----CCCTG---GAGACAGGGAGCA-GCCCGCCGGCGGCCAA-CCAAACTCTTGTTTCTACAGTGAATCTCTGAGT--AAAAAACATAAATGAATCAAAACTTTCAACAACGGATCTCTTGGTTCTGGCATCGATGAAGAACGCAGCGAAATGCGATAAGTAATGTGAATTGCAGAATTCAGTGAATCATCGAATCTTTGAACGCACATTGCGCCCTCTGGTATTCCGGAGGGCATGCCTGTTCGAGCGTCATTTCAACCCTCAAGCCTGGCTTGG-TGATGGGGCACTGCTTTCGTC-----CAGAAAGCAGGCCCTGAAATCTAGTGGCGAGCT-CGCCAGGAC-CCCGAGCGTAGTAG-TTATA-TCTCGCTCTGG-AAGGCCCTGGCGGTGCCCTGCCGTTAAA-CCCCCAACTTCTGAAAATT

**Alignment of the *cal* sequences used in the phylogenetic study**

>Diaporthe_acaciarum_CBS_138862

?????????????????????????????????????????????????????????????????????????????????????????????????????????????????????????????????????????????????????????????????????????????????????????????????????????????????????????????????????????????????????????????????????????????????????????????????????????????????????????????????????????????????????????????????????????????????????????????????????????????????????????????????????????????????????????????????

>Diaporthe_acericola_MFLUCC_17_0956

NNNNNNNNNNNNNNNNNNNNNNNNNNNNNNNNNNNNNCCTCGAGC-----TCCCCCGCCAT--GTTCTGCCTTTGCGCATG--ATGCTAACGGACCGTTTTCGGCCTGCAGGATAAGGATGGCGATGGTTAGTGCGGTCACCGCT----TCCTTCCCTCT-TTCTCAGCTACCCACGCGTCATACTCGATCCGCCGCGACGGTCTGCGCG-TGCAGTATACTC-TGAGCGAGCG-AACCTCAT----------ATCGATCACGAGAAATATGCTAAGAC--GGC-GTGTAGGACAAATCACCACCAAGGAGCTCGGCACAGTCATGCGGTCCCTTGGTCAAAACCCTTCCGAGTCCGAGCTGCAGGACATGATCAACGAGGTCGACGCCGACAACAATGGCACCATTGACTTCCCTGGTAAGTCTACATGT-CCACCCA-CTGAATATT

>Diaporthe_alangii_CFCC_52556

NNNNNNNNNNNNNNNNNNNNNNNNNNNNNNNNNNNNNNNNNNNNNNNNNNNNNNCCGCCAC--GCTCTGCTGTT---CATG--ATGCTAACGGACCGTTTTCGGCCCGCAGGATAAGGATGGCGATGGTTAGTGTGGTCACCACC----TTCTTCCCTCT-TCCTCAGCTACGCACGCGTCATGCTCGATCCGCCGCGACGGCCTGCGCG-TGCA-TATAATC-CAAGCGACCG-ATCATCG-----------ATCCATCACCAGTACCATGCTAAGAC--GGC-GTGCAGGACAAATCACCACCAAGGAGCTAGGCACGGTCATGCGGTCCCTGGGTCAAAACCCCTCCGAGTCTGAGCTGCAGGACATGATTAACGAGGTCGATGCCGACAACAATGGCACCATTGACTTCCCTGGTAAGCCAAGATGC-TCGCCCG-CCGAGTGTT

>Diaporthe_ambigua_CBS_114015

CTTCTCCCTCTTTGTAAGTTATCTTC---TGTA---GCCTTGAGC----CTCTCCCGCCGT--GCTTTGCCGTTGCGCATG--ATGCTAACGGCCCGTTTTCGGCCTGCAGGATAAGGATGGCGATGGTTAGTGCAGTCACCACG----TTCTTCCCTCT-TTCCCAGCTACGCACGCGTCACACTCGATCCGCCGCGACGGTCTGCGCG-TGCA-TATACTC-CAGACGACCG-ACCATCAC----------ATCCATCTCGAGTGCCATGCTAAGAC--GGC-GTGTAGGACAAATCACCACCAAGGAGCTCGGCACGGTCATGCGTTCCCTGGGCCAGAACCCCTCCGAGTCCGAGCTGCAGGATATGATTAACGAGGTCGACGCCGACAACAATGGCACCATTGACTTCCCTGGTAAGTCCAGATGC-TCGCGCA-CTGAAAATT

>Diaporthe_amygdali_CBS_126679

CTTCTCCCTCTTTGTAAGTTATTTTC---AGGA------ATGATC------CCGCAGCCCTCCTCGCCACTGTCGCGCATG--ATGCTAACGGACCGTTCTCGGCCTCCAGGATAAGGATGGCGATGGTTAGTGCAGCTGCCTCT----TCTTCAC-----CTCCCAGCTTCGTACGCGTCACGATCGACCCGCCGCGACGGCTTGCGCG-TGCA-CATTTTC-CAACCAAGC--ACCATAAC----------ATCTACTATGAGCTCGATGCTAAGAT--GAC-GTGTAGGACAAATCACCACAAAGGAGCTCGGCACGGTCATGCGATCTCTGGGTCAGAACCCGTCCGAGTCTGAGCTGCAGGATATGATCAACGAGGTCGACGCCGACAACAATGGAACCATCGACTTCCCTGGTACGTCCAGATGC-TCGCTTGTTTGGAAGGN

>Diaporthe_angelicae_CBS_111592

CTTCTCCCTCTTTGTAAGTTATTCTC---ACCA---GCCTTGAGC----TCCCCCCGCCAT--GTTCTGCTGTTGCGCATG--ATGCTAACGGACCGTTTTCGGCCTGCAGGATAAGGATGGCGATGGTTAGTGCGGTCACCGCT----TCCTTCCCTCT-CTCTCAGCTACCCACGCGTCATACTCGATCCGCCGCGACGGTCTGCGCG-TGCAGTACACTCTTGAGCGAGCG-AGCCTCAT----------ATCGATCACGAGAAATATGCTAAGAC--GGC-GTGTAGGACAAATCACCACCAAGGAGCTCGGCACAGTCATGCGGTCCCTTGGTCAAAACCCTTCCGAGTCCGAGCTGCAGGACATGATCAACGAGGTCGACGCCGACAACAATGGCACCATTGACTTCCCTGGTAAGTCTAGATGT-CCACCCA-CTGGATATT

>Diaporthe_arctii_CBS_136_25

NNNNNNNNNNNNNNNNNNNNNNNNNC---ACCA---GCCTTGAGC----TCCCCCCGCCAT--GTTCTGCCTTTGCGCATG--ATGCTAACGGACCGTTTTCGGCCTGCAGGATAAGGATGGCGATGGTTAGTGCGGTCACCGCT----TCCTCCCCTCT-CTCTCAGCTACCCACGCGTCATACTCGATCCGCCGCGACGGTCTGCGCG-TGCAGTACACTCTTGAGCGAGCG-AGCCTCAT----------ATCGATCACGAGAAATATGCTAAGAC--GGC-GTGTAGGACAAATCACCACCAAGGAGCTCGGCACAGTCATGCGGTCCCTTGGTCAAAACCCTTCCGAGTCCGAGCTGCAGGACATGATCAACGAGGTCGACGCCGACAACAATGGCACCATTGACTTCCCTGGTAAGTCTAAATGT-CCACCCA-CTGGATATT

>Diaporthe_arezzoensis_MFLU_19_2880

?????????????????????????????????????????????????????????????????????????????????????????????????????????????????????????????????????????????????????????????????????????????????????????????????????????????????????????????????????????????????????????????????????????????????????????????????????????????????????????????????????????????????????????????????????????????????????????????????????????????????????????????????????????????????????????????????

>Diaporthe_batatas_CBS_122_21

CTTCTCCCTCTTTGTAAGTTATCTTC------A---GCCTTGAGC-----TTCCCCGCCGT--TCTCTTCCGTTGCGCACG--ATGCTAACCGACCGTTTTCGGCATGTAGGATAAGGATGGCGATGGTTAGTGCGGTCACCGCT----TCCTCCCCTCT-TTCTCAGCTACGCACGCGTCATACTCGATCCACCGCGACGGTCTGCGCG-TGCAGTATACTG-CGAGCGACCG-ATCACCGA----------ATCTATCACGAGAAATCTACTAAGACGGCCCGGTGTAGGACAAATCACCACCAAGGAGCTCGGCACAGTCATGCGGTCCCTTGGTCAGAACCCTTCCGAGTCCGAGCTGCAGGACATGATCAACGAGGTCGACGCCGACAACAATGGCACCATTGACTTCCCTGGTAAGTCTGGATTC-CCACACA-CTGGGTATN

>Diaporthe_beilharziae_BRIP_54792

?????????????????????????????????????????????????????????????????????????????????????????????????????????????????????????????????????????????????????????????????????????????????????????????????????????????????????????????????????????????????????????????????????????????????????????????????????????????????????????????????????????????????????????????????????????????????????????????????????????????????????????????????????????????????????????????????

>Diaporthe_biguttulata_ICMP20657

?????????????????????????????????????????????????????????????????????????????????????????????????????????????????????????????????????????????????????????????????????????????????????????????????????????????????????????????????????????????????????????????????????????????????????????????????????????????????????????????????????????????????????????????????????????????????????????????????????????????????????????????????????????????????????????????????

>Diaporthe_breyniae

CTTCTCCCTCTTTGTAAGTTATATCC---AGGA---GCCTTGAGC-----TTCCCCGCCGT--TCTCTGCTGTTGCGCCTG--ATGCTAACGGACCGTTTTCGGCCTACAGGATAAGGATGGCGATGGTTAGTGCGGTCACCGCT----TCCTCCCCTCT-TTCTCAGCTACGCACGCGTCATACTCGATCCGCCGCGACGGTCTGCGCG-TGCAGTCTACTC-CGAGCGACCG-ACCATGAA----------ATCTATCACGAGTAGTATGCTAAGGCT-GGC-GTGTAGGACAAATCACCACCAAGGAGCTCGGCACAGTCATGCGGTCCCTTGGCCAGAACCCTTCCGAGTCCGAGCTGCAGGATATGATCAACGAGGTCGACGCCGACAACAACGGCACCATTGACTTCCCTGGTAAGACTATCTTC-GCGTACA-CTGGATACN

>Diaporthe_camporesii_JZB320143

?????????????????????????????????????????????????????????????????????????????????????????????????????????????????????????????????????????????????????????????????????????????????????????????????????????????????????????????????????????????????????????????????????????????????????????????????????????????????????????????????????????????????????????????????????????????????????????????????????????????????????????????????????????????????????????????????

>Diaporthe_caryae_CFCC_52563

NNNNNNNNNNNNNNNNNNNNNNNNNNNNNNNNNNNNNNNNNNNNNNNNNNNNNNCCGCCAC--ACTGTGCTTTTGCGCATG--ATGCTAACGGACCGTTTTCGGCCTGCAGGATAAGGATGGCGATGGTTAGTGTGGTCACCGCC----TTCTTCCCTCT-TTCTCAGCTACGCACGCGTCATATTCGATCCGCCGCGACGGTCTGCGCG-CGCAGCATGCTC-CGAGCGACCG-ATCATCAC----------GTCTATCACGAGTAACATGCTAAGTC--GGC-GTGTAGGACAAATTACCACCAAGGAGCTCGGCACAGTCATGCGGTCCCTTGGTCAAAACCCTTCCGAGTCCGAGCTGCAGGACATGATCAACGAGGTCGACGCCGACAACAATGGCACCATTGACTTCCCTGGTAAGTCTGGATGC-TTATCCC-CTAGATGTT

>Diaporthe_celtidis_NCYU_19_0357

?????????????????????????????????????????????????????????????????????????????????????????????????????????????????????????????????????????????????????????????????????????????????????????????????????????????????????????????????????????????????????????????????????????????????????????????????????????????????????????????????????????????????????????????????????????????????????????????????????????????????????????????????????????????????????????????????

>Diaporthe_cerradensis_CMRP4331

NNNNNNNNNNNNNNNNNNNNNNNNNNNNNNNNNNNNNNNNTTGAC-----TTCTCCGCCGT--CCGCTGCTGTTGCGCATG--ATGCTAACGGCCCGTTTTCGGCCTGCAGGATAAGGATGGCGATGGTTAGTGTTGTCACCACT----TTCTCTCCTCT-TTCTCAGCTACGCACGCGTCGGGCTCGACCCGCCGCGACGGCCTGCGCG-TGCA-TATACCC-TAAGCGACCG-AACGTCAC----------A-ATATGACGAGTATCAAGCTAAGGC--GGC-GTGTAGGACAAATCACCACCAAGGAGCTCGGCACTGTCATGCGGTCCCTGGGTCAAAACCCCTCCGAGTCCGAGCTGCAGGACATGATCAACGAGGTCGACGCCGACAACAACGGCACCATTGACTTCCCTGGTAAGTTCAAATGC-TCGCCTG-CTGAATATA

>Diaporthe_chimonanthi

?????????????????????????????????????????????????????????????????????????????????????????????????????????????????????????????????????????????????????????????????????????????????????????????????????????????????????????????????????????????????????????????????????????????????????????????????????????????????????????????????????????????????????????????????????????????????????????????????????????????????????????????????????????????????????????????????

>Diaporthe_chinensis_MFLUCC_19_0101

NNNNNNNNNNNNNNNNNNNNNNNNNNNNNNNNNNNNNNNNNNNNNNNNNNNNNCCCGCCCT--GCTCTGCTGTCGCGCATG--ATGCTAACGGACCGTTTTCGGCTCGCAGGATAAGGATGGCGATGGTTAGTGCAGCCACCACT----TCTCTCTCTCTTTTCCCAACTACGCACGCGTCACTCTTGATCCGCTACGACGGTCTACGCG-TGCA-TATACTC-CAACCGACCG-ATCATCAC----------ATCCATCACGAGTACCATGCTAAGAC--GGC-GTGTAGGACAAATCACCACCAAGGAGCTCGGCACGGTCATGCGGTCCCTGGGTCAAAACCCCTCCGAGTCCGAGCTGCAGGATATGATCAATGAGGTCGACGCCGACAACAATGGCACCATTGACTTCCCTGGTAAGTCTAGATGC-TCGTATA-CTGAATGTT

>Diaporthe_chromolaenae_MFLUCC_17_1422

?????????????????????????????????????????????????????????????????????????????????????????????????????????????????????????????????????????????????????????????????????????????????????????????????????????????????????????????????????????????????????????????????????????????????????????????????????????????????????????????????????????????????????????????????????????????????????????????????????????????????????????????????????????????????????????????????

>Diaporthe_cichorii_MFLUCC_17_1023

NNNNNNNNNNNNNNNNNNNNNNNNNNNNNNNNNNNNNCCTTGAGCCCCTCCCCCCCGCCAT--GTTCTGCCTTTGCGCTTG--ATGCTAACGGACCGTTTTCGGCCTGCAGGATAAGGATGGCGATGGTTAGTGCGGTCACCGCT----TCCTTCCCTCT-TTCTCAGCTACCCACGCGTCATACTCGATCCGCCGCGACGGTCTGCGCG-TGCAGTACACTCTTGAGCGAGCA-AACCTCAT----------ATCGATCACGAGAAATATGCTAAGAC--GGC-GTGTAGGACAAATCACCACCAAGGAGCTCGGCACAGTCATGCGGTCCCTTGGTCAAAACCCTTCCGAGTCCGAGCTGCAGGACATGATCAACGAGGTCGACGCCGACAACAATGGCACCATTGACTTCCCTGGTAAGTCTAGATGT-CCACCCA-CTGGATATT

>Diaporthe_cinnamomi_CFCC_52569

?????????????????????????????????????????????????????????????????????????????????????????????????????????????????????????????????????????????????????????????????????????????????????????????????????????????????????????????????????????????????????????????????????????????????????????????????????????????????????????????????????????????????????????????????????????????????????????????????????????????????????????????????????????????????????????????????

>Diaporthe_citriasiana_CBS_134240

CTTCTCCCCCTTCGTAAGTCACCATC---AGTA---GCCTCAAGC-----CTCCCCGCCCT--GCTCTGCTGTCGCGCATG--ATGCTAACGGACCGTTTTCGGATCGCAGGATAAGGATGGCGATGGTTAGTGCAACCACCTCT----TTCTTCTCTCTTTTTCCAACTACGCACGCGTCACGCTTGATCCGCTGCGACGGTCTGCGCG-TGTA-GATACTC-CAAGCGACCG-AACATCGC----------ATCCATCACGAGTATCATACTAAGAC--GGC-GCGTAGGACAAATCACCACCAAGGAACTCGGCACGGTCATGCGGTCCCTGGGTCAAAACCCCTCCGAGTCCGAGCTGCAGGATATGATCAATGAGGTCGACGCCGACAACAATGGCACCATTGACTTCCCTGGTAAGTCCAGATGC-TCGCTTA-CTGAACGGT

>Diaporthe_compacta_LC3083

?????????????????????????????????????????????????????????????????????????????????????????????????????????????????????????????????????????????????????????????????????????????????????????????????????????????????????????????????????????????????????????????????????????????????????????????????????????????????????????????????????????????????????????????????????????????????????????????????????????????????????????????????????????????????????????????????

>Diaporthe_convolvuli_CBS_124654

CTTCTCCCTCTTTGTAAGTTATCTCCAGGAGGAGGAGCCTTGAGC-----TTCCCCGCCAT--TCTCTACTGTTGCGCATG--ATGCTAACGGACCGTTTTCGACCTGCAGGATAAGGATGGCGATGGTTAGTGCGGTCACCGCT----TCCTCACCCCT-TTCTCAGCTACGCACGCGTCGTACTCGATCCGCCGCGACGGTCTGCGCG-TGCAGTCTACTC-CGAGCGACCG-ATCATCAA----------ATCTATCAC----AGTATGCTAAGGCT-GGC-GTGTAGGACAAATCACCACCAAGGAGCTCGGCACAGTCATGCGGTCGCTTGGTCAAAACCCTTCCGAGTCCGAGCTGCAGGACATGATCAACGAGGTCGACGCTGACAACAACGGCACCATTGACTTCCCTGGTAAGTCTCTCAAC-TGTCACA-CTGGAGATN

>Diaporthe_cucurbitae_DAOM_42078

?????????????????????????????????????????????????????????????????????????????????????????????????????????????????????????????????????????????????????????????????????????????????????????????????????????????????????????????????????????????????????????????????????????????????????????????????????????????????????????????????????????????????????????????????????????????????????????????????????????????????????????????????????????????????????????????????

>Diaporthe_cuppatea_CBS_117499

NNNNNNNNNNNNNNNNNNNNNNNNNNNNNNNNNNNNNNNNNNNNNNNNNNNNNNNNGCCAT--GTTCTGCCTTTGCGCATG--ATGCTAACGGACCGTTTTCGGCCTGCAGGATAAGGATGGCGATGGTTAGTGCGGTCACCGCT----TCCTTCCCTCT-TTCTCAGCTACCCACGCGTCATATTCGATCCGCCGCGACGGTCTGCGCG-TGCAGTACACTC-TGAGCGAGCG-AACCTCAC----------ATCGATCACGAGAAATATGCTAAGAC--GGC-GTGTAGGACAAATCACCACCAAGGAGCTCGGCACAGTCATGCGGTCCCTGGGTCAAAACCCTTCCGAGTCCGAGCTGCAGGACATGATCAACGAGGTCGATGCCGACAACAATGGCACCATTGACTTCCCTGGTAAGTCTAGATGC-CCACCCG-CTGAATATT

>Diaporthe_discoidispora_ICMP20662

?????????????????????????????????????????????????????????????????????????????????????????????????????????????????????????????????????????????????????????????????????????????????????????????????????????????????????????????????????????????????????????????????????????????????????????????????????????????????????????????????????????????????????????????????????????????????????????????????????????????????????????????????????????????????????????????????

>Diaporthe_durionigena_VTCC_930005

?????????????????????????????????????????????????????????????????????????????????????????????????????????????????????????????????????????????????????????????????????????????????????????????????????????????????????????????????????????????????????????????????????????????????????????????????????????????????????????????????????????????????????????????????????????????????????????????????????????????????????????????????????????????????????????????????

>Diaporthe_eres_CBS_138594

CTTTTCCCTCTTTGTAAGTCATTTCCAGCCGGCA--GACATGAGC------TCCCCGCCCT--CCTCTGCTGGTGCGCATG--ATGCTAACGGACCGTTTTCGGCTTGTAGGATAAGGATGGCGATGGTTAGTGCGGCCGCCTCT----TTGCCCC-----CTCCCACCTACGCACGCGTCATGTTCGATCCGCCGCGACAGCCTGCGCG-TGCA-TAATTTC-CAACCAAGCG-ATTATCAC----------ATCTATCACGAGTATCATGCTGAGATATGGC-GTGTAGGGCAAATCACCACCAAGGAGCTCGGCACGGTCATGCGATCCCTGGGTCAGAACCCGTCCGAGTCTGAGCTGCAAGATATGATTAACGAGGTCGACGCCGACAACAATGGCACCATTGACTTCCCTGGTACGTCCAGATGC-TCGCGCT-CTG?????N

>Diaporthe_fici_septicae_MFLU_18_2588

?????????????????????????????????????????????????????????????????????????????????????????????????????????????????????????????????????????????????????????????????????????????????????????????????????????????????????????????????????????????????????????????????????????????????????????????????????????????????????????????????????????????????????????????????????????????????????????????????????????????????????????????????????????????????????????????????

>Diaporthe_fructicola_MAFF_246408

CTTCTCCCTCTTTGTGAGTTATCTCC---AGGA---GCCTTGAGC-----TTCCCCGCCGT--TCTCTGCCGTTGCGCATG--ATGCTAACGGACCGTTTTCGGCCTGCAGGATAAGGATGGCGATGGTTAGTGCGGTCACCGCT----TCCTCCCCTCT-TTGCCGGCTACGCACGCGTCATGCTCGATCCGCCGCGACGGTCTGCGCG-TGCAGTCTACTC-CGAGCGACCG-ATCATCAA----------ATCCATCACGAGTGGTATGCTAAGGCT-GGC-ATGTAGGACAAATCACCACCAAGGAGCTCGGCACAGTCATGCGGTCGCTTGGTCAAAACCCTTCCGAGTCCGAGCTGCAGGACATGATCAACGAGGTCGACGCCGACAACAACGGCACCATTGACTTCCCTGGTAAGTCTGAACTG-T--TACA-CTGGGGATN

>Diaporthe_ganjae_CBS_180_91

CTTCTCCCTCTTTGTAAGTTATATCC---AGCA---GCCTTGGGC-----TCCCCCGCCGT--CCCCTGCTGTTGCGCATG--ATGCTAACGGACCGTTTTCGGCCTGCAGGATAAGGATGGCGATGGTTAGTGTGGTCACCGCT----TTCTTCCCTCC-TCCTCAGCCACGCACGCGTCATGCTCGATCCGCCGCGACGGTCTGCGCG-TGCATTATGCCC-AGAGCGATCGAATCATCAC----------ACCCATGACGAGCACCATGCTAAGAC--GGC-GTGCAGGACAAATCACCACCAAGGAGCTCGGCACGGTCATGCGGTCCCTGGGCCAAAACCCCTCCGAGTCCGAGCTGCAGGACATGATCAACGAGGTCGACGCCGACAACAACGGCACCATTGACTTCCCTGGTAGGTCCACATGTCCCTCCCA-CTGGATGTT

>Diaporthe_goulteri_BRIP_55657a

?????????????????????????????????????????????????????????????????????????????????????????????????????????????????????????????????????????????????????????????????????????????????????????????????????????????????????????????????????????????????????????????????????????????????????????????????????????????????????????????????????????????????????????????????????????????????????????????????????????????????????????????????????????????????????????????????

>Diaporthe_guangdongensis_ZHKUCC20_0014

CTTCTCCCTCTTTGTAAGTCATCTTC---AGGA---GCCTTGAGC-----TTCCCCGCCGT--TCTCTGCCGTTGCGCATG--ATGCTAACGGACCGTTTTCGGCCTGCAGGATAAGGATGGCGATGGTTAGTGCGGTCACCGCT----TCCTCCCCTCT-TCCTCAGCTACGCACGCGTCACACTCGATCCGCCGCGACGGTCTGCGCG-TGCAGTATATTC-CGAGCGACCG-ATCATCAC----------ATCCATCACGAGTAGTATGCTAAGGCG-GGC-GTGTAGGACAAATCACCACCAAGGAGCTCGGCACAGTCATGCGGTCCCTTGGTCAAAACCCTTCCGAGTCCGAGCTGCAGGACATGATCAACGAGGTCGACGCCGACAACAATGGCACCATTGACTTCCCTGGTGAGTCTA-ATTC-TGGCACA-CTGGATATN

>Diaporthe_gulyae_BRIP_54025

?????????????????????????????????????????????????????????????????????????????????????????????????????????????????????????????????????????????????????????????????????????????????????????????????????????????????????????????????????????????????????????????????????????????????????????????????????????????????????????????????????????????????????????????????????????????????????????????????????????????????????????????????????????????????????????????????

>Diaporthe_guttulata_CGMCC_3_20100

NNNNNNNNNNNNNNNNNNNNNNNNNNNNNNNNNNNNNNNNNNNNNNNNNNNNNCCCGCCGT--GTTCTGCCTTTGCGCATG--ATGCTAACGGACCGTTTTCGGCCTGCAGGATAAGGATGGCGATGGTTAGTGCGGTCACCGCT----TCCTCCCCTCT-CTCTCAGCTACCCACGCGTCATACTCGATCCGCCGCGACGGTCTGCGCG-TGCAGTACACTCTTGAGCGAGCG-AACCTCAT----------ATCGATCACGAGAAACATGCTAAGAC--GGC-GTGTAGGACAAATCACCACCAAGGAGCTCGGCACGGTCATGCGATCCCTGGGTCAGAACCCGTCCGAGTCTGAGCTGCAAGATATGATTAACGAGGTCGACGCCGACAACAATGGCACCATTGACTTCCCTGGTAAGTCTAGATGT-CCACCCA-CTGNNNNNN

>Diaporthe_helianthi_CBS_592_81

????????????????????????????????????????????????????????GCCGT--TCTCTGCTGTTGCGCCTGGTGTGCTAATGGACCGTTTTCTGCCTGCAGGATAAGGACGGCGATGGTTAGTGCGGTCACCGCT----TCCCGCCCTCT-CTCTCAGCTGCGCACGCGTCACAATCGATCCGCCGCAACGGTCTGCGCT-TGCAATGTACCC-CGAGCGACCG-ATCATTAAATCTATCACGATCTATCACGAGTCGTATGCTAAGGCG-GGC-GTGTAGGACAAATCACCACCAAGGAGCTGGGCACAGTCATGCGGTCCCTTGGTCAAAACCCTTCCGAGTCCGAGCTGCAGGACATGATCAACGAGGTCGACGCCGACAACAATGGCACCATTGACTTCCCTGGTACGTTTAGATTC-TCGTACA-CTGCATATN

>Diaporthe_heterostemmatis_SAUCC194_85

?????????????????GGCAATCTC---AGGA---GCCTTGAGC-----TT-CCCGCCGT--TCTCTGCCGTTGCGCATG--ATGCTAACGGACCGTTTTCGGCCTGCAGGATAAGGATGGCGATGGTTAGTGCGGTCACCGCT----TCCTCCCCTCT-TTCCCGGCTACGCACGCGTCATGCTCGATCCGCCGCGACGGTCTGCGCG-TGCAGTCTACTC-CGAGCGACCG-ATCATCAA----------ATCTATCACGAGTGGTATGCTAAGGCT-GGC-ATGTAGGACAAATCACCACCAAGGAGCTCGGCACAGTCATGCGGTCGCTTGGTCAAAACCCTTCCGAGTCCGAGCTGCAGGACATGATCAACGAGGTCGACGCCGACAACAACGGCACCATTGACTTCCCTGGTAAGTCTCAACTG-T--CACA-CTGGAGATN

>Diaporthe_hordei_CBS_481_92

CTTCTCCCTCTTTGTAAGTTATCTTC---AAGA---GCCTTAAGC-----TTCCCCGCCGTTCTCTCTGCCGTTGCGCATG--GTGCTAACGGACCGTTTTCGGCCTGCAGGATAAGGATGGTGATGGTTAGTGCGGTCACCGCTTCCCTCCCTCCCTCT-TTCTCAGCTTTGAACGCGTCATACTCGATCCGCCGCGACGGTCTGCGCT-TGCAATATACTC-CGAGCGACCA-ATCTTCAA----------ATCTATCACGAGTAGTATGCTAAGGCG-GGC-GTGTAGGACAAATCACCACCAAGGAGCTCGGCACAGTCATGCGGTCCCTTGGTCAAAACCCTTCCGAGTCCGAGCTGCAGGACATGATCAACGAGGTCGACGCCGACAACAATGGCACCATTGACTTCCCTGGTAAGTCTAGATTC-TCTCACA-CTGAATATN

>Diaporthe_hubeiensis_JZB320123

CTTTTCCCTCTTTGTAAGTCATTTCC---AGCCGGCAGACATGAG-----CTCCCCGCCCT--CCTCTGCTGTTGTCCATA--ATGCTAACGGACCGTTTTCGGCCCGCAGGATAAGGATGGCGATGGTTAGTGTGGTCACCACC----TTCTTCCCTCT-TCCTCAGCTACGCACGCGTCATGCTCGAACCGCCGCGACGGCCTGCGCT-TGCA-TATAATC-CAAGCGACCG-ATCATCG-----------ATCCATCACCAGTACCATGCTAAGACG-GGC-GTGCAGGACAAATCACCACCAAGGAGCTCGGCACGGTCATGCGGTCCCTGGGTCAAAACCCCTCCGAGTCTGAGCTGCAGGACATGATTAACGAGGTCGATGCCGACAACAATGGCACCATTGACTTCCCTGGTAAGCCAAGATGC-TCGCCCG-CCGAGTGTT

>Diaporthe_infecunda_CBS_133812

CTTCTCCCTCTTTGTAAGTTATCTTC---GGAG---GCCTGGAGC-----TTCCCCGCCGT--CCTGTGCTTTTGCGCATG--ATGCTAACGGACCGTTTTCGGCCTGCAGGATAAGGATGGCGATGGTTAGTGTGGTCACCGCT----TTCTTCCCTCT-TGCTCAGCTACGCACGCGTCATACTCGATCCGCCGCGACGGTCTGCGCG-CGCAGCATGCTC-CGAGCGACCG-ATCATCAC----------GTCTATCACGAGTAACATGCTAAGTC--GGC-GTGTAGGACAAATCACCACCAAGGAGCTCGGCACAGTCATGCGGTCCCTTGGTCAAAACCCTTCCGAGTCCGAGCTGCAGGACATGATCAACGAGGTCGACGCCGACAACAATGGCACCATTGACTTCCCTGGTAAGTCTGGATGC-TCATCCC-CTAGACGTT

>Diaporthe_infertilis_CBS_230_52

CTTCTCCCTCTTTGTAAGTTATCTTC---AGGA---GCCTTGAGC-----TTCCCCGCCGT--TCTTTGCCGTTGCGCATG--ATGCTAACTGACCGTTTTCGGCCTACAGGATAAGGATGGCGATGGTTAGTGCGGTCACCGCT----TCCTCCCCTCT-TTCTCAGCTACGCACGCGTCATACTCGATCCGCCGCGACGGTCTGCGCG-TGCAGTATATTC-CGAGCGACCG-TTCATCAA----------ATCTATCACGAGTAGTATGCTAAGGCG-GGC-GTGCAGGACAAATCACCACCAAGGAGCTCGGCACAGTCATGCGGTCCCTGGGCCAGAACCCTTCCGAGTCCGAGCTGCAGGACATGATCAACGAGGTCGACGCCGACAACAACGGCACCATTGACTTCCCTGGTGAGTCCAGATTC-TCGCACA-TTGGTTTTN

>Diaporthe_kochmanii_BRIP_54033

?????????????????????????????????????????????????????????????????????????????????????????????????????????????????????????????????????????????????????????????????????????????????????????????????????????????????????????????????????????????????????????????????????????????????????????????????????????????????????????????????????????????????????????????????????????????????????????????????????????????????????????????????????????????????????????????????

>Diaporthe_kongii_BRIP_54031

?????????????????????????????????????????????????????????????????????????????????????????????????????????????????????????????????????????????????????????????????????????????????????????????????????????????????????????????????????????????????????????????????????????????????????????????????????????????????????????????????????????????????????????????????????????????????????????????????????????????????????????????????????????????????????????????????

>Diaporthe_leucospermi_CBS_111980

NNNNNNNNTCTTTGTAAGTTATCTTC---GGAG---GCCTTGAGC-----TTCCCCGCCAT--CCTGTGCTTTTGCGCATG--ATGCTAACGGACCGTTTTCGGCCTGCAGGATAAGGATGGCGATGGTTAGTGTGGTCACCGCT----TTCTCCCCTCT-TTCTCAGCTACGCACGCGTCATACTCGATCCGCCGCGACGGTCTGCGCG-TGCAGCATGCTC-CGAGCGACCG-ATCATCAC----------GTCTATCACGAGTAACATGCTAAGTC--GGC-GTGTAGGACAAATCACCACCAAGGAGCTCGGCACAGTCATGCGGTCCCTTGGTCAAAACCCTTCCGAGTCCGAGCTGCAGGACATGATCAACGAGGTCGACGCCGACAACAATGGCACCATTGACTTCCCTGGTAAGTCTGGATGC-TCATCCC-CTAGACATT

>Diaporthe_longicolla_FAU_599

CTTCTCCCTCTTTGTAAGTTATATCC---AGGA---GCCTCGAGC-----TTCCCCGCCGT--TCTCTGCTGTTGCGCCTG--ATGCTAACGGACCGTTTTCGGCCTGCAGGATAAGGATGGCGATGGTTAGTGCGGTCACCGCT----TCCTCCCCTCT-TTCTCAGCTACGCACGCGTCATACTCGATCCGCCGCGACGGTCTGCGCG-TGCAG-CTACTC-CGACCGACCG-ACCATCAA----------ATCTATCACGAGTAGTATGCTAAGGCT-GGC-GTGTAGGACAAATCACCACCAAGGAGCTCGGCACAGTCATGCGGTCCCTTGGTCAAAACCCTTCCGAGTCCGAGCTGCAGGACATGATCAACGAGGTCGACGCCGACAACAACGGCACCATTGACTTCCCTGGTGAGTCTAGATCC-TCGTACA-CTGGATATN

>Diaporthe_longispora_CBS_194_36

NNNNNNNNNNNNNNNNNNNNNNNNNNNNNGGCA---GCCTTGAGC-----CTCCCCGCCGT--CCGCTGCTGTCGCGCATG--ATGCTAACGGACCGTTTTCGGCCTGCAGGATAAGGATGGCGATGGTTAGTGTGGTCACAACT----TTCTTCCCTCT-TTACTAGCTACGCATGCGTCACACTCGATCCCCCGCGACGGTCTGCGCGTTGCA-TATACTC-CGAGCGACCG-ATCGTCAC----------ATCTGTCA-GGATACCATGCTAAGAC--GGC-GTGTAGGACAAATCACCACCAAGGAGCTCGGCACGGTCATGCGTTCCCTGGGTCAAAACCCCTCCGAGTCCGAGCTGCAGGATATGATCAACGAGGTCGACGCCGACAACAATGGCACCATTGACTTCCCTGGTAAGTCTAGATGC-TCGCCCA-CCGGATGTT

>Diaporthe_lusitanicae_CBS_123212

CTTCTCCCTCTTTGTAAGTTATTCTC---ACCT---GCCCTGAGC----TCCCCCCGCCAT--GTTCTGCCTTTGCGCATG--ATGCTAACGGACCGTTTTCGGCCTGCAGGATAAGGATGGCGATGGTTAGTGCGGTCACCGCT----TCCTTCCCTCT-TTCTCAGCTACCCACGCGTCATACTCGATCCGCCTCGACGGTCTGCGCG-TGCAGTACACTC-TGAGCGAGCG-AACCTCAT----------ATCGACCACGAGAAACATGCTAAGAC--GGC-GTGTAGGACAAATCACCACCAAGGAGCTCGGCACAGTTATGCGGTCCCTTGGTCAAAACCCTTCCGAGTCCGAGCTGCAGGACATGATCAACGAGGTCGACGCCGACAACAATGGCACCATTGACTTCCCTGGTAAGTCTAGATGT-CCACCCG-CTGAATATT

>Diaporthe_machili_SAUCC194_111

NNNNNNNNNNNNNNNNNNNNTGAATC---TCGA---GGCTTGAGC------TTCCCGCCAC--ACTGTGCTTTTGCGCATG--ATGCTAACGGACCGTTTTCGGCCTGCAGGATAAGGATGGCGATGGTTAGTGTGGTCACCGCT----TTCTTCCCTCT-TTCTCAGCTACGCACGCGTCATACTCGATCCGCCGCGACGGTCTGCGCG-CGCAGCATGCTC-CGAGCGACCG-ATCATCAC----------GTCTATCACGAGTAACATGCTAAGTC--GGC-GTGTAGGACAAATTACCACCAAGGAGCTCGGCACAGTCATGCGGTCCCTTGGTCAAAACCCTTCCGAGTCCGAGCTGCAGGACATGATCAACGAGGTCGACGCCGACAACAATGGCACCATTGACTTCCCTGGTAAGCCTGGATGC-TTATCCC-CTAGACGTC

>Diaporthe_manihotia_CBS_505_76

CTTCTCCCTCTTTGTAAGTCATATCC---AGCA---GCCTCGGGC-----TCCCCTGTCGT--CCTCTGCTGTTGCGCATG--ATGCTAACGGACCGTTCTCGGCCTGCAGGATAAGGATGGCGATGGTTAGTGTGGTCACCGCT----TTCTTCCCTCC-TCCTAAGCCACGCACGCGTCATGCTCGATCCGCCGCGACGGTCTGCGCG-TGCATTATGCCC-CGAGCGACCGAATCATCAC----------ATCTATCACGAGTACCATGCTAAGAC--GGC-GTGCAGGACAAATCACCACCAAGGAGCTCGGCACGGTCATGCGGTCCCTGGGCCAAAACCCCTCCGAGTCCGAGCTGCAGGACATGATCAACGAGGTCGACGCCGACAACAACGGCACCATTGACTTCCCTGGTAGGTTCACATGT-CCACCCA-CTGGATGTT

>Diaporthe_mayteni_CBS_133185

CTTCTCCCTCTTTGTAAGCTGTCTTC---GCTA---GCCTTGAGC-----CACCCCGCCGT--CCGCTGTTGTTGCGCCTC--ATGCTAACGGACCGTTTTCGGCCTGCAGGATAAGGATGGCGATGGTTAGTGTGGTCACCACT----TTGTT-CCTCT-TTCCCAGCCACGCACGCGTCACACACGATCCGCCGCGA-GGTCTGCGCG-TTCA-TAAACCC-CAGGCGACCG-ATCACAAT----------ATCTATCACAAGTACCATGCTAAGAC--GGC-GTGTAGGACAAATCACCACCAAGGAGCTCGGCACTGTCATGCGCTCCCTGGGTCAAAACCCCTCCGAGTCCGAGCTGCAGGATATGATCAACGAGGTCGACGCCGACAACAACGGCACTATTGACTTCCCTGGTAAGTCCAGATGC-TCGCCTA-CTGAATATT

>Diaporthe_megalospora_CBS_143_27

CTTCTCCCTCTTTGTAAGTTATGTCC---AGGA---GCCTTGAGC-----TTCCCCGCCGT--CCCATGCCGTTGCGCATG--ATGTTAACGGACCGTTTTCGGCCTGCAGGATAAGGATGGCGATGGTTAGTGCGGTCACCGCT----TGATTCCCTCT-TTCTCAGCTACGCACGCGTCATATTCGATCCGCCGCGACGGTCTGCGCG-TGCAGTATACTC-CGAGCGACCG-ATCATCAA----------ATATATCACGAGTAGTATGCTAAGGCG-GGC-GTGTAGGACAAATCACCACCAAGGAGCTCGGCACAGTCATGCGGTCCCTTGGTCAAAACCCTTCCGAGTCCGAGCTGCAAGATATGATCAACGAGGTTGACGCCGACAACAATGGCACCATTGACTTCCCTGGTAAGTCGAGATTC-CTGCACA-CTGGATATN

>Diaporthe_melonis_CBS_507_78

CTTCTCCCTCTTTGTAAGTTGTCTTC---AGGA---GCCTTGAGC-----TTCCCCGCCGT--TCTCTGCCGTTGCGCATG--ATGCTAACGGACCGTTTTCGGCCTGCAGGATAAGGATGGCGATGGTTAGTGCGGTCACCGCT----TCCTCCCCTCT-TTCTCAGCTACGCACGCGTCA-ACTCGATCCGCCGCGACGGTCTGCGCG-TGCAGTATATTC-CGAGCGACTG-ATGATCAC----------ATCCATCACGAGTGGTATGCTAAGGCG-GGC-GTGTAGGACAAATCACCACCAAGGAGCTCGGCACAGTCATGCGGTCCCTTGGTCAAAACCCTTCCGAGTCCGAGCTGCAGGACATGATCAACGAGGTCGACGCCGACAACAATGGCACCATTGACTTCCCTGGTGAGTCTAGATTC-TCGCACA-GTGGATATN

>Diaporthe_middletonii_BRIP_54884e

?????????????????????????????????????????????????????????????????????????????????????????????????????????????????????????????????????????????????????????????????????????????????????????????????????????????????????????????????????????????????????????????????????????????????????????????????????????????????????????????????????????????????????????????????????????????????????????????????????????????????????????????????????????????????????????????????

>Diaporthe_miriciae_BRIP_54736j

?????????????????????????????????????????????????????????????????????????????????????????????????????????????????????????????????????????????????????????????????????????????????????????????????????????????????????????????????????????????????????????????????????????????????????????????????????????????????????????????????????????????????????????????????????????????????????????????????????????????????????????????????????????????????????????????????

>Diaporthe_myracrodruonis_URM7972

NNNNNNNNNNNNNNNNNNNNNNNNNNNNNNNNNNNNNNNNNNNNC-----TTCCCCGCCAT--CCTGTGCTTTTGCGCATG--ATGCTAACGGACCGTTTTCGGCCTGCAGGATAAGGATGGCGATGGTTAGTGTGGTCACCGCT----TTCTTCCCTCT-TTCTCAGCTACGCACGCGTCATACTCGATCCGCCGCGACGGTCTGCGCG-TGCAGCATGCTC-CGAGCGACCG-ACCATCAC----------GTCTATCACGAGTAACATGCTAAGTC--GGC-GTGTAGGACAAATCACCACCAAGGAGCTCGGCACAGTCATGCGGTCCCTTGGTCAAAACCCTTCCGAGTCCGAGCTGCAGGACATGATCAACGAGGTCGACGCCGACAACAATGGCACCATTGACTTCCCTGGTAAGTCTTGATGC-TCATACC-CTAAATGTT

>Diaporthe_neoarctii_CBS_109490

CTTCTCCCTCTTTGTAAGTTATTCTC---ACCA---GCCTTGAGC----TCCCCACGCCGT--GTTCTGCCTTTGCGCATG--ATGCTAACGGACCGTTTTCGGCCTGCAGGATAAGGATGGTGATGGTTAGTGCGGTCACCGCT----TCCCTCCCTCT-TTCTCAGCTACCCACGCGTCATACTCGATCCGCCGCGACGATCTGCGCG-TGCAGTACATCC-TGAGCGAGCG-AACCTCGT----------ATCGATCACGAGAAATATGCTAAGAC--GGC-GTGTAGGACAAATCACCACCAAGGAGCTCGGCACAGTCATGCGGTCCCTTGGTCAAAACCCTTCCGAGTCCGAGCTGCAGGACATGATCAACGAGGTCGACGCCGACAACAATGGCACCATTGACTTCCCTGGTAAGTCTAGATGT-CCACCCA-CTGGATGTT

>Diaporthe_neoraonikayaporum_MFLUCC_14_1136

NNNNNNNNNNNNNNNNNNNNNNNNNNNNNNNCA---GCTTTGGGC-----CTGCCCGCCGT--CCACTGCTGTCGCGCATG--ATGCTAACGGACCGTTTTCGGCCTGCAGGATAAGGATGGCGATGGTTAGTGTGATCACCACT----TTCTTCCCTA--GTCCTAGCTACGCACGCCTCACACTTGGCCCGCCGCGACGGTCTGCGCG-TGCA-TGT-----CAAGCGACCG-AATACT------------ACCCATCACGAGTATCATGCTAAGAC--GAC-GTGTAGGACAAATCACCACCAAGGAGCTCGGCACTGTCATGCGGTCCCTAGGTCAAAACCCGTCCGAGTCCGAGCTGCAGGATATGATCAACGAGGTTGACGCCGACAACAACGGCACCATTGACTTCCCTGGTAAGCCCAGATGC-TCGCCTA-CCGGATATT

>Diaporthe_novem_CBS_127271

CTTCTCCCTCTTTGTAAGTTATTCTC---GCCA---GCCTCGAGC----TCCCCCCGCCAT--GTTCTGCCTTTGCGCATG--ATGCTAACGGACCGTTTTCGGCCTGCAGGATAAGGATGGCGATGGTTAGTGCGGTCACCGCT----TCCTTCCCTCT-TTCTCAGCTACCCACGCGTCATACTCGATCCGCCGCGACGGTCTGCGCA-TGCAGTACACTC-TGAGCGAGCG-AACCTCAT----------ATCGATCACGAGAAATATGCTAAGAC--GGC-GTGTAGGACAAATCACCACCAAGGAGCTCGGCACAGTCATGCGGTCCCTTGGTCAAAACCCTTCCGAGTCCGAGCTGCAGGACATGATCAACGAGGTCGACGCCGACAACAATGGCACCATTGACTTCCCTGGTAAGTCTACATGT-CCACCCA-CTGAATATT

>Diaporthe_ovalispora_ICMP20659

?????????????????????????????????????????????????????????????????????????????????????????????????????????????????????????????????????????????????????????????????????????????????????????????????????????????????????????????????????????????????????????????????????????????????????????????????????????????????????????????????????????????????????????????????????????????????????????????????????????????????????????????????????????????????????????????????

>Diaporthe_pachirae_CDA_728

NNNNNNNNNNNNNNNNNNNNNNNNNNNNNNNNNNNNNNNNNNNNNNNNNNNNNNNNNNNNNNNCCTGTGCTTTTGCGCATG--ATGCTAACGGACCGTTTTCGGCCTGCAGGATAAGGATGGCGATGGTTAGTGTGGTCACCGCT----TTCTCCCCTCT-TTCTCAGCTACGCACGCGTCATACTCGATCCGCCGCGACGGTCTGCGCG-TGCAGCATGCTC-CGAGCGACCG-ATCATCAC----------GTCTATCACGAGTAACATGCTAAGTC--GGG-GTGTAGGACAAATCACCACCAAGGAGCTCGGCACAGTCATGCGGTCCCTTGGTCAAAACCCTTCCGAGTCCGAGCTGCAGGACATGATCAACGAGGTCGACGCCGACAACAATGGCACCATCGACTTCCCTGGTAAGTCTGGATGC-TCATCCC-CTAGACGTT

>Diaporthe_passifloricola_CBS_141329

?????????????????????????????????????????????????????????????????????????????????????????????????????????????????????????????????????????????????????????????????????????????????????????????????????????????????????????????????????????????????????????????????????????????????????????????????????????????????????????????????????????????????????????????????????????????????????????????????????????????????????????????????????????????????????????????????

>Diaporthe_pseudolongicolla_CBS_117165

?????????????????????????????????????????????????????????????????????????????????????????????????????????????????????????????????????????????????????????????????????????????????????????????????????????????????????????????????????????????????????????????????????????????????????????????????????????????????????????????????????????????????????????????????????????????????????????????????????????????????????????????????????????????????????????????????

>Diaporthe_pyracanthae_CBS142384

NNNNNNNNTCTTTGTAAGTTATCTTC---GGAG---GCCTTGAGC-----TTCCCCGCCAT--CCTGTGCTTTTGCGCATG--ATGCTAACGGACCGTTTTCGGCCTGCAGGATAAGGATGGCGATGGTTAGTGTGGTCACCGCT----TTCTCCCCTCT-TTCTCAGCTACGCACGCGTCATACTCGATCCGCCGCGACGGTCTGCGCG-TGCAGCATGCTC-CGAGCGACCG-ATCATCAC----------GTCTATCACGAGTAACATGCTAAGTC--GGC-GTGTAGGACAAATCACCACCAAGGAGCTCGGCACAGTCATGCGGTCCCTTGGTCAAAACCCTTCCGAGTCCGAGCTGCAGGACATGATCAACGAGGTCGACGCCGACAACAATGGCACCATTGACTTCCCTGGTAAGTCTGGATGC-TCATCCC-CTAGACATT

>Diaporthe_racemosae_CBS_143770

???????????????????????????????????????????????????????????GT--TCTCTGCTGTCGCGCATG--ATGCTAACGGACTGTTTTCGGCCTGCAGGATAAGGATGGCGATGGTTAGTGCGGTCACCGCT----TCCTCCCCTCT-TTCTCAGCTACGCACGCGTCATACTCGGTCCGCCGCGACGATCTGCGCG-TGCAGTACACTC-CGAGCGACCG-ATCGTCAA----------ATCTATCACGAGTAGTATGCTAAGGCG-GGC-GTGTAGGACAAATCACCACCAAGGAGCTCGGCACAGTCATGCGGTCCCTTGGTCAAAACCCTTCCGAGTCCGAGCTGCAGGACATGATCAACGAGGTCGACGCCGACAACAACGGCACTATTGACTTCCCTGGTAAGTCTAGATTC-TCGCACA-CTGGATATN

>Diaporthe_raonikayaporum_CBS_133182

CTTCTCCCTCTTTGTAAGTTGTCTTC---ACCA---GCTTTGGGC-----CTGCCCGCCGT--CCACTGCTGTCGCGCATG--ATGCTAACGGACCGTTTTCGGCCTGCAGGATAAGGATGGCGATGGTTAGTGTGATCACCACT----TTCTTCCCTA--GTCCTAGCTTCGCACGCCTCACGCTTGGCCCGCCGCGACGGTCTGCGCG-TGCA-TGT-----CAAGCGACCG-AATACT------------ACCCATCACGAGTATCATGCTAAGAC--GAC-GTGTAGGACAAATCACCACCAAGGAGCTCGGCACTGTCATGCGGTCCCTGGGTCAAAACCCGTCCGAGTCCGAGCTGCAGGATATGATCAACGAGGTTGACGCCGACAACAACGGCACCATTGACTTCCCTGGTAAGCCCAGATGC-TCGCCTA-CCGGATATT

>Diaporthe_rosae_MFLUCC_17_2658

CTTCTCCCTCTTTGTAAGTTATATCC---AGGA---GCCTTGAGC-----TTCCCCGCCGT--TCTCTGCTGTTGCGCCTG--ATGCTAACGGACCGTTTTCGGCCTACAGGATAAGGATGGCGATGGTTAGTGCGGTCACCGCT----TCCTCCCCTCT-TTCTCGGCTACACACGCGTCATACTCGATCCGCCGCGACGGTCTGCGCG-TGCAGTCCACTC-CGCGCGACCG-ACCACCAA----------ATCTGTCACGAGTAGTATGCTAAGGCT-GGG-ATGTAGGACAAATCACCACCAAGGAGCTCGGCACAGTCATGCGGTCCCTTGGTCAGAACCCTTCCGAGTCCGAGCTGCAGGACATGATCAACGAGGTCGACGCCGACAACAACGGCACCATTGACTTCCCTGGTAAGTCTAGATTC-TCGTACA-CTGGATATN

>Diaporthe_rosiphthora_COAD_2913

???CTCCCTCTTTGTAAGTTATCTTC---AGAA---GCTTTGAGC-----TTCCCCGCGGT--TCTCTGCTGTTGCGCACG--ATGCTAACGGACCGTTGTCGGCCTGCAGGATAAGGATGGCGATGGTTAGTGCGGTCACCGCT----TCCTTCCCTCT-TTCTCAGCTACGCACGCGTCACACTCGATCCGCCGCGACGGTCTGCGCG-TGCAGAATACTC-CGAGCGACCG-ATCATCAA----------ATCTATCACGAGGAAAATGCTAAGACC-GGC-GTGTAGGACAAATCACCACCAAGGAGCTCGGCACAGTCATGCGGTCCCTTGGTCAAAACCCTTCCGAGTCCGAGCTGCAGGACATGATCAACGAGGTCGACGCCGACAACAATGGCACCATTGACTTCCCTGGTAAGTCTAGATTC-TCGCACA-CTGGATATN

>Diaporthe_rossmaniae_CAA762

NNNNNNNNTCTTTGTAAGTTATCTTC---GGAG---GCCTTGAGC-----TTCCCCGCCAT--CCTGTGCTTTTGCGCATG--ATGCTAACGGACCGTTTTCGGCCTGCAGGATAAGGATGGCGATGGTTAGTGTGGTCACCGCT----TTCTCCCCTCT-TTCTCAGCTACGCACGCGTCATACTCGATCCGCCGCGACGGTCTGCGCG-TGCAGCATGCTC-CGAGCGACCG-ATCATCAC----------GTCTATCACGAGTAACATGCTAAGTC--GGC-GTGTAGGACAAATCACCACCAAGGAGCTCGGCACAGTCATGCGGTCCCTTGGTCAAAACCCTTCCGAGTCCGAGCTGCAGGACATGATCAACGAGGTCGACGCCGACAACAATGGCACCATTGACTTCCCTGGTAAGTCTGGATGC-TCATCCC-CTAGACATT

>Diaporthe_sackstonii_BRIP_54669b

?????????????????????????????????????????????????????????????????????????????????????????????????????????????????????????????????????????????????????????????????????????????????????????????????????????????????????????????????????????????????????????????????????????????????????????????????????????????????????????????????????????????????????????????????????????????????????????????????????????????????????????????????????????????????????????????????

>Diaporthe_schini_CBS_133181

CTTCTCCCTCTTTGTAAGTTATCTTT---AGGA---GCCTTGAGC-----TCCCCCGCCGT--TCTATGCTGTCGCGCATG--ATGCTAACGGACCGTTTTCGGCCTGCAGGATAAGGATGGCGATGGTTAGTGCGGTCACCGCT----TCCTTCCCTCT-TTCTCAGCTACGCACGCGTCATACTCGATCCGCCGCGACGGTCTGCGCG-TGCAGTATACTC-CGAGCGACCG-ATCATCAA----------ATCTATCACGAGTAGTATGCTAAGGCG-GGC-GTGTAGGACAAATCACCACCAAGGAGCTCGGCACAGTCATGCGGTCCCTTGGTCAAAACCCCTCCGAGTCCGAGCTGCAGGACATGATCAACGAGGTCGACGCCGACAACAATGGCACCATTGACTTCCCTGGTAAGTCGAGATTC-TCGCACA-CTGGATATN

>Diaporthe_schoeni_MFLU_15_1279

NNNNNNNNNNNNNNNNNNNNNNNNNNNNNNNNNNNNNNCTTAGCT-----CCCCCCGCCAT--GTTCTGCTGTTGCGCATG--ATGCTAACGGACCGTTTTCGGCCTGCAGGATAAGGATGGCGATGGTTAGTGCGGTCACCGCT----TCCTCCCCTCT-CTCTCAGCTACCCACGCGTCATACTCGATCCGCCGCGACGGTCTGCGCG-TGCAGTACACTCTTGAGCGAGCG-AACCTCAC----------ATCGATCACGAGAAATATGCTAAGAC--GGC-GTGTAGGACAAATCACCACCAAGGAGCTCGGCACAGTCATGCGGTCCCTTGGTCAAAACCCTTCCGAGTCCGAGCTGCAGGACATGATCAACGAGGTCGACGCCGACAACAACGGCACCATTGACTTCCCTGGTGAGTCTAGATAT-CCACCCA-CTG-ATATT

>Diaporthe_sclerotioides_CBS_296_67

CTTCTCCCTCTTTGTAAGTCATCTTC---AGCA---GCCTTGGGC-----CTCCCCGCCGC--CCGCTGCCGTCGCGCATG--ATGCTAACCGACCGTTTTCGGCCTGCAGGATAAGGATGGCGATGGTTAGTGTGGCCACCACT----TTCTTCCCTCT-TTGCTAGCTACGCACGCGTCACACTCGATCCGCCGCGACGGTCTGCGCG-TGCA-TATACTC-CGAGCGACCG-ATCGTCAC----------ATCCATCA-GGATACCATGCTAAGAC--GGC-GTGTAGGACAAATCACCACCAAGGAGCTCGGCACGGTCATGCGTTCCCTCGGTCAAAACCCCTCCGAGTCTGAGCTGCAGGATATGATCAACGAGGTCGACGCCGACAACAATGGCACCATTGACTTCCCTGGTAAGTCTAGATGC-TTGCCCA-CCGGGTGTT

>Diaporthe_serafiniae_BRIP_55665a

?????????????????????????????????????????????????????????????????????????????????????????????????????????????????????????????????????????????????????????????????????????????????????????????????????????????????????????????????????????????????????????????????????????????????????????????????????????????????????????????????????????????????????????????????????????????????????????????????????????????????????????????????????????????????????????????????

>Diaporthe_siamensis_MFLUCC_10_0573a

?????????????????????????????????????????????????????????????????????????????????????????????????????????????????????????????????????????????????????????????????????????????????????????????????????????????????????????????????????????????????????????????????????????????????????????????????????????????????????????????????????????????????????????????????????????????????????????????????????????????????????????????????????????????????????????????????

>Diaporthe_sinensis_ZJUP0033_4

?????????????????????????????????????????????????????????????????????????????????????????????????????????????????????????????????????????????????????????????????????????????????????????????????????????????????????????????????????????????????????????????????????????????????????????????????????????????????????????????????????????????????????????????????????????????????????????????????????????????????????????????????????????????????????????????????

>Diaporthe_stewartii_CBS_193_36

?????????????????????????????????????????????????????????????????????????????????????????????????????????????????????????????????????????????????????????????????????????????????????????????????????????????????????????????????????????????????????????????????????????????????????????????????????????????????????????????????????????????????????????????????????????????????????????????????????????????????????????????????????????????????????????????????

>Diaporthe_subordinaria_CBS_101711

CTTCTCCCTCTTTGTAAGTTATTCTC---ACCA---GCCTTGAGC----TCCCCCCGCCAT--GTTCTGCCTTTGCGCATG--ATGCTAACGGACCGTTTTCGGCCTGCAGGATAAGGATGGCGATGGTTAGTGCGGTCACCGCT----TCCTCCCCTCT-CTCTCAGCTACCCACGCGTCATACTCGATCCGCCGCGACGGTCTGCGCG-TGCAGTACACTCTTGAGCGAGCG-AACCTCAT----------ATCGATCACGAGAAATATGCTAAGAC--GGC-GTGTAGGACAAATCACCACCAAGGAGCTCGGCACAGTCATGCGGTCCCTTGGTCAAAACCCTTCCGAGTCCGAGCTGCAGGACATGATCAACGAGGTCGACGCCGACAACAATGGCACCATTGACTTCCCTGGTAAGTCTAGATAT-CCACCCA-CTGGATATT

>Diaporthe_tecomae_CBS_100547

CTTCTCCCTCTTTGTAAGTTATCTTT---AGGA---GCCTTGAGC-----TTCCCCGCCGT--TCTCTGCTGTTGCGCGTG--ATGCTAATGGACCGTTTTCGGCCTGCAGGATAAGGATGGCGATGGTTAGTGCGGTCACCGCT----TCCTTCCCTCT-TTCTCAGCTACGCACGCGTCATACTCGATCCACCGCGACGGTCTGCGCG-TGCAGTATACCC-CGAGCGACCG-ATCATCAA----------ATCTATCACGAGTAGTATGCTAAGGCG-GGC-GTGTAGGACAAATCACCACCAAGGAGCTCGGCACAGTCATGCGGTCCCTTGGTCAAAACCCTTCCGAGTCCGAGCTGCAGGACATGATCAACGAGGTCGACGCCGACAACAATGGCACCATTGACTTCCCTGGTAAGCCTAGATTC-TCGCATA-CTGGATATN

>Diaporthe_tectonae_MFLUCC_12_0777

NNNNNNNNNNNNNNNNNNNNNNNNNNNNNNNNNNNTGACCCTGAG-----CCTCCCGCCAC--GCTCTGCTGTTGTCCATG--ATGCTAACGGACCGTTTTCGGCCCGCAGGATAAGGATGGCGATGGTTAGTGTGGTCACCACC----TTCTTCCCTCT-TCCTCAGCTACGCACGCGTCATGCTCGATCCGCCGCGACGGCCTGCGCG-TGCA-TATAATC-CAAGCGACCG-ATCATCG-----------ATCCATCACCAGTACCATGCTAAGACG-GGC-GTGCAGGACAAATCACCACCAAGGAGCTCGGCACGGTCATGCGGTCCCTGGGTCAAAACCCCTCCGAGTCTGAGCTGCAGGACATGATTAACGAGGTCGATGCCGACAACAATGGCACCATTGACTTCCCTGGTAAGCCAAGATGC-TCGCCCG-CCGAGTGTT

>Diaporthe_tectonendophytica_MFLUCC_13_0471

?????????????????????????T---AGGT---GCCTTGAGC-----TTCCCCGCCGT--TCTCTGCTGTTGCGCATG--ATGCTAACTGACCGTTTTCGGCCTACAGGATAAGGATGGCGATGGTTAGTGCGGTCACCGCT----TCCTCCCCTCT-TTCTCAGCTACGCACGCGTCATACTCGATCCGCCGCGACGGTCTGCGCG-TGCAGTATATTC-CGAGCGACCG-ATCATCAA----------ATCTATCACGAGTAGTATGCTAAGGTG-GGC-GTGCAGGACAAATCACCACCAAGGAGCTCGGCACAGTTATGCGGTCCCTTGGTCAAAACCCTTCCGAGTCCGAGCTGCAGGACATGATCAACGAGGTCGACGCCGACAACAACGGCACCATTGACTTCCCTGGTAAGTCTAGATTC-TCGTACA-ATGGGTATN

>Diaporthe_terebinthifolii_CBS_133180

CTTCTCCCTCTTTGTAAGTTATATTT---AGGA---GCCTTGAGC-----TTCCCCGCCGT--TCTCTGCTGTTGCGCGTA--ATGCTAACGGACCGTTTTCGGCCTGCAGGATAAGGATGGCGATGGTTAGTGCGGTCACCGCT----TCCTTCCCTCT-TTCTCAGTTATGCACGCGTCATACTCGATCCGCCGCGACGGTCTGCGCG-TGCAGTATACCC-CGAGCGACCG-ATCATCAA----------ATCTATCACGAGTAGTATGCTAAGGCA-GGC-GTGTAGGACAAATCACCACCAAGGAGCTCGGCACAGTCATGCGGTCCCTTGGTCAAAACCCTTCCGAGTCCGAGCTGCAGGACATGATCAACGAGGTCGACGCCGACAACAATGGCACCATTGACTTCCCTGGTAAGTCTAGATTC-TCGCACA-CTGGATATN

>Diaporthe_thunbergiicola_MFLUCC_12_0033

?????????????????????????????????????????????????????????????????????????????????????????????????????????????????????????????????????????????????????????????????????????????????????????????????????????????????????????????????????????????????????????????????????????????????????????????????????????????????????????????????????????????????????????????????????????????????????????????????????????????????????????????????????????????????????????????????

>Diaporthe_tulliensis_BRIP_62248a

?????????????????????????????????????????????????????????????????????????????????????????????????????????????????????????????????????????????????????????????????????????????????????????????????????????????????????????????????????????????????????????????????????????????????????????????????????????????????????????????????????????????????????????????????????????????????????????????????????????????????????????????????????????????????????????????????

>Diaporthe_ueckerae_FAU_656

CTTCTCCCTCTTTGTAAGTTATATCC---AGGA---GCCTTGAGC-----TTCCCCGCCGT--TCTCTGCTGTTGCGCCTG--ATGCTAACGGACCGTTTTCGGCCTACAGGATAAGGATGGCGATGGTTAGTGCGGTCACCGCT----TCCTCCCCTCT-TTCTCGGCTACACACGCGTCATACTCGATCCGCCGCGACGGTCTGCGCG-TGCAGTCTACTC-CGAGCGACCG-ACCATCAA----------ATCTATCACGAGTAGTATGCTAAGGCT-GGG-GTGTAGGACAAATCACCACCAAGGAGCTCGGCACAGTCATGCGGTCCCTTGGTCAGAACCCTTCCGAGTCCGAGCTGCAGGACATGATCAACGAGGTCGACGCCGACAACAACGGCACCATTGACTTCCCTGGTGAGTCTAGATTC-TCGTACA-CTGGATATN

>Diaporthe_unshiuensis_CGMCC3_17569

?????????????????????????????????????????????????????????????????????????????????????????????????????????????????????????????????????????????????????????????????????????????????????????????????????????????????????????????????????????????????????????????????????????????????????????????????????????????????????????????????????????????????????????????????????????????????????????????????????????????????????????????????????????????????????????????????

>Diaporthe_vexans_CBS_127_14

CTTCTCCCTCTTTGTAAGTTTTGTTC---AGGAGGGGCCTTGAGC-----TTCCCCGCCGT--TCTCTGCCGTTGCGCGTC--ATGCTAACGAACCGTTTTCGGCCTGCAGGATAAGGATGGCGATGGTTAGTGCGGTCACCGCT----TCCCTCCCTCT-CTCTCAGCTACGCACGCATCACACTCGATCCGCCGCGACGGTCTGCGCT-CGCCATATGTTC-CGAGCGACCG-ATCATCAA----------ATATACAACGACTAGTGTGCTAAGGCG-GAC-GTGTAGGACAAATCACCACCAAGGAGCTCGGCACAGTCATGCGGTCCCTTGGTCAAAACCCTTCCGAGTCCGAGCTGCAGGACATGATCAACGAGGTCGACGCCGACAACAATGGCACCATTGACTTCCCTGGTAAGTCTAGATCC-TCGCACA-CTGGATATN

>Diaporthe_yunnanensis_CGMCC_3_18289

CTTCTCCCTCTTTGTAAGTCATCTTA---ACTA---GCCTCAAGC-----CTCCCCGCCCT--GCTCTGCTGTCGCGCATG--ATGCTAACGGACCGTTTTCGGCTCGCAGGATAAGGATGGCGATGGTTAGTGCAGCCACCACTTCTCTCTCTCTTTCTCTTTCCAACTACGCACGCGTCACTCTTGATCCGCTACGACGGTCTACGCG-TGCA-TATACTC-CAACCGACCG-ATCATCAC----------ATCCATCACGAGTACCATGCTAAGAC--GGC-GTGTAGGACAAATCACCACCAAGGAGCTCGGCACGGTCATGCGGTCCCTGGGTCAAAACCCCTCCGAGTCCGAGCTGCAGGATATGATCAATGAGGTCGACGCCGACAACAATGGCACCATTGACTTCCCTGGTAAGTCTAGATGC-TCGCATA-CTGAATGTT

>Phomopsis_glabrae_SCHM_3622

?????????????????????????????????????????????????????????????????????????????????????????????????????????????????????????????????????????????????????????????????????????????????????????????????????????????????????????????????????????????????????????????????????????????????????????????????????????????????????????????????????????????????????????????????????????????????????????????????????????????????????????????????????????????????????????????????

>Phomopsis_micheliae

?????????????????????????????????????????????????????????????????????????????????????????????????????????????????????????????????????????????????????????????????????????????????????????????????????????????????????????????????????????????????????????????????????????????????????????????????????????????????????????????????????????????????????????????????????????????????????????????????????????????????????????????????????????????????????????????????

>Phomopsis_vitimegaspora_STE_U2675

?????????????????????????????????????????????????????????????????????????????????????????????????????????????????????????????????????????????????????????????????????????????????????????????????????????????????????????????????????????????????????????????????????????????????????????????????????????????????????????????????????????????????????????????????????????????????????????????????????????????????????????????????????????????????????????????????

>Diaporthe_subellipicola_KUMCC_17_0153

?????????????????????????????????????????????????????????????????????????????????????????????????????????????????????????????????????????????????????????????????????????????????????????????????????????????????????????????????????????????????????????????????????????????????????????????????????????????????????????????????????????????????????????????????????????????????????????????????????????????????????????????????????????????????????????????????

>Diaporthe_masirevicii_BRIP_57892a

?????????????????????????????????????????????????????????????????????????????????????????????????????????????????????????????????????????????????????????????????????????????????????????????????????????????????????????????????????????????????????????????????????????????????????????????????????????????????????????????????????????????????????????????????????????????????????????????????????????????????????????????????????????????????????????????????

>Diaporthe_sojae_CBS_139282

CTTCTCCCTCTTTGTGAGTTATCTCC---AGGA---GCCTTGAGC-----TCCCCCGCCGT--TCTCTGCCGTTGCGCATG--ATGCTAACGGACCGTTTTCGGCCTGCAGGATAAGGATGGCGATGGTTAGTGCGGTCACCGCT----TCCTCCCCTCT-TTCCCGGCTACGCACGCGTCATGCTCGATCCGCCGCGACGGTCTGCGCC-TGCAGTCTACTC-CGAGCGACCG-ATCATCAA----------ATCTATCACGAGTGGTATGCTAAGGCT-GGC-ATGTAGGACAAATCACCACCAAGGAGCTCGGCACAGTCATGCGGTCGCTTGGTCAAAACCCTTCCGAGTCCGAGCTGCAGGACATGATCAACGAGGTCGACGCCGACAACAATGGCACCATTGACTTCCCTGGTAAGTCTCAACTG-T--CACA-CCGGGGATN

>Diaporthe_sambucusii_CFCC_51986

NNNNNNNNTCTTTGTAAGTTATATCC---AGCA---ACCTTGGGC-----CCCCCCGCCGT--CCTCTGCTGTTGCGCATG--ATGCTAACGGACCGTTTTCGGCCTGCAGGATAAGGATGGCGATGGTTAGTGTGGTCACCGCT----TTCTTCCCTCC-TCCTCAGCCACGCACGCGTCATGCTCGATCCGCCGCGACGGTCTGCGCG-TGCATTATGCCC-CGAGCGATCGAATCATCAC----------ACCCATCACGAGTACCATGCTAAGAC--GGC-GTGCAGGACAAATCACCACCAAGGAGCTCGGCACGGTCATGCGGTCCCTGGGCCAAAACCCCTCCGAGTCCGAGCTGCAGGACATGATCAACGAGGTCGACGCCGACAACAACGGCACCATTGACTTCCCTGGTAGGTCCACATGT-CCACCCA-CTGGATGTT

>Diaporthe_vochysiae_LGMF1583

CTTCTCCCTCTTTGTAAGTTATATCC---AGGA---GCCTTGAGC------TCCCCGCCGT--TCTCTGCTGTTGCGCCTG--ATGCTAACGGACCGTTTTCGGCCTACAGGATAAGGATGGCGATGGTTAGTGCGGTCACCGCT----TCCTCCCCTCT-TTCTCAGCTACACACGCGTCATACTCGATCCGCCGCGACGGTCTGCGCG-TGCAGTCTACTC-CGAGCGACCG-ACCATCAA----------ATCTATCACGAGTAGTATGCTAAGGCT-GGG-GTGTAGGACAAATCACCACCAAGGAGCTCGGCACAGTCATGCGGTCCCTTGGTCAGAACCCTTCCGAGTCCGAGCTGCAGGACATGATCAACGAGGTCGACGCCGACAACAACGGCACCATTGACTTCCCTGGTAAGTCTAGATTC-TCGTACA-CTGGATATN

>Diaporthe_endophytica_CBS_133811

CTTCTCCCTCTTTGTGAGTTATCTCC---CGGA---GCCTTGAGC-----TTCCCCGCCGT--TCTCTGCCGTTGCGCATG--ATGCTAACGGACCGTTTTCGGCCTGCAGGATAAGGATGGCGATGGTTAGTGCGGTCACCGCT----TCCTCCCCTCT-TTCCCGGCTACGCACGCGTCATGCTCGATCCGCCGCGACGGTCTGCGCG-TGCAGTCTACTC-CGAGCGACCG-ATCATCAA----------ATCTATCACGGGTGGTATGCTAAGGCT-GGC-ATGTAGGACAAATCACCACCAAGGAGCTCGGCACAGTCATGCGGTCGCTTGGTCAAAACCCTTCCGAGTCCGAGCTGCAGGACATGATCAACGAGGTCGACGCCGACAACAACGGCACCATTGACTTCCCTGGTAAGTCTCAACTG-T--TACA-CTGGGGATN

>Diaporthe_phaseolorum_CBS_113425

CTTCTCCCTCTTTGTGAGTTATCTCC---AGGA---GCCTTGAGC-----TCCCCCGCCGT--TCTCTGCCGTTGCGCATG--ATGCTAACGGACCGTTTTCGGCCTGCAGGATAAGGATGGCGATGGTTAGTGCGGTCACCGCT----TCCTCCCCTCT-TTCCCGGCTACGCACGCGTCATGCTCGATCCGCCGCGACGGTCTGCGCC-TGCAGTCTACTC-CGAGCGACCG-ATCATCAA----------ATCTATCACGAGTGGTATGCTAAGGCT-GGC-ATGTAGGACAAATCACCACCAAGGAGCTCGGCACAGTCATGCGGTCGCTTGGTCAAAACCCTTCCGAGTCCGAGCTGCAGGACATGATCAACGAGGTCGACGCCGACAACAATGGCACCATTGACTTCCCTGGTAAGTCTCAACTG-T--CACA-CCGGGGATN

>Diaporthe_caliensis_STMA_22040

?????????????????????????????????????????????????????????????????????????????????????????????????????????????????????????????????????????????????????????????????????????????????????????????????????????????????????????????????????????????????????????????????????????????????????????????????????????????????????????????????????????????????????????????????????????????????????????????????????????????????????????????????????????????????????????????????

**Alignment of the *his3* sequences used in the phylogenetic study**

>Diaporthe_acaciarum_CBS_138862

TCCGCGCCCTCCACCGGAGGTGTCAAGAAGCCTCACCGCTACAAGCCTGGTACCGTCGCTCTGCGTGAGATCCGTCGCTACCAGAAGAGCACCGAGCTGCTGATCCGCAAGCTGCCCTTCCAGCGTCTGGTATG----------------------------------------------------------------------------CAGGTCCGTGAGATCGCCCAGGACTTCAAGTCCGACCTGCGTTTCCAGTCTTCCGCCATCGGTGCCCTTCAGGAGTCCGTCGAGTCTTACCTCGTCTCCCTCTTTGAGGACACCAACCTGTGCGCCATCCACGCNNNNNNNNNNNNNNNNNNNNNNNNNNNNN

>Diaporthe_acericola_MFLUCC_17_0956

?????????????????????????????????????????????????????????????????????????????????????????????????????????????????????????????????????????????????????????????????????????????????????????????????????????????????????????????????????????????????????????????????????????????????????????????????????????????????????????????????????????????????????????????????????????????????????

>Diaporthe_alangii_CFCC_52556

TCCGCGCCCTCCACCGGAGGTGTCAAGAAGCCTCACCGCTACAAGCCTGGTACCGTCGCTCTGCGTGAGATCCGTCGCTACCAGAAGAGCACCGAGCTGCTGATCCGCAAGCTCCCCTTCCAGCGTCTGGTATGGCT--GCACCGTCCCAATAGCGC-------CCGCGCCCTCCTTATTCTCCTGCTGACCGCCT-------CCTCTTCCAGGTCCGTGAGATCGCCCAGGACTTCAAGTCCGACCTGCGCTTCCAGTCTTCTGCCATTGGTGCCCTGCAGGAGTCCGTCGAGTCTTACCTCGTCTCTCTCTTCGAGGACACCAACCTGTGCGCCATCCACGCCAAGCGTGTCACCATCCAGTCGGTACGTT

>Diaporthe_ambigua_CBS_114015

TCCGCGCCGTCCACCGGAGGTGTCAAGAAGCCTCACCGCTACAAGCCTGGTACCGTCGCTCTGCGTGAGATCCGTCGCTACCAGAAGAGCACCGAGCTGCTGATCCGCAAGCTCCCCTTCCAGCGTCTGGTACG----------------------------------------------------------------------------AAGGTTCGTGAGATCGCCCAGGACTTCAAGTCCGACCTGCGCTTCCAGTCTTCCGCCATCGGTGCCCTGCAGGAGTCCGTCGAGTCTTACCTCGTCTCCCTCTTCGAGGACACCAACCTGTGCGCCATCCACGCCAAGCGTGTCACCATCCAGTCGGTACGTC

>Diaporthe_amygdali_CBS_126679

TCCGCGCCCTCCACCGGAGGTGTCAAGAAGCCTCACCGCTACAAGCCTGGTACCGTCGCTCTGCGTGAGATTCGTCGCTACCAGAAGTCCACTGAGCTTCTGATCCGCAAGCTGCCCTTCCAGCGTCTGGTACG----------------------------------------------------------------------------CAGGTTCGTGAGATTGCCCAGGACTTCAAGTCCGACCTCCGCTTCCAGTCCTCCGCCATCGGTGCCCTGCAGGAGTCCGTCGAGTCCTACCTCGTCTCCCTCTTCGAGGACACCAACCTGTGCGCCATCCACGCCAAGCGTGTCACCATCCAGTCGGTATGTA

>Diaporthe_angelicae_CBS_111592

TCCGCGCCCTCCACCGGAGGTGTCAAGAAGCCTCACCGCTACAAGCCTGGTACCGTCGCTCTGCGTGAGATCCGTCGCTACCAGAAGAGCACCGAGCTGCTCATCCGCAAGCTCCCCTTCCAGCGTCTGGTAAG----------------------------------------------------------------------------CAGGTTCGTGAGATCGCCCAGGACTTCAAGTCCGACCTGCGCTTCCAGTCTTCCGCCATCGGCGCCCTCCAGGAGTCTGTCGAGTCTTACCTCGTCTCCCTCTTCGAGGACACCAACCTGTGCGCCATCCACGCCAAGCGTGTCACCATCCAGTCGGTACGTC

>Diaporthe_arctii_CBS_136_25

TCCGCGCCCTCCACCGGAGGTGTCAAGAAGCCTCACCGCTACAAGCCTGGTACCGTCGCTCTGCGTGAGATCCGTCGCTACCAGAAGAGCACTGAGCTGCTCATCCGCAAGCTGCCCTTCCAGCGTCTGGTAAG----------------------------------------------------------------------------CAGGTTCGTGAGATCGCCCAGGACTTCAAGTCCGACCTGCGCTTCCAGTCTTCCGCCATCGGCGCCCTCCAGGAGTCCGTCGAGTCCTACCTCGTCTCCCTCTTCGAGGACACCAACCTGTGCGCCATCCACGCCAAGCGTGTCACCATCCAGTCGGTACGTC

>Diaporthe_arezzoensis_MFLU_19_2880

?????????????????????????????????????????????????????????????????????????????????????????????????????????????????????????????????????????????????????????????????????????????????????????????????????????????????????????????????????????????????????????????????????????????????????????????????????????????????????????????????????????????????????????????????????????????????????

>Diaporthe_batatas_CBS_122_21

TCCGCGCCCTCCACCGGAGGTGTCAAGAAGCCTCACCGCTACAAGCCTGGTACCGTCGCTCTGCGTGAGATCCGTCGCTACCAGAAGAGCACCGAGCTGCTGATCCGCAAGCTCCCCTTCCAGCGTCTGGTATG----------------------------------------------------------------------------CAGGTCCGTGAGATCGCCCAGGACTTCAAGTCCGACCTGCGCTTCCAGTCCTCCGCCATCGGTGCTCTCCAGGAGTCCGTCGAGTCTTACCTCGTCTCCCTCTTTGAGGACACCAACCTGTGCGCCATCCACGCCAAGCGTGTCACCATCCAGTCGGTACGTC

>Diaporthe_beilharziae_BRIP_54792

?????????????????????????????????????????????????????????????????????????????????????????????????????????????????????????????????????????????????????????????????????????????????????????????????????????????????????????????????????????????????????????????????????????????????????????????????????????????????????????????????????????????????????????????????????????????????????

>Diaporthe_biguttulata_ICMP20657

TCCGCGCCCTCCACCGGAGGTGTCAAGAAGCCTCACCGCTACAAGCCTGGTACCGTCGCTCTGCGTGAGATCCGTCGCTACCAGAAGAGCACCGAGCTGCTGATCCGCAAGCTCCCCTTCCAGCGTCTGGTATG----------------------------------------------------------------------------CAGGTTCGTGAGATCGCCCAGGACTTCAAGTCCGACCTGCGCTTCCAGTCTTCCGCCATCGGTGCCCTGCAGGAGTCCGTCGAGTCCTACCTCGTCTCCCTCTTTGAGGACACCAACCTGTGCGCCATCCACGCCAAGCGTGTCACCATCCAGTCGGTACGTC

>Diaporthe_breyniae

TCCGCGCCCTCCACCGGAGGTGTCAAGAAGCCTCACCGCTACAAGCCTGGTACCGTCGCTCTGCGTGAGATCCGTCGCTACCAGAAGAGCACCGAGCTGCTGATCCGCAAGCTCCCCTTCCAGCGTCTGGTATGTTTTGCACCTCACCAA------------TCACCCTCATCCTCGTACACCCTGCTGACTGTCG--CGCCTCCCTCTCCAGGTCCGTGAGATCGCCCAGGACTTCAAGTCCGACCTGCGCTTCCAGTCTTCCGCCATCGGTGCTCTCCAGGAGTCCGTCGAGTCTTACCTCGTCTCCCTCTTCGAGGACACCAACCTGTGCGCCATTCACGCCAAGCGTGTCACCATCCAGTCGGTACGTC

>Diaporthe_camporesii_JZB320143

?????????????????????????????????????????????????????????????????????????????????????????????????????????????????????????????????????????????????????????????????????????????????????????????????????????????????????????????????????????????????????????????????????????????????????????????????????????????????????????????????????????????????????????????????????????????????????

>Diaporthe_caryae_CFCC_52563

TCCGCGCCCTCCACCGGAGGTGTCAAGAAGCCTCACCGCTACAAGCCTGGTACCGTCGCTCTGCGTGAGATCCGTCGCTACCAGAAGAGCACCGAGCTGCTGATCCGCAAGCTCCCCTTCCAGCGTCTGGTATGTCT-GCACCCG-CCAAAC-------------CCCTTCGTGCTACTTTCCCTGCTGACCGCCG-CCCTCTCTGTTTCCAGGTCCGTGAGATCGCCCAGGACTTCAAGTCCGACCTGCGCTTCCAGTCTTCCGCCATCGGTGCCCTTCAGGAGTCCGTCGAGTCTTACCTCGTCTCCCTCTTTGAGGACACCAACCTGTGCGCCATCCACGCCAAGCCAGTCACCATCCAGTCGGTACGTC

>Diaporthe_celtidis_NCYU_19_0357

?????????????????????????????????????????????????????????????????????????????????????????????????????????????????????????????????????????????????????????????????????????????????????????????????????????????????????????????????????????????????????????????????????????????????????????????????????????????????????????????????????????????????????????????????????????????????????

>Diaporthe_cerradensis_CMRP4331

TCCGCGCCCTCTACCGGAGGTGTCAAGAAGCCTCACCGCTACAAGCCTGGTACCGTCGCTCTGCGTGAGATCCGTCGCTACCAGAAGAGCACCGAGCTGCTGATCCGCAAGCTCCCCTTCCAGCGTCTGGTATGTCT--GCACTTGCCAATCA--------------CCGCCCCGACCCGCCAATTCCTGCTGACCATCGCCTCCTTTCCCAGGTTCGTGAGATCGCCCAGGACTTCAAGTCCGACCTGCGCTTCCAGTCTTCCGCCATCGGTGCCCTGCAGGAGTCCGTCGAGTCGTACCTCGTCTCCCTCTTCGAGGACACCAACCTGTGTGCCATCCACGCCAAGCGTGTCACCATCCAGTCGGTATGTC

>Diaporthe_chimonanthi

?????????????????????????????????????????????????????????????????????????????????????????????????????????????????????????????????????????????????????????????????????????????????????????????????????????????????????????????????????????????????????????????????????????????????????????????????????????????????????????????????????????????????????????????????????????????????????

>Diaporthe_chinensis_MFLUCC_19_0101

?????????????????????????????????????????????????????????????????????????????????????????????????????????????????????????????????????????????????????????????????????????????????????????????????????????????????????????????????????????????????????????????????????????????????????????????????????????????????????????????????????????????????????????????????????????????????????

>Diaporthe_chromolaenae_MFLUCC_17_1422

?????????????????????????????????????????????????????????????????????????????????????????????????????????????????????????????????????????????????????????????????????????????????????????????????????????????????????????????????????????????????????????????????????????????????????????????????????????????????????????????????????????????????????????????????????????????????????

>Diaporthe_cichorii_MFLUCC_17_1023

?????????????????????????????????????????????????????????????????????????????????????????????????????????????????????????????????????????????????????????????????????????????????????????????????????????????????????????????????????????????????????????????????????????????????????????????????????????????????????????????????????????????????????????????????????????????????????

>Diaporthe_cinnamomi_CFCC_52569

TCCGCGCCCTCCACCGGAGGTGTCAAGAAGCCTCACCGCTACAAGCCTGGTACCGTCGCTCTGCGTGAGATCCGTCGCTACCAGAAGAGCACCGAGCTGCTGATCCGCAAGCTCCCCTTCCAGCGTCTGGTATGTCT-GCAACCG-CCAAAGA-----------ACCCGTGCTCCCCACTCTCCTGCTGACCGTCG---CCTTCTCTCCCCAGGTTCGTGAGATCGCCCAGGACTTCAAGTCCGACCTGCGCTTCCAGTCTTCCGCCATCGGTGCCCTGCAGGAGTCCGTCGAGTCCTACCTCGTCTCCCTCTTTGAGGACACCAACCTGTGCGCCATCCACGCCAAGCGTGTCACCATCCAGTCGGTACGTT

>Diaporthe_citriasiana_CBS_134240

TCCGCGCCCTCCACCGGAGGTGTCAAGGAGCCTCACCGGCACAAGCGTGGGACCGTGGCTAGGCGTGAGATCCGTCGCTACCAGAAGAGCACCGAGCTGCTCATCCGCAAGCTCCCCTTCCAGCGTCTGGTATG----------------------------------------------------------------------------CAGGTTCGTGAGATCGCCCAGGACTTCAAGTCCGACCTGCGCTTCCAGTCTTCCGCCATCGGTGCCCTGCAGGAGTCCGTCGAGTCCTACCTCGTCTCCCTCTTCGAGGACACCAACCTGTGCGCCATCCACGCCAAGCGTGTCACCATCCAGTCGGTACGTC

>Diaporthe_compacta_LC3083

TCCGCGCCCTCCACCGGAGGTGTCAAGAAGCCTCACCGCTACAAGCCTGGTACCGTCGCTCTGCGTGAGATCCGTCGCTACCAGAAGAGCACCGAGCTGCTCATCCGCAAGCTCCCCTTCCAGCGTCTGGTATG----------------------------------------------------------------------------CAGGTCCGTGAGATCGCCCAGGACTTCAAGTCCGACCTGCGCTTCCAGTCTTCCGCCATCGGTGCCCTTCAGGAGTCCGTCGAGTCTTACCTCGTCTCCCTCTTTGAGGACACCAACCTGTGCGCCATCCACGCCAAGCGTGTCACCATCCAGTCGGTACGTC

>Diaporthe_convolvuli_CBS_124654

TCCGCGCCCTCCACCGGAGGTGTCAAGAAGCCTCACCGCTACAAGCCTGGTACCGTCGCTCTGCGTGAGATCCGTCGCTACCAGAAGAGCACCGAGCTGCTGATCCGCAAGCTCCCCTTCCAGCGTCTGGTATG----------------------------------------------------------------------------CAGGTCCGTGAGATCGCCCAGGACTTCAAGTCCGACCTGCGCTTCCAGTCTTCCGCCATCGGTGCTCTTCAGGAGTCCGTCGAGTCTTACCTCGTCTCTCTCTTCGAGGACACCAACCTGTGCGCCATCCATGCCAAGCGTGTCACCATCCAGTCGGTACGTT

>Diaporthe_cucurbitae_DAOM_42078

TCCGCGCCCTCCACCGGAGGTGTCAAGAAGCCTCACCGCTACAAGCCTGGTACCGTCGCTCTGCGTGAGATCCGTCGCTACCAGAAGAGCACCGAGCTGCTCATCCGCAAGCTGCCCTTCCAGCGTCTGGTAAG----------------------------------------------------------------------------CAGGTTCGTGAGATCGCCCAGGACTTCAAGTCCGACCTGCGCTTCCAGTCTTCCGCCATCGGCGCCCTCCAGGAGTCCGTCGAGTCCTACCTCGTCTCCCTCTTCGAGGACACCAACCTGTGCGCCATCCACGCCAAGCGTGTCACCATCCAGTCGGTACGTC

>Diaporthe_cuppatea_CBS_117499

TCCGCGCCCTCCACCGGAGGTGTCAAGAAGCCTCACCGCTACAAGCCTGGTACCGTCGCTCTGCGTGAGATCCGTCGCTACCAGAAGAGCACCGAGCTGCTGATCCGCAAGCTCCCCTTCCAGCGTCTGGTAAG----------------------------------------------------------------------------CAGGTTCGTGAGATCGCCCAGGACTTCAAGTCCGACCTGCGCTTCCAGTCTTCTGCCATCGGTGCCCTTCAGGAGTCCGTCGAGTCTTACCTCGTCTCCCTCTTTGAGGACACCAACCTGTGCGCCATCCACGCCAAGCGTGTCACCATCCAGTCGGTACGTC

>Diaporthe_discoidispora_ICMP20662

TCCGCGCCCTCCACCGGAGGTGTCAAGAAGCCTCACCGCTACAAGCCTGGCACCGTCGCTCTGCGTGAGATCCGTCGCTACCAGAAGAGCACCGAGCTGCTGATCCGCAAGCTCCCCTTCCAGCGTCTGGTATG----------------------------------------------------------------------------CAGGTTCGTGAGATCGCCCAGGACTTCAAGTCCGACCTGCGCTTCCAGTCTTCCGCCATCGGTGCCCTTCAGGAGTCCGTCGAGTCCTACCTCGTCTCCCTCTTTGAGGACACCAACCTGTGCGCCATCCACGCCAAGCGTGTCACCATCCAGTCGGTACGTT

>Diaporthe_durionigena_VTCC_930005

?????????????????????????????????????????????????????????????????????????????????????????????????????????????????????????????????????????????????????????????????????????????????????????????????????????????????????????????????????????????????????????????????????????????????????????????????????????????????????????????????????????????????????????????????????????????????????

>Diaporthe_eres_CBS_138594

TCCGCGCCCTCCACCGGAGGTGTCAAGAAGCCTCACCGCTACAAGCCTGGTACCGTCGCTCTGCGTGAGATCCGTCGCTACCAGAAGAGCACCGAGCTGCTGATCCGCAAGCTCCCCTTCCAGCGTCTGGTATG----------------------------------------------------------------------------TAGGTCCGTGAGATCGCCCAGGACTTCAAGTCCGACCTCCGCTTCCAGTCTTCCGCCATCGGTGCCCTGCAGGAGTCGGTTGAGTCTTACCTCGTCTCCCTCTTCGAGGACACCAACCTGTGCGCCATCCACGCCAAGCGTGTCACCATCCAGTCGGTACGTT

>Diaporthe_fici_septicae_MFLU_18_2588

?????????????????????????????????????????????????????????????????????????????????????????????????????????????????????????????????????????????????????????????????????????????????????????????????????????????????????????????????????????????????????????????????????????????????????????????????????????????????????????????????????????????????????????????????????????????????????

>Diaporthe_fructicola_MAFF_246408

TCCGCGCCCTCCACCGGAGGTGTCAAGAAGCCTCACCGCTACAAGCCTGGTACCGTCGCTCTGCGTGAGATCCGTCGCTACCAGAAGAGCACCGAGCTGCTGATCCGCAAGCTCCCCTTCCAGCGTCTGGTATGTTTTCACACCCACCCAAAATCAA-----TCAACTTCACCCTCGTTTACCCTGCTGACCGCCG--CCTCTTCCTCCCCAGGTCCGTGAGATCGCCCAGGACTTCAAGTCCGACCTGCGCTTCCAGTCTTCCGCCATCGGTGCTCTCCAGGAGTCCGTCGAGTCTTACCTCGTCTCCCTCTTCGAGGACACCAACCTGTGCGCCATCCACGCCAAGCGTGTCACCATCCAGTCGGTACGTC

>Diaporthe_ganjae_CBS_180_91

TCCGCGCCCTCCACCGGAGGTGTCAAGAAGCCTCACCGCTACAAGCCTGGTACCGTCGCTCTGCGTGAGATCCGTCGCTACCAGAAGAGCACCGAGCTGCTCATCCGCAAGCTCCCCTTCCAGCGTCTGGTATG----------------------------------------------------------------------------CAGGTTCGTGAGATCGCCCAGGACTTCAAGTCCGACCTGCGCTTCCAGTCTTCCGCCATCGGTGCCCTTCAGGAGTCCGTCGAGTCTTACCTCGTCTCCCTCTTTGAGGACACCAACCTGTGCGCCATCCACGCCAAGCGTGTCACCATCCAGTCGGTACGTC

>Diaporthe_goulteri_BRIP_55657a

?????????????????????????????????????????????????????????????????????????????????????????????????????????????????????????????????????????????????????????????????????????????????????????????????????????????????????????????????????????????????????????????????????????????????????????????????????????????????????????????????????????????????????????????????????????????????????

>Diaporthe_guangdongensis_ZHKUCC20_0014

?????????????????????????????????????????????????????????????????????????????????????????????????????????????????????????????????????????????????????????????????????????????????????????????????????????????????????????????????????????????????????????????????????????????????????????????????????????????????????????????????????????????????????????????????????????????????????

>Diaporthe_gulyae_BRIP_54025

?????????????????????????????????????????????????????????????????????????????????????????????????????????????????????????????????????????????????????????????????????????????????????????????????????????????????????????????????????????????????????????????????????????????????????????????????????????????????????????????????????????????????????????????????????????????????????

>Diaporthe_guttulata_CGMCC_3_20100

TCCGCGCCCTCCACCGGAGGTGTCAAGAAGCCTCACCGCTACAAGCCTGGTACCGTCGCTCTGCGTGAGATCCGTCGCTACCAGAAGAGCACCGAGCTGCTGATCCGCAAGCTCCCCTTCCAGCGTCTGGTAAGTCC--TGCATCACAAACTGTCAC------GCCTCTTGCCCTTGCCCTTGCTGACCATCGCCC---TTCTTCTCTTGCAGGTTCGTGAGATCGCCCAGGACTTCAAGTCCGACCTGCGCTTCCAGTCTTCCGCCATCGGTGCCCTGCAGGAGTCCGTCGAGTCTTACCTCGTCTCCCTGTTCGAGGACACCAACCTGTGCGCCATCCACGCCAAGCGTGTCACCATCCAGTCGGTACGTC

>Diaporthe_helianthi_CBS_592_81

TCCGCGCCCTCCACCGGAGGTGTCAAGAAGCCCCACCGCTACAAGCCTGGTACCGTCGCTCTGCGTGAGATCCGTCGTTATCAGAAGAGCACCGAGCTGCTGATTCGCAAGCTCCCCTTCCAGCGTCTGGTATG----------------------------------------------------------------------------TAGGTTCGTGAGATCGCCCAGGACTTCAAGTCCGATCTCCGCTTCCAGTCTTCCGCCATCGGTGCCCTGCAGGAGTCTGTCGAGTCTTACCTCGTCTCCCTCTTTGAGGACACCAACCTGTGCGCCATCCACGCCAAGCGTGTCACCATCCAGTCGGTATGTC

>Diaporthe_heterostemmatis_SAUCC194_85

TCCGCGCCCTCCACCGGAGGTGTCAAGAAGCCTCACCGCTACAAGCCTGGTACCGTCGCTCTGCGTGAGATCCGTCGCTACCAGAAGAGCACCGAGCTGCTGATCCGCAAGCTCCCCTTCCAGCGTCTGGTATGTTTTCACACCCACCCAAAATCAA-----TCAACTTCACCCTCGTTTACCCTGCTGACCGCCG--CCTCTTCCTCCCCAGGTCCGTGAGATCGCCCAGGACTTCAAGTCCGACCTGCGCTTCCAGTCTTCCGCCATCGGTGCTCTCCAGGAGTCCGTCGAGTCTTACCTCGTCTCCCTCTTCGAGGACACCAACCTGTGCGCCATCCACGCCAAGCGTGTCACCATCCAGTCGGTACGTC

>Diaporthe_hordei_CBS_481_92

TCCGCGCCATCCACCGGAGGTGTCAAGAAGCCTCACCGCTACAAGCCTGGTACCGTCGCTCTGCGTGAGATCCGTCGCTACCAGAAGAGCACTGAGCTGCTGATCCGCAAGCTCCCCTTCCAGCGCCTGGTATG----------------------------------------------------------------------------CAGGTCCGTGAGATCGCCCAGGACTTCAAGTCCGACCTGCGCTTCCAGTCTTCCGCCATCGGTGCCCTTCAGGAGTCCGTCGAGTCTTACCTCGTCTCCCTCTTTGAGGACACCAACCTGTGCGCCATCCACGCCAAGCGTGTCACCATCCAGTCGGTACGTC

>Diaporthe_hubeiensis_JZB320123

?????????????????????????????????????????????????????????????????????????????????????????????????????????????????????????????????????????????????????????????????????????????????????????????????????????????????????????????????????????????????????????????????????????????????????????????????????????????????????????????????????????????????????????????????????????????????????

>Diaporthe_infecunda_CBS_133812

TCCGCGCCCTCCACCGGAGGTGTCAAGAAGCCTCACCGCTACAAGCCTGGTACCGTCGCTCTGCGTGAGATCCGTCGCTACCAGAAGAGCACCGAGCTGCTGATCCGCAAGCTGCCCTTCCAGCGTCTGGTATG----------------------------------------------------------------------------TAGGTCCGTGAGATCGCCCAGGACTTCAAGTCCGACCTGCGCTTCCAGTCATCCGCCATCGGTGCCCTTCAGGAGTCCGTCGAGTCTTACCTCGTCTCCCTCTTTGAGGACACCAACCTGTGCGCCATCCACGCCAAGCGTGTCACCATCCAGTCGGTACGTC

>Diaporthe_infertilis_CBS_230_52

TCCGCGCCCTCCACCGGAGGTGTCAAGAAGCCTCACCGCTACAAGCCTGGTACCGTCGCTCTGCGTGAGATCCGTCGCTACCAGAAGAGCACCGAGCTGCTGATCCGCAAGCTCCCCTTCCAGCGTCTGGTATG----------------------------------------------------------------------------CAGGTCCGTGAGATCGCCCAGGACTTCAAGTCCGACCTGCGCTTCCAGTCTTCCGCCATCGGTGCGCTCCAGGAGTCCGTCGAGTCTTACCTCGTCTCCCTCTTTGAGGACACCAACCTGTGCGCCATCCACGCCAAGCGTGTCACCATCCAGTCGGTACGTC

>Diaporthe_kochmanii_BRIP_54033

?????????????????????????????????????????????????????????????????????????????????????????????????????????????????????????????????????????????????????????????????????????????????????????????????????????????????????????????????????????????????????????????????????????????????????????????????????????????????????????????????????????????????????????????????????????????????????

>Diaporthe_kongii_BRIP_54031

?????????????????????????????????????????????????????????????????????????????????????????????????????????????????????????????????????????????????????????????????????????????????????????????????????????????????????????????????????????????????????????????????????????????????????????????????????????????????????????????????????????????????????????????????????????????????????

>Diaporthe_leucospermi_CBS_111980

TCCGCGCCCTCCACCGGAGGTGTCAAGAAGCCTCACCGCTACAAGCCTGGTACCGTCGCTCTGCGTGAGATCCGTCGCTACCAGAAGAGCACCGAGCTGCTGATCCGCAAGCTGCCCTTCCAGCGTCTGGTATG----------------------------------------------------------------------------TAGGTCCGTGAGATCGCCCAGGACTTCAAGTCCGACCTGCGCTTCCAGTCTTCCGCCATCGGTGCCCTTCAGGAGTCCGTCGAGTCTTACCTCGTCTCCCTCTTTGAGGACACCAACCTGTGCGCCATCCACGCCAAGCGTGTCACCATCCAGTCGGTACGTC

>Diaporthe_longicolla_FAU_599

TCCGCGCCCTCCACCGGAGGTGTCAAGAAGCCTCACCGCTACAAGCCTGGTACCGTCGCTCTGCGTGAGATCCGTCGCTACCAGAAGAGCACCGAGCTGCTGATCCGCAAGCTCCCCTTCCAGCGTCTGGTATG----------------------------------------------------------------------------CAGGTCCGTGAGATCGCCCAGGACTTCAAGTCCGACCTGCGCTTCCAGTCTTCCGCCATCGGTGCCCTGCAGGAGTCCGTCGAGTCTTACCTCGTCTCCCTCTTTGAGGACACCAACCTGTGCGCCATCCACGCCAAGCGTGTCACCATCCAGTCGGTACGTC

>Diaporthe_longispora_CBS_194_36

TCCGCGCCCTCCACCGGAGGTGTCAAGAAGCCTCACCGCTACAAGCCTGGTACCGTCGCTCTGCGTGAGATCCGTCGCTACCAGAAGAGCACTGAGCTGCTGATCCGCAAGCTCCCCTTCCAGCGTCTGGTATG----------------------------------------------------------------------------CAGGTCCGTGAGATCGCCCAGGACTTCAAGTCCGACCTGCGCTTCCAGTCTTCTGCCATCGGTGCCCTGCAGGAGTCTGTCGAGTCTTACCTCGTCTCTCTCTTCGAGGACACCAACCTGTGCGCCATCCATGCCAAGCGTGTCACCATCCAGTCGGTACGTC

>Diaporthe_lusitanicae_CBS_123212

TCCGCGCCCTCCACCGGAGGTGTCAAGAAGCCTCACCGCTACAAGCCTGGTACCGTCGCTCTGCGTGAGATCCGTCGCTACCAGAAGAGCACCGAGCTGCTGATCCGCAAGCTCCCCTTCCAGCGTCTGGTAAG----------------------------------------------------------------------------CAGGTTCGTGAGATCGCCCAGGACTTCAAGTCCGACCTGCGCTTCCAGTCTTCTGCCATTGGTGCCCTTCAGGAGTCCGTCGAGTCTTACCTCGTCTCCCTTTTCGAGGACACCAACCTGTGCGCCATCCACGCCAAGCGTGTCACCATCCAGTCGGTACGTC

>Diaporthe_machili_SAUCC194_111

TCCGCGCCCTCCACCGGAGGTGTCAAGAAGCCTCACCGCTACAAGCCTGGTACCGTCGCTCTGCGTGAGATCCGTCGCTACCAGAAGAGCACCGAGCTGCTGATCCGCAAGCTCCCCTTCCAGCGTCTGGTATGTC--GCACCCG-CCAAAC-------------CCCCTCGTGCTACTTCCCCTGCTGACCGTCG-CCCTCTTTGCTTCCAGGTCCGTGAGATCGCCCAGGACTTCAAGTCCGACCTGCGCTTCCAGTCTTCCGCCATCGGTGCCCTTCAGGAGTCCGTCGAGTCTTACCTCGTCTCCCTCTTTGAGGACACCAACCTGTGCGCCATCCACGCCAAGCGTGTCACCATCCAGTCGGTACGTC

>Diaporthe_manihotia_CBS_505_76

TCCGCGCCCTCCACCGGAGGTGTCAAGAAGCCTCACCGCTACAAGCCTGGTACCGTCGCTCTGCGTGAGATCCGTCGCTACCAGAAGAGCACCGAGCTGCTCATCCGCAAGCTCCCCTTCCAGCGTCTGGTATG----------------------------------------------------------------------------CAGGTTCGTGAGATCGCCCAGGACTTCAAGTCCGACCTGCGCTTCCAGTCTTCCGCCATCGGTGCTCTCCAGGAGTCCGTCGAGTCTTACCTCGTCTCCCTCTTTGAGGACACCAACCTGTGCGCCATCCACGCCAAGCGTGTCACCATCCAGTCGGTACGTC

>Diaporthe_mayteni_CBS_133185

TCCGCACCCTCCACCGGAGGTGTCAAGAAGCCCCACCGCTACAAGCCTGGTACCGTCGCTCTGCGTGAGATCCGTCGCTACCAGAAGAGCACTGAGCTGCTGATCCGCAAGCTCCCCTTCCAGCGTCTGGTAAG----------------------------------------------------------------------------CAGGTTCGTGAGATTGCCCAGGACTTCAAGTCCGACCTGCGCTTCCAGTCCTCCGCCATCGGTGCCCTGCAGGAGTCCGTCGAGTCTTACCTCGTCTCCCTCTTCGAGGACACCAACCTGTGCGCCATCCACGCCAAGCGTGTCACCATCCAGTCGGTACGTT

>Diaporthe_megalospora_CBS_143_27

TCCGCGCCCTCCACCGGAGGTGTCAAGAAGCCTCACCGCTACAAGCCTGGTACCGTCGCTCTGCGTGAGATCCGTCGCTACCAGAAGAGCACCGAGCTGTTGATCCGCAAGCTCCCCTTCCAGCGTCTGGTACG----------------------------------------------------------------------------TAGGTCCGTGAGATCGCCCAGGACTTCAAGTCCGACCTGCGCTTCCAGTCTTCCGCCATCGGTGCCCTTCAGGAGTCCGTCGAGTCTTACCTCGTCTCCCTCTTCGAGGACACCAACCTGTGCGCCATCCACGCCAAGCGTGTCACCATCCAGTCGGTACGTC

>Diaporthe_melonis_CBS_507_78

TCCGCGCCCTCCACCGGAGGTGTCAAGAAGCCTCACCGCTACAAGCCTGGTACCGTCGCTCTGCGTGAGATCCGTCGCTACCAGAAGAGCACCGAGCTGCTGATCCGCAAGCTCCCCTTCCAGCGTCTGGTACG----------------------------------------------------------------------------CAGGTCCGTGAGATCGCCCAGGACTTCAAGTCCGACCTGCGCTTCCAGTCTTCCGCCATCGGTGCTCTTCAGGAGTCCGTCGAGTCTTACCTCGTCTCCCTCTTTGAGGACACCAACCTGTGTGCCATCCACGCCAAGCGTGTCACCATCCAGTCGGTACGTC

>Diaporthe_middletonii_BRIP_54884e

?????????????????????????????????????????????????????????????????????????????????????????????????????????????????????????????????????????????????????????????????????????????????????????????????????????????????????????????????????????????????????????????????????????????????????????????????????????????????????????????????????????????????????????????????????????????????????

>Diaporthe_miriciae_BRIP_54736j

?????????????????????????????????????????????????????????????????????????????????????????????????????????????????????????????????????????????????????????????????????????????????????????????????????????????????????????????????????????????????????????????????????????????????????????????????????????????????????????????????????????????????????????????????????????????????????

>Diaporthe_myracrodruonis_URM7972

?????????????????????????????????????????????????????????????????????????????????????????????????????????????????????????????????????????????????????????????????????????????????????????????????????????????????????????????????????????????????????????????????????????????????????????????????????????????????????????????????????????????????????????????????????????????????????

>Diaporthe_neoarctii_CBS_109490

TCCGCGCCCTCCACCGGAGGTGTCAAGAAGCCTCACCGCTACAAGCCTGGTACCGTCGCTCTGCGTGAGATCCGTCGCTACCAGAAGAGCACCGAGCTGCTGATCCGCAAGCTCCCCTTCCAGCGTCTGGTAAG----------------------------------------------------------------------------CAGGTTCGTGAGATCGCCCAGGACTTCAAGTCCGACCTGCGCTTCCAGTCTTCCGCCATCGGCGCTCTTCAGGAGTCTGTCGAGTCTTACCTCGTCTCCCTCTTTGAGGACACCAACCTGTGCGCCATCCACGCCAAGCGTGTCACCATCCAGTCGGTACGTC

>Diaporthe_neoraonikayaporum_MFLUCC_14_1136

?????????????????????????????????????????????????????????????????????????????????????????????????????????????????????????????????????????????????????????????????????????????????????????????????????????????????????????????????????????????????????????????????????????????????????????????????????????????????????????????????????????????????????????????????????????????????????

>Diaporthe_novem_CBS_127271

TCCGCGCCCTCCACCGGAGGTGTCAAGAAGCCTCACCGCTACAAGCCTGGTACCGTCGCTCTGCGTGAGATCCGTCGCTACCAGAAGAGCACCGAGCTGCTCATCCGCAAGCTCCCCTTCCAGCGTCTGGTAAG----------------------------------------------------------------------------CAGGTTCGTGAGATCGCCCAGGACTTCAAGTCCGACCTGCGCTTCCAGTCTTCTGCCATCGGCGCCCTTCAGGAGTCCGTCGAGTCTTACCTCGTCTCCCTCTTCGAGGACACCAACCTGTGCGCCATCCACGCCAAGCGTGTCACCATCCAGTCGGTACGTC

>Diaporthe_ovalispora_ICMP20659

TCCGCGCCCTCCACCGGAGGTGTCAAGAAGCCTCACCGCTACAAGCCTGGTACCGTCGCTCTGCGTGAGATCCGTCGCTACCAGAAGAGCACCGAGCTGCTGATCCGCAAGCTCCCCTTCCAGCGTCTGGTATG----------------------------------------------------------------------------CAGGTCCGTGAGATCGCCCAGGACTTCAAGTCCGACCTGCGCTTCCAGTCTTCCGCCATCGGTGCTCTCCAGGAGTCCGTCGAGTCTTACCTCGTCTCCCTCTTCGAGGACACCAACCTGTGCGCCATCCACGCCAAGCGTGTCACCATCCAGTCGGTACGTC

>Diaporthe_pachirae_CDA_728

?????????????????????????????????????????????????????????????????????????????????????????????????????????????????????????????????????????????????????????????????????????????????????????????????????????????????????????????????????????????????????????????????????????????????????????????????????????????????????????????????????????????????????????????????????????????????????

>Diaporthe_passifloricola_CBS_141329

TCCGCGCCCTCCACCGGAGGTGTCAAGAAGCCTCACCGCTACAAGCCCGGTACCGTCGCTCTGCGTGAGATCCGTCGCTACCAGAAGAGCACCGAGCTGCTGATCCGCAAGCTCCCCTTCCAGCGTCTGGTATG----------------------------------------------------------------------------CAGGTCCGTGAGATCGCCCAGGACTTCAAGTCCGACCTGCGCTTCCAGTCTTCCGCCATCGGTGCTCTCCAGGAGTCCGTCGAGTCTTACCTCGTCTCCCTCTTCGAGGACACCAACCTGTGCGCCATCCACGCCAAG?????????????????????????

>Diaporthe_pseudolongicolla_CBS_117165

?????????????????????????????????????????????????????????????????????????????????????????????????????????????????????????????????????????????????????????????????????????????????????????????????????????????????????????????????????????????????????????????????????????????????????????????????????????????????????????????????????????????????????????????????????????????????????

>Diaporthe_pyracanthae_CBS142384

TCCGCGCCCTCCACCGGAGGTGTCAAGAAGCCTCACCGCTACAAGCCTGGTACCGTCGCTCTGCGTGAGATCCGTCGTTACCAGAAGAGCACTGAGCTGCTGATCCGCAAGCTCCCCTTCCAGCGTCTGGTATG----------------------------------------------------------------------------CAGGTTCGTGAGATCGCCCAGGACTTCAAGTCCGACCTCCGCTTCCAGTCCTCCGCCATCGGTGCCCTGCAGGAGTCCGTCGAGTCTTACCTCGTCTCCCTGTTCGAGGACACCAACTTGTGCGCCATCCACGCCAAGCGTGTCACCATCCAGTCGGTACGTT

>Diaporthe_racemosae_CBS_143770

TCCGCGCCCTCCACCGGAGGTGTCAAGAAGCCTCACCGCTACAAGCCTGGTACCGTCGCTCTGCGTGAGATCCGTCGCTACCAGAAGAGCACCGAGCTGCTGATCCGCAAGCTCCCCTTCCAGCGTCTGGTATG----------------------------------------------------------------------------TAGGTCCGTGAGATCGCCCAGGACTTCAAGTCCGACCTGCGCTTCCAGTCTTCTGCCATCGGTGCCCTCCAGGAGTCTGTCGAGTCTTACCTCGTCTCCCTCTTTGAGGACACCAACCTGTGCGCCATCCACGCCAAGCGTGTCACCATCCAGTCGGTACGTA

>Diaporthe_raonikayaporum_CBS_133182

?????????????????????????????????????????????????????????????????????????????????????????????????????????????????????????????????????????????????????????????????????????????????????????????????????????????????????????????????????????????????????????????????????????????????????????????????????????????????????????????????????????????????????????????????????????????????????

>Diaporthe_rosae_MFLUCC_17_2658

?????????????????????????????????????????????????????????????????????????????????????????????????????????????????????????????????????????????????????????????????????????????????????????????????????????????????????????????????????????????????????????????????????????????????????????????????????????????????????????????????????????????????????????????????????????????????????

>Diaporthe_rosiphthora_COAD_2913

?????????????????????????????????????????????????????????????????????????????????????????????????????????????????????????????????????????????????????????????????????????????????????????????????????????????????????????????????????????????????????????????????????????????????????????????????????????????????????????????????????????????????????????????????????????????????????

>Diaporthe_rossmaniae_CAA762

TCCGCGCCCTCCACCGGAGGTGTCAAGAAGCCTCACCGCTACAAGCCTGGTACCGTCGCTCTGCGTGAGATCCGTCGCTACCAGAAGAGCACCGAGCTGCTGATCCGCAAGCTGCCCTTCCAGCGTCTGGTATGTCT-GCACCTGCCCAAACT-------------CACTCGCCCTGCTCCCCCTGCTGACCGTCG-CCCGCTTTCCCTCTAGGTCCGTGAGATCGCCCAGGACTTCAAGTCCGACCTGCGCTTCCAGTCTTCCGCCATCGGTGCCCTTCAGGAGTCCGTCGAGTCTTACCTCGTCTCCCTCTTTGAGGACACCAACCTGTGCGCCATCCACGCCAAGCGTGTCACCATCCAGTCGGTACGTC

>Diaporthe_sackstonii_BRIP_54669b

?????????????????????????????????????????????????????????????????????????????????????????????????????????????????????????????????????????????????????????????????????????????????????????????????????????????????????????????????????????????????????????????????????????????????????????????????????????????????????????????????????????????????????????????????????????????????????

>Diaporthe_schini_CBS_133181

TCCGCGCCCTCCACCGGAGGTGTCAAGAAGCCTCACCGCTACAAGCCTGGTACCGTCGCTCTGCGTGAGATCCGTCGCTACCAGAAGAGCACCGAGCTGCTGATCCGCAAGCTCCCCTTCCAGCGTCTGGTATG----------------------------------------------------------------------------CAGGTCCGTGAGATCGCCCAGGACTTCAAGTCCGACCTGCGCTTCCAGTCTTCCGCCATCGGTGCCCTTCAGGAGTCCGTCGAGTCTTACCTCGTCTCCCTCTTTGAGGACACCAACCTGTGCGCCATCCACGCCAAGCGTGTCACCATCCAGTCGGTACGTC

>Diaporthe_schoeni_MFLU_15_1279

?????????????????????????????????????????????????????????????????????????????????????????????????????????????????????????????????????????????????????????????????????????????????????????????????????????????????????????????????????????????????????????????????????????????????????????????????????????????????????????????????????????????????????????????????????????????????????

>Diaporthe_sclerotioides_CBS_296_67

TCCGCGCCTTCCACCGGAGGTGTCAAGAAGCCTCACCGCTACAAGCCCGGTACCGTCGCTCTGCGTGAGATCCGTCGCTACCAGAAGAGTACCGAGCTGCTGATCCGCAAGCTCCCCTTCCAGCGTCTTGTATG----------------------------------------------------------------------------CAGGTTCGTGAGATCGCCCAGGACTTCAAGTCCGACCTGCGCTTCCAGTCTTCCGCCATCGGTGCCCTGCAGGAGTCTGTCGAGTCTTACCTCGTCTCTCTCTTCGAGGACACCAACCTGTGCGCCATCCACGCCAAGCGTGTCACCATCCAGTCGGTACGTT

>Diaporthe_serafiniae_BRIP_55665a

?????????????????????????????????????????????????????????????????????????????????????????????????????????????????????????????????????????????????????????????????????????????????????????????????????????????????????????????????????????????????????????????????????????????????????????????????????????????????????????????????????????????????????????????????????????????????????

>Diaporthe_siamensis_MFLUCC_10_0573a

?????????????????????????????????????????????????????????????????????????????????????????????????????????????????????????????????????????????????????????????????????????????????????????????????????????????????????????????????????????????????????????????????????????????????????????????????????????????????????????????????????????????????????????????????????????????????????

>Diaporthe_sinensis_ZJUP0033_4

?????????????????????????????????????????????????????????????????????????????????????????????????????????????????????????????????????????????????????????????????????????????????????????????????????????????????????????????????????????????????????????????????????????????????????????????????????????????????????????????????????????????????????????????????????????????????????

>Diaporthe_stewartii_CBS_193_36

?????????????????????????????????????????????????????????????????????????????????????????????????????????????????????????????????????????????????????????????????????????????????????????????????????????????????????????????????????????????????????????????????????????????????????????????????????????????????????????????????????????????????????????????????????????????????????

>Diaporthe_subordinaria_CBS_101711

TCCGCGCCCTCCACCGGAGGTGTCAAGAAGCCTCACCGCTACAAGCCTGGTACCGTCGCTCTGCGTGAGATCCGTCGCTACCAGAAGAGCACCGAGCTGCTCATCCGCAAGCTGCCCTTCCAGCGTCTGGTAAG----------------------------------------------------------------------------CAGGTTCGTGAGATCGCCCAGGACTTCAAGTCCGACCTGCGCTTCCAGTCTTCCGCCATCGGCGCCCTCCAGGAGTCCGTCGAGTCCTACCTCGTCTCCCTCTTCGAGGACACCAACCTGTGCGCCATCCACGCGAAGCGTGTCACCATCCAGTCGGTACGTC

>Diaporthe_tecomae_CBS_100547

TCCGCGCCTTCCACCGGAGGTGTCAAGAAGCCTCACCGCTACAAGCCTGGTACTGTTGCTCTGCGTGAGATCCGTCGCTACCAGAAGAGCACCGAGCTGCTGATCCGCAAGCTCCCTTTCCAGCGTCTGGTATG----------------------------------------------------------------------------CAGGTCCGTGAGATCGCCCAGGACTTCAAGTCCGACCTGCGCTTCCAGTCCTCCGCCATCGGTGCCCTTCAGGAGTCCGTCGAGTCTTACCTCGTCTCCCTCTTTGAGGACACCAACCTGTGCGCCATCCACGCCAAGCGTGTCACCATCCAGTCGGTACGTC

>Diaporthe_tectonae_MFLUCC_12_0777

?????????????????????????????????????????????????????????????????????????????????????????????????????????????????????????????????????????????????????????????????????????????????????????????????????????????????????????????????????????????????????????????????????????????????????????????????????????????????????????????????????????????????????????????????????????????????????

>Diaporthe_tectonendophytica_MFLUCC_13_0471

?????????????????????????????????????????????????????????????????????????????????????????????????????????????????????????????????????????????????????????????????????????????????????????????????????????????????????????????????????????????????????????????????????????????????????????????????????????????????????????????????????????????????????????????????????????????????????

>Diaporthe_terebinthifolii_CBS_133180

TCCGCGCCCTCCACCGGAGGTGTCAAGAAGCCTCACCGCTACAAGCCTGGTACCGTCGCTCTGCGTGAGATCCGTCGCTACCAGAAGAGCACCGAGCTGCTGATCCGCAAGCTCCCCTTCCAGCGTCTGGTATG----------------------------------------------------------------------------CAGGTCCGTGAGATCGCCCAGGACTTCAAGTCCGACCTGCGCTTCCAGTCTTCCGCCATCGGTGCCCTTCAGGAGTCCGTCGAGTCTTACCTCGTCTCCCTCTTTGAGGACACCAACCTGTGCGCCATCCACGCCAAGCGTGTCACCATCCAGTCGGTACGTC

>Diaporthe_thunbergiicola_MFLUCC_12_0033

?????????????????????????????????????????????????????????????????????????????????????????????????????????????????????????????????????????????????????????????????????????????????????????????????????????????????????????????????????????????????????????????????????????????????????????????????????????????????????????????????????????????????????????????????????????????????????

>Diaporthe_tulliensis_BRIP_62248a

?????????????????????????????????????????????????????????????????????????????????????????????????????????????????????????????????????????????????????????????????????????????????????????????????????????????????????????????????????????????????????????????????????????????????????????????????????????????????????????????????????????????????????????????????????????????????????

>Diaporthe_ueckerae_FAU_656

TCCGCGCCCTCCACCGGAGGTGTCAAGAAGCCTCACCGCTACAAGCCCGGTACCGTCGCTCTGCGTGAGATCCGTCGCTACCAGAAGAGCACCGAGCTGCTGATCCGCAAGCTCCCCTTCCAGCGTCTGGTATG----------------------------------------------------------------------------CAGGTCCGTGAGATCGCCCAGGACTTCAAGTCCGACCTGCGCTTCCAGTCTTCCGCCATCGGTGCTCTCCAGGAGTCCGTCGAGTCTTACCTCGTCTCCCTCTTTGAGGACACCAACCTGTGCGCCATCCACGCCAAGCGTGTCACCATCCAGTCGGTACGTC

>Diaporthe_unshiuensis_CGMCC3_17569

TCCGCGCCCTCCACCGGAGGTGTCAAGAAGCCTCACCGCTACAAGCCTGGTACCGTCGCTCTGCGTGAGATCCGTCGCTACCAGAAGAGCACCGAGCTGCTGATCCGCAAGCTCCCCTTCCAGCGTCTGGTATG----------------------------------------------------------------------------CAGGTCCGTGAGATCGCCCAGGACTTCAAGTCCGACCTGCGCTTCCAGTCTTCCGCCATCGGTGCCCTGCAGGAGTCCGTCGAGTCTTACCTCGTCTCCCTCTTTGAGGACACCAACCTGTGCGCCATCCACGCCAAGCGTGTCACCATCCAGTCGGTACGTC

>Diaporthe_vexans_CBS_127_14

TCCGCGCCCTCCACCGGAGGTGTCAAGAAGCCTCACCGCTACAAGCCTGGTACCGTCGCTCTGCGTGAGATCCGTCGCTACCAGAAGAGCACTGAGCTGCTGATCCGCAAGCTCCCCTTCCAGCGTCTGGTATG----------------------------------------------------------------------------TAGGTCCGTGAGATCGCCCAGGACTTCAAGTCCGACCTGCGCTTCCAGTCCTCCGCCATCGGTGCCCTTCAGGAGTCGGTCGAGTCCTACCTCGTCTCCCTCTTTGAGGACACCAACCTGTGCGCCATCCACGCCAAGCGTGTCACCATCCAGTCGGTACGTC

>Diaporthe_yunnanensis_CGMCC_3_18289

TCCGCGCCCTCCACCGGAGGTGTCAAGAAGCCTCACCGCTACAAGCCTGGTACCGTCGCTCTGCGTGAGATCCGTCGTTACCAGAAGAGCACCGAGCTGCTGATCCGCAAGCTCCCCTTCCAGCGTCTGGTATG----------------------------------------------------------------------------CAGGTTCGTGAGATCGCCCAGGACTTCAAGTCCGACCTGCGCTTCCAGTCTTCCGCCATCGGTGCCCTGCAGGAGTCCGTCGAGTCTTACCTCGTCTCCCTCTTTGAGGACACCAACCTGTGCGCCATCCACGCCAAGCGTGTCACCATCCAGTCGGTACGTC

>Phomopsis_glabrae_SCHM_3622

?????????????????????????????????????????????????????????????????????????????????????????????????????????????????????????????????????????????????????????????????????????????????????????????????????????????????????????????????????????????????????????????????????????????????????????????????????????????????????????????????????????????????????????????????????????????????????

>Phomopsis_micheliae

?????????????????????????????????????????????????????????????????????????????????????????????????????????????????????????????????????????????????????????????????????????????????????????????????????????????????????????????????????????????????????????????????????????????????????????????????????????????????????????????????????????????????????????????????????????????????????

>Phomopsis_vitimegaspora_STE_U2675

?????????????????????????????????????????????????????????????????????????????????????????????????????????????????????????????????????????????????????????????????????????????????????????????????????????????????????????????????????????????????????????????????????????????????????????????????????????????????????????????????????????????????????????????????????????????????????

>Diaporthe_subellipicola_KUMCC_17_0153

?????????????????????????????????????????????????????????????????????????????????????????????????????????????????????????????????????????????????????????????????????????????????????????????????????????????????????????????????????????????????????????????????????????????????????????????????????????????????????????????????????????????????????????????????????????????????????

>Diaporthe_masirevicii_BRIP_57892a

?????????????????????????????????????????????????????????????????????????????????????????????????????????????????????????????????????????????????????????????????????????????????????????????????????????????????????????????????????????????????????????????????????????????????????????????????????????????????????????????????????????????????????????????????????????????????????

>Diaporthe_sojae_CBS_139282

TCCGCGCCCTCCACCGGAGGTGTCAAGAAGCCTCACCGCTACAAGCCTGGTACCGTCGCTCTGCGTGAGATCCGTCGCTACCAGAAGAGCACCGAGCTGCTGATCCGCAAGCTCCCCTTCCAGCGTCTGGTATG----------------------------------------------------------------------------CAGGTCCGTGAGATCGCCCAGGACTTCAAGTCCGACCTGCGCTTCCAGTCTTCCGCCATCGGTGCTCTCCAGGAGTCCGTCGAGTCTTACCTCGTGTCCCTCTTCGAGGACACCAACCTGTGCGCCATCCACGCCAAGCGTGTCACCATCCAGTCGGTACGTC

>Diaporthe_sambucusii_CFCC_51986

TCCGCGCCCTCCACCGGAGGTGTCAAGAAGCCTCACCGCTACAAGCCTGGTACCGTCGCCCTGCGTGAGATCCGTCGCTACCAGAAGAGCACCGAGCTGCTCATCCGCAAGCTCCCCTTCCAGCGTCTGGTATGTTT--GCGCCTGCCAATCGGCCT-----CCACCCCGCTCCTGCTCCCGTCCCCTTGCTGACAGCCGTCCCCTCTTGCAGGTCCGTGAGATCGCCCAGGACTTCAAGTCCGACCTGCGCTTCCAGTCTTCCGCCATCGGTGCCCTTCAGGAGTCCGTCGAGTCCTACCTCGTCTCCCTCTTCGAGGACACCAACCTGTGCGCCATCCACGCCAAGCGTGTCACCATCCAGTCGGTACGTC

>Diaporthe_vochysiae_LGMF1583

TCCGCGCCCTCCACCGGAGGTGTCAAGAAGCCTCACCGCTACAAGCCCGGTACCGTCGCTCTGCGTGAGATCCGTCGCTACCAGAAGAGCACCGAGCTGCTGATCCGCAAGCTCCCCTTCCAGCGTCTGGTATGTTCTGCACCCCACCAACCATCCTCGCTGTCGCCCTCACCCTCGTACACCCTGCTGACTGTCG--CGCCTCCCTCTCCAGGTCCGTGAGATCGCCCAGGACTTCAAGTCCGACCTGCGCTTCCAGTCTTCCGCCATCGGTGCTCTCCAGGAGTCCGTCGAGTCTTACCTCGTCTCCCTCTTCGAGGACACCAACCTGTGCGCCATCCACGCCAAGCGTGTCACCATCCAGTCGGTACGTC

>Diaporthe_endophytica_CBS_133811

TCCGCGCCCTCCACCGGAGGTGTCAAGAAGCCTCACCGCTACAAGCCTGGTACCGTCGCTCTGCGTGAGATCCGTCGCTACCAGAAGAGCACCGAGCTGCTGATCCGCAAGCTCCCCTTCCAGCGTCTGGTATG----------------------------------------------------------------------------CAGGTCCGTGAGATCGCCCAGGACTTCAAGTCCGACCTGCGCTTCCAGTCTTCCGCCATCGGTGCTCTCCAGGAGTCCGTCGAGTCTTACCTCGTCTCCCTCTTTGAGGACACCAACCTGTGCGCCATCCACGCCAAGCGTGTCACCATCCAGTCGGTACGTC

>Diaporthe_phaseolorum_CBS_113425

TCCGCGCCCTCCACCGGAGGTGTCAAGAAGCCTCACCGCTACAAGCCTGGTACCGTCGCTCTGCGTGAGATCCGTCGCTACCAGAAGAGCACCGAGCTGCTGATCCGCAAGCTCCCCTTCCAGCGTCTGGTATG----------------------------------------------------------------------------CAGGTCCGTGAGATCGCCCAGGACTTCAAGTCCGACCTGCGCTTCCAGTCTTCCGCCATCGGTGCTCTCCAGGAGTCCGTCGAGTCTTACCTCGTGTCCCTCTTCGAGGACACCAACCTGTGCGCCATCCACGCCAAGCGTGTCACCATCCAGTCGGTACGTC

>Diaporthe_caliensis_STMA_22040

TCCGCGCCCTCCACCGGAGGTGTCAAGAAGCCTCACCGCTACAAGCCTGGTACCGTCGCTCTGCGTGAGATCCGTCGCTACCAGAAGAGCACCGAGCTGCTGATCCGCAAGCTCCCCTTCCAGCGTCTGGTATGTTTTCACAYCCACCCAAATCAATCAACTTCACCCTCGTTTACCCTGCTGACCGTCGCCTCTT----CCTCCC----CAGGTCCGTGAGATCGCCCAGGACTTCAAGTCCGACCTGCGCTTMCAGTCTTCCGCCATCGGTGCTCTCCAGGAGTCCGTCGAGTCTTACCTCGTGTCCCTCTTCGAGGACACCAACCTGTGCGCCATCCACGCCAAGCGTGTCACCATCCAGTCGGTACGTC

**Alignment of the *tef1* sequences used in the phylogenetic study**

>Diaporthe_acaciarum_CBS_138862

????????????????????????????????????????????????????????????????????????????????????????????????????????????????????????????????????????????????????????????????????????????????????????????????????????????????????????????????????????????????????????????????????????????????????????????????????????????????????????????????????????????????????????????????????????????????????????????????????????????????????????????????????????????????????????????????????

>Diaporthe_acericola_MFLUCC_17_0956

NNNNNNNNNNNNNNNNNNNNNNNNNNNNNNNNNNNNNNNNNNNNNNNGGAT--GACT--CCA-C-CAAG---CCCACCGTGCGCAC-------ATCGTCATCATTG--GTGCG----CGAC-TGCCTGCGCGGCTTCAT--CACCC----CTCAAACCA-TTTTCACCCCTCCCTCTGGGTTTTTTTTTCATT---TTCAGTGC-------------------GGGTGCGGGGCGCGCTTATCA-G----CCGCTTATCTCCT--A--TGCAAAA---------CCCTGCT---GGCATTA--ACCACTCCTT--------GCCACCG-CCAACACCAACTCTACCACTTTCAACCCTATCAATCTCCCAGGCGAAACG---AACTGTCAA--------GAATATGAAGCTGACTTAC----TTTCCACACAGCCGCCGAGCTGGGTAAGGG

>Diaporthe_alangii_CFCC_52556

GAGAAGGAAGGTGAGTAAACAT--CAAAA----------GTCTACGGCATA--CACT--CCCAT-TG-----TACCCCAGTTGCAC-------ATCAGGATTACTG--GCGCG----CTGC-CGTTTGCGCGGCTTCGTCACACCC----GCCAGGGCA-TTTTCACCCCTCCCTCTGGA----TTTTCCATT---TTCAGTGC-------------------GGGTGCGGGGTGCGCTTATCAGG----CGGCTTATCTCTGACG-CTCACAAA---------CCCTGCT----GCATCA--ACCATTCCTT--------GTCGCTG-CCACCACCACCATCACCGTCAAATTCATCGCACGGTCAAATTGATCGGATGACTTGTGTCTTGGAGA---AATCATGAAGCTGACTTTC----CATCTCTATAGCCGCCGAGCTCGGTAAGGG

>Diaporthe_ambigua_CBS_114015

GAGAAGGAAGGTCAGTAAACAT--CGAAT----------GCCTACGGCATA--CACC--CCCAT-TG-----CCTTTCATATGCAC-------TTCAGAATCACTT--GTGCG----CGGC-CGTTTGCGCGGCTTCGCCACACCC----TTCAAGGCATTTTTCACCCCTCCCTCTGGA----TTTTCCATT---TTCAGTGC-------------------GGGTGCGGGGTGTGCTTATCAGG----CGGCTTATCTCTTACA-CTCACAAACC-------CCTTGTT----GCATCA-ACCCACTCGGT--------GCTGCTG-TTATCACC--------------------------------------------------------------GAACATCATGCTGACCTCTT---TTCCCACACAGCCGCCGAGCTGGGTAAGGG

>Diaporthe_amygdali_CBS_126679

GAGAAGGAAGGTTAGTAAACATCAACCTCTAC-------AATCGCAACACA--TTCT--GCA-T-GC-----CCCTTCACATGCTCGACT---TCATAAATCCTGG--GCGTG----CGCC-CGTTCCCACAGCGTCATCACACCT----GGGGGCGCA-TTTTCACCCCTCGCTCTGGA----TTTTCCATT---TTCAGTGC-------------------GGGTGCGGGGTGCGCTTATCAGC----GAGCTTATCTCCCACC---CGAAAA---------CCCTGCA--------------CACACTAC--------ATCACTACATACCACC--------------------------------------------------------------TTGGATCATGCTGACTTCC----CATCTTCACAGCCGCTGAGCTCGGTAAGGG

>Diaporthe_angelicae_CBS_111592

GAGAAGGAAGGTCAGTAAACAT--CACACATCCAACCCTTCCCACAGCATC--AACT--CCA-C--AAG---TCCTCCATGCGCAC-------ATTGTCATCATTG--GTGCG----CGAC-TGCCTGCGCGGCTTGGTCACACCC----GTCAAGCCA-TTTTCACCCCT-CCTCTGGG--TTTTTTCCATT---TTCAGTGC-------------------GGGTGCGGGGTGCGGTTATCAGG----CCGCTTATCTCCCGCA--CGCAAAA---------CCCTGCT---GGCATTA--ACCACTCCCT--------ACCACCG-TCAACACC--------------------------------------------------------------GAACATGACGCTGACTTAA----TTTCCACACAGCCGCCGAGCTGGGCAAGGG

>Diaporthe_arctii_CBS_136_25

GAGAAGGAAGGTCAGTAAACAT--CACACATCCAACCCTTCCCACAGCATC--AACT--CCA-C-AAAG---CCCTCCATGCGCAC-------ATTGTCATCATTG--GTGCG----CGAC-TGCCTGCGCGGCTTCGTCACACCC----GTCAAGCCA-TTTTCACCCCTCCCTCTGGG--TTTTTTCCATT---TTCAGTGC-------------------GGGTGCGGGGTGCGCTTATCAGG----CCGCTTATCTCCCACA--CGCAAAA---------CCCTGCT---GGCATTA--ACCACTCCCT--------ACCACCG-TCAACACC--------------------------------------------------------------GAACATGACGCTGACTTGA----TTTCCATACAGCCGCCGAGCTGGGCAAGGG

>Diaporthe_arezzoensis_MFLU_19_2880

????????????????????????????????????????????????????????????????????????????????????????????????????????????????????????????????????????????????????????????????????????????????????????????????????????????????????????????????????????????????????????????????????????????????????????????????????????????????????????????????????????????????????????????????????????????????????????????????????????????????????????????????????????????????????????????????????

>Diaporthe_batatas_CBS_122_21

?????????????????????????CATCA---------GAACCCAGCTTG--ACCA--CTA-C-GT-----CCCTCCACATGCAC-------TCAGAAATCCTTG--GCCCG----CG-C-TGTCTGTGACGCTTCGTCACACCT----GTGAAAGCA-TTTTCACCCCTCCCTCTGGA----TTTTCCATT---TTCAGTGC-------------------GGGTGCGGGGTGCGCTTATCAGG----CCGCTTATCTCCCA---CACCAAAA---------CCCTGTC----GCACCC--ACCACTCTTTG-------ACCAGTG-ACATTCC---------------------------------------------------------------GGAAACCATGCTGACTC------TCTCTACACAGCCGCCGAGCTGGGTAAGGG

>Diaporthe_beilharziae_BRIP_54792

NNGAAGGAAGGTCAGTTAATAT--CACACA---------TGCCGCATCATG--AACC--CCA-T-GAAGC--CCCCCCACACGCAC-------ATCCTCACCATGG--GCGCG----CGAC-TGTTTGCGCGGCTGCGTCACACCC----GTCAAGCCA-TTTTCACCCCTCCCTCTGGA---TTTTTCCATT---TTCAGTGC-------------------GGGTGCGGGGTGCGCTTATCAGG----CCGCTTATCTTCCACA--CGCAAAA---------CCCTGCT---GGCGTCA--ATCACTTCCT--------TGCACTG-CCAGCACC--------------------------------------------------------------GACAATCATACTGACTTAC----TTCCCACACAGCCGCCGAGCTGGGCAAGGG

>Diaporthe_biguttulata_ICMP20657

GAGAAGGAAGGTTAGTAAACAT--CATGA----------GTACACAGCATA--AACT--CCC-T-TG------CGTCCGTATGCAC-------CTCCGAATTATCG--GCGCG----CGGC-CATCTGCGCGGCTTCGTCACATCT----GTGAAGGCA-TTTTCACCCCTCGCTCTGGA----TTTTCCATT---TTCAGTGC-------------------GGGTGCGGGGTGCGCTTATCAGG----CGGCTTATCTCTTACA--CCCAAAA---------CCCTGTT----GCATCC--ACCACTCCAT--------GCCGC-G-CTACCACC--------------------------------------------------------------GGAACATATGCTGACCATC----TTTCTACACAGCCGCCGAGCTTGGTAAGGG

>Diaporthe_breyniae

GAGAAGGAAGGTTAGTAAACAT--CACTGC---------ATGTGCAGCTTG--CGCT--CCG-T-AT-----CCCTCCATATGCAC-------ATGGGAGTCGTTG--CCGTG----CG-C-TGTCTGCGACGCTTCGTCACACCT----GTCAAGGCA-TTTTCACCCCTCCCTCTGGA----TTTTCCATT---TTCAGTGC-------------------GGGTGCGGGGTGCGCTTATCAGG----CCGCTTATCTCACA---CATCAA-----------CCCTGTC----GCACCTCTACCACTGTTC--------ACCAGTG-ACACACCAATAGCATCACTTTCATTCCCA--TTTGTTGCTCCAGGAAGCTTTTTGGATCGTTC-------GAAAATCATGCTGACTC------TTTCTACACAGCCGCCGAGCTGGGTAAGG?

>Diaporthe_camporesii_JZB320143

????????????????????????????????????????????????????????????????????????????????????????????????????????????????????????????????????????????????????????????????????????????????????????????????????????????????????????????????????????????????????????????????????????????????????????????????????????????????????????????????????????????????????????????????????????????????????????????????????????????????????????????????????????????????????????????????????

>Diaporthe_caryae_CFCC_52563

GAGAAGGAAGGTCAGTAAACAT--TACACA---------TCCCGCATCATG--AACC--CCA-T-GAAC---TCCGCCTGACGCAC-------ATCGTCACCATGG--GCGCG----CGAC-TTTTTGCACGGCTGCGTCACACCC----GTCAAGCCA-TTTTCGCCCCTCCCTCTGGA---TTTTTCCATT---TTCAGTGC-------------------GGGTGCGGGGTGTGCTTATCAGG----CCGCTTATCTCCCACA--CGCAAAA---------CCCTGCT---GGCATCA--ATCACTTCCT--------TGCACTG-CCTGCTCCAACATCACAGGCATCAAACACCATCAACCTCTACTTGCGCAACA-AACTGTTCG--------GACAATCATGCTGACTTAC----ATCCCACACAGCCGCCGAGCTGGGTAAGGG

>Diaporthe_celtidis_NCYU_19_0357

GAGAAGGAAGGTTAGTAAACAT--CAAAA----------GTCTACGGCACA--CACT--CCCAT-TG-----TACCCCATTTGCAC-------ATCAGGATTACTG--GCGCG----CTGC-CGTTTGCGCGGCTTCGTCACACCC----GCCAGGGCA-TTTTCACCCCTCCCTTTGGA----TTTTCCATT---TTCAGTGC-------------------GGGTGCGGGGTGCGCTTATCAGG----CGGCTTA--TCTGACG-CTCACAAA---------CCCTGCT----GCATCA--ACCATTCCTT--------GTCGCTG-CCACCACCACCATCACCGTCAAATTCATCGCACTGTCAAATTGATCGGATGACTTGTGTCTTGGAGG---AATCATGAAGCTGACTTTT----CATCTCTATAGCCGCCGAGCTCGGTAAGGG

>Diaporthe_cerradensis_CMRP4331

NNNNNNNNNNNNNNNNNNNNNNNNNNNNNNNNNNNNNNNNNNNACAGCTTA--CACT--C------------TGCCCTCTATCCCT-------CAGAGAATCGTTG--GCGCA----CGGC-CGTCTGCGCGGCTTCGT--CACCC----GTGAAGGCA-TTTTCACCCCTCCCTCTGGA----TTTTCCATT---TTCAGTGC-------------------GGGTGCGGGGTGCGCTTATCAGG----CCGCTTATCTCTGACC--TTTACAA---------CCCTGGC----GCATCA--ACCACTCATT--------ACCGCTG-CTACCACCAACAACATCACTGCCGATTCCACTGACGTCTCCCGCCAAAACAACCTGATGAGCCCTGCTGGAAAATTCACGCTGACCTTT----TTTCTACACAGCCGCCGAGCTGGGTAAGGG

>Diaporthe_chimonanthi

????????????????????????????????????????????????????????????????????????????????????????????????????????????????????????????????????????????????????????????????????????????????????????????????????????????????????????????????????????????????????????????????????????????????????????????????????????????????????????????????????????????????????????????????????????????????????????????????????????????????????????????????????????????????????????????????????

>Diaporthe_chinensis_MFLUCC_19_0101

NNNNNNNNNNNNNNNNNNNNNNNNNNNNNNNNNNNNNNNNNNNNNNNNNNNNNNNNNNNNNC-T-TG-----CCCTCCATGTGCAT-------CTCAGAATCATTG--GCGCG----TGGC-CATCCGCGAGGCTTCGTCACATCC----GTCAAGGCA-TTTTCACCCCTCGCTCTGGA----TTTTCCATT---TTCAGTGC-------------------GGGTGCGGGGTGTGCTTATCAGG----CGGCTTATCTCTTACA--TCTACAA---------CCCTGTT----GCATCA--ACCACTCCAT--------CCCGCTG-CCTCCTCCAACACCATCACTATCATCTTTCAGCTAAAAAAAA--AACCTGATGAAATGCGCC--------GGAATTCATGCTGACCATT-GTTTTTAAAAACAGCCGCCGAGCTTGGTAANNN

>Diaporthe_chromolaenae_MFLUCC_17_1422

????????????????????????????????????????????????????????????????????????????????????????????????????????????????????????????????????????????????????????????????????????????????????????????????????????????????????????????????????????????????????????????????????????????????????????????????????????????????????????????????????????????????????????????????????????????????????????????????????????????????????????????????????????????????????????????????????

>Diaporthe_cichorii_MFLUCC_17_1023

NNNNNNNNNNNNNNNNNNNNNNNNNNNNNNNNNNNNNNNNNNNNNNGTATC--AACC--CCA-C-AAAG---TCCTCCATGCGCAC-------ATTGTCCTGATTG--GTGCG----CGAC-TGCCTGCGCGGCTTCGTCGCACCC----GTCAAGCCA-TTTTCACCCCTCCCTCTGGG--TTTTTTCCATT---TTCAGTGC-------------------GGGTGCGGGGTGCGCTTATCAGG----CCGCTTATCTCCAACA--CGCAAAA---------CCCTGCT---GGCATGA--ACCACTCCCT--------ACCACCG-TCAACACCAACTCTACCACTTTGAACCCTATCAAACACCCTCCTGCCGAAAAGATCTGTCAA--------GAACATGACGCTGACTTAC----TTTCCACACAGCTGCCGAGCTGGGTAAGGG

>Diaporthe_cinnamomi_CFCC_52569

GAGAAGGAAGGTTAGTAAACAT--CATGA----------GCTCGCAGCATA--CACT--CTC-T-TG-----CCCCCCATATGCAT-------CTCGAAGTCATTG--GCGCG----CGAC-CATCTGCGCGGCTTCGTCACATCC----GTAAAGGCA-TTTTCACCCCTCGCTCTGGA---TTTTTCCATT---TTCAGTGC-------------------GGGTGCGGGGTGCGCTTATCAGG----CGGCTTATCTCTTACA-TCACAAAA---------CCCTGTT----GCATCA--CCCACTCCAT--------CCCGCTG-CTAGTACCAACACCATCACTATCATCTTTGAGCTGGAAACAG--AACCTGATGAATTGCGCT--------GGAATCTATGCTGACCATT----TTCCTACACAGCCGCCGAGCTTGGTAAGGG

>Diaporthe_citriasiana_CBS_134240

GAGAAGGAAGGTTAGTAAACAT--CATGA----------GTTCGCAGCATA--CACT--TCC-T-TG-----CCCTCCATATGCAT-------CTCAGAATCATTG--GCGCG----CGGC-CATCTGCGCGGCTTCGTCACATCC----GTCAAGGCA-TTTTCACCCCTCGCTCTGGA----TTTTCCATT---TTCAGTGC-------------------GGGTGCGGGGTGCGCTTATCAGG----CGGCTTATCTCTTACA--TCCACAA---------CCCTGTT----GCATCA--ACCACTCCAT--------CCCGCTG-CCACCTCC--------------------------------------------------------------GGAATTTATGCTGACCATT----TTTCTACACAGCCGCCGAGCTTGGTAAGGG

>Diaporthe_compacta_LC3083

GAGAAGGAAGGTCAGTAAACAT--AACAAA---------CTCCACAACATC--AACC--CCAA--AGTC---ATCCCAGTGCACAT-------CGCGGGATCATCG--GCGCG----CGAC-CGTCTGCGCCGCTTCGT--CACTC----GTCAGAGCA-TTTTCACCCCTCGTTCTGGA----TTTTCCACT---TTCAGTGC-------------------GGGTGCGGGGTGTGCTTATCAGG----CCGCTTATCGCTTGCG-TTCCAAAA---------CCCTGCTGGGGGCATCA--ACCACTCTTT--------GCCGCTG-CCCACATT--------------------------------------------------------------GAAAACAATGCTGACTCGC----TTTCCATACAGCCGCCGNNNNNNNNNNNNN

>Diaporthe_convolvuli_CBS_124654

GAGAAGGAAGGTTAGTAAACAT---ACTAA---------ATGGGTGGCCTG--ACCT--CCA-T-AT-----CCCTCCACATGAAC---------GGAAATCGTTG--GCGCG----CG-C-TGTCTGCGAGGCTTCGTCACACCT----GTCAAGCCA-TTTTCACCCCTCCCTCTGGA----TTTTCCATT---TTCAGTGC-------------------GGGTGCGGGGTGCGCTTATCAGG----CCGCTTATCTCTCACA-CACCAAAG---------CCCTGTC----GCACCTCATCACTCTTTT--------ACCAGCT-ACACACC---------------------------------------------------------------GAAAATCATGCTGACTCTG----TTTCTACACAGCCGCCGAGCTGGGTAAGGG

>Diaporthe_cucurbitae_DAOM_42078

GAGAAGGAAGGTCAGTAAACAT--CACACATCCAACCCTTCCCACAGCATC--AACT--CCA-C-AAAG---CCCTCCATGCGCAC-------ATTGTCATCATTG--GTGCG----CGAC-TGCCTGCGCGGCTTCGTCACACCC----GTCAAGCCA-TTTTCACCCCTCCCTCTGGG--TTTTTTCCATT---TTCAGTGC-------------------GGGTGCGGGGTGCGCTTATCAGG----CCGCTTATCTCCCACA--CGCAAAA---------CCCTGCT---GGCATTA--ACCACTCCCT--------ACCACCG-TCAACACC--------------------------------------------------------------GAACATGACGCTGACTTAA----TTTCCATACAGCCGCCGAGCTGGGTAAGGG

>Diaporthe_cuppatea_CBS_117499

NNNNNNNNNNGTCAGTAAACAT--CACACA---------TCCCACAGCATG--AACT--CCA-C-AAAG---TCCTCCATGCGCAC-------ATTGCCATCATAG--GTGCG----CGAC-TGTCTGCGCGGCTTCGTCACACCC----GTCAAGCCA-TTTTCACCCCTCCCTCTGGG--TTTTTTCCATT---TTCAGTGC-------------------GGGTGCGGGGTGCGCTTATCAGG----CCGCTTATCTCCTACA--CGCAAAA---------CCCTGCT---GGCAATA--ACCACTCCTT--------CCCGTCG-CCAACACC--------------------------------------------------------------GAATATGACGCTGACTCAC----TTTCCACACAGCCGCCGAGCTGGGTAAGGG

>Diaporthe_discoidispora_ICMP20662

GAGAAGGAAGGTTAGTAAACAT--CATGA----------GCTCGCAGCATA--CAAT--CTC-T-TG-----CCCTCCATATGCA--------CTCAGAATCATCG--GCGCG----CTAC-ATTCTGCGCGGCTTCGTAACATCC----GTAAAGGCA-TTTTCACCCCTCGCTCTGGA----TTTTCCATT---TTCAGTGC-------------------GGGTGCGGGGTGCGCTTATCAGG----CGGCTTATCTCTTACA-TCACAAAA---------CCCTCTT----GCATCA--ACCACTCCAT--------CCCGTTG-CTACTACC--------------------------------------------------------------GGAACCTAAGCTGACCTTTTTTTCTTCTACACAGCCGCCGAGCTTGGTAAGGG

>Diaporthe_durionigena_VTCC_930005

????????????????????????????????????????TGTGCTGCTTG--CGCT--CCA-T-AT-----CCCTCCACATGCAC-------ATGGGAATCGTTG--CCGTG----CG-C-TGTCTGCGACGCTTCGTCACACCC----GTCAAGGCA-TTTTCACCCCTCCCTCTGGA----TTTTCCATT---TTCAGTGC-------------------GGGTGCGGGGTGCGCTTATCAGG----CCGCTTATCTCACA---CATCAAA----------CCCTGTC----GCACCTCTGCCACTCTTG--------ACCAGTG-ACACACCAACAGCGTCACTTTCATTCCCA--TTTGTTGCTCCAGGAAGCTTTTTGAATCGTTC-------GAAAATCATGCTGACTC------TTTCTACACAGCCGCCGAGCTGGGTAAGGG

>Diaporthe_eres_CBS_138594

GAGAAGGAAGGTTAGTAAATAT--CAC------------AGTCACGGAACA--TGCT--ACC-T-GG-----CCCTCCATAC-TGC-------ACCTCAATCATCA--GCCCG----CAGC-TGCTCGCGCGGCCTCGCCATGTCG----GGGGGCGCA-TTTTCACCCCTCGCTTTGGA----TTTTCAATT---TTCAGTGC-------------------GAGTGCGGGGTGCGCTTATCAGGGGGCGGGCTTATCTCCTACA--ACCAAAA---------CCCTGTT----ACATCA--CTCACTCAATC-------CTTGTCA-CCACCACC--------------------------------------------------------------GAATATTATGCTGACCCTC----TATCTACACAGCCGCCGAGCTTGGTAAGGG

>Diaporthe_fici_septicae_MFLU_18_2588

GAGAAGGAAGGTTAGTAAACAT--CATGA----------GTTCGCAGCATA--CACT--TCC-T-TG-----CCCTCCACATGCAT-------CGCAGAATCATTG--GCGCG----CGGC-CATCTGCGCGGCTTCGTCACATCC----GTCAAGGCA-TTTTCACCCCTCGCTCTGGA----TTTTCCATT---TTCAGTGC-------------------GGGTGCGGGGTACGCTTATCAGG----CCGCTTATCTCTTGCA--TCCACAA---------CCCTGTT----GCATCA--CCCACTCCAT--------CCCGCCG-CCACCACCAACACCATCACTACCATCTTTGAACTGAAAAAGAACAACCTGATGAATTGCGCT--------GGAATCTATGCTGACCATT----TTTCTACACAGCCGCCGAGCTTGGTAAGGG

>Diaporthe_fructicola_MAFF_246408

GAGAAGGAAGGTTAGTAAACAT--CGCCCA---------ACGGGCACCTTG--ACCT--CCA-C-AT-----CACCCCATACGCCC---------AGAACTCGTTG--GCGCG----CG-C-TGTATGCGAGGCTTCGTCACACCT----GTCAAGGCA-TTTTCACCCCTCCCTCTGGA----TTTTCCATT---TTCAGTGC-------------------GGGTGCGGGGTGCGCTTATCAGG----CCGCTTATCTTTCA---CACCAAAA---------CCCTGTC----GCACCTTACCTCTCTTTC--------ACCAGTG-ACGCACCAACAGCATCACCTTCACTCCCA--TCTTTTGCTTGAGGGAGCTTTTCAACTCGCTC-------GAAAATGATGCTGACTC------TTTCTGAACAGCCGCCGAGCTGGGTAAGGG

>Diaporthe_ganjae_CBS_180_91

NNNNNNNNNNNNNNNNNNNNNNNNNNNNAA---------CTCCACAACATC--AACC--CCAA--AGTC---ATCCCAGTGCACAT-------CGCGCGATCATCG--GCGCG----CGAC-CGTCTGCGCCGCTTCGT--CACTC----GTCACAGCA-TTTTCACCCCTCGTCCTGGA----TTTTCCACT---TTCAGTGC-------------------GGGTGCGGGGTGTGCTTATCAGG----CCGCTTATCGCTTGCG-TTCCAAAA---------CCCTGCCTGGGGCACCA--ACCACTCTTC--------GCCGCTG-CCCACATT--------------------------------------------------------------GAAAACCATGCTGACTCGC----TTTCCATACAGCCGCCGAGCTGGGTAAGGG

>Diaporthe_goulteri_BRIP_55657a

GAGAAGGAAGGTTAGTAAAAAC--AACACAA--------ATCCTCTGCATA--TACT--CCA----------CTCTTTCACAGTAC-------TATGGGAGTACTG--GTGCG----CGGC---CTGTGGCGGCTTCGTCACACCC----GTCAAGCCA-TTTTCACCCCTCCCTCTGGA----TTTTCCATT---TTCAGTGC-------------------GAGTGCGGGGTGTCCTTATCAGG----CGGCCTATCTCTTACACTCACAAAACTCGCAAACCCCTGCT----CCTTCA--ACCGCTGCCC--------AGAGCAA-ACACCACC--------------------------------------------------------------GAATATCATGCTGACA-------TGAGTATACAGCCGCCGAGCTGGGCAAGGG

>Diaporthe_guangdongensis_ZHKUCC20_0014

GAGAAGGAAGGTTAGTAAACAT--CACCAA---------ACCCGCAGCTTG--ACCT--CCA-T-GC-----TCCTCCGTATGCAC-------ATGGAAATCGTTG--GCGCG----CG-C-TGTCTGCGAGGCTTCGTCACACCT----GTCAAGGCA-TTTTCACCCCTCCCTCCGGA----TTTTCCATT---TTCAGTGC-------------------GGGTGCGGGGTGCGCTTATCAGA----CCGCTTATCTCCTCG--CACCAAAA---------CCCTGCC----GCACCT--AGCACTCTTC--------ACCTGTG-ACGCACCAATATCATCGTTTTTATTCCCAATTTTGTTGCGCGAAGGAGCTACTCGAATCGCTT-------GGAAGTCATGCTGACTC------TTTCTGTACAGCCGCCGAGCTGGGTAAGGG

>Diaporthe_gulyae_BRIP_54025

NAGAAGGAAGGTCAGTAAACAT--CACACATCCAACACTTCCCACAGTATC--AACT--CCA-C-AAAG---TACTCCATGCGCAC-------ATTGTCATCATTG--GTGCG----CGAC-TGCCTGCGCGGCTTCGTCACACCC----GTCAAGCCA-TTTTCACCCCTCCCTCTGGG--TTTTTTCCATT---TTCAGTGC-------------------GGGTGCGGGGTGCGCTTATCAGG----CCGCTTATCTCCCACA--CGCAAAA---------CCCTGCT---GGCATTA--ACCACTCCCT--------ACCACCG-TCAACACC--------------------------------------------------------------GAACATGACGCTGACTTAA----TTTTCATACAGCCGCCGAGCTGGGCAAGGG

>Diaporthe_guttulata_CGMCC_3_20100

NNNNNNNNNNNNNNNNNNNNNNNNNNNNNNNNNNNNNNNNNTCACAGCATC--AACT--CCA-C-AAAG---CCCTCCATGCGCAC-------ATTGTCATCATTG--GTGCG----CGAC-TGCCTGCGCGGCTTCGTCACACCC----GTCAAGCCA-TTTTCACCCCTCCCTCTGGG--TTTTTTCCATT---TTCAGTGC-------------------GGGTGCGGGGTGCGCTTATCAGG----CCGCTTATCTCCCACA--CGCAAAA---------CCCTGCT---GGCATTA--ACCACTCCCT--------ACCACCG-TCAACACCGACTCTACCACTTTGAACCCTATCAAACACCCTCCTGCCGAAAAGAACTGTCAA--------GAACATGACGCTGACTTGA----TTTCCATACAGCCGCCGAGCTTGGTNNNNN

>Diaporthe_helianthi_CBS_592_81

GAGAAGGAAGGTTAGTAAACAT--TACCA----------GGCCGCACCTTG--AACCCATAA-CCCT-----CACTCCACATGCACATC----ATGATAATCGTTG--GCGCG----CCAC-TGCATGCGAGGCTTCGTCACACCT----GTCAAGGCA-TTTTCACCCCTCCCTCTGGA----TTTTCCATT---TTCAGTGCGGGTGCGGGTACGGGTGCGGGGTGCGGGGTGCGCTTATCAGG----CCGCTTATCTCTTACA---CCAAAA---------CCCTCCT-TATGCACCC--ACCGCACTCC--------TGCACCA-GTACAACCTT------------------------------------------------------------CAGAATCGGACTGACTC------TTTCTATATAGCCGCCGAGTTGGGTAAGGG

>Diaporthe_heterostemmatis_SAUCC194_85

??????????????????GCGT--ACTCGC---------ACGGGCACCTTG--ACCT--CCA-C-AT-----CACCCCATACGCCC---------AGAACTCGTTG--GCGCG----CG-C-TGTCTACGAGGCTTCGTCACACCT----GTCAAGGCA-TTTTCACCCCTCCCTCTGGA----TTTTCCATT---TTCAGTGC-------------------GGGTGCGGGGTGCGCTTATCAGG----CCGCTTATCTTTCA---CACCAAAA---------CCCTGTC----GCACCTTACCTCTCTTTC--------ACCAGTG-ACGCACCAACAGCATCACCTTCATTCCCA--TCTTTTGCTTGAGGGAGCTTTTCAACTCGCTC-------GAAAATGATGCTGACTC------TTTCTGAACAGCCGCCGAGCTG????????

>Diaporthe_hordei_CBS_481_92

GAGAAGGAAGGTTAGTAAACAT--CACCCA---------ATCTGCAGCTTG--AACT--CCA-T-AG-----CCCGCCACATGCCC-------ATCAAATTCG-----ACGCG----CGAC-TCTCTGCGAGGCTTCGTCATACCT----GTCAAGGCA-TTTTCACCCCTCCCTCTTGA----TTTTCCATT---TTCAGTGC-------------------GGGTGCGGGGTCCGCTTATCAGG----CCGCTTATCTCTTA---CACCAAAA---------CCCTGTC----GCACCT--ACCATTATTC--------GCCAGCG-CCACCCC---------------------------------------------------------------GAAAATCATGCTGACTC---------CTATACAGCCGCTGAGCTGGGTAAGGG

>Diaporthe_hubeiensis_JZB320123

GAGAAGGAAGGTTA-------------------------GTCTACGGCATA--CACT--CCCAT-TG-----TACCCCAGTTGCAC-------ATCAGGATTACTG--GCGCG----CTGC-CGTTTGCGCGGCTTCGTCACACCC----GCCAGGGCA-TTTTCACCCCTCCCTCTGGA----TTTTCCATT---TTCAGTGC-------------------GGGTGCGGGGTGCGCTTATCAGG----CGGCTTATCTCTGACG-CTCACAAA---------CCCTGCT----GCATCA--ACCATTCCTT--------GTCGCTG-CCACCACCACCATCACCGTCAAATTCATCGCACGGTCAAATTGATCGGATGACTTGTGTCTTGGAGA---AATCATGAAGCTGACTTTC----CATCTCTATAGCCGCCGAGCTCGGTAAGGG

>Diaporthe_infecunda_CBS_133812

GAGAAGGAAGGTCAGTAAATAT--CACACA---------TGCCGCATCATG--AACC--CCA-T-CAAG---TCCCCCACACGCAC-------ATCGTCACCATGG--GCGCG----CGAC-TGTTTGCGCGGCTGCGTCGCACCC----TTCAAGCCA-TTTTCACCCCTCCCTCTGGA---TTTTTCCATT---TTCAGTGC-------------------GGGTGCGGGGTGCGCTTATCAGG----CCGCTTATCTTCCACA--TGCAAAA---------CCCTGCT---GGTGTCA--ATCACTTCCT--------TGCACTG-CCAGCACC--------------------------------------------------------------GACAATCATGCTGACTTAC----TTCCCACACAGCCGCCGAGCTGGGTAAGGG

>Diaporthe_infertilis_CBS_230_52

GAGAAGGAAGGTTAGTAAATAT--CACAAC---------ATGCGCAGTTTG--TCCA--CCA-T-AT-----TCCTCCATGTGCTC-------ACGAAACTCGTCG--GCGCA----CC-C-CGTCTGCGAGGCTTCGTCACACCT----GTCAAGGCA-TTTTCCCCCCTCCCTCTGGA----TTTTCCATT---TTCAGTGC-------------------GGGTGCGGGGTGCGCTTATCAGG----CCGCTTATCTCTCA---CACCAAAA---------CCCTGTC----GCACCT-TACCACTCCTT--------GCCAGTC-ATGCACA---------------------------------------------------------------GAAACTCATGCTGACTT------TTCCTACACAGCCGCTGAGCTGGGTAAGGG

>Diaporthe_kochmanii_BRIP_54033

??GAAGGAAGGTTAGTAAACAT--CGCCCA---------ACGGGCACCTTG--ACCT--CCA-C-AT-----CACCCCATACGCAC---------AGAACTCGTTG--GCGCG----CG-C-TGCCTGCGAGGCTTCGTCACACCT----GTCAAGGCA-TTTTCACCCCTCCCTCTGGA----TTTTCCATT---TTCAGTGC-------------------GGGTGCGGGGTGCGCTTATCAGG----CCGCTTATCTCTCA---CACCAAAA---------CCCTGTC----GCACCT-ACCTCTCTTCC--------ACCAGTG-ACGCACC---------------------------------------------------------------GAAAATGATGCTGACTC------TTTCTGAACAGCCGCCGAGCTGGGCAAGGG

>Diaporthe_kongii_BRIP_54031

???AAGGAAGGTTAGTAAACAT--CGCCCA---------ACGGGCACCTTG--ACCT--CCA-C-AT-----CACCCCATACGCCC---------AGAACTCGTTG--GCGCG----CG-C-TGTCTGCGAGGCTTCGTCACACCT----GTCAAGGCA-TTTTCACCCCTCCCTCTGGA----TTTTCCATT---TTCAGTGC-------------------GGGTGCGGGGTGCGCTTATCAGG----CCGCTTATCTTTCA---CACCAAAA---------CCCTGTC----GCACCTTACCTCTCTTTC--------ACCAGTG-ACGCACC---------------------------------------------------------------GAAAATGATGCTGACTC------TTTCTGAACAGCCGCCGAGCTGGGCAAGGG

>Diaporthe_leucospermi_CBS_111980

NNNNNNNAAGGTCAGTAAATAT--CACACA---------TGCCGCATCATG--AACC--CCA-T-CAAG---TCCCCCACACGCAC-------ATCGTCACCATGG--GCGCG----CGAC-TGTTTGCGCGGCTGCGTCGCACCC----TTCAAGCCA-TTTTCACCCCTCCCTCTGGA---TTTTTCCATT---TTCAGTGC-------------------GGGTGCGGGGTGCGCTTATCAGG----CCGCTTATCTTCCACA--TGCAAAA---------CCCTGCT---GGCGTCA--TTCACCTCCT--------TGCACTG-CCAGCACC--------------------------------------------------------------GACAATCATGCTGACTCAC----TTCCCACACAGCCGCCGAGCTGNNNNNNNN

>Diaporthe_longicolla_FAU_599

?AGAAGGAAGGTCAGTAAATAC--CACCAC---------ACGTGCAGCTTG--CGCT--CCA-T-AT-----CCCTCCATGTGCAC-------ATAGAAACCGTTG--GCGTG----CG-C-TGTCTGCGACGCTCCGTCACACCTACCTGTAAAGGCA-TTTTCACCCCTCCCTCTGGA----TTTTCCATT---TTCAGTGC-------------------GGGTGCGGGGTGCGCTTATCAGG----CCGCTTATCTCACA---CATCAA-----------CCCTGTC----GCACCTTTACCACTGTTC--------GCCAGTG-ACACACC---------------------------------------------------------------CAAAATCATGCTGACTC------TTTCTACACAGCCGCCGAGCTGGGTAAGGG

>Diaporthe_longispora_CBS_194_36

GAGAAGGAAGGTCAGTAAACAC--CAAAAA---------GTCCATGCCACA--CACC--TCA-T-TG-----CCCTCCATGTGCAC-------CTCGGAATCATTG--GCGCG----CGGC-CGTTTGCGCGGCTTCGTCCCACCC----CTCAAGGCA-TTTTCACCCCTCCCTCTGGA----TTTTCCATT---TCTAGTGC-------------------GGGTGCGGGGT--GCTTATCAGG----CGGCTTATCTCTCGCA--CCCAAAA---------CCCTGTT----GCATCA--ACCACTCCTT--------ACTGCTGCCCACCACC--------------------------------------------------------------GGAAATGAAGCTGACATTT----TTTCTACACAGCCGCCGAGCTGGGTAAGGG

>Diaporthe_lusitanicae_CBS_123212

GAGAAGGAAGGTCAGTAAACAT--CACGCA---------TCCCACAGCATG--AACT--CCA-C-AAAG---TCCTCCATGCGCAC-------ATTGTCATCATTG--GTGCG----CGAC-TGCCTGCGCGGCTTCGT--CACCC----GTCAAGGCA-TTTTCACCCCTCCCTCTGGG-TTTTTTTTCATT---TTCAGTGC-------------------GGGTGCGGGGTGCGCTTATCAGG----CCGCTTATCTCCTACA--CGCAAAA---------CCCTGCT---GGCATTA--ACCACTCCTT--------ACCACCG-CCAACACC--------------------------------------------------------------GAAAATGACGCTGACTTAC----TTTTCACCCAGCCGCCGAGCTGGGTAA-GG

>Diaporthe_machili_SAUCC194_111

NNNNNNNNNNNNNNNGATTCTT--CTTAAA---------TCCCGCATCATG--AACC--CCA-T-GAAC---TCCGCCTCACGCAC-------ATCGTCACCATGG--GCGCG----CGACTTTTTTGCACGGCTGCGTCACACCC----GTCAAGCCA-TTTTCGCCCCTCCCTCTGGA---TTTTTCCATT---TTCAGTGC-------------------GGGTGCGGGGTGTGCTTATCTGG----CGGCTTATCTCCCACA--CGCAAAA---------CCCTGCT---GGCATCA--ATCACTTCCT--------TGCACTG-CCTGCTCCAACATCACAAACATCAAACACCATCAACCTCTACTTGCGCAACA-ATCTGTTCG--------GACAATCATGCTGACTTAC----ATCCCACACAGCCGCCGAGCTGNNNNNNNN

>Diaporthe_manihotia_CBS_505_76

GAGAAGGAAGGTCAGTAAACAC--TACAAA---------CTCCACAACACC--AACC--CCAA--AGTC---ACCCCAGTGCACCT-------CGCGCGACCATTGGCGCGCG----CGAC-AGCCTGCGCGGCTTCGT--CACTC----GTCACAGCA-TTTTCACCCCTCGTTCTGGA----TTTTCCACT---TTCAGTGC-------------------GGGTGCGGGGTGTGCTTATCAGG----CCGCTTATCGCGTGCC-TTCCAAAA---------CCCTGCTGCGGGCATCA--ACCACTCTCT--------GCCGCTG-CCCACCAC--------------------------------------------------------------GAAAACCATGCTGACTCGC----TTTCCATACAGCCGCCGAGCTGGGTAAGGG

>Diaporthe_mayteni_CBS_133185

NNNNNNNNNNNNNNNNNNNNNNNNNNNNNNNNNNNNNNNNNNNNNNNNNNNNNNNNNNNNNNNNNNNNNNNNNNNNNNNNNNNNNNNNNNNNNNNNNNNNNNNNNNNNNNNNNNNNNCGGC-CATCTGTGCGGCTTCGTCTCTCCC----G-CAAGGCA-TTTTCACCCCTCCCTCTGGA----TTTTCCATT---TTCAGTGC-------------------GGGTGCGGGGTGCGCTTATCAGG----CCGCTTATCTCTCACA--CCCAAAA---------CCCTGGT----GCATCA--ACCACTCCTT--------ACCGCAG-CTACCATC--------------------------------------------------------------GGAAATCATGCTGANNNNNNNNNNNNNNNNNNNNNNNNNNNNNNNNNNNNNNN

>Diaporthe_megalospora_CBS_143_27

GAGAAGGAAGGTTAGTAAACATACCCCCCT---------CTCCGCAGCTTG--AACT--CCA-C-AG-----CCCTCTATATGCAC-------AAGGAAATCTTTG--GGCCG----CGAC-TGTCTGCGAGGCTTCGTAAGACCT----GCCATGGCA-TTTTCGCCCCGCCCTCTGGA----TTTTTCATT---TTCAGTGC-------------------GGGTGCGGGGTGCGCTTATCAGG----CCGCTTATCTCTTGAA-CACCAAAA---------CCCTGTT----GCACCT--ACCACTCTTC--------ACCACTG-CCACCCC---------------------------------------------------------------GAAAATCATGCTGACTC------TTTCTTCACAGCCGCCGAGCTGGGTAAGGG

>Diaporthe_melonis_CBS_507_78

GAGAAGGAAGGTTAGTAAACAT--CACCAA---------ACCCGCAGTTTG--ACCT--CCA-T-GT-----CCCTCCATACGCAC-------ATAGAAATCGTTG--GCGCG----CG-C-TGCCTGCGGGGCTTCGTCACACCT----GTCCAGGCA-TTTTCACCCCTCCCTCCGGA----TTTTCCATT---TTCAGTGC-------------------GGGTGCGGGGTGTGCTTATCAGA----CCGCTTATCTCCTCG--CACCAAAA---------CCCTGCC----GCACCT--AGCACACTTC--------ACCTGTG-ACGCACC---------------------------------------------------------------GAAAGTCATGCTGACTC------TTTCTGTACAGCCGCCGAGCTGGGTAAGGG

>Diaporthe_middletonii_BRIP_54884e

NAGAAGGAAGGTCAGTAAACAT--CACACA---------TCCCGCATCATG--AACC--CCA-T-GAAC---CCCGCCTCACGCAC-------ATCGTCACCATGG--GCGCG----CGAC-TTTTTGCGCGGCTGCGT--CACCC----GTCAAGCCA-TTTTCGCCCCTCCCTCTGGA---TTTTTCCATT---TTCAGTGC-------------------GGGTGCGGGGTGTGCTTATCAGG----CCGCTTATCTCCCACA--CGCAAAA---------CCCTGCT---GGCATCA--ATCACTTCCT--------TGCACTG-CCAGCTCC--------------------------------------------------------------GACAATCATGCTGACTTAC----TTCCCACACAGCCGCCGAGCTGGGCAAGGG

>Diaporthe_miriciae_BRIP_54736j

GAGAAGGAAGGTTAGTAAATAT--CACTAC---------ATGTGCTGCTTG--CGCT--CCA-T-AT-----CCTTCCACATGCAC-------ATGGGAATCGTTG--CCGTG----CG-C-TGTCTGCGACGCTTCGTCACACCC----GTCAAGGCA-TTTTCACCCCTCCCTCTGGA----TTTTCCATT---TTCAGTGC-------------------GGGTGCGGGGTGCGCTTATCAGG----CCGCTTATCTCACA---CATCAAA----------CCCTGTC----GCACCTCTGCCACCCTTC--------ACCAGTG-ACACACC---------------------------------------------------------------GAAAATCATGCTGACTT------TTTCTACACAGCCGCCGAGCTGGGCAAGGG

>Diaporthe_myracrodruonis_URM7972

NNNNNNNNNNNNNNNNNNNNNNNNNNNNNNNNNNNNNNNNNNNNNNNNNNNNNNNNNNNNNNNNNNNNNNNNNNNNNNNNNNNNNNNNNNNNNNNNNNNNNNNNNNNNNNNNNNNNNNNNNNNNNNNNNNNNNNNGTGTCACACCC----GTCATGCCA-TTTTCACCCCTCCCTCTGGA---TTTTTCCATT---TTCAGTGC-------------------GGGTGCGGGGTGCGCTTATCAGG----CCGCTTATCTCCCACA--CGCAAAA---------CCCTGCT---GGCATCA--ATCACTTCCT--------TGCACTG-CCAGCACCAACGTCACAAACATCAAACCCCATCAACCTCTACTTGCGCAACA-AACTGCTCG--------GACAATCATGCTGACTTAC----TTCTCACACAGCTGCCGAGCTGGGTAAGGG

>Diaporthe_neoarctii_CBS_109490

GAGAAGGAAGGTCAGTAAACAT--CCCACA---------TCCCACAGCATC--ATTT--CCT-C-AAGGCCATCCTCCATGTGCAC-------ATTGTCATCATTG--GCGCGCGACCGAC-CGCCTGCGCGGCTTCGTCACACCC----GTCAAGCCA-TTTTCACCCCTCCCTCTGGG--TTTTTTCCATT---TTCAGTGCGGGC---------------GGGTGCGGGGTGTGCTTATCAGG----CCGCTTATCT-CCACA--CGCAAAA---------CCCTGCT---GGCATTA--ACCACGCCTT--------ACCACCG-CCAACACC--------------------------------------------------------------GAATATGACGCTGACGTAA----TTTCCACACAGCCGCCGAGCTGGGTAAGGG

>Diaporthe_neoraonikayaporum_MFLUCC_14_1136

GAGAAGGAAGGTTAGTAAATAT--CAAAA----------ACTCCCAGCATA--CACT--GCA-T-TA-----TCCTCCATGTGCTA-------CTTAGAATCATCG--CTGCG----CGGC-CACCTGCGCGGC-TCATCACACCC----GTCAACGCA-TTTTCACCCCTCCCTCTGGA----TTTTCCATT---TTCAGTGC-------------------GGGTGCGGGGTGTGCTTATCTGG----CGGCTTATCTCCCACACCTCAAAAA---------CCCTGTC----GCATCA--ACCACCACCT--------GCCACCA-CCATCAAC--------------------------------------------------------------AGAAATCGTGCTGACNNNNNNNNNNNNNNNNNNNNNNNNNNNNNNNNNNNNNN

>Diaporthe_novem_CBS_127271

GAGAAGGAAGGTCAGTAAATAT--CGCACA---------TCCCACAGCATG--AACT--CCA-C-CAAG---CCCACCGTGCGCAC-------ATCGTCATCATTG--GTGCG----CGAC-TGCCTGCGCGGCTTCAT--CACCC----CTCAAACCA-TTTTCACCCCTCCCTCTGGGTTTTTTTTCCATT---TTCAGTGC-------------------GGGTGCGGGGTGCGCTTATCA-G----CCGCTTATCTCCT--A--TGCAAAA---------CCCTGCT---GGCATTA--ACCACTCCTT--------GCCACCG-CCAACACC--------------------------------------------------------------GAATATGAAGCTGACTTAC----TCTCCACACAGCCGCCGAGCTGGGTAAGGG

>Diaporthe_ovalispora_ICMP20659

GAGAAGGAAGGTTAGTAAACAT--CACCGA---------ACGGGCACCTTG--ACCT--CCA-C-AT-----CTCTCCATATCCAC---------AGAACTCGCTG--GCGCG----TG-A-TGTCTGCGAGGCTTCGTCACGCCT----GTCAGGGCA-TTTTCACCCCTCCCTCTGGA----TTTTCCATT---TTCAGTGC-------------------GGGTGCGGGGTGCGCTTATCAGG----CCGCTTATCTAT-----CACCAAAA---------CCCTGTC----GCACCTCACCACTCTTTC--------ACCAGTG-GCGTGCC---------------------------------------------------------------GAGAATCATGCTGACTC---------CTACACAGCCGCCGAGCTGGGTAAGGG

>Diaporthe_pachirae_CDA_728

NNNNNNNAAGGTCAGTAAATAT--CACACA---------TGCCGCATCATG--AACC--CCA-T-CAAG---TCCCCCACACGCAC-------ATCGTCACCATGG--GCGCG----CGAC-TGTTTGCGCGGCTGCGTCGCACCC----TTCCAGCCA-TTTTCACCCCTCCCTCTGGA---TTTTTCCATT---TTCAGTGC-------------------GGGTGCGGGGTGCGCTTATCAGG----CCGCTTATCTTCCACA--TGCGAAA---------CCCTGCT---GGCGTCA--ATCACTTCCT--------TGCACTG-CCAGCACCAACGTCACAAACATCAAACCCCATCAACCTCTACTTGCGCGACAGAACTGTTCG--------GACAATCATGCTGACTTAC----TTCCCACACAGCCGCCGAGCTGGGCAAGGG

>Diaporthe_passifloricola_CBS_141329

????????????????????????????????????????????????????????????????????????????????????????????????????????????????????????????????????????????????????????????????????????????????????????????????????????????????????????????????????????????????????????????????????????????????????????????????????????????????????????????????????????????????????????????????????????????????????????????????????????????????????????????????????????????????????????????????????

>Diaporthe_pseudolongicolla_CBS_117165

NNNNNNNNNNGTCAGTAAATAT--CGCACA---------TCCCACAGCATG--AACT--CCA-C-CAAG---CCCACCGTGCGCAC-------ATCGTCATCATTG--GTGCG----CGAC-TGCCTGCGCGGCTTCAT--CACCC----CTCAAACCA-TTTTCACCCCTCCCTCTGGGTTTTTTTTCCATT---TTCAGTGC-------------------GGGTGCGGGGTGCGCTTATCA-G----CCGCTTATCTCCT--A--TGCAAAA---------CCCTGCT---GGCATTA--ACCACTCCTT--------GCCACCG-CCAACACCAACTCTACCACTTTCAACCCTATCAATCTCCCAGGCGAAACG---AACTGTCAA--------GAATATGAAGCTGACTTAC----TCTCCACACAGCCGCCGAGCTGGGTAAGGG

>Diaporthe_pyracanthae_CBS142384

NNNNNNNAAGGTCAGTAAATAT--TACACA---------TGCCGCATCATG--AACC--CCA-T-CAAG---TCCCCCACACGCAC-------ATCGTCACCATGG--GCGCG----CGAC-TGTTTGCGCGGCTGCGTCGCACCC----TTCAAGCCA-TTTTCACCCCTCCCTCTGGA---TTTTTCCATT---TTCAGTGC-------------------GGGTGCGGGGTGCGCTTATCAGG----CCGCTTATCTTCCACA--TGCAAAA---------CCCTGCT---GGCGTCA--TTCACCTCCT--------TGCACTG-CCAGCACC--------------------------------------------------------------GACAATCATGCTGACTCAC----TTCCCACACAGCCGCCGAGCTGNNNNNNNN

>Diaporthe_racemosae_CBS_143770

GAGAAGGAAGGTTAGTTAACAT--CACCCA---------ACCCGCAGCCTGAAAACT--TCA-T-AG-----CCTTC-ACATGCAC-------ATCAAAATCGTTG--GCGCG----CGAC-TGTCTGCGAGGCTTCGTCACAACT----GTCAAGGCA-TTTTCACCCCTCGCTCTGGA----TTTTCCATT---TTCAGTGC-------------------GGGTGCGGGGTGCGCTTATCAGG----CCGCTTATCTGCTC---CACCAAAA---------CCCTGTT----GCACCA--ACCGCTCTTT--------ACCACTG-CCACGCA---------------------------------------------------------------GGAAATCATGCTGACTT------GTTCTGTACAGCCGCCGAGCTGGGCAAGGG

>Diaporthe_raonikayaporum_CBS_133182

GAGAAGGAAGGTTAGTAAATAT--CAAA-----------ACTCCCAGCATA--CACT--GCA-T-TA-----TCCTCCATGTGCTA-------CTCAGAATCATCG--CTGCG----CGGC-CACCTGCGCGGC-TCATCACACCC----GTCAATGCATTTTTCACCCCTCCCTCTGGA----TTTTCCATT---TTCAGTGC-------------------GGGTGCGGGGTGTGCTTATCTGG----CGGCTTATCTCCCACA-CTCAAAAA---------CCCTGTT----GCATCA--ACCACTCCCTAC----CTGCCACCA-CCATCAAC--------------------------------------------------------------AGAAATCGTGCTGACCTCTT---TGTCTGCGTAGCCGCCGAGCTCGGTAAGGG

>Diaporthe_rosae_MFLUCC_17_2658

????????????????????????????????????????????????????????????????????????????????????????????????????????????????????????????????????????????????????????????????????????????????????????????????????????????????????????????????????????????????????????????????????????????????????????????????????????????????????????????????????????????????????????????????????????????????????????????????????????????????????????????????????????????????????????????????????

>Diaporthe_rosiphthora_COAD_2913

GAGAAGGAAGGTCAGTAAACAT--CACCCA---------ACCCGCAGCCTG--AACA--TCA-T-AG-----CCTTC-ACATGCAC-------ATCAAAATCGTTG--GCGCG----CGAC-TGTCTG-GAGATTTCGTCACAACT----GTCAAGCCA-TTTTCACCCCTCGCTCTGGA----TTTTCCATT---TTCAGTGC-------------------GGGTGCGGGGTGCGCTTATCAGG----CCGCTTATCTCCTC---CACCAAAA---------CCCTGTT----GCACCA--ATCGCTCTTT--------ACCACTG-CCACGCAAACGTCGTTGCCATCATCCTCAAATCTCGCCAACAAACCTTCAATGTGGCTG-----------GGAAATCATGCTGACCC------CTTCTGTACAGCCGCCGAGCTGGGTAAGGG

>Diaporthe_rossmaniae_CAA762

NNNNNNNAAGGTCAGTAAATAT--TACACA---------TGCCGCATCATG--AACC--CCA-T-CAAG---T-CCCCACACGCAC-------ATCGTCACCATGG--GCGCG----CGAC-TGTTTGCGCGGCTGCGTCGCACCC----TTCAAGCCA-TTTTCACCCCTCCCTCTGGA---TTTTTCCATT---TTCAGTGC-------------------GGGTGCGGGGTGCGCTTATCAGG----CCGCTTATCTTCCACA--TGCAAAA---------CCCTGCT---GGTGTCA--ATCACCTCCT--------TGCACTG-CCAGCACCAACGTCACAAACCTCAAACCCCATCAACCTCTACTTGCGCGACAGAACTGTTCG--------GACAATCATGCTGACTCAC----TTCCCACACAGCCGCCGAGCTGGGCAAGGG

>Diaporthe_sackstonii_BRIP_54669b

NAGAAGGAAGGTCAGTAAACAT--TACACA---------TCCCGCATCATG--AACC--CCA-T-GAAC---TCCGCCTCACGCAC-------ATCGTCACCATGG--GCGCG----CGAC--TCTTGCACGGCTGCGTCACACCC----GTCAAGCCA-TTTTCGCCCCTCCCTCTGAA---TTTTTCCATT---TTCAGTGC-------------------GAGTGCGGGGTGTGCTTATCAGG----CCGCTTATCTCCCACA--CGCAAAA---------CCCTGCT---GGCATTA--ATCACTTCCT--------TGCACTG-CCTGCTCC--------------------------------------------------------------GACAATCATGCTGACTTAC----ATCTCACACAGCCGCCGAGCTGGGCAAGGG

>Diaporthe_schini_CBS_133181

GAGAAGGAAGGTCAGTAAACAT--CACCCA---------ACCCGCAGCCTG--AACA--TCA-T-AG-----CCTTC-ACATGCAC-------ATCAAAATCGTTG--GCGCG----CGAC-TGTCTGCGAGATTTCGTCACAACT----GTCAAGCCA-TTTTCACCCCTCGCTCTGGA----TTTTCCATT---TTCAGTGC-------------------GGGTGCGGGGTGCGCTTATCAGG----CCGCTTATCTCCTC---CACCAAAA---------CCCTGTT----GCACCA--ATCGCTCTTT--------ACCACTG-CCACGCA---------------------------------------------------------------GGAAATCATGCTGACTC------CTTCTGTACAGCCGCCGAGCTGGGTAAGGG

>Diaporthe_schoeni_MFLU_15_1279

NNNNNNNNNNNNNNNNNNNNNNNNNNNNNNNNNNNNNNNNNNNNNNNNNNNNNNNNNNNNNNNNNNNNNNNNNNNNNNNNNNNNACATGCAGAATTGTCATCATTG--GTGCG----CGAC-TGCCTGCGCGGCTTCGTCACACCC----GTCAAGCCA-TTTTCACCCCTCCCTCTGGG--TTTTTTCCATT---TTCAGTGC-------------------GGGTGCGGGGTGCGCTTATCAGG----CCGCTTATCTCCCACA--CGCAAAA---------CCCTGCT---GGCATCA--ACTACTCCCT--------ACCACCG-TCAACACCAACTCTACCACTTTGAACCCTATCAAACACCCTCCTGCCGAAAAGAACTGTCAA--------GAACATGACGCTGACTTAA----TTTCCATACAGCCGCCGAGCTGGGTAAGGG

>Diaporthe_sclerotioides_CBS_296_67

NNNNNNNNNNNNNNNNNNNNNNNNNNNNNNNNNNNNNNNNTCCATGCCACA--CACT--CCA-T-TG-----CCCTCCATGTGCAG-------CTCAGAGTCATCG--TCGCG----CGGC-CGTTTGCCCGGCTGTGTCCCACCC----GTCAAGGCA-TTTTCACCCCTCCCTCTGGA----TTTTCCATT---TCCAGTGC-------------------GGGTGCGGGGT--GCTTATCAGG----CGGCTTATCTCTCGCA--TCCAAAA---------CCCTGTT----GCACCA--ACCACTCCCTCCCTTGCTGCTGCTG-CCACCACC--------------------------------------------------------------GGAGATCAAGCTGACATTT----CTTGTACACAGCCGCCGAGCTGGGCAAGGG

>Diaporthe_serafiniae_BRIP_55665a

NNNAAGGAAGGTCAGTAAACAT--CACACA---------TGCCGCATCATG--AACC--ACA-T-GAAG---TCCCCCACACGCAC-------ATCGTCACCATGG--GCGCG----CGAC-TGCTTGCGCGGCTGCGTCACACCC----GTCAAGCCA-TTTTCACCCCTCCCTCTGGA---TTTTTCCATT---TTCAGTGC-------------------GGGTGCGGGGTGCGCTTATCAGG----CCGCTTATCTCCCACACGCGCAAAA---------CCCTGCC---GGTATCA--ATCACTTCCT--------TGCACTG-CCAGCACC--------------------------------------------------------------GACAATCATGCTGACTTAC----TTCC--CACAGCCGCCGAGCTGGGCAAGGG

>Diaporthe_siamensis_MFLUCC_10_0573a

GAGAAGGAAGGTTAGTAAATAT--TACGA----------GTCCGTTGCATA--CACT--CTC-T-CG-----CCCTCCATATGCAT-------CTCAGAATCATTG--GCGCG----TGGC-CATCCGCGCGGCCTCGTCACATCT----GTCAAGGCA-TTTTCACCCCTCGCTCTGGA----TTTTCCATT---TTCAGTGC-------------------GGGTGCGGGGTGTGCTTATCAGG----CGGCTTATCTCTCACA--TCTATAA---------CCCTGCT----GCATCA--ACCACTCCAT--------CCCGCTG-CCACCACC--------------------------------------------------------------GGAATATATGCTGACCATTAGTGTTTGAAAACAGCCGCCAAGCTTGGTAAGGG

>Diaporthe_sinensis_ZJUP0033_4

GAGAAGGAAGGTTAGTAAACAA--CACA-----------GCCCCAAGCATG--AACC--CTA-C-AAAG---TCCTCCAGTCGCAC-------ATCGTCATTATTG--GTGCG----CGAC-TGCCCGCGCGGCTTTGTTAGACCC----GTCAGGCCA-TTTTCACCCCTCCCTCTGGT----TTTTCCCCCTCGTTCAGTGC-------------------GGGTGCGGGGTGCGCTTATCAAG----CCACTTATCTCCCACA--CGCAATA---------CCGTGCT---GGCAAGG--GCGGCTCCTC--------ACCACTG-TCAACACTCTCCCCGCCACCACCATCAACCCAATCCATCTCCCTGGCGAAACGAGCTTTCAG--------GATAATCAAGCTGACTTAC----TTTTCACATAGCCGCCGAGCTGGGTAAGGG

>Diaporthe_stewartii_CBS_193_36

NNNNNNNAAGGTCAGTAAACAT--CACACATCCAACCCTTCCCACAGCATC--AACT--CCA-C-AAAG---CCCTCCATGCGCAC-------ATTGTCATCATTG--GTGCG----CGAC-TGCCTGCGCGGCTTCGTCACACCC----GTCAAGCCA-TTTTCACCCCTCCCTCTGGG--TTTTTTCCATT---TTCAGTGC-------------------GGGTGCGGGGTGCGCTTATCAGG----CCGCTTATCTCCCACA--CGCAAAA---------CCCTGCT---GGCATTA--ACCACTCCCT--------ACCACCG-TCAACACC--------------------------------------------------------------GAACATGACGCTGACTTAA----TTTCCATACAGCCGCCGAGCTGNNNNNNNN

>Diaporthe_subordinaria_CBS_101711

GAGAAGGAAGGTCAGTAAACAT--CACACATCCAACCCTTCCCACAGCATC--AACT--CCA-C-AAAG---CCCTCCATGCGCAC-------ATTGTCATCATTG--GTGCG----CGAC-TGCCTGCGCGGCTTCGTCACACCC----GTCAAGCCA-TTTTCACCCCTCCCTCTGGG--TTTTTTCCATT---TTCAGTGC-------------------GGGTGCGGGGTGCGCTTATCAGG----CCGCTTATCTCCCACC--CGCAAAA---------CCCTGCT---GGCATTA--ACCACTCCCT--------ACCACCG-TCAACACC--------------------------------------------------------------GAACATGACGCTGACTTAAAT--TTCCCATACAGCCGCCGAGCTGGGTAAGGG

>Diaporthe_tecomae_CBS_100547

GAGAAGGAAGGTTAGT-AACAT--CACCCA---------ACCCGCAGCCCG--GGCT--CCA-T-AG-----CCCTCCGTATGCAC-------ATCAAATTCGTTG--GTGCG----CGAC-TGTCCGCAAGACTTGGTCACAACT----GTCAAGGCA-TTTTCACCCCTCGCTCTGGA----TTTTCCATT---TTCAGTGC-------------------GAGTGCGGGGTGCGCTTATCAGG----CCGCTTATCTCTTA---CACCAAAA---------CCCTTTT----GCACCA--ATCGCGCTTT--------ATCACTG-CCACGCA---------------------------------------------------------------GGAAATCATGCTGACTC------CTTCTGTACAGCCGCCGAGCTGGGTA????

>Diaporthe_tectonae_MFLUCC_12_0777

GAGAAGGAAGGTTAGTAAACAT--CAAAA----------GTCTACGGCATA--CACT--CCCAT-TG-----TACCCCAGTTGCAC-------ATCAGGATTACTG--GCGCG----CTGC-CGTTTGCGCGGCTTCGTCACAGCC----GCCAGGGCA-TTTTCACCCCTCCCTCTGGA----TTTTCCATT---TTCAGTGC-------------------GGGTGCGGGGTGCGCTTATCAGG----CGGCTTATCTCTGACG-CTCACAAA---------CCCTGCT----GCATCA--ACCATTCTTT--------GTCGCTG-CCACCACC--------------------------------------------------------------AATCATGAAGCTGACNNNNNNNNNNNNNNNNNNNNNNNNNNNNNNNNNNNNNN

>Diaporthe_tectonendophytica_MFLUCC_13_0471

GAGAAGGAAGGTTAGTAAATAT--CACTAT---------ATGCGCAGCTTG--CGCT--CCA-C-AT-----CCCTCCATATGCAC-------ATAGAGATCGTTG--GTGTG----CG-C-TGTCTGCGACGCTTCGTCACACCT----GTCAAGGCA-TTTTCACCCCTCCCTCTGGA----TTTTCCATT---TTCAGTGC-------------------GGGTGCGGGGTGCGC-TATCAGG----CCGCTTATCTCACA---CATCAA-----------CCCTGTC----GCACCTTTATCACTGTTC--------ACCAGTG-ACATACC---------------------------------------------------------------GAAAATCATGCTGAC??????????????????????????????????????

>Diaporthe_terebinthifolii_CBS_133180

GAGAAGGAAGGTTAGTAAACAT--TACCCA---------ACCCGCAGCCTG--AGCC--CCA-T-AG-----CCCTCTGTATGCAC-------ATCAAAATCGTTG--GCGCG----CGAC-TGTCCGCAAGACCTGGTCACAACT----GTCAAGGCA-TTTTCACCCCTCGCTCTGGA----TTTTCCATT---TTCAGTGC-------------------GAGTGCGGGGTGCGCTTATCAGG----CCGCTTATCTCTTA---CACCAAAA---------CCCTGTT----GCACCA--ATCGCTCTTT--------ACCACTG-CCACGCA---------------------------------------------------------------GGAAATCATGCTGACTC------CTACTGTACAGCTGCCGAGCTGGGTAAGG?

>Diaporthe_thunbergiicola_MFLUCC_12_0033

GAGAAGGAAGGTTAGTAAATAT--CACTAC---------ATGTGCTGCTTG--CACT--CCA-T-AA-----CCCTCC-CATGCAC-------ATGGAAATCGTTG--CCGTG----CG-C-TGTCTGCGACGCTTCGTCACACCC----GTCAAGGTA-TTTTCCCCCCTCCCTCTGGA----TTTTCCTTT---TTCAGTGC-------------------GGGTGCGGGGTGTGCTTATCAGG----CCGCTTATCTCCCA---CATCAAA----------CCCCGTC----GCACCTCTGCCACTCCTG--------ACCAGGG-ACACACCAACAGCGTCCTTTTCATTCCCA--TTTGTTGCTCCATGAAGTTTTTTGAATCGTTC-------GAAAAACAAGCTGACTC------TTTCTACACAGCCGCCAAGCTGGGTTAGGG

>Diaporthe_tulliensis_BRIP_62248a

G-GAAGGAAGGTTAGTAAACAT--CAAAA----------GTCTACGGCACA--CACT--CCCAT-TG-----TACCCCATTTGCAC-------ATCAGGATTACTG--GCGCG----CTGC-CGTTTGGGCGGCTTCGTCACACCC----GCCAGGGCA-TTTTCACCCCTCCCTTTGGA----TTTTCCATT---TTCAGTGC-------------------GGGTGCGGGGTGCGCTTATCAGG----CGGCTTA--TCTGACG-CTCACAAA---------CCCTGCT----GCATCA--ACCATTCCTT--------GTCGCTG-CCACCACC--------------------------------------------------------------AATCATGAAGCTGACTTTC----CATCTCTACAGCCGCCGAGCTCGGCAAGGG

>Diaporthe_ueckerae_FAU_656

?AGAAGGAAGGTTAGTAAATAT--CACTAC---------ATGTGCTGCTTG--CGCT--CCA-T-AT-----CCTTCCACATGCAC-------ATGGGAATCGTTG--CCGTG----CG-C-TGTCTGCGACGCTTCGTCACACCC----GTCAAGGCA-TTTTCACCCCTCCCTCTGGA----TTTTCCATT---TTCAGTGC-------------------GGGTGCGGGGTGCGCTTATCAGG----CCGCTTATCTCACA---CATCAAA----------CCCTGTC----GCACCTCTGCCACCCTTC--------ACCAGTG-ACACACC---------------------------------------------------------------GAAAATCATGCTGACTT------TTTCTACACAGCCGCCGAGCTGGGTAAGGG

>Diaporthe_unshiuensis_CGMCC3_17569

GAGAAGGAAGGTTAGTAAATAC--CACCAC---------ACCTGCAGCTTG--CGCT--CCA-T-AT-----CCCTCTATATGCAC-------ATAGAAACCGTTG--GCGTG----CG-C-TGTCTGCGACGCTCCGTCACACCT----GTAAAGGCA-TTTTCACCCCTCCCTCTGGA----TTTTCCATT---TTCAGTGC-------------------GGGTGCGGGGTGCGCTTATCAGG----CCGCTTATCTCACA---CATCAA-----------CCCTGTC----GCACCTTTACCACTGTTC--------GCCAGTG-ACACACC---------------------------------------------------------------CAAAATCATGCTGACTC------TTTCTACACAGCCGCCGAGCTGGGTAAGGG

>Diaporthe_vexans_CBS_127_14

GAGAAGGAAGGTTAGTAAACAT--CACCCA---------GCCTGCAGCTTG--AACCCACAG-T-CA-----CTCCATATATGCAC-------ATGAAAATCGTTT--G-GCG----CGAC-ACACTGCGAGGCTTCGTCATACCT----GTCAAGGCA-TTTTCACCCCTCTCTCTGGA----TTTTCCATT---TTCAGTGC-------------------GGGTGCGGGGTGTGCTTATCAGG----CCGCTTATCTCTCACC-AAACAAAA---------CCCCGTC----AAACCT--GCCACTCTTC--------ACCAGTG-CCACCCC---------------------------------------------------------------GAAAATCATGCTGACTT------TTTCTACACAGCCGCCGAGCTGGGTAAGGG

>Diaporthe_yunnanensis_CGMCC_3_18289

GAGAAGGAAGGTTAGTAAACAT--CATGA----------GTTCGCTGCATA--CACT--CCC-T-TG-----CCCTCCATGTGCAT-------CTCAGAATCATTG--GCGCG----TGGC-CATCCGCGAGGCTTCGTCACATCC----GTCAAGGCA-TTTTCACCCCTCGCTCTGGA----TTTTCCATT---TTCAGTGC-------------------GGGTGCGGGGTGTGCTTATCAGG----CGGCTTATCTCTTACA--TCTACAA---------CCCTGTT----GCATTA--ACCACTCCAT--------CCCGCTG-CCTCCACCAACACC--------------------------------------------------------GGAATTCATGCTGACCATT-GTTTTTGAAAACAGCCGCCGAGCTTGGTAAGGG

>Phomopsis_glabrae_SCHM_3622

????????????????????????????????????????????????????????????????????????????????????????????????????????????????????????????????????????????????????????????????????????????????????????????????????????????????????????????????????????????????????????????????????????????????????????????????????????????????????????????????????????????????????????????????????????????????????????????????????????????????????????????????????????????????????????????????????

>Phomopsis_micheliae

????????????????????????????????????????????????????????????????????????????????????????????????????????????????????????????????????????????????????????????????????????????????????????????????????????????????????????????????????????????????????????????????????????????????????????????????????????????????????????????????????????????????????????????????????????????????????????????????????????????????????????????????????????????????????????????????????

>Phomopsis_vitimegaspora_STE_U2675

????????????????????????????????????????????????????????????????????????????????????????????????????????????????????????????????????????????????????????????????????????????????????????????????????????????????????????????????????????????????????????????????????????????????????????????????????????????????????????????????????????????????????????????????????????????????????????????????????????????????????????????????????????????????????????????????????

>Diaporthe_subellipicola_KUMCC_17_0153

??GAAGGAAGGTTAGTAAACAT--CGCCCA---------ACGGGCACCTTG--ACCT--CCA-C-AT-----CACCCCATACGCCC---------AGAACTCGTTG--GCGCG----CG-C-TGTCTACGAGGCTTCGTCACACCT----GTCAAGGCA-TTTTCACCCCTCCCTCTGGA----TTTTCCATT---TTCAGTGC-------------------GGGTGCGGGGTGCGCTTATCAGG----CCGCTTATCTTTCA---CACCAAAA---------CCCTGTC----GCACCTTACCTCTCTTTC--------ACCAGTG-ACGCACCAACAGCATCACCTTCATTCCCA--TCTTTTGCTTGAGGGAGCTTTTCAACTCGCTC-------GAAAATGATGCTGACTC------TTTCTGAACAGCCGCCGAGCTGGGCAAGGG

>Diaporthe_masirevicii_BRIP_57892a

????AGGAAGGTTAGTAAACAT--CGCCCA---------ACGGGCACCTTG--ACCT--CCA-CAAT-----CACCCCATACGCCC---------AGAACTCGTTG--GCGCG----CG-C-TGTCTGCGAGGCTTCGTCACACCT----GTCAAGGCA-TTTTCACCCCTCCCTCTGGA----TTTTCCATT---TTCAGTGC-------------------GGGTGCGGGGTGCGCTTATCAGG----CCGCTTATCTTTCA---CACCAAAA---------CCCTGTC----GCACCTTACCTCTCTTTC--------ACCAGTG-ACGCACC---------------------------------------------------------------GAAAATGATGCTGACTC------TTTCTGAACAGCCGCCGAGCTGGGCAAGGG

>Diaporthe_sojae_CBS_139282

NAGAAGGAAGGTTAGTAAACAT--CGCCCA---------ACGGGCACCTTG--ACCT--CCA-C-AT-----CACCCCATACGCAC---------AGAACTCGTTG--GCGCG----CG-C-TGCCTGCGAGGCTTCGTCACACCT----GTCAAGGCA-TTTTCACCCCTACCTCTGGA----TTTTCCATT---TTCAGTGC-------------------GGGTGCGGGGTGCGCTTATCAAG----CCGCTTATCTCTCA---CACCAAAA---------CCCTGTC----GCACCTTAC--CTCTTCC--------ACCAGTG-ACGCACC---------------------------------------------------------------GAAAATGATGCTGACTC------TTTCTGAACAGCCGCCGAGCTGGGTAAGGG

>Diaporthe_sambucusii_CFCC_51986

NNNNNGGAAGGTCAGTAAACAT--TACAAA---------CTCCACAACATC--AACC--CCAA--AGTC---ATTCCAGTGCACAT-------CGCGCGATCATCG--GCGCG----CGAC-CGTCTGCGCCGCTTCGT--CACTC----GTCACAGCA-TTTTCACCCCTCGTTCTGGA----TTTTCCACT---TTCAGTGC-------------------GGGTGCGGGGTGTGCTTATCAGG----CCGCTTATCGCTTGCG-TTCCAAAA---------CCCTGCTGGGGGCATCA--ACCACTCTTT--------GCCGCTG-CCCACATTAACGCCAACGCCATCAATCTCATCACCCCACCCCCTCTGACAAGACAAACCCTGAG------GAAAACCATGCTGACTCGC----TTTCCATACAGCCGCCGAGCTGGGTAANNN

>Diaporthe_vochysiae_LGMF1583

???????????????????????????????????????????????????????CT--CCA-T-AT-----CCTTCCACATGCAC-------ATGGGAATCGTTG--CCGTG----CG-C-TGTCTGCGACGCTTCGTCACACCC----GTCAAGACA-TTTTCACCCCTCCCTCTGGA----TTTTCCATT---TTCAGTGC-------------------GGGTGCGGGGTGCGCTTATCAGG----CCGCTTATCTCACA---CATCAAA----------CCCTGTC----GCACCTCTGCCACTCTTC--------ACCAGTG-ACACACCAATAGCGTCAC-TTAATTCCCA--TTTGTTGCTCCAGGAAGCTTTTTGAATCGTTC-------GAAAATCATGCTGACTT------TTTCTACACAGCCGCCGAGCTGGGTAAGGG

>Diaporthe_endophytica_CBS_133811

GAGAAGGAAGGTTAGTAAACAT--CGCCCA---------ACGGGCACCTTG--ACCT--CCA-C-AT-----CACCCCATACGCCC---------AGAACTCGTTG--GCGCG----CG-C-TGTCTACGAGGCTTCGTCACACCT----CTCAAGGCA-TTTTCACCCCTCCCTCTGGA----TTTTCCATT---TTCAGTGC-------------------GGGTGCGGGGTGCGCTTATCAGG----CCGCTTATCTTTCA---CACCAAAA---------CCCTGTC----GCACCTTACCTCTCTTTC--------ACCAGTG-ACGCACC---------------------------------------------------------------GAAAATGATGCTGACTC------TTTCTGAACAGCCGCCGAGCTGGGTAAGGG

>Diaporthe_phaseolorum_CBS_113425

GAGAAGGAAGGTTAGTAAACAT--CGCCCA---------ACGGGCACCTTG--ACCT--CCA-C-AT-----CACCCCATACGCAC---------AGAACTCGTTG--GCGCG----CG-C-TGCCTGCGAGGCTTCGTCACACCT----GTCAAGGCA-TTTTCACCCCTCCCTCTGGA----TTTTCCATT---TTCAGTGC-------------------GGGTGCGGGGTGCGCTTATCAGG----CCGCTTATCTCTCA---CACCAAAA---------CCCTGTC----GCACCT-ACCTCTCTTCC--------ACCAGTG-ACGCACC---------------------------------------------------------------GAAAATGATGCTGACTC------TTTCTGAACAGCCGCCGAGCTGGGTAAGGG

>Diaporthe_caliensis_STMA_22040

GAGAAGGAAGGTTAGTAAACAT--CGCCCA---------ACGGGCACCTTG--ACCT--CCA-C-AT-----CACCCCATACGCAC---------AGAACTCGTTG--GCGCG----CG-C-TGCCTGCGAGGCTTCGTCACACCT----GTCAAGGCA-TTTTCACCCCTCCCTCTGGA----TTTTTCATT---TTCAGTGC-------------------GGGTGCGGGGTGCGCTTATCAGG----CCGCTTATCTCTCA---CACCAAAA---------CCCTGTC----GCACCTTACCTCGCTTCC--------ACCAGTG-ACGCACCAACAGCATCACCTTCATTCCCA--TCTGTTGCTCGGGGGAGCTTTtCGACTCGCTT-------GAAAATGATGCTGACTC------TTTCTGAACAGCCGCCGAGCTGGGTAAG??

**Alignment of the *tub2* sequences used in the phylogenetic study**

>Diaporthe_acaciarum_CBS_138862

GTCGGC-CCTTGCTGTGCTCTCGCA-CCCTCCTCTGC----CCCTGAGCCTCAGGC----TACCCCACCATCGCGACCACACCCACGG--TCGGGCCC--AAAACACCACCAGCACCCTGCGATGAGCACCCAAATG-CGTTTGGAAGACGCGTCAGATTGCTAACGTGAACTTT-TTCTCGCCCACAGGTTCACCTTCAGACCGGCCAATGCGTAAGTTGCCTCCTGTCAAC-ACC-GCCCGACCTTATCGCCA-CCCATAGCTGACACGTTTCCCAGGGTAACCAAATCGGTGCTGCTTTCTGGTGCGTC------------------------CCAG--CTCCAG---CCCCGAGC-------------------------------CTCCCACCACG-ATGCTCGAC-------GCGCGACA-AGACTAGCTCGCAACATCG-AT----ACTGATCTCGTCTCGTTAGGCAAACCATCTCTGGCGAGCACGGTCTCGACAGCAATGGCGTGTATGTACCTCCTGTTCCC--TGGCAG-CCG-----ATCTCGTC--CTCTCCTCC---GGCTTGGCACTGATGATCGCACAGTTACAACGGCACTTCCGAGCTCCAGCTCGAGCGCATGAACGTCTACTTCAACGAGGTAAGC----CTACG-GCCACGTCTTC------------------AA-TCCAAA-TTTGA-C----------CGTCT-CG-GC-ATGG-TTTACTGCCGCCGC----CAGG----GCCTTGCTAACGCG-CTCTCGCCC-AGGCCTCCGGCAACAAGTATGTGCCTCGCGCCGTCCTCGTCGATCTCGAGCCTGGTACCATGGACGCCGTC

>Diaporthe_acericola_MFLUCC_17_0956

NNNNNNNNNNNNNNNNNNNNNNNNNNNNNNNNNNNNNNNNNNNNNNNNNNNNNNNNNNNNNNNNNNNNNNNNNNNNNNNNNNNNNNNNNNNNNNNNNNNNNNNNNNNNNNNNNNNNNNNNNNNNNNNNNNNNNNNNNNNNNNNNNNNNNNNNNNNNNNNNNNNNNNNNNNNNNNNNNNNNNNNNNNNNNNNNNNNNNNNNNNNNNNNNNNNNNNNNNNNNNNNNNNNNNNNNNNNNNNNNNNNNNNNNNNNNNNNNNNNNNNNNNNNNNNNNNNNNNNNNNNNNNNNNNNNNNNNNNNNNNNNNNNNNNNNNNNNNNNNNNNNNNNNNNNNNNNNNNNNNNNNNNNNNNNNNNNNNNNNNNNNNNNNNNNNNNNNNNNNNNNNNNNNNNNNNNNNATCGCG--GTCTCGAC-------GCGCGATG-ACAGGACCTCGCAACATCG-TT----ATTGACTTCGACT-TTTAGGCAAACCATCTCTGGCGAGCACGGCCTCGACAGCAATGGCGTGTATGTACCTCCCATTCCC--TACTCG-TCG-----GTCTCGTC-------CGCC---GGCTTGGCACTGACAGCTTCACAGTTACAACGGCTCTTCTGAGCTCCAGCTCGAGCGCATGAACGTCTACTTCAACGAGGTCAGT----CCTCG-ATATTTTTATT------------------GC--------ACCCA-C----------GATCTCCA-AA-ATTG-CCTTGTGTTGTCGT----TTGG----ACTTTGCTGACACC-TTATCGTCC-AGGCTTCCGGCAACAAGTATGTGCCTCGCGCTGTCCTCGTCGATCTCGAGCCCGGTACCATGGACGCCGTC

>Diaporthe_alangii_CFCC_52556

NNNNGC-CCATGC--TGCTTTCGCA----TCCTCTGC----CCCTGAACCTCAGGC----TACCCCACCATCGCGACCACACCCACAG--TCAGGCCTCAAAAACACCATCAACACCCTGGGAAAGGCCACCCAGATGCTCTCAAAAGACGCGTCGGATTGCTAACATGGACTTT-TTCTTGCCCACAGGTTCACCTCCAGACTGGCCAATGCGTAAGTTGCTTCCTGTCAAC-ACCG-CCCGACCTTATCGCCA-CCTCTAGCTGACACGTTTCCCAGGGTAACCAAATCGGTGCTGCTTTCTGGTGCGTC------------------------CCAG--CTCCAG---CTCAGAGT-------------------------------CGACCACCGCG-ACAATCGAC-------GAGCGACA-ACAGTAGCTCGTAGCATTG-AT----ACTGACATCGGCTC-CTAGGCAAACCATCTCTGGCGAGCACGGCCTCGACAGCAATGGCGTGTATGCACCTCCTATTCCC--TGCCTA-CTG-----GTCTCGTCCTCTCTCCTAC---GCCTTGGCACTGACAATGGCACAGCTACAACGGCACTTCTGAGCTCCAGCTCGAGCGCATGAACGTCTACTTCAACGAGGTAAGC----CAAAG-CCCACGTTGTC------------------AA-TCCGGA-TTTGA-C----------CACCTGCA-GC-ACAA-TCCCTTGCCACCGC----CAAG----GCCTAGCTAACGCG-TTATCGTCC-AGGCCTCCGGCAACAAGTATGTGCCTCGCGCCGTCCTCGTCGATCTCGAGCCCGGTACCATGGATGCCGNN

>Diaporthe_ambigua_CBS_114015

GTCGGC-CCATGC--TGCTTTCGCA-TCCTCCTCTGC----CCCTGAACCTGAGGC----TACCCCACCATCGCGACCACACCCACGG-TTCAAACCTC-AAAACACCATCAACACCCTGGGAAGAGCACCCAGATG-CACTCGGAAGACGCGTCAGATTGCTAACATGAACTTT-TTCTCGCCCACAGGTTCACCTTCAGACCGGCCAATGCGTAAGTTGCTCCCTGTCAAC-ACCACCCGGACCTTATCGCCA-CCTGTAGCTGACACGTTTCCCAGGGTAACCAAATCGGTGCTGCTTTCTGGTGCGTC------------------------CCAG--CTCCAG---CTCCAAGT-------------------------------CTACAACCGCG-ACACTCGAC-------GCGCGACA-ACACTAGCTCGGGGCATCA-TT----ACTGACCTCAGCTCT-TAGGCAAACCATCTCTGGCGAGCACGGTCTCGACAGCAATGGCGTGTACGTACCTCGTATCCCC--TGCCCA-CTG-----GTCTCGTC--CTCTCCCTC---GGCTTGGCACTGACAACTGCACAGTTACAACGGCACTTCCGAGCTCCAGCTCGAGCGCATGAACGTCTACTTCAACGAGGTAAGT----CAACA-GCCACGTCGTC------------------AA-TTCAAA-TTTGA-C----------AACCTACG-GC-ATGG-TTTCCCGCCGTCGC----CAAG----GCCTTGCTAACGCATTTATCGCCC-AGGCCTCCGGCAACAAGTATGTGCCTCGCGCCGTCCTCGTCGATCTCGAGCCCGGTACCATGGACGCCGTC

>Diaporthe_amygdali_CBS_126679

GTCGGC-CCATGGC-TGCTTTCGCA----TCCTCTGC----CCCTGAGCC----------TACCCCACCATCGCGACCACACCCACGA--TCGGGCCTC-AAAACACCACAAATACCCTGAAACAAGCACTTCCATG--CCTTCGAAGACGCGTCAGATTGCTAACATGGCCTTT-TTCTCGCCCACAGGTTCATCTCCAGACCGGCCAATGCGTAAGTTGCTCCTGTCAACACACC-ACCGCACCTTATCGCCG-CCTGTAGCTGACACGTTTCCCAGGGTAACCAAATCGGTGCTGCTTTCTGGTGCGTC------------------------CCAG--CT----------------------------------------------CCATCACCGCG-ATACTCGAC-------GCGCGACA-ACACGACCTCGCAACATCC-TT----ACTGACCTCGACTC-GTAGGCAAACCATCTCTGGCGAGCACGGCCTCGACACCAATGGCGTGTATGCACCTCCTATTCCA--TGCCCA-TCA-----ATCTCGGC--CT---CGG----GGATTGGCACTGACAATTGCACAGCTACAACGGCACTTCCGAGCTCCAGCTCGAGCGCATGAACGTCTACTTCAACGAGGTAAGT----CAATC-ACCATGTCATG------------------GC-GTTAAC----GA-G----------CCCCCAAC-AC-GATG-CCTTCTTTTGTCGC----TTGG----GCTTTGCTGACCGC-TTATCGCCCTAGGCCTCCGGCAACAAGTATGTTCCCCGCGCCGTCCTCGTCGATCTCGAGCCCGGTACCATGGACGCCGTC

>Diaporthe_angelicae_CBS_111592

GTCGGCCCTTTGCTGTGCTCTCGCA-TCCTCCTCTGC---CCCCTGAGCCTCAGGC----TACCCCACCATCGCGACCACACCCACGT--TCAGGCCTC-AAAACACCACCAGCACCCTGCGAAGTGCACCCAGATG-CCCTTGGAAGACGCGTCAGATTGCTAACATGGACTTTTCTCTCTCCTACAGGTTCACCTTCAGACCGGCCAATGCGTAAGTCGCCTCCTGTCAAC-ACCGCCGGGACCTTATCGCCA-CCCGTAGCTGACACGTTTCCCAGGGTAACCAAATCGGTGCTGCTTTCTGGTGCGTC------------------------CCAG--CTCCAG---CTCCAGCTCCAGCTCCAGCCCAAGCTCAACCAAAACCTGCCACGGCCGCG-ACATTCGAC-------ACGCGACA-AGATTAGCTCGCAACATCT-TTAT--ACTGACCTCGGCTGTTTAGGCAAACCATCTCTGGCGAGCACGGTCTCGACAGCAATGGCGTGTATGGACCTCCTATTCCCC-TGCCCA-CTG-----ATCTCGTC--CTCCCCTCC---GGCTTGGCACTGATGTTTGCACAGTTACAACGGCACTTCTGAGCTCCAGCTCGAGCGCATGAACGTCTACTTCAACGAGGTGAGT----CAAAATGCCACGTCTTC------------------AA-TTCAAG-TTTGA-G----------CCTTCTCG-GC-ATGA-TTTCCTGCCGCCGC----AAAAGA--CCTTTACTGACGCG-CTTTCGCCC-AGGCCTCCGGCAACAAGTATGTGCCCCGCGCCGTCCTCGTCGATCTCGAGCCCGGTACCATGGACGCCGTC

>Diaporthe_arctii_CBS_136_25

GTCGGCCCTTTGCTGTGCTCTCGCA-TCCTCCTCTGCTGCCCCCTGAGCCTCAGGC----TACCCCACCATCGCGACCACACCCACGT--TCAGGCCTC-AAAACACCACCAGCGCCCTGCGAAGTGCACCCAGATG-GCCTTGGAAGACGCGTCAGATTGCTAACATGGACTTTGTTCTCTCCTACAGGTTCACCTTCAGACCGGCCAATGCGTAAGTTGCCTCCTGTCAAC-ACCGCCGAGACCTTATCGCCA-CCCGTAACTGACACGTTTCCCAGGGTAACCAAATCGGTGCTGCTTTCTGGTGCGTC------------------------CCAG--CTCCAG---CTCCAGCTCCAGTTCCAGCTCAAGCT----CCAAGCCTGCCACGGCCGCG-ACATTCGAC-------ACGCGACA-AGATTAGCTCGCAACACCT-TTAT--ACTGACCTCGGCTGTTTAGGCAAACCATCTCTGGCGAGCACGGTCTCGACAGCAATGGCGTGTATGCACCTCCTATTCCCC-TGCCCA-CTG-----ATCTCGTC--CTCCCCTCC---GGCTTGGCACTGATGTTTGCACAGTTACAACGGCACTTCTGAGCTCCAGCTCGAGCGCATGAACGTCTACTTCAACGAGGTGAGT----CGAAATGCCACGTTTTC------------------AA-TTCAAG-TTTGA-G----------CCTTCTCG-GC-ATGA-TTTCCTGCCGCCGC----AAAGA---CCTTTACTGACGCG-CTTTCGCCC-AGGCCTCCGGCAACAAGTATGTGCCTCGCGCCGTCCTCGTCGATCTCGAGCCCGGTACCATGGACGCCGTC

>Diaporthe_arezzoensis_MFLU_19_2880

NNNNNNNNNNNNNNNNNNNNNNNNNNNNNNNNNNNNNNNNNNNNNNNNNNNNNNNNNNNNNNNNNNNNNNNNNNNNNNNNNNNNNNNNNNNNNNNNNNNNNNNNNNNNNNNNNNNNNNNNNNNNNNNNNNNNNNNNNNNNNNNNNNNNNNNNNNNNNNNNNNNNNNNNNNNNNNNNNNNNNNNNNNNNNNNNNNNNNNNNNNNNNNNNNNNNNNNNNNNNNNNNNNNNNNNNNNNNNNNNNNNNNNNNNNNNNNNNNNNNNNNNNNNNNNNNNNNNNNNNNNNNNNNNNTCGGTGCTGCTTTCTGGTGCGTC------------------------CCAG--CTCCAG---CTCCGAGC-------------------------------CTGCCACCGCG-ACGCTCGAC-------GC-CGGCA-ACACTAGCTCGCAACATCG-TTCCTGACTGACCTCGGCTCTTTAGGCAAACCATCTCTGGCGAGCACGGTCTCGACAGCAATGGCGTGTATGCACCTCCTATTCCC--TGCCCA-CTG-----ATCTCGTC--CTCCCCTCC---GGCTTGGCACTGATGATTATACAGTTACAACGGCACTTCCGAGCTCCAGCTCGAGCGCATGAGCGTCTACTTCAACGAGGTATGT----CAACA-GCCACGTCGTC------------------AA-TTCAAA-CTTGA-C----------CGTCT-CG-GCAATGG-TTCGCTGCCGCCGC----AAAT----GCCTTGCTAACGCG-TCTTCGCCC-AGGCCTCCGGCAACAAGTATGTGCCTCGCGCCGTCCTCGTCGATCTCGAGCCCGGTACCATGGACGCCGTC

>Diaporthe_batatas_CBS_122_21

GTCGGC-CCATGCTGTGCTCTCGCA-TCCTCCTCTGC----CCCTGAGCCTAAGGC----TACCCCACCATCGCGACCACACCCACCG--TCGGGCCTC-AAAACACCACCAGCTGCCTGCGAAAAGCACCCAGGTG-CCCTTGGAACACGCGTCAGATTGCTAACGTGACCTTT-TTCTCGCCCACAGGTTCACCTCCAGACCGGCCAATGCGTAAGTTGCCTCCTGTCAACAACC-GCCCGACCTTATCGCCA-CCCGTAGCTGACACGTTTCCCAGGGTAACCAAATCGGTGCTGCTTTCTGGTGCGTC------------------------GCAGCTCTCCAG---CTCCAAGTC------------------------------CTACCACCGCGACGACTCGAC-------ACGCGCGATAGGCGAGCTCGAAGCATCG-GT----ACTGACCTCGGCTGTTTAGGCAAACCATCTCTGGCGAGCACGGTCTCGACAGCAATGGCGTGTATGCACCTCCTAATCCC---------CTA-----CCCTCGTC--CTCTCCTCC---GCCTTGGCACTGACGATCGCACAGTTACAACGGCACTTCCGAGCTCCAGCTCGAGCGCATGAACGTCTACTTCAACGAGGTAAGT----CAACA-GCCACGTCGTC------------------AA-TCCAAA-CTTGA-C----------CGCCT-CG-GC-GTGG-TCAACTGCCGCCGC----CAAG----CCCTTGCTAACGCG-TTTTCACCC-AGGCCTCCGGCAACAAGTATGTTCCTCGCGCCGTCCTCGTCGATCTCGAGCCCGGTACCATGGACGCCGTC

>Diaporthe_beilharziae_BRIP_54792

GTCGGC-CCTTGCTGTGCTCTCGCA-TTCTCCTCTGC----CCCTGAGCCTCAGGC----TACCCCACCATCGCGACCACACCCACGG--TCGGGCTC--AAAACACCACCAGCACCCTGCGAAGAGCACCCAGATG-CGTTTTGAAGACGCGTCAGATTGCTAACATGAACTTT-TTCTTGCCCACAGGTTCACCTTCAGACCGGCCAATGCGTAAGTTGCCTCCTGTCAAC-ACC-GCCCGACCTTATCGCCACCCCGTAGCTGACACGTTTCCCAGGGTAACCAAATCGGTGCTGCTTTCTGGTGCGTA------------------------CCAG--CTCCAG---CTCCGAGC-------------------------------CTGCCACTGCG-ATGCTCGAC-------GCGCGACA-AGACCACCTCCTAGCATCG-TT----ATTGACCTCGTCTCTTTAGGCAAACCATCTCTGGCGAGCACGGTCTCGACAGCAATGGCGTGTATGTACCTCCTATTCCC--GGCCCA-CCG-----ATCTCGTC--CTCTCCTCC---GGCTTGGCACTGATGATCGCACAGTTACAATGGCTCTTCCGAGCTCCAGCTCGAGCGCATGAACGTCTACTTCAACGAGGTAAGC----CTACG-GTCACGTCTTC------------------GA-TCCAAA-TTTGA-C----------CGTCT-CG-GC-ATGG-TTTACTGCCGCCGC----CAGG----GCCTTGCTAACGCG-CTCTCGCCC-AGGCCTCCGGCAACAAGTATGTGCCTCGCGCCGTCCTCGTCGATCTCGAGCCTGGTACCATGGACGCCGTC

>Diaporthe_biguttulata_ICMP20657

NNNNNNNNNNNNNNNNNNNNNNNNNNNNNNNNNNNNNNNNNNNNNNNNNNNNNNNNNNNNNNNNNNNNNNNNNNNNNNNNNNNNNNNNNNNNNNNNNNNNNNNNNNNNNNNNNNNNNNNNNNNNNNNNNNNNNNNNNNNNNNNNNNNNNNNNNNNNNNNNNNNNNNNNNNNNNNNNNNNNNNNNNNNNNNNNNNNNNNNNNNNNNNNNNNNNNNNNNNNNNNNNNNNNNNNNNNNNNNNNNNNNNNNNNNNNNNNNNNNNNNNNNNNNNNNNNNNNNNNNNNNNNNNNNNNNNNNNNNNNNNNNNNNNNNNNNNNNNNNNNNNNNNNNNNNNNNNNNNNNNNNNNNNNNNNNNNNNNNNNNNNNNNNNNNNNNNNNNNNNNNNNNNNNNNNNNNNNNNNNNNNNNNNNNNNNNNNNNNNNNNNNNNNNNNNNAGCTCGCAGCATCG-TT----ACTGACATCTGCTTTC-AGGCAAACCATCTCTGGCGAGCACGGCCTCGACAGCAATGGCGTGTATGCACCTCCTATTCCC--TGCCCA-TTG-----ATCTCGTC--CTCTCCTCC---GGCTTGGCACTGACAATTGCACAGTTACAACGGCACTTCCGAGCTCCAGCTCGAGCGCATGAACGTCTACTTCAACGAGGTAAGT----CAACA-GCCACGTCGTC------------------AA-TTCGAG-TCTAT-C----------CATCTACG-GC-ATGG-TTTCCTGCCGCCGC----CAAG----GTCTTGCTAACGCG-TTATCTCCC-AGGCCTCCGGCAACAAGTATGTGCCTCGCGCTGTCCTCGTCGATCTCGAGCCCGGTACCATGGACGCCGTC

>Diaporthe_breyniae

?????????????????????????????????????????????????????????????????????????????????????????????????????????????????????????????????????????????????????????????????????????????????????????????????????????????????????????????????????????????????????????????????????????????????????????????????????TGCTGCTTTCTGGTGCGTC------------------------GCAGCTCTCCAG---CTCCAAGC-------------------------------CTACCACCGCG-ACCCTCGAC-------GCGCGACA-AGGCGAGCTCGAAGCATCG-AT----ACTGACCTCGGTTCTTTAGGCAAACCATCTCTGGCGAGCACGGTCTCGACAGCAATGGCGTGTATGCACCTCCTATTCCC--TGCCCG-TGG-----CCCTCGTC--CTCTCCTCC---GGCTTGGCACTGATGATCGCACAGTTACAACGGCACTTCCGAGCTCCAGCTCGAGCGCATGAACGTCTACTTCAACGAGGTCAGT----CAATA-GCCACGTTGTC------------------AA-TTCAAA-TTTGA-G----------CCTCT-CG-GC-ATGG-TCAACTGCCGCCGC----CAAG----CCCTTGCTAACGCG-TTTTCGCCC-AGGCCTCCGGCAACAAGTATGTGCCCCGCGCCGTCCTCGTCGATCTCGAGCCCGGTACCATGGACGCCGTC

>Diaporthe_camporesii_JZB320143

NNNNNNNNNNNNNNNNNNNNNNNNNNNNNNNNNNNNNNNNNNNNNNNNNNNNNNNNNNNNNNNNNNNNNNNNNNNNNNNNNNNNNNNNNNNNNNNNNNNNNNNNNNNNNNNNNNNNNNNNNNNNNNNNNNNNNNNNNNNNNNNNNNNNNNNNNNNNNNNNNNNNNNNNNNNNNNNNNNNNNNNNNNNNNNNNNNNNNNNNNNNNNNNNNNNNNNNNNNNNNNNNNNNNNNNNNNNNNNNNNNNNNNNNNNNNNNNNNNNNNNNNNNNNNNNNNNNNNNNNNNNNNNNNNNNNNNNNNTATGAACATTAGCTC--------------------AGCTACAG--CTCCAG---CTCGAAGC-------------------------------CTACCGCCGCG-ATGCTCGAC-------GCGCGACA-AGACCAGCTCGCAACATTG-TT----ACTGACCTCGGCTCCGTAGGCAAACCATCTCTGGCGAGCACGGCCTCGACAGCAATGGCGTGTATGCACCTCCTATCCCC--TGCCCA-CTG-----ATCTCGTC--CTGTCCTCC---GGCCTGGCACTGATGATTGCACAGCTACAACGGCACTTCCGAGCTCCAGCTCGAGCGCATGAACGTCTACTTCAACGAGGTATGT----CAATG-GCCACGTCGTC------------------AA-TCCAGA-TTTGC-C----------CATCTGCT-GC-ATGG-TGTGCTGCTGTAGC----CAAA----GCCCTCCTAACGCG-TTATCGCCC-AGGCTTCCGGCAACAAGTATGTGCCCCGCGCTGTCCTCGTCGACCTCGAGCCCGGTACCATGGACGCCGTC

>Diaporthe_caryae_CFCC_52563

NNNNGC-CCTTGCTGCGCTCTCGCA----TCCTCTGC----CCCTGAGCCTCAGGC----TACCCCACCATCGCGACCACACCCACGG--TCGGGCTC--AAAACACCACCAGCACCCTGCGATGAGCACCCAGATA-TGTTTGGAAGACGCGTCAGATTGCTAACATGAACTTT-TTCTTGCCCACAGGTTCACCTTCAGACCGGCCAATGCGTAAGTTGCCTCCTGTCAAC-ACCG-CCCGACCTTATCGCCACCCCGTAGCTGACACGTTTCCCAGGGTAACCAAATCGGTGCTGCTTTCTGGTGCGTA------------------------CCAG--CTCCAG---CTCCGAGC-------------------------------CTGCCACCGCG-ACGCTCGAC-------GCGCGACA-AGACTAGCTCGCAACATCT-TT----ACTGACCTCGTCTCTTTAGGCAAACCATCTCTGGCGAGCACGGTCTCGACAGCAATGGCGTGTATGTACCTCCTATTCCC--TGCCCA-CCG-----ATCTCGTC--CTCTCCTCC---GGCTTGGCACTGATGATCGCACAGTTACAACGGCACTTCCGAGCTCCAGCTCGAGCGCATGAATGTCTACTTCAACGAGGTAAGC----CTATG-GCCACGTCTTC------------------AA-TCCAAA-TTTGA-C----------CGTCT-CG-GC-ATGG-TTTACTGCCGCCGC----CAGG----GCCTTGCTAATGCG-CTCTCCCCC-AGGCCTCCGGCAACAAGTATGTGCCTCGCGCCGTCCTCGTCGATCTCGAGCCCGGTACCATGGACGCCGNN

>Diaporthe_celtidis_NCYU_19_0357

NNNNNNNNNNNNNNNNNNNNNNNNNNNNNNNNNNNNNNNNNNNNNNNNNNNNNNNNNNNNNNNNNNNNNNNNNNNNNNNNNNNNNNNNNNNNNNNNNNNNNNNNNNNNNNNNNNNNNNNNNNNNNNNNNNNNNNNNNNNNNNNNNNNNNNNNNNNNNNNNNNNNNNNNNNNNNNNNNNNNNNNNNNNNNNNNNNNNNNNNNNNNNNNNNNNNNNNNNNNNNNNNNNNNNNNNNNNNNNNNNNNNNNNNNNNNNNNNNNNNNNNNNNNNNNNTTTCCCAGGGTAACCAAATCGGTGCTGCTTTCTGGTGCGTC------------------------CCAG--CTCCAG---CTCAGAGT-------------------------------CGACCACCGCG-ACAATCGAC-------GCGCGACA-ACAGTAGCTCGTAGCATTG-TT----ACTGACATCGGCTC-CTAGGCAAACCATCTCTGGCGAGCACGGCCTCGACAGCAATGGCGTGTATGCACCTCCTATCCCC--TGCCTA-CTG-----GTCTCGTC--CTCTCCTCC---GGCTTGGCACTGACAATGGCACAGCTACAACGGCACTTCTGAGCTCCAGCTCGAGCGCATGAACGTCTACTTCAACGAGGTAAGC----CAAAG-CCCACGTTGGC------------------AA-TCCGGA-TTTGA-C----------CATCTGCG-GC-ACAA-TCCCCTGCCACCGC----CAAG----GCCTAGCTAACGCG-TTATCGTCC-AGGCCTCCGGCAACAAGTATGTGCCTCGCGCCGTCCTCGTCGATCTCGAGCCCGGTACCATGGATGCCGTC

>Diaporthe_cerradensis_CMRP4331

NNNNNNNNNNNNNNNNNNNNNNNNNNNNNNNNNNNNNNNNNNNNNNNNNNNNNNNNNNNNNNNNNNNNNNNNNNNNNNNNNNNNNNNNNNNNNNNNNNNNNNNNNNNNNNNNNNNNNNNNNNNNNNNNNNNNNNNNNNNNNNNNNNNNNNNNNNNNNNNNNNNNNNNNNNNNNNNNNNNNNNNNNNNNNNNNNNNNNNNNNNNNNNNNNNNNNNNNNNNNNNNNNNNNNNNNNNNNNNNNNNNNNNNNNNNNNNNNNNNNNNNNNNNNNNNNNNNNNNNNNNNNNNNNNNNNNNNNNNNNNNNNNNNNNNNNNNNNNNNNNNNNNNNNNNNNNNNNNNNNNNNNNNNNNNNNNNNNNNNNNNNNNNNNNNNNNNNNNNNNNNNNNNNNNNCGACGACCGCG-ACGCTCGAC-------GCGCGACA-AGACTAGCTGGTAGCATTG-TT----ACTGACTTCGGCTC-TTAGGCAAACCATCTCTGGCGAGCACGGCCTCGACAGCAATGGCGTGTATGCACCTCCTATCCCC--TGTCCG-ATG-----TCCTCGTC--CTCTCCTCC---GGCTTGGCACTGATGATCGCACAGTTACAACGGCACTTCCGAGCTCCAGCTCGAGCGCATGAACGTCTACTTCAACGAGGTCAGT----CAACA-GCCACGTCGTA------------------AA-TTCGGA-TTTGA-T----------CGTCTACG-GC-ATGG-TTTCCTGCCGCCGC----CAGG----GCCTTGCTAACGCG-TTATCGCCC-AGGCTTCCGGCAACAAGTATGTGCCTCGCGCCGTCCTCGTCGATCTCGAGCCCGGTACCATGGACGCCGTC

>Diaporthe_chimonanthi

??????????????????????????????????????????????????????????????????????????????????????????????????????????????????????????????????????????????????????????????????????????????????????????????????????????????????????????????????????????????????????????????????????????????????????????????????????????????????????????????????????????????????????????????????????????????????????????????????????????????????????????????????????????????????????????????????????????????????????????????????????????????????????????????????????????????????????????????????????????????????????????????????????????????????????????????????????????????????????????????????????????????????????????????????????????????????????????????????????????????????????????????????????????????????????????????????????????????????????????????????????????????????????????????????????????????????????????????

>Diaporthe_chinensis_MFLUCC_19_0101

NNNNNNNNNNNNNNNNNNNNNNNNNNNNNNNNNNNNNNNNNNNNNNNNNNNNNNNNNNNNNNNNNNNNNNNNNNNNNNNNNNNNNNNNNNNNNNNNNNNNNNNNNNNNNNNNNNNNNNNNNNNNNNNNNNNNNNNNNNNNNNNNNNNNNNNNNNNNNNNNNNNNNNNNNNNNNNNNNNNNNNNNNNNNNNNNNNNNNNNNNNNNNNNNNNNNNNNNNNNNNNNNNNNNNNNNNNNNNNNNNNNNNNNNNNNNNNNNNNNNNNNNNNNNNNNNNNNNNNNNNNNNNNNNNNNNNNNNNNNNNNNNNNNNNNNNNNNNNNNNNNNNNNNNNNNNNNNNNNNNNNNNNNNNNNNNNNNNNNC-------------------------------CTACCATCGCG-ACTCTCGAC-------GCACGACA-ACACTAGCTCGCATCATTG-TT----ACTGACCTCTGCTCTTTAGGCAAACCATCTCTGGCGAGCACGGCCTCGACAGCAATGGCGTGTATGTACCTCCTATTCCC--TGCCCA-CTG-----ATCTCGTC--CTGTTCCCC---CGCTTAGCACTGACAATTGCATAGTTACAACGGCACTTCCGAGCTCCAGCTCGAGCGCATGAACGTCTACTTCAACGAGGTCAGT----AAACA-GCCACGTCGCC------------------AA-TTCAAA-TCTGA-C----------CATCTACG-AC-ATGG-TTCCATGTCGCCGC----CAAG----GTCTTGCTAACGCG-TTATCGCCC-AGGCCTCCGGCAACAAGTATGTGCCTCGCGCCGTCCTCGTCGATCTCGAGCCCGGTACCATGGATGCCGTC

>Diaporthe_chromolaenae_MFLUCC_17_1422

??????????????????????????????????????????????????????????????????????????????????????????????????????????????????????????????????????????????????????????????????????????????????????????????????????????????????????????????????????????????????????????????????????????????????????????????????????????????????????????????????????????????????????????????????????????????????????????????????????????????????????????????????????????????????????????????????????????????????????????????????????????????????????????????????????????????????????????????????????????????????????????????????????????????????????????????????????????????????????????????????????????????????????????????????????????????????????????????????????????????????????????????????????????????????????????????????????????????????????????????????????????????????????????????????????????????????????????????

>Diaporthe_cichorii_MFLUCC_17_1023

NNNNNNNNNNNNNNNNNNNNNNNNNNNNNNNNNNNNNNNNNNNNNNNNNNNNNNNNNNNNNNNNNNNNNNNNNNNNNNNNNNNNNNNNNNNNNNNNNNNNNNNNNNNNNNNNNNNNNNNNNNNNNNNNNNNNNNNNNNNNNNNNNNNNNNNNNNNNNNNNNNNNNNNNNNNNNNNNNNNNNNNNNNNNNNNNNNNNNNNNNNNNNNNNNNNNNNNNNNNNNNNNNNNNNNNNNNNNNNNNNNNNNNNNNNNNNNNNNNNNNNNNNNNNNNNNNNNNNNNNNNNNNNNNNNNNNNNNNNNNNNNNNNNNNNNNNNNNNNNNNNNNNNNNNNNNNNNNNNNNNNNNNNNNNNNNNNNNNNNNNNNNNNNNNNNNNNNNNNNNNNNNNNNNNNNNACGACCGCG-ACATTCGAC-------ACGCGACA-AGATTAGCTCGCAACGTCG-TTAT--ACTGACCTCGGCTGTTTAGGCAAACCATCTCTGGCGAGCACGGTCTCGACAGCAATGGCGTGTATGCACCTCCTGTTTCCC-TGCCCA-CTG-----ATCTCGTC--CTTCCCTCC---GGCTTGGCACTGATGTTTGCACAGTTACAACGGCACTTCTGAGCTCCAGCTCGAGCGCATGAACGTCTACTTCAACGAGGTGAGT----CAAAA-GCCACGTCTTC------------------AA-TTCGAG-TTTGA-G----------CCTCCTCG-GC-ATGA-TTTCCTGCCGCCGC----AAAAAG--ACCTTACTGACGCG-CTTTCGCCC-AGGCCTCCGGCAACAAGTATGTGCCTCGCGCCGTCCTCGTCGATCTCGAGCCCGGTACCATGGACGCCGTC

>Diaporthe_cinnamomi_CFCC_52569

NNNNGC-CCATGC--TGCTTTCGCA----TCCTCTGC----CCCTGAGCCTAAGGC----TACCCCACCATCGCGACCACACCCACGG--TCAAGCCTCAAAAACACCATCGACACCCTGGGAAGAGCACCCAGATG-CCCTCGGAAGACGCGTCAGATTGCTAACATGAACTTT-TTCTCGACCACAGGTTCACCTTCAGACCGGCCAATGCGTAAGTTGCTTCCTGTCAAC-ACCA-CCGGACCTTATCGCCA-CCTGTAGCTGACACGTTTCCCAGGGTAACCAAATCGGTGCTGCTTTCTGGTGCGTC------------------------CCAG--CTCCAG---CTCCAAGC-------------------------------CTACCACCGCG-ACTCTCGAC-------GCGCGATA-AGACTAGCTCGCAACATCG-TT----ACTGACCTCGGATCTTTAGGCAAACCATCTCTGGCGAGCACGGCCTCGACAGCAATGGCGTGTATGTACCTCCTATTCCC--TGCCCA-CTG-----ATCTCGTCCTCCCCCCCCCCC-GGCTTGGCACTGACAACTGCACAGTTACAACGGCACTTCTGAGCTCCAGCTCGAGCGCATGAACGTCTACTTCAACGAGGTAAGT----CAACA-GCCACGTCGTC------------------AA-TACAAT-TTTGT-C----------CATCTACT-GC-ATGG-TCTCCTGCCGCCGC----CAAG----GTCTTGCTAACGCA-TTATCGCCC-AGGCCTCCGGCAACAAGTATGTGCCTCGCGCCGTCCTCGTCGATCTCGAGCCCGGTACCATGGACGCCGNN

>Diaporthe_citriasiana_CBS_134240

NNNNNNNNNNNNNNNNNNNNNNNNNNNNNNNNNNNNNNNNNNNNNNNNNNNNNNNNNNNNNNNNNNNNNNNNNNNNNNNNNNNNNNNNNNNNNNNNNNNNNNNNNNNNNNNNNNNNNNNNNNNNNNNNNNNNNNNNNNNNNNNNNNNNNNNNNNNNNNNNNNNNNNNNNNNNNNNNNNNNNNNNNNNNNNNNNNNNNNNNNNNNNNNNNNNNNNNNNNNNNNNNNNNNNNNNNNNNNNNNNNNNNNNNNNNNNNNNNNNNNNNNNNNNNNNNNNNNNNNGGTAACCAAATCGGTGCTGCTTTCTGGTGCGTC------------------------CCAG--CTCCAG---CTCCAAGC-------------------------------CTACCACCGCG-ACTCTTGAC-------GCGCGACA-ACACTAGCTCGCAACATCG-TT----ACTGACCTCGGTTCTTTAGGCAAACCATCTCTGGCGAGCACGGTCTCGACAGCAATGGCGTGTATGCACCTCCTATTCCC--TGCCCA-CTG-----ATCTCGTC--GTCTCCTCC---GGCCTCGCACTGACAATTGCACAGTTACAACGGCACTTCCGAGCTCCAGCTCGAGCGCATGAACGTCTACTTCAACGAGGTAAGT----CAACA-GCCACGTCGTC------------------AA-TACGAT-TTTGT-C----------CATCTACG-GC-ACGG-CCTCCTGCCGCCGC----TAAG----GTCTTGCTAACGCG-TCATCGCCC-AGGCCTCCGGCAACAAGTATGTGCCTCGCGCCGTCCTCGTCGATCTCGAGCCCGGTACCATGGACGCCGTC

>Diaporthe_compacta_LC3083

NNNNNNNNNNNNNNNNNNNNNNNNNNNNNNNNNNNNNNNNNNNNNNNNNNNNNNNNNNNNNNNNNNNNNNNNNNNNNNNNNNNNNNNNNNNNNNNNNNNNNNNNNNNNNNNNNNNNNNNNNNNNNNNNNNNNNNNNNNNNNNNNNNNNNNNNNNNNNNNNNNNNNNNNNNNNNNNNNNNNNNNNNNNNNNNNNNNNNNNNNNNNNNNNNNNNNNNNNNNNNNNNNNNNNNNNNNNNNNNNNNNNNNNNNNNNNNNNNNNNNNNNNNNNNNNNNNNNNNNNNNNNNNNNNNNNNNNNNNNNNNNNNNNNNNNNNNNNNNNNNNNNNNNNNNNNNNNNNNNNNNNNNNNNNNNNNNNNNNNNNNNNNNNNNNNNNNNNNNNNNNNNNNNNNNNNNNNNNNNNNNNNNNNNNNNNNNNNNNNNNNNNNNNNNNNNAGCTCGCAACATTG-TT----ACTGACCTCGGCTCTGTAGGCAAACCATCTCTGGCGAGCACGGCCTCGACAGCAATGGCGTGTATGCACCTCCTATTCCC--TGCCCA-CTG-----ATCCCGTC--CTGTCCTCC---GGCTTGGCACTGATGATTGCACAGTTACAACGGCACTTCCGAGCTCCAGCTCGAGCGCATGAACGTCTACTTCAACGAGGTATGT----CAATG-GCCACGTCGTC------------------AA-TCCAGA-TTTGC-C----------CATCTACT-GC-GCCG-CGTGCTGCCGTAGC----CAAA----GCCTTGCTAACGCG-TTGTCGCCC-AGGCTTCCGGCAACAAGTATGTGCCCCGCGCTGTCCTCGTCGATCTCGAGCCCGGTACCATGGACGCCGTC

>Diaporthe_convolvuli_CBS_124654

GTCGGC-CCATGCTGTGCTCTCACT----TCCTCTGC----CCCTGAGCATCAGGC----TACCCCACCATCGCGACCACACCCACGG--TCGGGCCTC-AAAACACCACCAGCTCCCTGCGACGAGCACCCAGATA-CCCTTGGAACACGCGTCAGATTGCTAACGTGACCTTT-TCCTCGCCCACAGGTTCACCTTCAGACCGGCCAATGCGTAAGTTGCTCCTCGTTAAC-ACC-GCCAGACCTTATCGCCA-CCCGTAGCTGACACGTTTCCCAGGGTAACCAAATCGGTGCTGCTTTCTGGTGCGTC------------------------GCAACTCTCCAG---CTCCAAAC-------------------------------CTACCACCGCG-ACGCTCGAC-------GCGCGATC-AGGCGAGCTCCAAGCATCG-GT----GCTGACCTCGGTTCTTTAGGCAAACCATCTCTGGCGAGCACGGTCTCGACAGCAATGGCGTGTATGCACCCCCTATTCCC--TGCCCA-CTG---------CCCA--GTCCTCTCC---GGCTTGGCACTGATGATCGCACAGTTACAACGGCACTTCCGAGCTCCAGCTCGAGCGCATGAACGTCTACTTCAACGAGGTATGT----CAATA-G-CACGTCGTC------------------AA-TTCAAATTTTGA-C----------CCTCT-CG-GC-ATGC-TTAACCGCCGCCGCC---CAAG----CCATTGCTAACGCG-TCTTCTCCC-AGGCTTCCGGCAACAAGTATGTGCCCCGCGCCGTCCTCGTCGATCTCGAGCCCGGTACCATGGACGCCGTC

>Diaporthe_cucurbitae_DAOM_42078

NNNNNNNNNNNNNNNNNNNNNNNNNNNNNNNNNNNNNNNNNNNNNNNNNNNNNNNNNNNNNNNNNNNNNNNNNNNNNNNNNNNNNNNNNNNNNNNNNNNNNNNNNNNNNNNNNNNNNNNNNNNNNNNNNNNNNNNNNNNNNNNNNNNNNNNNNNNNNNNNNNNNNNNNNNNNNNNNNNNNNNNNNNNNNNNNNNNNNNNNNNNNNNNNNNNNNNNNNNNNNNNNNNNNNNNNNNNNNNNNNNNNNNNNNNNNNNNNNNNNNNNNNNNNNNNNNNNNNNNNNNNNNNNNNNNNNNNNNNNNNNNNNGTGCGTC------------------------CCAG--CTCCAG---CTCCAGCTCCAGTTCCAGCTCAAGCT----CCAAGCCTGCCACGGCCGCG-ACATTCGAC-------ACGCGACA-AGATTAGCTCGCAACACCT-TTAT--ACTGACCTCGGCTGTTTAGGCAAACCATCTCTGGCGAGCACGGTCTCGACAGCAATGGCGTGTATGCACCTCCTATTTCCC-TGCCCA-CTG-----ATCTCGTC--CTCCCCTCC---GGCTTGGCATTGATGTTTGCACAGTTACAACGGCACTTCTGAGCTTCAGCTCGAGCGCATGAACGTCTACTTCAACGAGGTGAGT----CAAAATGCCACGTCTTC------------------AA-TTCAAG-TTTGA-G----------CGTTCTTG-GC-ATGA-TTTCCTGCCGCCGC----AAAGA---CCTTTACTGACGCG-CTTTCGCCC-AGGCCTCCGGCAACAAGTATGTGCCTCGCGCCGTCCTCGTCGATCTCGAGCCCGGTACCATGGACGCCGTC

>Diaporthe_cuppatea_CBS_117499

NNNNNNNNNNNNNNNNNNNNNNNNNNNNNNNNNNNNNNNNNNNNNNNNNNNNNNNNNNNNNNNNNNNNNNNNNNNNNNNNNNNNNNNNNNNNNNNNNNNNNNNNNNNNNNNNNNNNNNNNNNNNNNNNNNNNNNNNNNNNNNNNNNNNNNNNNNNNNNNNNNNNNNNNNNNNNNNNNNNNNNNNNNNNNNNNNNNNNNNNNNNNNNNNNNNNNNNNNNNNNNNNNNNNNNNNNNNNNNNNNNNNNNNNNNNNNNNNNNNNNNNNNNNNNNNNNNNNNNNNNNNNNNNNNNNNNNNNNNNNNNNNNNNNNNNNNNNNNNNNNNNNNNNNNNNNNNNNNNNNNNNNNNNNNNNNNNNNNNNNNNNNNNNNNNNNNNNNNNNNNNNNNNNNNNNNNNNNNNNNNNNNNNNNNNNNNNNNNNNNNNNNNNNNNNNNAGCTCGCAATATCG-TTAT--GCTGACCTCGGCTGTTTAGGCAAACCATCTCTGGCGAGCACGGTCTCGACAGCAATGGCGTGTATGCACCTCCTATTCCC--TGTCCT-CTG-----ATCTCGTC--CTCCCCTCC---GGCTTGGCACTGATGTTTGCACAGTTACAACGGCACTTCCGAGCTCCAGCTCGAGCGCATGAACGTCTACTTCAACGAGGTGAGT----CAAAA-GCCACGTCTTC------------------GA-TTCAAA-TTGGA-G----------CGTTCTCG-GC-ATGA-TTTACTGCCGCCGC----AAAG----ACTCTGCTAACGCG-CTTTCGCCC-AGGCCTCCGGCAACAAGTATGTGCCTCGCGCCGTCCTCGTCGATCTCGAGCCCGGTACCATGGACGCCGTC

>Diaporthe_discoidispora_ICMP20662

NNNNNNNNNNNNNNNNNNNNNNNNNNNNNNNNNNNNNNNNNNNNNNNNNNNNNNNNNNNNNNNNNNNNNNNNNNNNNNNNNNNNNNNNNNNNNNNNNNNNNNNNNNNNNNNNNNNNNNNNNNNNNNNNNNNNNNNNNNNNNNNNNNNNNNNNNNNNNNNNNNNNNNNNNNNNNNNNNNNNNNNNNNNNNNNNNNNNNNNNNNNNNNNNNNNNNNNNNNNNNNNNNNNNNNNNNNNNNNNNNNNNNNNNNNNNNNNNNNNNNNNNNNNNNNNNNNNNNNNNNNNNNNNNNNNNNNNNNNNNNNNNNNNNNNNNNNNNNNNNNNNNNNNNNNNNNNNNNNNNNNNNNNNNNNNNNNNNNNNNNNNNNNNNNNNNNNNNNNNNNNNNNNNNNNNNNNNNNNNNNNNNNNNNNNNNNNNNNNNNNNNNNNNNNNNNAGCTCGCAACATCG-TT----ACTGACCTAGACTCTTTAGGCAAACCATCTCTGGCGAGCACGGTCTCGACAGCAATGGCGTGTATGCAACTCCTATTCCC--TGCCCA-CTG-----ATATCGTC--CTCTCCTCC---GGCCTGGCACTGACAATTGCACAGTTACAACGGCACTTCCGAGCTCCAGCTCGAGCGCATGAACGTCTACTTCAACGAGGTAAGT----CAATA-GCCACGTCGTC------------------AA-TACAAC-TTTGT-C----------CATCTACATGC-ATGG-TCTCCTGCCGCCGC----CGAG----GTCTTGCTAACGCG-TTACCGCCC-AGGCCTCCGGCAACAAGTATGTGCCTCGCGCCGTCCTCGTCGATCTCGAGCCCGGTACCATGGACGCCGTC

>Diaporthe_durionigena_VTCC_930005

??????????????????????????????????????????????????????????????????????????????????????????????????????????????????????????????????????????????????????????????????????????????????????????????????????????????????????????????????????????????????????????????????????????????????????????????????CGGTGCTGCTTTCTGGTGCGTC------------------------GCAGCTCTCCAG---CTCCAAGC-------------------------------CTGCCACCGCGGACCCTCGAC-------GCGCGACC-AGGCGAGCTCGAAGCATCG-AT----ACTGACCTCGGTTCTTTAGGCAAACCATCTCTGGCGAGCACGGTCTCGACAGCAATGGCGTGTATGCACCTCCTATTCCC--TGCCCG-TGG-----CCCTCGTC--CTCTTCTCC---GGCTTGGCACTGATGATCGCACAGTTACAACGGCACTTCCGAGCTCCAGCTCGAGCGCATGAACGTCTACTTCAACGAGGTCAGT----CAATA-GCCACGTCGTC------------------AA-TTCAAA-TTTGA-A----------CCTCT-CG-GC-ATGG-TCAACTGCCGCCGC----CAAG----CCCCTGCTAACGCG-TTTTCGCCC-AGGCCTCCGGCAACAAGTATGTGCCCCGCGCCGTCCTCGTCGATCTCGAGCCCGGTACCATGGACGCCGTC

>Diaporthe_eres_CBS_138594

NNNNNNN????????????????????????????????????????????????????????????????????????????????????????????????????????????????????????????????????????????????????????????????????????????????????????????????????????????????????????????????????????????????????????????????????????????????????????????GGTGCTGCTTTCTGGTGCGTT------------------------CCAG--CTCGAG---CTCCAAGT-------------------------------CCACCGCCGCG-ACGCTTGAC-------ACGCGACA-ATACGACCTCGAAGCATCG-TT----GCTGACCTCGACT-TTTAGGCAAACCATCTCTGGCGAGCACGGCCTCGACAGCAATGGCGTGTATGCACCTCCTATGCCC--TGTCCA-CTG-----ATCTTGAC--CTCTCTTCC---GGCTTGGCACTGACAATCGCACAGTTACAACGGCACTTCTGAGCTCCAGCTCGAGCGCATGAACGTCTACTTCAACGAGGCAAGT----CAATA-ACAGCAC----------------------AA-CATTCA-TCCGA-C----------CATCTCCA-AC-ACGG-TTTACTGCCGTCGC----CCGA----AGTTCGCTAACGCG-TTATCGCCC-AGGCCTCCGGCAACAAGTATGTTCCTCGCGCCGTCCTCGTCGATCTCGAGCCCGGTACCATGGACGCCGTC

>Diaporthe_fici_septicae_MFLU_18_2588

NNNNNNNNNNNNNNNNNNNNNNNNNNNNNNNNNNNNNNNNNNNNNNNNNNNNNNNNNNNNNNNNNNNNNNNNNNNNNNNNNNNNNNNNNNNNNNNNNNNNNNNNNNNNNNNNNNNNNNNNNNNNNNNNNNNNNNNNNNNNNNNNNNNNNNNNNNNNNNNNNNNNNNNNNNNNNNNNNNNNNNNNNNNNNNNNNNNNNNNNNNNNNNNNNNNNNNNNNNNNNNNNNNNNNNNNNNNNNNNNNNNNNNNNNNNNNNNNNNNNNNNNNNNNNNNTTTCCCAGGGTAACCAAATCGGTGCTGCTTTCTGGTGCGTC------------------------CCAG--CTCCAG---CTCAGAGT-------------------------------CGACCACCTCG-ACTCTCGAC-------GCGCGACA-ACACTAGCTCGCATCAACG-TT----ACTGACCTCGGCTCTTTAGGCAAACCATCTCTGGCGAGCACGGTCTCGACAGCAATGGCGTGTATGCACCTCCTATTCCC--TGCCCA-CTG-----ATCTCGTC--CTCTCCCCC---GGCCTGGCACTGACGATTGTACAGTTACAACGGCACTTCCGAGCTCCAGCTCGAGCGCATGAACGTCTACTTCAACGAGGTAAGT----CAACA-GCCACGTCATC------------------AA-TACAAT-TTTGT-C----------CATCTACG-GC-ATGG-TCTCCTGCAGCCGC----CAAG----GTCTTGCTAACGCG-TTATCGCTC-AGGCCTCCGGCAACAAGTATGTGCCTCGCGCCGTCCTCGTCGATCTCGAGCCCGGTACCATGGACGCCGTC

>Diaporthe_fructicola_MAFF_246408

GTCGGC-CCATGCTGTGCTCTCGCA----TCCTCTGC----CCCTGAGCCTGAGGC----TACCCCACCATCGCGACCACACCCATGG--TCGGGCCTC-AAAACACCACCAGCGCCCTGCGAAGAGCACCCAGACG-CTCTTGTAACACGCGTCAGATTGCTAACATGACCTTT-TTCTCGCCCACAGGTTCACCTTCAGACCGGCCAATGCGTAAGTTGCCTCCTGTCAAC-ACC-GCCAGACCTTATCGCCA-CCCGTAGCTGACACGTTTCCCAGGGTAACCAAATCGGTGCTGCTTTCTGGTGCGTC------------------------GCAGCTCTCCAG---CTCCAAGC-------------------------------CTACCACCGCG-ACCCTCAAC-------GCGCGACA-AGGCGAGCTCGAAGCATCG-AT----ACTGACCTCGTTTCTTTAGGCAAACCATCTCTGGCGAGCACGGTCTCGACAGCAATGGCGTGTATGCACCTCCTATTCCC--TGCCCA-CTG-ACTGCCCTGGTC--CTCTCCTCC---GGCTTGGCACTGATGATCGCACAGTTACAACGGCACTTCCGAGCTCCAGCTCGAGCGCATGAACGTCTACTTCAACGAGGTATGT----CAACG-GCCACGTCGTC------------------AA-TTCAAA-TTTGA-C----------CCTCT-CG-GC-GTGGATCAACCGCCGCCGC----CAAG----CCCTTGCTAACGCG-TTTTCCCCC-AGGCTTCCGGCAACAAGTATGTGCCCCGCGCCGTCCTCGTCGATCTCGAGCCCGGTACCATGGACGCCGTC

>Diaporthe_ganjae_CBS_180_91

GTCGGC-CCATC---TGCTCTCGCA-TCCTCCTCTGC----CCCTGAGCCTCAGCC----TACCCCACCATCGCGACCACACTCCCACAGCAGGGCCTC-AAAACATCACCAGCACCTTGTGATGAGCACCCGTGTG-CCCTTGGAACACGCGTCAGATTGCTAACATGGACTTT-TTCTGGCCTGCAGGTTCACCTCCAGACCGGCCAATGCGTAAGTTGCTCCCTGTCAAC-ACCG-CCCGACCTTATCGCCA-CCCGTAGCTGACACGTTTCCCAGGGTAACCAAATCGGTGCTGCTTTCTGGTGCGTCCCAGCTCCAGCTCCAGCTCCAGCTCCAG--CTCCAG---CTCGAGGC-------------------------------CTACCGCCGCG-ATGCTCGAC-------GCGCGACA-AGACCAGCTCGCAACATTG-TT----ACTGACCTAGGCTCTGTAGGCAAACCATCTCTGGCGAGCACGGTCTCGACAGCAATGGCGTGTATGCACCTCCTATTCCC--TGCCCA-CTG-----ATCTCGTC--CTGTCCTCC---GGCTTGGCACTGATGATTGCACAGTTACAACGGCACTTCCGAGCTCCAGCTCGAGCGCATGAACGTCTACTTCAACGAGGTATGT----CAATG-GCCACGTCGTC------------------AA-TCCAAA-TTTGC-C----------CATCTACT-GC-GCGG-CGTGCTGCCGTAGC----CAAA----GCCTTGCTAACGCG-TTGTCGCCC-AGGCTTCCGGCAACAAGTATGTGCCCCGCGCTGTCCTCGTCGATCTCGAGCCCGGTACCATGGACGCCGTC

>Diaporthe_goulteri_BRIP_55657a

NNNNNNNNNNNNNNNNNNNNNNNNNNNNNNNNNNNNNNNNNNNNNNNNNNNNNNNNNNNNNNNNNNNNNATCGCGACCACACCCACAT--TCAGGC-TC-AAAACACCATCAAAACCCTTGGAAGAGCACCCAGGTG-TCCTCTGAAGACGCGTCAGATTGCTAACGTGAACTTT-CTCTCGCCCACAGGTTCACCTCCAGACCGGCCAGTGCGTAAGTTGCTCCCTGTCAAC-ACCACCCGGACCTTATCGCCA-CCTGTAGCTGACACGTTTCCCAGGGTAACCAAATCGGTGCTGCTTTCTGGTGCGTC------------------------CCTG--CTCCAG---TCCCAAGC-------------------------------CTACGACCGCG-ATACTCGAC-------GCGCGAGA-ATACCAGCTCGCATCATCA-TC----ACTGACCTCAGCTC-TTAGGCAAACCATCTCTGGCGAGCACGGTCTCGACAGCAATGGCGTGTATGCACCTCCTATCCCC--CATCCA-CCG-----GTCTCGTC--CACTTCTCC---GGCTTGCCACTGACAATCGCACAGCTACAATGGCACCTCCGAGCTCCAGCTCGAGCGCATGAACGTCTACTTCAACGAGGTAAGT----CTACA-GTCACTTCGTC------------------AA-TCTAAA-TTTGA-T----------CACCTGCG-GCATTGG-TTTGCTGCCGCCGC----CAAG----GCCCTGCTAACGCG-TTCTCGCCC-AGGCCTCCGGCAACAAGTATGTGCCTCGCGCCGTCCTCGTCGATCTCGAGCCCGGTACCATGGACGCCGTC

>Diaporthe_guangdongensis_ZHKUCC20_0014

???????????????????????????????????????????????????????????????????????????????????????????????????????????????????????????????????????????????????????????????????????????????????????????????????????????????????????????????????????????????????????????????????????????????????????GGTAACCAAATCGGTGCTGCTTTCTGGTGCGTC------------------------GCAGCTCTCCAG---CTCCGAGC-------------------------------CTACCACCGCG-ACACCCGAC-------GCGCGACA-AGGCGAGCTCGAAACAGCG-AT----ACTGACCTCGATTCTTTAGGCAAACCATCTCTGGCGAGCACGGTCTCGACAGCAATGGCGTGTATGGACCTCCTATTCCC--TGACTA-CCG-----ACCTCGTC--CTCTCCTCC---GGCTTGGCACTGATGATCGCACAGTTACAACGGCACTTCCGAGCTCCAGCTCGAGCGCATGAACGTCTACTTCAACGAGGCAAGT----CAATA-GCCACGTCGCC------------------AA-TTCAGA-CTTGA-C----------CGTCT-TG-GC-ATGG-TAAACTGCCGCCGC----CAAG----CCCTTGCTAACGCG-TATTCGCCC-AGGCCTCCGGCAACAAGTATGTGCCCCGCGCCGTCCTCGTCGATCTCGAGCCCGGTACCATGGACGCCGTC

>Diaporthe_gulyae_BRIP_54025

GTCGGCCCTTTGCTGTGCTCTCGCA-TCCTCCTCTGCTGCCCCCTGAGCCTCAGGC----TACCCCACCATCGCGACCACACCCACGT--TCAGGCCTC-AAAACACCACCAGCGCCCTGCGAAGTGCACCCAGATG-CCCTTGGAAGACGCGTCAGATTGCTAACATGGACTTCGTTCTCTCCTACAGGTTCACCTTCAGACCGGCCAATGCGTAAGTTGCCTCCTGTCAAC-ACCGCCGAGACCTTATCGCCA-CCCGTAACTGACACGTTTCCCAGGGTAACCAAATCGGTGCTGCTTTCTGGTGCGTC------------------------CCAG--CTCCAG---CTCCAGCTCCAGTTCCAGCTCAAGCT----CCAAGCCTGCCACGGCCGCG-ACATTCGAC-------ACGCGACA-AGATTAGCTCGCAACACCT-TTAT--ACTGACCTCGGCTGTTTAGGCAAACCATCTCTGGCGAGCACGGTCTCGACAGCAATGGCGTGTATGCACCTCCTATTCCCC-TGCCCA-CTG-----ATCTCGTC--CTCCCCTCC---GGCTTGGCACTGATGTTTGCACAGTTACAACGGCACTTCTGAGCTCCAGCTCGAGCGCATGAACGTCTACTTCAACGAGGTGAGT----CAAAATGCCACGTCTTC------------------AA-TTCGAG-TTTGA-G----------CGTTCTCG-GC-ATGA-TTTCCTGCCGCCCC----AAAGA---CCTTTACTGACGCG-CTTTCGCCC-AGGCCTCCGGCAACAAGTATGTGCCTCGCGCCGTCCTCGTCGATCTCGAGCCCGGTACCATGGACGCCGTC

>Diaporthe_guttulata_CGMCC_3_20100

NNNNNNNNNNNNNNNNNNNNNNNNNNNNNNNNNNNNNNNNNNNNNNNNNNNNNNNNNNNNNNNNNNNNNNNNNNNNNNNNNNNNNNNNNNNNNNNNNNNNNNNNNNNNNNNNNNNNNNNNNNNNNNNNNNNNNNNNNNNNNNNNNNNNNNNNNNNNNNNNNNNNNNNNNNNNNNNNNNNNNNNNNNNNNNNNNNNNNNNNNNNNNNNNNNNNNNNNNNNNNNNNNNNNNNNNNNNNNNNNNNNNNNNNNNNNNNNNNNNNNNNNNNNNNNNNNNNNNNNNNNNNNNNNNNNNNNNNNNNNNNNNNNNNNNNNNNNNNNNNNNNNNNNNNNNNNNNNNNNNNNNNNNNNNNNNNNNNNNNNNNNNNNNNNNNNNNNNNNNNNNNCAGCCTGCCACGACCGCG-ACATTCGAC-------ACGCGACA-AGATTAGCTCGCAACATCT-TTAT--ACTGACCTCGGCTGTTTAGGCAAACCATCTCTGGCGAGCACGGTCTCGACAGCAATGGCGTGTATGCACCTCCTATTCCCC-TGCCCA-CTG-----ATCTCGTC--CTCCCCTCC---GGCTTGGCACTGATGTTTGCACAGTTACAACGGCACTTCTGAGCTCCAGCTCGAGCGCATGAACGTCTACTTCAACGAGGTGAGT----CAAAATGCCACGTCTTC------------------AA-TTCAAG-TTTGA-G----------CCTTCTCG-GC-ATGA-TTTCCTGCCGCCGC----AAAAG---ACCTTACTGACGCG-CTTTCGCCC-AGGCCTCCGGCAACAAGTATGTGCCTCGCGCCGTCCTCGTCGATCTCGAGCCCGGTACCATGGACGCCGTC

>Diaporthe_helianthi_CBS_592_81

GTCGGC-CCATGCTGTGCTCTCGCA-TCCTCCTCTGC----CCCTGAGCCTCAACGC---TACCCCACCATCGCGACCACACCCACGG--TCGCGCCTC-AAAACACCACCATCACCCTACGAAGAGCACCCAGATG-CGCTTGAAGGACGCGTCAGATTGCTAACATGAGCTTT-TCCTCGCCTACAGGTTCACCTTCAGACCGGCCAATGCGTAAGTTGCTCCCTGTCAAC-ACG-GCCGGGCCGTTTCGCCA-CCTGTAGCTGACACGTTTCCCAGGGTAACCAAATCGGTGCTGCTTTCTGGTGCGTC------------------CCTGCTCCAG--CTTCTG---CTCCAAGC-------------------------------CTACCACCGCG-ACGCTCGAC-------ACGCGAAA-AGGCTGGCTCGTAGTCTCG-AT----GCTGACCCCGGCTCTTCAGGCAAACCATCTCTGGCGAGCACGGTCTCGACAGCAATGGCGTGTATGTACCTCCTATTCCC--TGACTT-CTA-----AACTCGTC--CTCTCCTCA---AGCTTGCCACTAATGATCGCACAGCTACAACGGCACTTCCGAGCTCCAGCTCGAGCGCATGAACGTCTACTTCAACGAGGTACGC----GAACG-GCCACCTCGTC------------------AA-TTCAGA-TTTGA-C----------CCTCT-CG-GC-ATAATTAAACGGCTGTCGC----CAAG----CCCTTGCTAACGTG-TTTTCGCCC-AGGCTTCCGGCAACAAGTATGTGCCTCGCGCCGTCCTCGTCGATCTCGAGCCCGGTACCATGGACGCCGTC

>Diaporthe_heterostemmatis_SAUCC194_85

??????????????????????????????????????????????????????????????????????????????????????????????????????????????????????????????????????????????????????????????????????????????????????????????????????????????????????????????????????????????????????????????????????????????????????????????????????????????????????????????????????????????????????CTCTCA---GCTC-AGC-------------------------------CTACCACCGCG-ACCCTCGAC-------GCGCGACA-AGGCGAGCCCGAAGCATCG-AT----ACTGACCTCGTTTCTTTAGGCAAACCATCTCTGGCGAGCACGGTCTCGACAGCAATGGCGTGTATGCACCTCCTATTCCC--TGCCCA-CTG-ACTGCCCTGGTC--CTCTCCTCC---GGCTTGGCACTGATGATCGCACAGTTACAACGGCACTTCCGAGCTCCAGCTCGAGCGCATGAACGTCTACTTCAACGAGGTATGT----CAACG-GCCACGTCGTC------------------AA-TTCAAA-TTTGA-C----------CCTCT-CG-GC-GTGGATCAACCGCCGCCGC----CAAG----CCCTTGCTAACGCG-TTTTCCCCC-AGGCTTCCGGCAACAAGTATGTGCCCCGCGCCGTCCTCGTCGATCTCGAGCCCGGTACCATGGACGCCGTC

>Diaporthe_hordei_CBS_481_92

GTCGGC-CCATGCTGTGCTCTCGCA-TCCTCCTCTGC----CCCTGAGACTCAGGCTACCTACCCCACCATCGCGACCACACCCACGG--TCGGGCCTC-AAAACACCACCAGCACCTTGCGAAGAGCACCCAG------------AGACGCGTCAGATTGCTAACATAGCCTTT-TCCTTGCCCACAGGTTCACCTCCAGACCGGCCAATGCGTAAGTTGCCTCCTGTCAAC-ACC-GTCGGACCTTACCGCCA-CCCGTAGCTGACACATTTCCCAGGGTAACCAAATCGGTGCTGCTTTCTGGTGCGTC------------------------CCAG--CTTCAG---CTCCGAGC-------------------------------CTACCACCGCG-ACGCTCGAC-------GCGCGACA-AGGCTAGCTCGTAGCCTCG-AT----ACTGACCTCGGCTCTTTAGGCAAACCATCTCTGGCGAGCACGGTCTCGACAGCAATGGCGTGTATGCACCTCCTATTCCC--TGACTT-CTG-----ACCTCGTC--CTCTCCTGC---GGCTTGGCACTGATGATCGCACAGTTACAACGGCACTTCCGAGCTCCAGCTCGAGCGCATGAATGTCTACTTCAACGAGGTAAGT----CAACA-GCCACGTCGCC------------------AATTTCAGA-TTTGA-C----------CGTCT-CG-GC-ATGG-TTGACTGCCACCGC----CAAG----GACTTGCTAACGCG-TTTTCGCCC-AGGCCTCCGGCAACAAGTATGTGCCCCGGGCCGTCCTCGTCGACCTCGAGCCCGGTACCATGGACGCCGTC

>Diaporthe_hubeiensis_JZB320123

NNNNNNNNNNNNNNNNNNNNNNNNNNNNNNNNNNNNNNNNNNNNNNNNNNNNNNNNNNNNNNNNNNNNNNNNNNNNNNNNNNNNNNNNNNNNNNNNNNNNNNNNNNNNNNNNNNNNNNNNNNNNNNNNNNNNNNNNNNNNNNNNNNNNNNNNNNNNNNNNNNNNNNNNNNNNNNNNNNNNNNNNNNNNNNNNNNNNNNNNNNNNNNNNNNNNNNNNNNNNNNNNNNNNNNNNNNNNNNNNNNNNNNNNNNNNNNNNNNNNNNNNNNNNNNNNNNNNNNNNNNNNNNNNNNNGGTGCTGCTTTCTGGTGCGTT------------------------CCCG--CT-CAG---CTCAGAGT-------------------------------CGACCACCGCG-ACAATCGAC-------GCGCGACA-ACAGTAGCTCGTGGCATTG-AT----ACTGACATCGGCTC-CTAGGCAAACCATCTCTGGCGAGCACGGCCTCGACAGCAATGGCGTGTATGCACCTCCTATTCCC--TGCCTA-CTG-----GTCTCGTCCTCTCTCCTAC---GCCTTGGCACTGATAATGGCACAGCTACAACGGCACTTCTGAGCTCCAGCTCGAGCGCATGAACGTCTACTTCAACGAGGTGAGC----CAAAG-CCCACGTTGTC------------------AA-TCCGGA-TTTGA-C----------CACCTGCA-GC-ACAA-TCCCCTGCCACCGC----CAAG----GCCTAGCTAACGCG-TTATCGTCC-AGGCCTCCGGCAACAAGTATGTGCCTCGCGCCGTCCTCGTCGATCTCGAGCCCGGTACCATGGATGCCGTC

>Diaporthe_infecunda_CBS_133812

GTCGGC-CCTTGCTGTGCTCTCGCA-TCCTCCTCTGC----CCCTGAGCCTCAGGC----TACCCCACCATCGCGACCACACCCACGG--TCGGGCTC--AAAACACCACCAGCACCCTGCGATGAGCACCCAGATA-TGTTTGGAAGACGCGTCAGATTGCTAACATGAACTTT-TTCTTGCCCACAGGTTCACCTTCAGACCGGCCAATGCGTAAGTTGCCTCCTGTCAAC-ACCG-CCCGACCTTATCGCCA-CCCATAGCTGACACGTTTCCCAGGGTAACCAAATCGGTGCTGCTTTCTGGTGCGTA------------------------CCAG--CTCCAG---CTCCGAGC-------------------------------CTGCCACCGCG-ATGCTCGAC-------GCGCGACA-AGACCACCTCCAAGCATCG-TT----ACTGACCTTGTTTCTTTAGGCAAACCATCTCTGGCGAGCACGGTCTCGACAGCAATGGCGTGTATGTACCTCCTATTCCC--TGCCCA-CCG-----ATCTCGTC--CTCTCCTCC---GGCTTGGCACTGATGATCGCACAGTTACAACGGCACTTCCGAGCTCCAGCTCGAGCGCATGAACGTCTACTTCAACGAGGTAAGC----CTATG-GCCACGTCTTC------------------AA-TCCAAG-TTTGA-C----------CGTCT-CG-GC-ATGG-TTTACTGCCGCCGC----CAGG----GCCTTGCTAATGCG-CTCTTGCCC-AGGCCTCCGGCAACAAGTATGTGCCTCGCGCCGTCCTCGTCGATCTCGAGCCCGGTACCATGGACGCCGTC

>Diaporthe_infertilis_CBS_230_52

GTCGGC-CCATGCTGTGCTCTCGCA----TCCTCTTC----CCCTGAGCATCAGGC----TACCCCACCATCGCGACCACACCCACGG--TCGGGCCTC-AAAACACAACCAGCTCCCTTCGAAGAGCACCCAGATG-CCCTTGGAACACGCGTCAGATTGCTAACATGACCTTT-TCCTCGCCCACAGGTTCACCTTCAGACCGGCCAATGCGTAAGTTACCTCCTGTCAAC-ACC-GCCAGACCTTATCGCCA-CCCGTAGCTGACACGTTTCCCAGGGTAACCAAATCGGTGCTGCTTTCTGGTGCGTC------------------------GCAGCTCTCCAG---CTCCAAGC-------------------------------CTACTGCCGCG-ACCCTCGAC-------GCGCGACA-AGGCGAGCTCGAAGCATCG-AT----ACTGACCTCGGTTCTCTAGGCAAACCATCTCTGGCGAGCACGGTCTCGACAGCAATGGCGTGTATGTACCTCCTATTCCC--TGCCGA-CCG-----CCCTCGTC--CTCTCCTCC---GGCTTGGCACTGATGATCGCACAGTTACAACGGCACTTCCGAGCTCCAGCTCGAGCGCATGAACGTCTACTTCAACGAGGTAAGT----CAATA-GCCACGTTGCC------------------AA-TTCAGA-TTTGA-G----------CCTCT-CG-GC-ATGG-TTGACTGCCGCCGC----CAAG----CCCTAGCTAACGCG-TATTCGCCC-AGGCCTCCGGCAACAAGTATGTCCCCCGCGCCGTCCTCGTCGATCTCGAGCCCGGTACCATGGACGCCGTC

>Diaporthe_kochmanii_BRIP_54033

??????????????????????????????????????????????????????????????????????????????????????????????????????????????????????????????????????????????????????????????????????????????????????????????????????????????????????????????????????????????????????????????????????????????????????????????????????????????????????????????????????????????????????????????????????????????????????????????????????????????????????????????????????????????????????????????????????????????????????????????????????????????????????????????????????????????????????????????????????????????????????????????????????????????????????????????????????????????????????????????????????????????????????????????????????????????????????????????????????????????????????????????????????????????????????????????????????????????????????????????????????????????????????????????????????????????????????????????

>Diaporthe_kongii_BRIP_54031

GTCGGC-CCATGCTGTGCTCTCGCA----TCCTCTGC----CCCTGAGCCTGAGGC----TACCCCACCATCGCGACCACACCCATGG--TCGGGCCTC-AAAACACCACCGTCGCCCTGCGAAGAGCACCCAGATG-CTACTGGAACACGCGTCAGATTGCTAACATGACCTTT-TTCTCGCCCACAGGTTCACCTTCAGACCGGCCAATGCGTAAGTTGCCTCCTGTCAAC-ACC-GCCAGACCTTATCGCCA-CCCGTAGCTGACACGTTTCCCAGGGTAACCAAATCGGTGCTGCTTTCTGGTGCGTC------------------------GCAGCTCTCCAG---CTTCAAGC-------------------------------CTACCACCGCG-ACCCTCGAC-------GCGCGACA-AGGCGAGCTCGAAGCATCG-AT----ACTGACCTCGTTTCTTTAGGCAAACCATCTCTGGCGAGCACGGTCTCGACAGCAATGGCGTGTATGCACCTCCTATTCCC--TGCCCA-CTG-ACTGCCCTGGTC--CTCTCCTCC---GGCTTGGCACTGATGATCGCACAGTTACAACGGCACTTCCGAGCTCCAGCTCGAGCGCATGAACGTCTACTTCAACGAGGTATGT----CAACG-GCCACGTCGTC------------------AA-TTCAAA-TTTGA-A----------CCTCT-CG-GC-ATGG-TCAACTGCCGCCGC----CAAG----CCCTTGCTAACGCG-TTTTCCCCC-AGGCTTCCGGCAACAAGTATGTGCCCCGCGCCGTCCTCGTCGATCTCGAGCCCGGTACCATGGACGCCGTC

>Diaporthe_leucospermi_CBS_111980

NNNNNNNNNNNNNNNNNNNNNNNNNNNNNNNNNNNNNNNNNNNNNNNNNNNNNNNNNNNNNNNNNNNNNNNNNNNNNNNNNNNNNNNNNNNNNNNNNNNNNNNNNNNNNNNNNNNNNNNNNNNNNNNNNNNNNNNNNNNNNNNNNNNNNNNNNNNNNNNNNNNNNNNNNNNNNNNNNNNNNNNNNNNNNNNNNNNNNNNNNNNNNNNNNNNNNNNNNNNNNNNNNNNNNNNNNNNNNNNNNNNNNNNNNNNNNNNNNNNNNNNNNNNNNNNNNNNNNNNNNNNNNNNNNNNNNNNNNNNNNNNTGGTGCGTA------------------------CCAG--CTCCAG---CTCCGAGT-------------------------------CTGCCACCGCG-ATGCTCAAC-------GCGCGACA-AGACCACCTCCAAGCATCG-AT----ACTGACCTTGTTTCTTTAGGCAAACCATCTCTGGCGAGCACGGTCTCGACAGCAATGGCGTGTATGTACCTCCTATTCCC--TGCCTA-CCG-----ATCTCGTC--CTCTCCTCC---GGCTTGGCACTGATGATCGCACAGTTACAACGGCACTTCCGAGCTCCAGCTCGAGCGCATGAACGTCTACTTCAACGAGGTAAGC----CTACG-GCCACGTCTTC------------------AA-TCCAAA-TTTGA-C----------AGTCT-CG-GC-ATGG-TTTACTGCCGCCGC----CAGG----GCCTTGCTAATGCG-CTCTTGCCC-AGGCCTCCGGCAACAAGTATGTGCCTCGCGCCGTCCTCGTCGATCTCGAGCCCGGTACCATGGACGCCGTC

>Diaporthe_longicolla_FAU_599

????????????????????????????????????????????????????????????????????????????????????????????????????????????????????????????????????????????????????????????????????????????????????????????????????????????????????????????????????????????????????????????????????????????????????????????????????GTGCTGCTTTCTGGTGCGTC------------------------GCAGCTCTCCAG---CTCCAAGC-------------------------------CTACCACCGCG-ACCCTCGAC-------GCGCGACA-AGGCGAGCTCGAAGCATCG-AT----ACTGACCTCGGTCCTTTAGGCAAACCATCTCTGGCGAGCACGGTCTCGACAGCAATGGCGTGTATGCACCTCCTATCCCC--TGCCCG-TGG----CCCCTCGTC--CTCTCCTCC---GGCTTGGCACTGATGATCGCACAGTTACAACGGCACTTCCGAGCTCCAGCTCGAGCGCATGAACGTCTACTTCAACGAGGTAAGT----CAATA-GCCACGTCGTC------------------AA-TTCAAA-TTTGA-CCCTCTCGGCACCTCT-CG-GC-ATGG-TCAACTGCTGCCGC----CAAG----CCTTTGCTAACGCG-TTTTCGCCC-AGGCCTCCGGCAACAAGTATGTGCCCCGCGCCGTCCTCGTCGATCTCGAGCCCGGTACCATGGACGCCGTC

>Diaporthe_longispora_CBS_194_36

GTCGGC-CCATGC--TGCTTTCGCA-TCCTCCTCTGC----CCCTGAACCTCAGGC----TACCCCACCATCGCGACCACGCCCACGG--TCAGGCCTC-AAAACACCACCAACACCCTGCTGGGAGCACCCAGATG-CTCTCCGAAGACACGTCAGATTGCTAACATGGTCTTT-TTCTCGCCCACAGGTTCACCTTCAGACCGGCCAATGCGTAAGTTGCTTCCTGTCAAC-ACCACCCGGACCTCATCGCCA-CCTGTAGCTGACACGTTTCCCAGGGTAACCAAATCGGTGCTGCTTTCTGGTGCGTC------------------CCAGCTCCAG--CTCCAG---CTCCAAGT-------------------------------CTGCCGCCGCG-ACCCTCGAC-------GCGCGACA-ACACTAGCTCGCGACATCA-TC----ACTGACCTCGCCTCT-TAGGCAAACCATCTCTGGCGAGCACGGTCTCGACAGCAATGGCGTGTATGTACCTCTTATTCCC--TGCCTATCTG-----ATCTCGTC--CCCTCCTCC---GGCTTGACACTGACAATTGCACAGTTACAACGGCACTTCCGAGCTCCAGCTCGAGCGCATGAACGTCTACTTCAACGAGGTAAGT----CAACA-GCCACGTCGTC------------------AA-TCCAAA-TTTGA-C----------CAC--ACG-GC-ATGG-TTTCCTGCCGGCGC----CAAG----GCCTTGCTAACGCG-TTATCGCCC-AGGCTTCCGGCAACAAGTATGTGCCTCGCGCCGTCCTCGTCGATCTCGAGCCCGGTACCATGGATGCCGTC

>Diaporthe_lusitanicae_CBS_123212

GTCGGCCCTCTGCTGTGCTCTCGCA-TCCTCCTCTGC----CCCTGAGCCTCAGGC----GACCCCACCATCGCGACCACACCCACGG--TCAGGCCTC-AAAACACCACCAGCGCCCTGCGAAGTGCACCCAGATG-CCCTTGGAAGACGCGTCAGATTGCTAACATGGACTTTTTTCTCTCCTACAGGTTCACCTTCAGACCGGCCAATGCGTAAGTTGCCTCCTGTCAAC-ACC-GCCCGACCTTATCGCCA-CCCGTAGCTGACACGTTTCCCAGGGTAACCAAATCGGTGCTGCTTTCTGGTGCGTC------------------------CCAG--CTCGAG---CTCCAGCT----------------------CCAAGCCTGCCACGACCGCG-ACATTCGAC-------ACGCGACA-AGACTGGCTCGCAATATCC-TTAT--ACTGACCTCGGCTGTTTAGGCAAACCATCTCTGGCGAGCACGGTCTCGACAGCAATGGCGTGTATGCACCTCCTATTCCC--TGCCCT-TTG-----ATCTCATC--CTCCCCTCC---GGCTTGGCACTGATGTTTGCACAGTTACAACGGCACTTCCGAGCTCCAGCTCGAGCGCATGAACGTCTACTTCAACGAGGTGAGT----CAAAA-GCCACGTCTTC------------------AG-TTCAAA-TTTGA-G----------CGACCTCG-GC-ATGA-TTTACTGCCGCCGC----AAAG----ACTTTGCTAACGCG-CTTTCGCCC-AGGCCTCCGGCAACAAGTATGTGCCTCGCGCCGTCCTCGTCGATCTCGAGCCCGGTACCATGGACGCCGTC

>Diaporthe_machili_SAUCC194_111

NNNNNNNNNNNNNNNNNNNNNNNNNNNNNNNNNNNNNNNNNNNNNNNNNNNNNNNNNNNNNNNNNNNNNNNNNNNNNNNNNNNNNNNNNNNNNNNNNNNNNNNNNNNNNNNNNNNNNNNNNNNNNNNNNNNNNNNNNNNNNNNNNNNNNNNNNNNNNNNNNNNNNNNNNNNNNNNNNNNNNNNNNNNNNNNNNNNNNNNNNNNNNNNNNNNNNNNNNNNNNNNNNNNNNNNNNNNNNNNNNNNNNNNNNNNNNNNNNNNNNNNNNNNNNNNNNNNNNNNNNNNNNNNNNNNNNNNNNNNNNNNNNNNNNNNNNNNNNNNNNNNNNNNNNNNNNNNNNNNNNNNNCTCA---GCTCGAGC-------------------------------CTGCCACCGCG-ACGCTCGAC-------GCGCGACA-AGACTAGCTCGCAACATCT-TT----ACTGACCTCGTCTCTTTAGGCAAACCATCTCTGGCGAGCACGGTCTCGACAGCAATGGCGTGTATGTACCTCCTATTCCC--TGCCCA-CCG-----ATCTCGTC--CTCTCCTCC---GGCTTGGCACTGATGATCGCACAGTTACAACGGCACTTCCGAGCTCCAGCTCGAGCGCATGAACGTCTACTTCAACGAGGTAAGC----CTATG-GCCACGGCTCC------------------AA-TCCAAA-TTTGA-C----------CGTCT-CG-GC-ATGG-TTTACTGCCGCCGC----CAGG----GCCTTGCTAATGCG-CTCTCGCCC-AGGCCTCCGGCAACAAGTATGTGCCTCGCGCCGTCCTCGTCGATCTCGAGCCCGGTACCATGGACGCCGTC

>Diaporthe_manihotia_CBS_505_76

GTCGGC-CCATGC--TGCTCTCGCA-CCCTCCTCTGC----CCCTGAACCTCAGCC----TACCCCACCATCGCGACCACGCTCCCACAGCAGGGCCTC-AAAACACCACCAGCACCTTGCGATGAGCACCCGCGTG-CCCTTGGAACACGCGTCAGATTGCTAACATGGACTTT-TTCTCGCCTGCAGGTTCACCTCCAGACCGGCCAATGCGTAAGTTGCTCCCTGTCAAC-ACCG-CCCGACCTTATCGCCA-CCCGTAGCTGACACGTTTCCCAGGGTAACCAAATCGGTGCTGCTTTCTGGTGCGTC------------CCAGCTCCAGCTCCAG--CTCCAG---CTCGAGGC-------------------------------CTACCGCCGCG-ATGCTCGAC-------GCGCGACA-AGACCAGCTCGCAACATTG-TT----ACTGACCTCGGCTCCGTAGGCAAACCATCTCTGGCGAGCACGGCCTCGACAGCAATGGCGTGTATGCACCTCCTATTCCC--TGCCCA-CCG-----ATCTCGTC--CTGTCCTCC---GGCTTGGCACTGATGATCGCACAGTTACAACGGCACTTCTGAGCTCCAGCTCGAGCGCATGAACGTCTACTTCAACGAGGTATGT----CAATG-GCCACGTCGTC------------------AA-TCCAAA-TTTGC-C----------CATCTGCT-GC-ATTG-TGTGCTGCCGTAGC----CAAA----GCCTTGCTAACGCG-TTATCGCCC-AGGCTTCCGGCAACAAGTATGTGCCCCGCGCTGTCCTCGTCGATCTCGAGCCCGGTACCATGGACGCCGTC

>Diaporthe_mayteni_CBS_133185

GTCGGC-CCTTGC--TGCTTTCGCA-TCTTCCTCTGC----CCCTGAGCCTCAGGC----TACCCCACCATCGCGACCACACCCACGG--TCAGGCCTC-AAAACATCACCAACACCCTGAGGAGAGCACCATGATG-CCCTCGGAACACGCGTCAGATTGCTAACATGGACTTT-TTCTCGCCCACAGGTTCACCTTCAGACCGGCCAATGCGTAAGTTGCTTTCTGTCAAC-ACCT-CCAGACCTTATCGCCA-CCTGTAGCTGACACGTTTCGCAGGGTAACCAAATCGGTGCTGCTTTCTGGTGCGTC------------------------CCAG--CTCCAG---CTCCAAGT-------------------------------CTACCACCGCG-ACGCTCGAC-------ACGCGACA-AGACTAGCTCACAGCATCT-TT----ACTGACCTCTGCCCT-TAGGCAAACCATCTCTGGCGAGCACGGCCTCGACAGCAATGGCGTGTATGCACCTCCTATTCCC--TGAACA-ATG-----TTCTCGTC--CTCTCCTCC---GGCTTGGCACTGATGATCGCACAGTTACAACGGCACTTCCGAGCTCCAGCTCGAGCGCATGAACGTCTACTTCAACGAGGTAAGT----CAAAA-GCCACGTCGTG------------------AA-TTCAAT-TTTGACC----------CTCTTACG-GC-ATGA-TTTCCTGCCGCCGC----CAAG----GCCTTGCTAACGCG-TTATCGCCC-AGGCTTCCGGCAACAAGTATGTGCCTCGCGCCGTCCTCGTCGATCTCGAGCCCGGTACCATGGACGCCGTC

>Diaporthe_megalospora_CBS_143_27

GTCGGC-CCATGCT--GCTCTCGCA-TCCTCCTCTGC----CCCTGAGCCTCAGGC----TACCCCACCATCGCGATCACACCCACGG--TCGGGCCTC-AAAACACCACCAGCACCCTGCAAAGAACACCCAGATG-CCGTTGTAAGACGCGTCAGATTGCTAACATGACCTTT-TTCTTGCCCACAGGTTCACCTTCAGACCGGCCAATGCGTAAGTTGCTCCCTGTCAAC-ACC-CCCAGACCTCATCGCCA-CCTGTAGCTGACACGTTTCCCAGGGTAACCAAATCGGTGCTGCTTTCTGGTGCGTC------------------------CCAG--CTCCAG---CTTCGAGC-------------------------------CTACCACCGCG-ACGCTCGACGCTCGTTGCGCGACA-GGGCTAGCTCGTAGTATCG-AC----ACTGACCTCTGTTCTTTAGGCAAACCATCTCTGGCGAGCACGGTCTCGACAGCAATGGCGTGTATGTACCTCCTATTCCC--CGACTA-CCG-----ACCTCGTCCTCTCTCCTCC---GGCTTGGCACTGATGATCGCACAGTTACAACGGCACTTCCGAGCTCCAGCTCGAGCGCATGAACGTCTACTTCAACGAGGTATGT----CAATA-GCCACGTCGCC------------------AA-TTCAAA-TTTGA-C----------CGTCT-CG-GC-ATGG-TTAACTGCCGCCGC----CAAG----CCCTTGCTAATTCG-TTTTCGCCC-AGGCCTCCGGCAACAAGTATGTGCCCCGCGCCGTCCTCGTCGATCTCGAGCCCGGTACCATGGACGCCGTC

>Diaporthe_melonis_CBS_507_78

GTCGGC-CCATGCT--GCTCTCGCA-TCCTCCTCTGC----CCCTGAACCTCAGCC----TACCCCACCATCGCGACCACACCCACGG--TCGGGCCTC-AAAACACCACCAGCGCCCTGCAAAGAGCTCCAAGATG-CCCTTGGAATACGCGTCAGATTGCTAACATGACCTTT-TTCTCGCCCACAGGTTCACCTTCAGACCGGCCAATGCGTAAGTTGCTCCTTGTCAAC-ACC-GCCAGACCTTATCGCCA-CCCGTAGCTGACACGTTTCCCAGGGTAACCAAATCGGTGCTGCTTTCTGGTGCGTC------------------------GCAGCTCTCCAG---CTCCGAGC-------------------------------CTACCACCGCG-ACACTCGAC-------GCGCGACA-AGGCGAGCTCGAAACAGCC-AT----ACTGACCTCGATTCTTTAGGCAAACCATCTCTGGCGAGCACGGTCTCGACAGCAATGGCGTGTATGGACCTCCTATTCCC--TGACTA-CCG------------C--CTCTCCTCT---GGCTTGGCACTGATGATCGCACAGTTACAACGGCACTTCCGAGCTCCAGCTCGAGCGCATGAACGTCTACTTCAACGAGGTAAGT----CAATA-GCCACGTCGCC------------------AA-TTCAGA-CTTGA-C----------CGTCT-CG-GC-ATGG-TGAACTGCCGCCGC----CAAG----CCCTTGCTAACGCG-TTTTCCTTT-AGGCCTCCGGCAACAAGTATGTGCCCCGCGCCGTCCTCGTCGATCTCGAGCCCGGTACCATGGACGCCGTC

>Diaporthe_middletonii_BRIP_54884e

GTCGGC-CCTTGCTGTGCCCTCGCA-TCCTCCTCTGC----CCCTGAACCTCAGGC----TACCCCACCATCGCGACCACACCCACGG--TCGGGCTC--AAAACACCACCAGCACCCTGCGATGAGCACCAAGATT-CGTTTGGAAGACGCGTCACATTGCTAACATGAACTTT-CTCCT--CCACAGGTTCACCTTCAGACCGGCCAATGCGTAAGTTGCCTCCTGTCAAC-ATCG-CCCGACCTTATCGCCACCCCGTAGCTGACACGTTTCCCAGGGTAACCAAATCGGTGCTGCTTTCTGGTGCGTA------------------------CCAG--CTCCAG---CTCCGAGC-------------------------------CTGCCACCGCG-ACACTCGAC-------GCGCGACA-ATACTAGCTCGCAATATCG-TT----GCTGACCTCGTCTCTTCAGGCAAACCATCTCTGGCGAGCACGGTCTCGACAGCAATGGCGTGTATGTACCTCCTATTCCC--TGCCGA-CCG-----ATCTCATC--CTCTCCTCC---GGCTTGGCACTGATGATCGCACAGTTACAACGGCACTTCCGAGCTCCAGCTCGAGCGCATGAACGTCTACTTCAACGAGGTAAGC----CTATG-GCCACGTCTTC------------------GA-TCCAAG-TTTGA-C----------CGTCT-CG-GC-ATGG-TTTACTGCCGCCGC----CAGG----ACCTTGCTAATGCG-CTCTCGCCC-AGGCCTCCGGCAACAAGTATGTGCCTCGCGCCGTCCTCGTCGATCTCGAGCCCGGTACCATGGACGCCGTC

>Diaporthe_miriciae_BRIP_54736j

GTCGGC-CCAAGCTGTGCTCTCGCA----TCCTCTGC----CCCTGAGCCTGAGGC----TACCCCACCATCGCGACCACACTCACGG--TCGGGCCTC-AAAACACCACCAGCTCCCTGCGAAGAGCACCCAGATG-CCCTTGGAACACGCGTCAGATTGCTAACATAGCCTTT-TTCTCGCCTACAGGTTCACCTTCAGACCGGCCAATGCGTAAGTTGCTCCCTGTCAAC-ACC-GCCGGACCTTATCGCCA-CCCGTAGCTGACACGTTTACCAGGGTAACCAAATCGGTGCTGCTTTCTGGTGCGTC------------------------GCAGCTCTCCAG---CTCCAAGC-------------------------------CTACCGCCGCGAACCCTCGAC-------GCGCGACA-AGGCGAGCTCGAAGCATCG-AT----ACTGACCTCGGTTCTTTAGGCAAACCATCTCCGGCGAGCACGGTCTCGACAGCAATGGCGTGTATGCACCTCCTATTCCC--TGCCCG-TGG-----CCCTCATC--CTCTTCTCC---GGCTTGGCACTGATGATCGCACAGTTACAACGGCACTTCCGAGCTCCAGCTCGAGCGCATGAACGTCTACTTCAACGAGGTCAGT----CAATA-GCCACGTCGTC------------------AA-TTCAAA-TTTGA-A----------CCTCT-CG-GC-ATGG-TCAACTGCCGCCGC----CAAG----CCCCTGCTAACGCG-TTTTCGCCC-AGGCCTCCGGCAACAAGTATGTGCCCCGCGCCGTCCTCGTCGATCTCGAGCCCGGTACCATGGACGCCGTC

>Diaporthe_myracrodruonis_URM7972

NNNNNNNNNNNNNNNNNNNNNNNNNNNNNNNNNNNNNNNNNNNNNNNNNNNNNNNNNNNNNNNNNNNNNNNNNNNNNNNNNNNNNNNNNNNNNNNNNNNNNNNNNNNNNNNNNNNNNNNNNNNNNNNNNNNNNNNNNNNNNNNNNNNNNNNNNNNNNNNNNNNNNNNNNNNNNNNNNNNNNNNNNNNNNNNNNNNNNNNNNNNNNNNNNNNNNNNNNNNNNNNNNNNNNNNNNNNNNNNNNNNNNNNNNNNNNNNNNNNNNNNNNNNNNNNNNNNNNNNNNNNNNNNNNNNNNTGCTGCTTTCTGGTGCGTA------------------------ACTG--CTCCAG---CTCCGAGC-------------------------------CTGCCACCGCG-GCGCTCGAC-------GCGCGACA-ATACTAGCTCGCAACATCG-TT----ACTGACCTCGTCTCGTTAGGCAAACCATCTCTGGCGAGCACGGTCTCGACAGCAATGGCGTGTATGTACCTCCTGTTCCC--TGCCCA-CCG-----ATCTCGTC--CTCTCCTCC---GGCTTGGCACTGATGATCGCACAGTTACAACGGCACTTCCGAGCTCCAGCTCGAGCGCATGAACGTCTACTTCAACGAGGTAAGC----CTACG-GCCACGTCTTC------------------AA-TTCAAA-TTTGA-C----------CGTCT-CG-GC-ATGG-TTTACTGCCGCCGC----CAGG----GCCTTGCTAACGCG-CTTTCGCCC-AGGCCTCCGGCAACAAGTATGTGCCTCGCGCCGTCCTCGTCGATCTCGAGCCCGGTACCATGGACGCCGTC

>Diaporthe_neoarctii_CBS_109490

GTCGGCCCTTTGCTGTGCTCTCGCATTCCTCCTCTGC---CCCCTGAGCCTCAGGCTAC-TACCCCACCATCGCGACCACACCCACGG--TCAGGCCTC-AAAACACCACCAGCGCCCTGCAAAGTGCACCCAGATG-CCCTTGGAAGACGCGTCAGATTGCTAACATGGACCTTGTTCTCTCCTACAGGTTCACCTCCAGACCGGCCAATGCGTAAGTTGCCTCCTGTCAAC-ACCGCCGGGACCTTATCGCCA-CCCGTAGCTGACACGTTTCCCAGGGTAACCAAATCGGTGCTGCTTTCTGGTGCGTC------------------------CCAG--CTCCAG---CTCAAGCTTCAGCTCCAGCTCCAGCT----CTAAGCCTGCTACGACCGCG-ACATTCGAC-------ACGCGACA-GGAATAGCTCGCAATATCT-TTAT--ACTGACCTCGGCTGTTTAGGCAAACCATCTCTGGCGAGCACGGTCTCGACAGCAATGGCGTGTATGCACCTCCTATTCCCCTTGCCCA-CTG-----ATCTCGTCCTCTCCCCCCCTCGGGCCTAGCACTGATGTTTGCACAGTTACAACGGCACTTCTGAGCTCCAGCTCGAGCGCATGAACGTCTACTTCAACGAGGTGAGT----CAAAA-GCCACGTCTTC------------------AA-TTCAAG-TTTGA-G----------TGTTCTCG-GC-ATGA-TTTCCTGCTACCGCCGCAAAAAG---ACCTTACTGACACATCTTTCGCCC-AGGCCTCCGGCAACAAGTATGTGCCTCGCGCCGTCCTCGTCGATCTCGAGCCCGGTACCATGGACGCCGTC

>Diaporthe_neoraonikayaporum_MFLUCC_14_1136

NNNNNNNNNNNNNNNNNNNNNNNNNNNNNNNNNNNNNNNNNNNNNNNNNNNNNNNNNNNNNNNNNNNNNNNNNNNNNNNNNNNNNNNNNNNNNNNNNNNNNNNNNNNNNNNNNNNNNNNNNNNNNNNNNNNNNNNNNNNNNNNNNNNNNNNNNNNNNNNNNNNNNNNNNNNNNNNNNNNNNNNNNNNNNNNNNNNNNNNNNNNNNNNNNNNNNNNNNNNNNNNNNNNNNNNNNNNNNNNNNNNNNNNNNNNNNNNNNNNNNNNNNNNNNNNNNNNNNNNNNNNNNNNNNNNNNNNNNNNNNNNNNNNNNNNNNNNNNNNNNNNNNNNNNNNNNNNNNNNNNNNNNNNNNNNNNNNNNNT-------------------------------CTACCACCGCG-ACGCTTGAC-------TCGCGACA-AGACTAGTTAGCAGCTTCG-TT----ACTGACCTCGGCTC-TTAGGCAAACCATCTCTGGCGAGCACGGCCTCGACAGCAATGGCGTGTATGCACCTCCTATTCCC--TGCCAA-ATG-----ATATCGTC--CTCTCTTCC---GGCTTGGCACTGACAATTGCATAGTTACAACGGCACTTCCGAGCTGCAGCTCGAGCGCATGAATGTCTACTTCAACGAGGTCAGT----AAACA-GCTACATCGTG------------------AA-TCCAGA-TTTGG-C----------CATCTACC-AC-ATTG-TATCCTGCCGCCGA----CAAG----GCTTCGCTAACGTG-TTATCGCCC-AGGCCTCCGGCAACAAGTATGTGCCTCGCGCCGTCCTCGTCGATCTCGAGCCCGGTACCATGGACGCCGTC

>Diaporthe_novem_CBS_127271

GTCGGCCCTCTGCTGGGCTCTCGCATTCCTCCTCTGC----CCCTGAGCCTCAGGC----TACCCCACCATCGCGACCACACCCACGT--TCAGGCCTC-AAAACACCACCAGCACCCTGCGAAGTGCACCCAGATG-CCCTTGAAAGACGCGTCAGACTGCTAACATGGACTTT-TTCTCTCCTACAGGTTCACCTTCAGACCGGCCAATGCGTAAGTTGCCTCCTGTCAAC-ACCGCCGGGACCTTATCGCCA-CCCGTAGCTGACACGTTTCCCAGGGTAACCAAATCGGTGCTGCTTTCTGGTGCGTC------------------------CCAG--CTCCAG---CTCCAGCTCCAGCTCCAGCT----------TCAAGCCTGCCACGACCGCG-ACATTCGAC-------ACGCGACA-AGACGAGCTCGAAACATGA-TTAT--ACTGACCTCGGCTGTTTAGGCAAACCATCTCTGGCGAGCACGGTCTCGACAGCAATGGCGTGTATGCACCTCCTATTCCCC-TGCCCA-CTG-----ATCTCGTC--CTCCTCTCC---GGCTTGGCACTGATGTTTGCACAGTTACAACGGCACTTCTGAGCTCCAGCTCGAGCGCATGAACGTCTACTTCAACGAGGTAAGC----CAAACAGCCACGTCTTC------------------AA-TTCACA-TTTGA-G----------CGTTCTCG-GC-ATGG-ATTATTGCCGCCGC----AGAG----ACCTGACTGACGCG-CTTTCGCCT-AGGCCTCCGGCAACAAGTATGTGCCTCGCGCCGTCCTCGTCGATCTCGAGCCCGGTACCATGGACGCCGTC

>Diaporthe_ovalispora_ICMP20659

????????????????????????????????????????????????????????????????????????????????????????????????????????????????????????????????????????????????????????????????????????????????????????????????????????????????????????????????????????????????????????????????????????????????????????????????????????????????????????????????????????????????????????????????????????????????????????????????????????????????????????????????????????????????AGCTCGAAGCATCG-AT----GCTGACTTCGGTTCTTTAGGCAAACCATCTCTGGCGAGCACGGTCTCGACAGCAATGGCGTGTATGCACCTCCTATTCCC--TGCCCA-CTG-ACTGCCCTGGTC--CTCTCCTCC---AGCTTGGCACTGATGATCGCACAGTTACAACGGCACTTCGGAGCTCCAGCTCGAGCGCATGAACGTCTACTTCAACGAGGTATGT----CAACG-GCCACGTCGTC------------------AA-TTCAAA-TTTGA-C----------TCTGT-CG-GC-ATGG-TCAGCTGCCGCCGC----CAAG----CCCTTGCTAACGCG-TTTTCCCCC-AGGCTTCCGGCAACAAGTATGTGCCCCGCGCCGTCCTCGTCGATCTCGAGCCCGGTACCATGGACGCCGTC

>Diaporthe_pachirae_CDA_728

NNNNNNNNNNNNNNNNNNNNNNNNNNNNNNNNNNNNNNNNNNNNNNNNNNNNNNNNNNNNNNNNNNNNNNNNNNNNNNNNNNNNNNNNNNNNNNNNNNNNNNNNNNNNNNNNNNNNNNNNNNNNNNNNNNNNNNNNNNNNNNNNNNNNNNNNNNNNNNNNNNNNNNNNNNNNNNNNNNNNNNNNNNNNNNNNNNNNNNNNNNNNNNNNNNNNNNNNNNNNNNNNNNNNNNNNNNNNNNNNNNNNNNNNNNNNNNNNNNNNNNNNNNNNNNNNNNNNNNNNNNNNNNNNNNNNNNNNNNNNNNNNNNNNNNNNNNNNNNNNNNNNNNNNNNNNNNNNNNNNNNNNNNNNNNNNNNNNNNNNNNNNNNNNNNNNNNNNNNNNNNNNNNNNNNNNNNNNCCGCG-ATGCTCGAC-------GCGCGACA-AGACCACCTCCAAGCATCG-TT----ACTGACCTTGTTTCTTTAGGCAAACCATCTCTGGCGAGCACGGTCTCGACAGCAATGGCGTGTATGTACCTCCTATTCCC--TGCCCA-CCG-----ATCTCGTC--CTCTCCTCC---GGCTTGGCACTGATGATCGCACAGTTACAACGGCACTTCCGAGCTCCAGCTCGAGCGCATGAACGTCTACTTCAACGAGGTAAGC----CTATG-GCCACGTCTTC------------------AA-TCCAAG-TTTGA-C----------CGTCT-CG-GC-ATGG-TTTACTGCCGCCGC----CAGG----GCCTTGCTAATGCG-CTCTCGCCC-AGGCCTCCGGCAACAAGTATGTGCCTCGCGCCGTCCTCGTCGATCTCGAGCCCGGTACCATGGACGCCGTC

>Diaporthe_passifloricola_CBS_141329

????????????????????????????????????????????????????????????????????????????????????????????????????????????????????????????????????????????????????????????????????????????????????????????????????????????????AATGCGTAAGTTGCTCCCTGTCAAC-ACC-GCCGGACCTTATCGCCA-CCCGTAGCTGACACGTTTACTAGGGTAACCAAATCGGTGCTGCTTTCTGGTGCGTC------------------------GCAGCTCTCCAG---CTCCAAGC-------------------------------CTACCACCGCGAACCCTCGAC-------GCGCGACC-AGGCGAGCTCGAAGCATCG-AT----ACTGACCTCGGTTCTTTAGGCAAACCATCTCTGGCGAGCACGGTCTCGACAGCAATGGCGTGTATGCACCTCCTATTCCC--TGCCCG-TGG-----CCCTCGTC--CTCTTCTCC---GGCTTGGCACTGATGATCGCACAGTTACAACGGCACTTCCGAGCTCCAGCTCGAGCGCATGAACGTCTACTTCAACGAGGTCAGT----CAATA-GCCACGTCGCC------------------AA-TTCAAA-TTTGA-A----------CCTCT-CG-GC-ATGG-TCAACTGCCGCCGC----CAAG----CCCCTGCTAACGCG-TTTTCGCCC-AGGCCTCCGGCAACAAGTATGTGCCCCGCGCCGTCCTCGTCGATCTCGAGCCCGGTACCATGGACGCCGTC

>Diaporthe_pseudolongicolla_CBS_117165

??????????????????????????????????????????????????????????????????????????????????????????????????????????????????????????????????????????????????????????????????????????????????????????????????????????????????????????????????????????????????????????????????????????????????????????????????????????????????????????????????????????????????????????????????????????????????????????????????????????????????????????????????????????????????????????????????????????????????????????????????????????????????????????????????????????????????????????????????????????????????????????????????????????????????????????????????????????????????????????????????????????????????????????????????????????????????????????????????????????????????????????????????????????????????????????????????????????????????????????????????????????????????????????????????????????????????????????????

>Diaporthe_pyracanthae_CBS142384

NNNNNNNNNNNNNNNNNNNNNNNNNNNNNNNNNNNNNNNNNNNNNNNNNNNNNNNNNNNNNNNNNNNNNNNNNNNNNNNNNNNNNNNNNNNNNNNNNNNNNNNNNNNNNNNNNNNNNNNNNNNNNNNNNNNNNNNNNNNNNNNNNNNNNNNNNNNNNNNNNNNNNNNNNNNNNNNNNNNNNNNNNNNNNNNNNNNNNNNNNNNNNNNNNNNNNNNNNNNNNNNNNNNNNNNNNNNNNNNNNNNNNNNNNNNNNNNNNNNNNNNNNNNNNNNNNNNNNNNNNNNNNNNNNNNNNNNNNNNNNNNTGGTGCGTA------------------------CCAG--CTCCAG---CTCCGAGC-------------------------------CTGCCACCGCG-ATGCTCGAC-------GCGCGACA-AGACCACCTCCAAGCATCG-AT----ACTGACCTTGTTCCTTTAGGCAAACCATCTCTGGCGAGCACGGTCTCGACAGCAATGGCGTGTATGTACCTCCTATTCCC--TGCCCA-CCG-----ATCTCGTC--CTCTCCTCC---GGCTTGGCACTGATGATCGCACAGTTACAACGGCACTTCCGAGCTCCAGCTCGAGCGCATGAACGTCTACTTCAACGAGGTAAGC----CTACG-GCCACGTCTTC------------------AA-TCCAAA-TTTGA-C----------CGTCT-CG-GC-ATGG-TTTACTGCCGCCGC----CAGG----GCCTTGCTAACGCG-CTCTCGCCC-AGGCCTCCGGCAACAAGTATGTGCCTCGCGCCGTCCTCGTCGATCTCGAGCCCGGTACCATGGACGCCGTC

>Diaporthe_racemosae_CBS_143770

??????????????????????????????????????????????????????????????????????????????????????????????????????????????????????????????????????????????????????????????????????????????????????????????????????????????????????????????????????????????????????????????????????????????????????????????????????????????????????TA------------------------CCAG--CTCCAG---CTCCGAGC-------------------------------CTACCACCGCG-ATGATCGAC-------GCGCGACA-AGGCGAGCTCGAAGCATCG-AT----ACTGACCTCGGTCCTTTAGGCAAACCATCTCTGGCGAGCACGGTCTCGACAGCAATGGCGTGTATGTACCTCCTATTCCC--TGACTA-CTG-----ACCTCGTC--CTCTCCTCC---GGCTTGGCACTGACGATCGCACAGTTACAACGGCACTTCCGAGCTCCAGCTCGAGCGCATGAACGTCTACTTCAACGAGGTAAGT----CAACA-GCCACGTCGTC------------------AA-TTCGAA-TTTGA-C----------CGTCT-CG-GC-ATGG-TTAAATGCCGCCGC----CAAG----CCCTTGCTAACGCG-TTCTCGCCC-AGGCATCCGGCAACAAGTATGTGCCCCGCGCCGTCCTCGTCGATCTCGAGCCCGGTACCATGGACGCCGTC

>Diaporthe_raonikayaporum_CBS_133182

GTCGGC-CCATGC--TGCTTTCGCA-TCCTCCTCTGC----CCCTGAGCCTCAGAC----TACCCCACCATCGCGACCACACCCACAG--TCAGGCCTC-AAAACACCACCAAC---------AGAGCACCCAGATG-CCCTGAGAAGACGCGTCAGATCGCTAACATGGACTTT-TTCTCGCCCACAGGTTCACCTTCAGACCGGCCAATGCGTAAGTTGCTTCCCGTCAAC-ACCA-CTGGACCTTATCGCCA-CCTGTAGCTGACACGTTTCCCAGGGTAACCAAATCGGTGCTGCTTTCTGGTGTGTA------------------------CCAG--CTCCAG--CCCTCAAGC-------------------------------CTACCACCGCG-ACGCTTGAC-------TCGCGACA-AGACTAGTTAGCAGCTTCG-TT----ACTGACCTCGGCTC-TTAGGCAAACCATCTCTGGCGAGCACGGCCTCGACAGCAATGGCGTGTATGCACCTCCTATTCCC--TGCCAA-ATG-----ATATCGTC--CTCTCTTCC---GGCTTGGCACTGACAATTGCACAGTTACAACGGCACTTCCGAGCTGCAGCTCGAGCGCATGAATGTCTACTTCAACGAGGTCAGT----AAACA-GCTACATCGTG------------------AA-TCCAGA-TTTGG-C----------CATCTACC-AC-ATGG-TATCCTGCCGCCGA----CAAG----GCTTCGCTAACGTG-TTATCGCCC-AGGCCTCCGGCAACAAGTATGTGCCTCGCGCCGTCCTCGTCGATCTCGAGCCCGGTACCATGGACGCCGTC

>Diaporthe_rosae_MFLUCC_17_2658

???????????????????????????????????????????????????????????????????????????????????????????????????????????????????????????????????????????????????????????????????????????????????????????????????????????????????????????????????????????????????????????????????????????????????????????????????????????????????????????????????????????????????????????????????????????????????????????????????????????ACCGCGAACCCTCGAC-------GCGCGACC-AGGCGAGCTCGAAGCATCG-AT----ACTGACCTCGGTTCTTTAGGCAAACCATCTCTGGCGAGCACGGTCTCGACAGCAATGGCGTGTATGCACCTCCTATTCCC--TGCCCG-TGG-----CCCTCGTC--CTCTTCTTC---GGCTTGGCACTGATGATCGCACAGTTACAACGGCACTTCCGAGCTCCAGCTCGAGCGCATGAACGTCTACTTCAACGAGGTCAGT----CAATA-GCCACGTCGTC------------------AA-TTCAAA-TTTGA-A----------CCTCT-CG-GC-ATGG-TCAACTGCCGCCGC----CAAG----CCCCTGCTAACGCG-TTTTCGCCC-AGGCCTCCGGCAACAAGTATGTGCCCCGCGCCGTCCTCGTCGATCTCGAGCCCGGTACCATGGACGCCGTC

>Diaporthe_rosiphthora_COAD_2913

??????????????????????????????????????????????????????????????????????????????????????????????????????????????????????????????????????????????????????????????????????????????????????????????????????????????????????????????????????????????????????????????????????????????????????????????????????????????????????????????????????????????????????????????????????????????????????????????????????????????????????????????????????????????????????????????????????????????????????????????????????????????????????????????????????????????????????????????????????????????????????????????????????????????????????????????????????????????????????????????????????????????????????????????????????????????????????????????????????????????????????????????????????????????????????????????????????????????????????????????????????????????????????????????????????????????????????????????

>Diaporthe_rossmaniae_CAA762

GTCGGC-CCTTGCTGTGCTCTCGCA-CCCTCCTCTGC----CCCTGAGCCTCAGGC----TACCCCACCATCGCGACCACACCCACGG--TCGGGCTC--AAAACACCACCAGCACCCTGCGATGAGCACCCAGATA-CGTTCGGAAGACGCGTCAGATTGCTAACATGAACTTT-TTCTTGCCCACAGGTTCACCTTCAGACCGGCCAATGCGTAAGTTGCCTCCTGTCAAC-ACCG-CCCGACCTTATCGCCA-CCCATAGCTGACACGTTTCCCAGGGTAACCAAATCGGTGCTGCTTTCTGGTGCGTA------------------------CCAG--CTCCAG---CTCCGAGC-------------------------------CTGCCACCGCG-ATGCTCAAC-------GCGCGACA-AGACCACCTCCAAGCATCG-AT----ACTGACCTTGTTTCTTTAGGCAAACCATCTCTGGCGAGCACGGTCTCGACAGCAATGGCGTGTATGTACCTCCTATTCCC--TGCCTA-CCG-----ATCTCGTC--CTCTCCTCC---GGCTTGGCACTGATGATCGCACAGTTACAACGGCACTTCCGAGCTCCAGCTCGAGCGCATGAACGTCTACTTCAACGAGGTAAGC----CTACG-GCCACGTCTTC------------------AA-TCCAAA-TTTGA-C----------AGTCT-CG-GC-ATGG-TTTACTGCCGCCGC----CAGG----GCCTTGCTAATGCG-CTCTTGCCC-AGGCCTCCGGCAACAAGTATGTGCCTCGCGCCGTCCTCGTCGATCTCGAGCCCGGTACCATGGACGCCGTC

>Diaporthe_sackstonii_BRIP_54669b

GTCGGC-CCTTGCTGCGCTCTCGCA----TCCTCTGC----CCCTGAGCCTCAGGC----TACCCCACCATCGCGAC--CACCTACGG--TCGGGCTC-AAAAACACCACCAGCACCCTGCGATGAGCACACAGATG-CGTTTGGAAGACGCGTCAGATTGCTAACATGAACTTT-TTCTTGCCCACAGGTTCACCTTCAGACCGGCCAATGCGTAAGTTGCCTCCTGTCAAC-ACCG-CCCGACCTTATCGCCA-CCCATAACTGACACGTTTCCCAGGGTAACCAAATCGGTGCTGCTTTCTGGTGCGTA------------------------CCAG--CTCCAG---CTCCGAGC-------------------------------CTGCCACCGCG-ATGCTCGAC-------GCGCGACA-AGATCACCTCCAAGCATCG-TT----ACTGACCGTGTTTCTTTAGGCAAACCATCTCTGGCGAGCACGGTCTCGACAGCAATGGCGTGTATGTACCTCCTATTCCC--TGCCCA-CCG-----ATCTCATC--CTCTCCTCC---GGCTTGGCACTGATGATCGCACAGTTACAACGGCACTTCCGAGCTCCAGCTCGAGCGCATGAACGTCTACTTCAACGAGGTAAGC----CTACG-GACACGTCTTC------------------AA-TCCAAA-TTTGA-C----------CGTCT-CG-GC-ATGG-TTTACTGCCGCCGC----CAGG----GCCTTGCTAATGCG-CTCTCGCCC-AGGCCTCCGGCAACAAGTATGTGCCTCGCGCCGTCCTCGTCGATCTCGAGCCCGGTACCATGGACGCCGTC

>Diaporthe_schini_CBS_133181

GTCGGC-CCATGCT--GCTCTCGCA-CCTTCCTCTGC----CCCTGAGCCTCAGGC----TACCCCACCATCGCGACCACACCCACGG--TCGGGCCTC-AAAACACCACCAGCACCTTGCGAAGACCACCCAGATG-CCCTTGGAACACGCGTCAGATTGCTAACATGAACTTT-TTTTCGCCTACAGGTTCACCTTCAGACCGGCCAATGCGTAAGTTGCCTCCTGTCAAC-ACC-GCCCGACCTTATCGCCA-CCCGTAGCTGACACGTTTCCCAGGGTAACCAAATCGGTGCTGCTTTCTGGTGCGTA------------------------CCAG--CTCCAG---CTCCGAGC-------------------------------CTACCACCGCG-ATGATCGAC-------GCGCGACA-AGGCGAGCTCGAAGCATCG-AT----ACTGACCTCGGTTCTTTAGGCAAACCATCTCTGGCGAGCACGGTCTCGACAGCAATGGCGTGTATGTACCTCCTATTCCC--TGACTA-CTG-----ACCTCGTC--CTCTCCTCC---GGCTTGGCACTGACGATCGCACAGTTACAACGGCACTTCCGAGCTCCAGCTCGAGCGCATGAACGTCTACTTCAACGAGGTAAGT----CAATA-GCCACGTCGTC------------------AA-TTCGAA-TTTGA-C----------CCTCT-CG-GC-ATGG-TTGACTGCCGCCGC----CAAA----CCCTTGCTAACGCG-TTCTCGCCC-AGGCCTCCGGCAACAAGTATGTGCCCCGCGCCGTCCTCGTCGATCTCGAGCCCGGTACCATGGACGCCGTC

>Diaporthe_schoeni_MFLU_15_1279

NNNNNNNNNNNNNNNNNNNNNNNNNNNNNNNNNNNNNNNNNNNNNNNNNNNNNNNNNNNNNNNNNNNNNNNNNNNNNNNNNNNNNNNNNNNNNNNNNNNNNNNNNNNNNNNNNNNNNNNNNNNNNNNNNNNNNNNNNNNNNNNNNNNNNNNNNNNNNNNNNNNNNNNNNNNNNNNNNNNNNNNNNNNNNNNNNNNNNNNNNNNNNNNNNNNNNNNNNNNNNNNNNNNNNNNNNNNNNNNNNNNNNNNNNNNNNNNNNNNNNNNNNNNNNNNNNNNNNNNNNNNNNNNNNNNNNNNNNNNNNNNNNNNNNNNNNNNNNNNNNNNNNNNNNNNNNNNNNNNNNNNNNNNNNNNNNNNNNNNNNNNNNNNNNNNNNNNNNNNNNNNNNNNNNNNNNNNATCGCG---GCTCGAC-------GCGCGATG-ACAGGACCTCGCAACATCG-TT----ATTGACTTCGACT-TTTAGGCAAACCATCTCTGGCGAGCACGGCCTCGACAGCAATGGCGTGTATGTACCTCCCATTCCC--TACTCG-TCG-----GTCTCGTC-------CGCC---GGCTTGGCACTGACAGCTTCACAGTTACAACGGCTCTTCTGAGCTCCAGCTCGAGCGCATGAACGTCTACTTCAACGAGGTCAGT----CCTCG-ATATTTTTATT------------------GC--------ACCCA-C----------GATCTCCA-AA-ATTG-CCTTGTGTTGTCGT----TTGG----ACTTTGCTGACACC-TTATCGTCC-AGGCTTCCGGCAACAAGTATGTGCCTCGCGCTGTCCTCGTCGATCTCGAGCCCGGTACCATGGACGCCGTC

>Diaporthe_sclerotioides_CBS_296_67

GTCGGCACCATGT--TGCTTTCGCA-TCCTCCTCTGC----CCCTGAACCTCAGGC----TACCCCACTATCGCGACCACACCCACAG--TCGGGCTTCAAAAACATCACCAACACCCTGGGAAAAGCACCCAGATGCCACTCGGAAGACGCGTCAGATTGCTAACATGGACTTT-TTCTCGCCCATAGGTTCACCTTCAGACCGGCCAATGCGTAAGTTGCTTCCTGTCAAC-ACCACCCGCACCTTATCGCCA-CCTGTAGCTGACACGTTTCCCAGGGTAACCAAATCGGTGCTGCTTTCTGGTGCGTC------------------------CCAG--CTCCAG---CTCCAAGC-------------------------------CTGCCGCCGCG-ACGCTCGAC-------TCGCGACA-GCACTAGCTCGCGGCATCA-TT----ACTAACCGCAGCTCT-TAGGCAAACCATCTCTGGCGAGCACGGTCTCGACAGCAATGGCGTGTATGTACCTCCTATTCCC--TGCCCAACTG-----ATCCCGTC--CTCTCCTCC---GGCTTGGCACTGACAATTGCACAGTTACAACGGCACTTCCGAGCTCCAGCTCGAGCGTATGAATGTCTACTTCAACGAGGTAAGT----CAACA-GCCACGTCGTC------------------AA-TCCGAA-TTTAAAC----------AACTTACG-GC-ATGG-TTTCCTGCCGCCGC----CAAGC---GCCTTGCTAACGCG-TCATCGTCC-AGGCCTCCGGCAACAAGTATGTGCCTCGCGCCGTCCTCGTCGATCTCGAGCCCGGTACCATGGACGCCGTC

>Diaporthe_serafiniae_BRIP_55665a

GTCGGC-CCTTGCTGTGCTCTCGCA-TCCTCCTCTGC----CCCTGAGCCTCAGGC----TACCCCACCATCGCGACCACACCCACGG--TCGGGCTC--AAAACACCACCAGCACCCTGCGAAGAGCACCTAGATG-CGTTTGGAAGACGCGTCAGATTGCTAACATGAACTTT-TTCTTGCCCACAGGTTCACCTTCAGACCGGCCAATGCGTAAGTTGCCTCCTGTCAAC-ACCG-CCCGACCTTATCGCCA-CCCATAGCTGACACGTTTCCCAGGGTAACCAAATCGGTGCTGCTTTCTGGTGCGTA------------------------CCAG--CTCCAG---CTCCGAGC-------------------------------CTGCCACCGCG-ATGCTCGAC-------GCGCGACA-AGACCACCTCCAAGCATCG-TT----ACTGACCTTGTTTCTTTAGGCAAACCATCTCTGGCGAGCACGGTCTCGACAGCAATGGCGTGTATGTACCTCCTATTCCC--TGCCCA-CCG-----ATCTCGTC--CTCTCCTCC---GGCTTGGCACTGATGATCGCACAGTTACAACGGCACTTCCGAGCTCCAGCTCGAGCGCATGAACGTCTACTTCAACGAGGTAAGC----CTATG-GCCACGTCTTC------------------AA-TCCAAG-TTTGA-C----------CGTCT-CG-GC-ATGG-TTTACTGCCGCCGC----CAGG----GCCTTGCTAATGCG-CTCTCGCCC-AGGCCTCCGGCAACAAGTATGTGCCTCGCGCCGTCCTCGTCGATCTCGAGCCCGGTACCATGGACGCCGTC

>Diaporthe_siamensis_MFLUCC_10_0573a

NNNNNNNNNNNNNNNNNNNNNNNNNNNNNNNNNNNNNNNNNNNNNNNNNNNNNNNNNNNNNNNNNNNNNNNNNNNNNNNNNNNNNNNNNNNNNNNNNNNNNNNNNNNNNNNNNNNNNNNNNNNNNNNNNNNNNNNNNNNNNNNNNNNNNNNNNNNNNNNNNNNNNNNNNNNNNNNNNNNNNNNNNNNNNNNNNNNNNNNNNNNNNNNNNNNNNNNNNNNNNNNNNNNNNNNNNNNNNNNNNNNNNNNNNNNNNNNNNNNNNNNNNNNNNNNNNNNNNNNNNNNNNNNNNNNNNNNNNNNNNNNNNNNNNNNNNNNNNNNNNNNNNNNNNNNNNNNNNNNNNNNNNNNNNNNNNNNNNNNNNNNNNNNNNNNNNNNNNNNNNNNNNNNNNNNNNNNNNNNNNNNNNNNNNNNNNNNNNNNNNNNNNNNNNNNNAGCTGGCATCATCG-TT----ACTGACCTCTGCTCTTTAGGCAAACCATCTCTGGCGAGCACGGCCTCGACAGCAATGGCGTGTATGCACCTCCTATTCCC--TGCCCA-TTG-----ATCTCGCC--CTCTCCTCC---GGCTCGGCACTGACAATTGCACAGTTACAACGGCACTTCTGAGCTCCAGCTCGAGCGCATGAATGTCTACTTCAACGAGGTAAGT----CAACA-GCCACGTCGCC------------------AA-TTCAAA-TCTGA-C----------CATCTACG-GC-ATGG-TTCCATGCCGCCGC----CAAG----GTCTTGCTAACACA-TTATCGCCC-AGGCTTCCGGCAACAAGTATGTGCCTCGCGCCGTCCTCGTCGATCTCGAGCCCGGTACCATGGACGCCGTC

>Diaporthe_sinensis_ZJUP0033_4

GTCGGCCCTCTGCTGTGCTCTCGCA----TCCTCTGC---CCCCTGAGCCTCGGGC----TACCCCACCATCGCGACCACACCCACAG--TCAGGCCTC-AAAACATCACGAGCGTCCTGCGAGCTGCAGCCCGATG-CCCTTGGAAGACGCGCCAGATTGCTAACATGGACTTTTCCCTCGCCCATAGGTTCACCTTCAGACCGGCCAATGCGTAAGTAACCTCCTGTCAAC-ACC-TTAGGACCTTGCCGCCA-CCCCTAGCTGACACGTTTCCCAGGGTAACCAAATCGGTGCTGCTTTCTGGTGCGTC------------------------CCAG--CTCCAG---CCCCAGCCTCAGCTCCAGCCCCCGCT----CCAAGCCTGCTACGACCGCG-ACATTCGAT-------ACGCGACA-AGACAAGCTCCCAGCATCG-TTTT--ACTGACCTCGGCTGTTTAGGCAAACCATCTCTGGCGAGCACGGTCTCGACAGTAATGGCGTGTATGCACCTCCTATTCCC-TGGCCCA-CTG-----ACCTCGTC--CTTCCCTCC---GGCATGGCACTGATGATGGCGCAGTTACAACGGCACTTCCGAGCTGCAGCTCGAGCGCATGAACGTCTACTTCAACGAGGTGAGT----CAAGAATCCACGTCTTC------------------AA-GTCAAA-ATTGA-G----------CGTTCTCG-GC-ATGG-TTTATTGCCGCACC----AAAG----ACCTCGCTAACGCG-CCTTCGCCC-AGGCCTCCGGCAACAAGTATGTGCCTCGCGCCGTCCTCGTCGACCTCGAGCCCGGTACCATGGACGCCGTC

>Diaporthe_stewartii_CBS_193_36

??????????????????????????????????????????????????????????????????????????????????????????????????????????????????????????????????????????????????????????????????????????????????????????????????????????????????????????????????????????????????????????????????????????????????????????????????????????????????????????????????????????????????????????????????????????????????????????????????????????????????????????????????????????????????????????????????????????????????????????????????????????????????????????????????????????????????????????????????????????????????????????????????????????????????????????????????????????????????????????????????????????????????????????????????????????????????????????????????????????????????????????????????????????????????????????????????????????????????????????????????????????????????????????????????????????????????????????????

>Diaporthe_subordinaria_CBS_101711

GTCGGCCCTTTGCTGTGCTCTCGCA-TCCTCCTCTGCTGCTCCCTGAGCCTCAGGCT---TACCCCACCATCGCGACCACACCCACGT--TCAGGCCTC-AAAACACCACCAGCGCCCTGCAAAGTGCACCCAGATG-CCCTTGGAAAACGCGTCAGATTGCTAACATGGACTTTGTTCTCTCCTACAGGTTCACCTTCAGACCGGCCAATGCGTAAGTTGCCTCCTGTCAAC-ACCGCCGGGACCTTATCGCCA-CCCGTAGCTGACACGTTTCCCAGGGTAACCAAATCGGTGCTGCTTTCTGGTGCGTC------------------------CCAG--CTCCAG---CTCCAGCTCCAGCCCAAGCT----------CCAAGCCTGCCACGGCCCGC-GACATCGAC-------ACGCGACA-AGATTAGCTCGCAACATCT-TTAT--ACTGACCTCGGCTGTTTAGGCAAACCATCTCTGGCGAGCACGGTCTCGACAGCAATGGCGTGTATGCACCTCCTATTCCCC-TGCCCA-CTA-----ATCTCGTC--CTCCCCTCC---GGCTTGGTACTGATGTTGGCACAGTTACAACGGCACTTCTGAGCTCCAGCTCGAGCGCATGAACGTCTACTTCAATGAGGTGAGT----CAAAATGCCACGTCTTC------------------AA-TTCAAA-TTTGA-G----------CCTTCTCG-GC-ATGA-TTTCCTGCCGCCGC----AAAGA---CCTTTACTGACGCG-CTTTCGCCC-AGGCCTCCGGCAACAAGTATGTGCCTCGCGCCGTCCTCGTCGATCTCGAGCCCGGTACCATGGACGCCGTC

>Diaporthe_tecomae_CBS_100547

GTCGGC-CCATGCTG-GCTCTCGCA-TCCTCCTCTGC----CCCTGAGCCTCAGGC----TACCCCACCATCGCGACCACACCCACGG--TCGGGCCTC-AAAACACCACCAGCACCCTGCAAAGAGCACTCAGATG-CCCTTGGAACACGCGTCAGATTGCTAACATGAACTTT-TTCTCGCCCACAGGTTCACCTTCAGACCGGCCAATGCGTAAGTTGCCTCCTGTCAAC-ACC-GCCCGACCTTATCGCCA-CCCGTAGCTGACACGTTTCCCAGGGTAACCAAATCGGTGCTGCTTTCTGGTGCGTC------------------------CCAG--CTCCAGCTCCTCCGAGC-------------------------------CTACCACCGCG-ACCCTCGAC-------GCGCGACA-AGGCGAGCTCGAAGCATCG-AT----ACTGACCTTGGCTGTTTAGGCAAACCATCTCTGGCGAGCACGGTCTCGACAGCAATGGCGTGTACGTACCTCCTATTCCC--TGACTA-CTC-----ACCTCGTC--CTCTCCTCC---GGCTTGGCACTGACCATCGCACAGTTACAACGGCACTTCCGAGCTCCAGCTCGAGCGCATGAACGTCTACTTCAACGAGGTAAGT----CAACA-GCCACGTCGTC------------------AA-TTCAGA-CTTGA-C----------CCCCT-CG-GC-ATGG-TTAATTGCCGCCGC----CAAG----CCCTTGCTAAGGCG-TTTTCGCCC-AGGCCTCCGGCAACAAGTATGTGCCCCGCGCCGTCCTCGTCGATCTCGAGCCCGGTACCATGGACGCCGTC

>Diaporthe_tectonae_MFLUCC_12_0777

NNNNNNNNNNNNNNNNNNNNNNNNNNNNNNNNNNNNNNNNNNNNNNNNNNNNNNNNNNNNNNNNNNNNNNNNNNNNNNNNNNNNNNNNNNNNNNNNNNNNNNNNNNNNNNNNNNNNNNNNNNNNNNNNNNNNNNNNNNNNNNNNNNNNNNNNNNNNNNNNNNNNNNNNNNNNNNNNNNNNNNNNNNNNNNNNNNNNNNNNNNNNNNNNNNNNNNNNNNNNNNNNNNNNNNNNNNNNNNNNNNNNNNNNNNNNNNNNNNNNNNNNNNNNNNNNNNNNNNNNNNNNNNNNNNNNNNNNNNNNNNNNNNNNNNNC------------------------TCAG--CT----------AGAGT-------------------------------CGACCACCGCG-ACAATCGAC-------GCGCGACA-ACAGTAGCTCGTAGCATTG-AT----ACTGACATCAGCTC-CTAGGCAAACCATCTCTGGCGAGCACGGCCTCGACAGCAATGGCGTGTATGCACCTCCTATCCCC--TGCCTA-CGG-----GTCTCGTC--CTCTCCTAC---GCCTCGGCACTGACAATGGCACAGCTACAACGGCACTTCTGAGCTCCAGCTCGAGCGCATGAACGTCTACTTCAACGAGGTAAGC----CAAAG-CCCACGTTGTC------------------AA-TCCGGA-TTTGA-C----------CATCTGCG-GC-ACAA-TCCCCTGCCACCGC----CAAG----GCCTAGCTAACGCG-TTATCGTCC-AGGCCTCCGGCAACAAGTATGTGCCTCGCGCCGTCCTCGTCGATCTCGAGCCCGGTACCATGGATGCCGTC

>Diaporthe_tectonendophytica_MFLUCC_13_0471

????????????????????????????????????????????????????????????????????????????????????????????????????????????????????????????????????????????????????????????????????????????????????????????????????????????????????????????????????????????????????????????????????????????????????????????????????????????????????????????????????????????????????CTCT-CAG---CTCC-AGC-------------------------------CTACCACCGCG-ACCCTCGAC-------GCGCGACA-AGGCGAGCTCGAAGCATCG-AT----ACTGACCTCGGTTCTTTAGGCAAACCATCTCTGGCGAGCACGGTCTCGACAGCAATGGCGTGTATGTACCTCCTATTCCC--TGCCCA-CTG-----CCCTCGTC--CTCTCCTCC--GGGCTTGGCACTGATGATCGCGCAGTTACAACGGCACTTCCGAGCTCCAGCTCGAGCGCATGAACGTCTACTTCAACGAGGTAAGT----CAATA-GCCACGTCGCC------------------AA-TTCAAT-TTTGA-C----------CGTCT-CG-GT-ATGA-TGAACTGCCGCCGC----CAAG----CCATTGCTAACGCG-TTTTCCCCC-AGGCCTCCGGCAACAAGTATGTGCCCCGCGCCGTCCTCGTCGATCTCGAGCCCGGTACCATGGATGCCGTC

>Diaporthe_terebinthifolii_CBS_133180

GTCGGC-CCATGCT--GCTCTCGCA-TCCTCCTCTGC----CCCTGAACCTCAGGC----TACCCCACCATCGCGACCACACCCACGG--TCGGGCCTC-AAAACACCACCAGCACCTTGCGAAGACCACCCAGATG-CCCTTGGAATACGCGTCAGATTGCTAACATGACCTTT-TTCTCGCCCACAGGTTCACCTTCAGACCGGCCAATGCGTAAGTTGCCTCCTGTCAAC-ACC-GCCCGACCTTATCGCCA-CCCGTAGCTGACACGTTTCCCAGGGTAACCAAATCGGTGCTGCTTTCTGGTGCGTC------------------------CCAG--CTCCAG---CTCCGAGC-------------------------------CTACCACCGCG-ACCCTCGAC-------GCGCGACA-AGGCGAGCTCGAAGCATCG-AT----ACTGACCTTGGCTGTTTAGGCAAACCATCTCTGGCGAGCACGGTCTCGACAGCAATGGCGTGTACGTACCTCCTATTCCC--TGACTA-CTG-----ACCTCGTC--CTCTCCTCC---GGCTTGGCACTGACGATCGCACAGTTACAACGGCACTTCCGAGCTCCAGCTCGAGCGCATGAACGTCTACTTCAACGAGGTAAGT----CAATA-GCCACGTCGTC------------------AA-TTCAAA-TTTGA-C----------CGTCT-CG-GC-ATGA-TTGATAACCGCCGC----CAAA----CCCTTGCTAACGCG-CTCTCGCCC-AGGCCTCCGGCAACAAGTATGTGCCCCGCGCCGTCCTCGTCGATCTCGAGCCCGGTACCATGGACGCCGTC

>Diaporthe_thunbergiicola_MFLUCC_12_0033

??????????????????????????????????????????????????????????????????????????????????????????????????????????????????????????????????????????????????????????????????????????????????????????????????????????????????????????????????????????????????????????????????????????????????????????????????????????????????????????????????????????????????????????????????????????????????????????????????????????????????????????????????????????????????????????????????????????????????????????????????????????????????????????????????????????????????????????????????????????????????????????????????????????????????????????????????????????????????????????????????????????????????????????????????????????????????????????????????????????????????????????????????????????????????????????????????????????????????????????????????????????????????????????????????????????????????????????????

>Diaporthe_tulliensis_BRIP_62248a

GTCGGC-CCATGC--TGCTTTCGCA----TCCTCTGC----CCCTGAACCTCAGGC----TACCCCACCATCGCGACCACACCCACAG--TCAGGCCTCAAAAACACCATCAACACCCTGGGAAAGACCACCCAGATGCTCTCAAAAGACGCGTCGGATTGCTAACATGGACTTT-TTCTCGCCCACAGGTTCACCTCCAGACTGGCCAATGCGTAAGTTGCTTCCTGTCAAC-ACCG-CCCGACCTTATCGCCA-CCTGTAGCTGACACGTTTCCCAGGGTAACCAAATCGGTGCTGCTTTCTGGTGCGTC------------------------CCAG--CTCCAG---CTCAGAGT-------------------------------CGACCACCGCG-ACAATCGAC-------GCGCGACA-ACAGTAGCTCGTAGCATTG-TT----ACTGACATCGGCTC-CTAGGCAAACCATCTCTGGCGAGCACGGCCTCGACAGCAATGGCGTGTATGCACCTCCTATTCCC--TGCCTA-CTG-----GTCTCGTC--CTCTCCTAC---GCCTTGGCACTGACAATGGCACAGCTACAACGGCACTTCTGAGCTCCAGCTCGAGCGCATGAACGTCTACTTCAACGAGGTAAGC----CAAAG-CCCACGTTGTC------------------AA-TCCGGA-TTTGA-C----------CATCTGCG-GC-ACAA-TCCCCTGCCACCGC----CAAG----GCCTAGCTAACGCG-TTATCGTCC-AGGCCTCCGGCAACAAGTATGTGCCTCGCGCCGTCCTCGTCGATCTCGAGCCCGGTACCATGGATGCCGTC

>Diaporthe_ueckerae_FAU_656

????????????????????????????????????????????????????????????????????????????????????????????????????????????????????????????????????????????????????????????????????????????????????????????????????????????????????????????????????????????????????????????????????????????????????????????????????GTGCTGCTTTCTGGTGCGTC------------------------GCAGCTCTCCAG---CTCCAAGC-------------------------------CTACCGCCGCGAACCCTCGAC-------GCGCGACA-AGGCGAGCTCGAAGCATCG-AT----ACTGACCTCGGTTCTTTAGGCAAACCATCTCCGGCGAGCACGGTCTCGACAGCAATGGCGTGTATGCACCTCCTATTCCC--TGCCCG-TGG-----CCCTCATC--CTCTTCTCC---GGCTTGGCACTGATGATCGCACAGTTACAACGGCACTTCCGAGCTCCAGCTCGAGCGCATGAACGTCTACTTCAACGAGGTCAGT----CAATA-GCCACGTCGTC------------------AA-TTCAAA-TTTGA-A----------CCTCT-CG-GC-ATGG-TCAACTGCCGCCGC----CAAG----CCCCTGCTAACGCG-TTTTCGCCC-AGGCCTCCGGCAACAAGTATGTGCCCCGCGCCGTCCTCGTCGATCTCGAGCCCGGTACCATGGACGCCGTC

>Diaporthe_unshiuensis_CGMCC3_17569

????????????????????????????????????????????????????????????????????????????????????????????????????????????????????????????????????????????????????????????????????????????????????????????????????????????????????????????????????????????????????????????????????????????????????????????????????????????????????????????????????????????????????????????????????????????????????????????????????????????????????????????????????????????????AGCTCGAAGCATCG-AT----ACTGACCTCCGTCCTTTAGGCAAACCATCTCTGGCGAGCACGGTCTCGACAGCAATGGCGTGTATGCACCTCCTTTTCCC--TGCCCG-TGG----CCCCTCGTC--CTCTCCTCC---GGCTTGGCACTGATGATCGCACAGTTACAACGGCACTTCCGAGCTCCAGCTCGAGCGCATGAACGTCTACTTCAACGAGGTAAGT----CAATA-GCCAGGTCGTC------------------AA-TTCAAA-TTTGA-CCCTCTCGGCACCTTT-CG-GC-ATGG-TCAACTGCTGCCGC----CAAG----CCTTTGCTAACGCG-TTTTCGCCC-AGGCCTCCGGCAACAAGTATGTGCCCCGCGCCGTCCTCGTCGATCTCGAGCCCGGTACCATGGACGCCGTC

>Diaporthe_vexans_CBS_127_14

GTCGGC-CCATGCTGTGCTCTCGCA-TCCTCCTCTGC----CCCTGAGCCTCAGGC----TACCCCACCATCGCGGCCACACCAACGA--TCGGGCCTC-AAAACACCACCAGCACCTTGCGAAGAGCACCCAG------------AGACGCGTCAGATTGCTAACATGGCGTTT-TTCTTGCCCACAGGTTCACCTTCAGACCGGCCAATGCGTAAGTTGCCTACTGTCAAC-ACC-GTCGGACCTTCTCGCCA-CCCGTAGCTGACACGTTTCCCAGGGTAACCAAATCGGTGCTGCTTTCTGGTGCGTC------------------CCAGCTCCAG--CTCCAG---CTCTCTGC-------------------------------CTACCACCGCG-ACCCTCGAC-------GCGCGACA-AGGCTAGCTCGTAGTCTCGAAT----ACTGACCTCGAATCCTTAGGCAAACCATCTCTGGCGAGCACGGTCTCGACAGCAATGGCGTGTATGCACCTCCTATTCCC--TGACTC-CTGACCTCGTCTCGTC--CTCTCCTCC---GGCCTGGCACTGATGATCGAACAGTTACAACGGCACTTCCGAGCTCCAGCTCGAGCGCATGAACGTCTACTTCAACGAGGTAAGCTAAACAACA-GCCACGTCACC------------------AA-TTCAGA-TTTGA-C----------CGTCC-CG-GC-ATGG-TTGACTGCCGCCGC----CAAAGACTGACTTGCTAACGCG-TTCTCGCCC-AGGCCTCCGGCAACAAGTATGTGCCCCGCGCCGTCCTCGTCGATCTCGAGCCCGGTACCATGGACGCCGTC

>Diaporthe_yunnanensis_CGMCC_3_18289

NNNNNNNNNNNNNNNNNNNNNNNNNNNNNNNNNNNNNNNNNNNNNNNNNNNNNNNNNNNNNNNNNNNNNNNNNNNNNNNNNNNNNNNNNNNNNNNNNNNNNNNNNNNNNNNNNNNNNNNNNNNNNNNNNNNNNNNNNNNNNNNNNNNNNNNNNNNNNNNNNNNNNNNNNNNNNNNNNNNNNNNNNNNNNNNNNNNNNNNNNNNNNNNNNNNNNNNNNNNNNNNNNNNNNNNNNNNNNNNNNNNNNNNNNNNNNNNNNNNNNNNNNNNNNNNNNNNNNNNNNNNNNNNNNNNGGTGCTGCTTTCTGGTGCGTC------------------------CCAG--CTCCAG---CTCCAAGC-------------------------------CTACCATCGCG-ACTCTCGTC-------GCGCGACA-ACACTAGCTTGCAGCATCG-TT----ACTGACATCTGCTCTTTAGGCAAACCATCTCTGGCGAGCACGGCCTCGACAGCAATGGCGTGTATGTGCCTCCTATTCTC--TGCCCA-ATG-----ATCTCGTC--CTCTCCTCC---CGCTTGGCACTGACAATTGCATAGTTACAACGGCACTTCCGAGCTCCAGCTCGAGCGCATGAACGTCTACTTCAACGAGGTCAGT----AAACA-GCCACGTCGCC------------------AA-TTCACA-TCTGA-C----------CATCTACG-AC-ATGG-TTCCATGTCGCCGC----CAAG----GTCTTGCTAACGCG-TTATCGCCC-AGGCCTCCGGCAACAAGTATGTGCCTCGCGCCGTCCTCGTCGATCTCGAGCCCGGTACCATGGATGCCGTC

>Phomopsis_glabrae_SCHM_3622

??????????????????????????????????????????????????????????????????????????????????????????????????????????????????????????????????????????????????????????????????????????????????????????????????????????????????????????????????????????????????????????????????????????????????????????????????????????????????????????????????????????????????????????????????????????????????????????????????????????????????????????????????????????????????????????????????????????????????????????????????????????????????????????????????????????????????????????????????????????????????????????????????????????????????????????????????????????????????????????????????????????????????????????????????????????????????????????????????????????????????????????????????????????????????????????????????????????????????????????????????????????????????????????????????????????????????????????????

>Phomopsis_micheliae

??????????????????????????????????????????????????????????????????????????????????????????????????????????????????????????????????????????????????????????????????????????????????????????????????????????????????????????????????????????????????????????????????????????????????????????????????????????????????????????????????????????????????????????????????????????????????????????????????????????????????????????????????????????????????????????????????????????????????????????????????????????????????????????????????????????????????????????????????????????????????????????????????????????????????????????????????????????????????????????????????????????????????????????????????????????????????????????????????????????????????????????????????????????????????????????????????????????????????????????????????????????????????????????????????????????????????????????????

>Phomopsis_vitimegaspora_STE_U2675

??????????????????????????????????????????????????????????????????????????????????????????????????????????????????????????????????????????????????????????????????????????????????????????????????????????????????????????????????????????????????????????????????????????????????????????????????????????????????????????????????????????????????????????????????????????????????????????????????????????????????????????????????????????????????????????????????????????????????????????????????????????????????????????????????????????????????????????????????????????????????????????????????????????????????????????????????????????????????????????????????????????????????????????????????????????????????????????????????????????????????????????????????????????????????????????????????????????????????????????????????????????????????????????????????????????????????????????????

>Diaporthe_subellipicola_KUMCC_17_0153

GTCGGC-CCATGCTGTGCTCTCGCA----TCCTCTGC----CCCTGAGCCTGAGGC----TACCCCACCATCGCGACCACACCCATGG--TCGGGCCTC-AAAACACCACCAGCGCCCTGCGAAGAGCACCCAGATG-CTACTGGAACACGCGTCAGATTGCTAACATGACCTTT-TTCTCGCCCACAGGTTCACCTCCAGACCGGCCAATGCGTAAGTTGCCTCCTGTCAAC-ACC-GCCAGACCTTATCGCCA-CCCGTAGCTGACACGTTTCCCAGGGTAACCAAATCGGTGCTGCTTTCTGGTGCGTC------------------------GCAGCTCTCCAG---CTCCAAGC-------------------------------CTACCACCGCG-ACCCTCGAC-------GCGCGACA-AGGCGAGCCCGAAGCATCG-AT----ACTGACCTCGTTTCTTTAGGCAAACCATCTCTGGCGAGCACGGTCTCGACAGCAATGGCGTGTATGCACCTCCTATTCCC--TGCCCA-CTG-ACTGCCCTGGTC--CTCTCCTCC---GGCTTGGCACTGATGATCGCACAGTTACAACGGCACTTCCGAGCTCCAGCTCGAGCGCATGAACGTCTACTTCAACGAGGTATGT----CAACG-GCCACGTCGTC------------------AA-TTCAAA-TTTGA-C----------CCTCT-CG-GC-GTGG-TCAACCGCCGCCGC----CAAG----CCCTTGCTAACGCG-TTTTCCCCC-AGGCTTCCGGCAACAAGTATGTGCCCCGCGCCGTCCTCGTCGATCTCGAGCCCGGTACCATGGACGCCGTC

>Diaporthe_masirevicii_BRIP_57892a

GTCGGC-CCATGCTGTGCTCTCGCA----TCCTCTGC----CCCTGAGCCTGAGGC----TACCCCACCATCGCGACCACACCCATGG--TCGGGCCTC-AAAACACCACCGTCGCCCTGCGAAGAGCACCCAGATG-CTACTGGAACACGCGTCAGATTGCTAACATGACCTTT-TTCTCGCCCACAGGTTCACCTTCAGACCGGCCAATGCGTAAGTTGCCTCCTGTCAAC-ACC-GCCAGACCTTATCGCCA-CCCGTAGCTGACACGTTTCCCAGGGTAACCAAATCGGTGCTGCTTTCTGGTGCGTC------------------------GCAGCTCTCCAG---CTTCAAGC-------------------------------CTACCACCGCG-ACCCTCGAC-------GCGCGACA-AGGCGAGCTCGAAGCATCG-AT----ACTGACCTCGTTTCTTTAGGCAAACCATCTCTGGCGAGCACGGTCTCGACAGCAATGGCGTGTATGCACCTCCTATTCCC--TGCCCA-CTG-ACTGCCCTGGTC--CTCTCCTCC---GGCTTGGCACTGATGATCGCACAGTTACAACGGCACTTCCGAGCTCCAGCTCGAGCGCATGAACGTCTACTTCAACGAGGTATGT----CAACG-GCCACGTCGTC------------------AA-TTCAAA-TTTGA-A----------CCTCT-CG-GC-ATGG-TCAACTGCCGCCGC----CAAG----CCCTTGCTAACGCG-TTTTCCCCC-AGGCTTCCGGCAACAAGTATGTGCCCCGCGCCGTCCTCGTCGATCTCGAGCCCGGTACCATGGACGCCGTC

>Diaporthe_sojae_CBS_139282

????????????????????????????????????????????????????????????????????????????????????????????????????????????????????????????????????????????????????????????????????????????????????????????????????????????????????????????????????????????????????????????????????????????????????????????????????GTGCTGCTTTCTGGTGCGTC------------------------GCAGCTCTCCAG---CTCCAAGC-------------------------------CTACCACCGCG-ACCCTCGAC-------GCGCGACA-AGGCGAGCTCGAAGCATCG-AT----ACTGACCTCGTTTCTTTAGGCAAACCATCTCTGGCGAGCACGGTCTCGACAGCAATGGCGTGTATGCACCTCCTATTCCC--TGCCCA-CTG-ACTGCCCTGGTC--CTCCCCGCC---GGCTTGGCACTGATGATCGCACAGTTACAACGGCACTTCCGAGCTCCAGCTCGAGCGCATGAACGTCTACTTCAACGAGGTATGT----CAACG-GCCACGTCGTC------------------AA-TTCAAA-TTTGA-C----------CCTCT-CG-GC-ATGGATCAACCGCCGCCGC----CAAG----CCCTTGCTAACGCG-TTTTCCCCC-AGGCTTCCGGCAACAAGTATGTGCCCCGCGCCGTCCTCGTCGATCTCGAGCCCGGTACCATGGACGCCGTC

>Diaporthe_sambucusii_CFCC_51986

GTCTGC-CCATGC--TGCTCTCGCA-TCCTCCTCTGC----CCCTGAGCCTCAGCC----TACCCCACCATCGCGACCACACTCCCACAGCAGGGCCTC-AAAACATCACCAGCACCTTGCGATGAGCACCCGTGTG-CCCTTGGAACACGCGTCAGATTGCTAACATGGACTTT-TTCTCGCCTGCAGGTTCACCTCCAGACCGGCCAATGCGTAAGTTGCTCCCTGTCAAC-ACTG-CCCGACCTTATCGCCA-CCCGTAGCTGACACGTTTCCCAGGGTAACCAAATCGGTGCTGCTTTCTGGTGCGTC------------CCAGCTCCAGCTCCAG--CTCCAG---CTCCAAGC-------------------------------CTACCGGCGCG-ATGCTCGAC-------GCGCGACA-AGACCAGCTCGCAACATTG-TT----ACTGACCTCGGCTCTGTAGGCAAACCATCTCTGGCGAGCACGGCCTCGACAGCAATGGCGTGTATGCACCTCCTATTCCC--TGCCCA-CTG-----ATCTCGTC--CTGTCCTCC---GGCTTGACACTGATGATTGCACAGTTACAACGGCACTTCCGAGCTCCAGCTCGAGCGCATGAACGTCTACTTCAACGAGGTATGT----CAATG-GCCACGTCGTC------------------AA-TCCAAA-TTTGC-C----------CATCTACT-GC-GCGG-CGTGCTGCCGTAGC----CAAA----GCCTTGCTAACGCG-TTGTCGCCC-AGGCTTCCGGCAACAAGTATGTGCCCCGCGCTGTCCTCGTCGATCTCGAGCCCGGTACCATGGACGCCGTC

>Diaporthe_vochysiae_LGMF1583

GTCGGC-CCATGCTGTGCTCTCGCA----TCCTCTGC----CCCTGAGCCTGAGGC----TACCCCACCATCGCGACCACACCCACGG--TCGGGCCTC-AAAACACCACCAGCTCCCTGCGAAGAGCACCCAGATG-CCCTTGGAACACGCGTCAGATTGCTAACATGACCTTT-TTCTCGCCTGCAGGTTCACCTTCAGACCGGCCAATGCGTAAGTTGCTCCCCGTCAAC-ACC-GCCGGACCTTATCGCCA-CCCGTAGCTGACACGTTTACCAGGGTAACCAAATCGGTGCTGCTTTCTGGTGCGTC------------------------GCAGCTCTCCAG---CTCCAAGC-------------------------------CTACCACCGCGAACCCTCGAC-------GCGCGACA-AGGCGAGCTCGAAGCATCG-AT----ACTGACCTCGTTCCTTTAGGCAAACCATCTCTGGCGAGCACGGTCTCGACAGCAATGGCGTGTATGCACCTCCTATTCCC--TGCCCG-TGG-----CCCTCGTA--CTCTTCTCC---GGCTTGGCACTGATGATCGCACAGTTACAACGGCACTTCCGAGCTCCAGCTCGAGCGCATGAACGTCTACTTCAACGAGGTCAGT----CAATA-GCCACGTTGTC????????????????????????????????????????????????????????????????????????????????????????????????????????????????????????????????????????????????????????????????????????????????????????

>Diaporthe_endophytica_CBS_133811

GTCGGC-CCATGCTGTGCTCTCGCA----TCCTCTGC----CCCTGAGCCTGAGGC----TACCCCACCATCGCGACCACACCCATGG--TCGGGCCTC-AAAATACCACCAGCGCCCTGCGAAGAGCACCCAGATG-CTCTTGGAACACGCGTCAGATTGCTAACATGACCTTT-TTCTCGCCCACAGGTTCACCTTCAGACCGGCCAATGCGTAAGTTGCCTCCTGTCAAC-ACC-GCCAGGGCTTATCGCCA-CCCGTAGCTGACACGTTTCCCAGGGTAACCAAATCGGTGCTGCTTTCTGGTGCGTC------------------------GCAGCTCTCCAG---CTCCAAAC-------------------------------CTACCACCGCG-GCCCTCGAC-------GCGCGACA-AGGCGAGCTCGAAGCATCG-AT----ACTGACCTCGTTTCTCTAGGCAAACCATCTCTGGCGAGCACGGTCTCGACAGCAATGGCGTGTATGCACCTCCTATTCCC--TGCCCA-CTG-ACTGCCCTGGTC--CTCTCCTCC---GGCTTGGCACTGATGATCGCACAGTTACAACGGCACTTCCGAGCTCCAGCTCGAGCGCATGAACGTCTACTTCAACGAGGTATGT----CAACG-GCCACGTCGTC------------------AA-TTCAAA-TTTGA-A----------GCTCT-CG-GC-ATGG-TCAACTGCCGCCGC----CAAG----CCCTTGCTAACGCG-TTTTCCCCC-AGGCTTCCGGCAACAAGTATGTGCCCCGCGCCGTCCTCGTCGATCTCGAGCCCGGTACCATGGACGCCGTC

>Diaporthe_phaseolorum_CBS_113425

GTCGGC-CCATGCTGTGCTCTCGCA----TCCTCTGC----CCCTGAGCCTGAGGC----TACCCCACCATCGCGACCACACCCATGG--TCGGGCCTC-AAAACACCACCAGCGCCCTGTGAAGAGCACCCAGATG-CTCTTGGAACACGCGTCAGATTGCTAACATGACCTTT-TTCTCGCCCACAGGTTCACCTTCAGACCGGCCAATGCGTAAGTTGCCTCCTGTCAAC-ACC-GCCAGACCTTATCGCCA-CCCGTAGCTGACACGTTTCCCAGGGTAACCAAATCGGTGCTGCTTTCTGGTGCGTC------------------------GCAGCTCTCCAG---CTCCAAGC-------------------------------CTACCACCGCG-ACCCTCGAC-------GCGCGACA-AGGCGAGCTCGAAGCATCG-AT----ACTGACCTCGTTTCTTTAGGCAAACCATCTCTGGCGAGCACGGTCTCGACAGCAATGGCGTGTATGCACCTCCTATTCCC--TGCCCA-CTG-ACTGCCCTGGTC--CTCCCCGCC---GGCTTGGCACTGATGATCGCACAGTTACAACGGCACTTCCGAGCTCCAGCTCGAGCGCATGAACGTCTACTTCAACGAGGTATGT----CAACG-GCCACGTCGTC------------------AA-TTCAAA-TTTGA-C----------CCTCT-CG-GC-ATGGATCAACCGCCGCCGC----CAAG----CCCTTGCTAACGCG-TTTTCCCCC-AGGCTTCCGGCAACAAGTATGTGCCCCGTGCCGTCCTCGTCGATCTCGAGCCCGGTACCATGGACGCCGTC

>Diaporthe_caliensis_STMA_22040

???????????????????????????????????????????????????????????????????????????????????????????????????????????????????????????????????????????????????????????????????????????????????????????????????????????????????????????????????????????????????????????????????????????????????????????????????GGTGCTGCTTTCTGGTGCGTC------------------------GCAGCTCTCCAG---CTCCAAGCC------------------------------CCACCACCGCG-AC-CTCGAC-------GCGCGACA-AGGCGAGCTCGAAGCGTCG-AT----ACTGACCTCGTTTCTTTAGGCAAACCATCTCTGGCGAGCACGGTCTCGACAGCAATGGCGTGTATGCACCTCCTATTCCC--TGCCCA-CTG-ACTGCCCTGGTC--CTCTCCTCC---GGCTTGGCACTGATGATCGCACAGTTACAACGGCACTTCCGAGCTCCAGCTCGAGCGCATGAACGTCTACTTCAACGAGGTATGT----CAACG-GCCACGTCGTC------------------AA-TTCAAA-TTTGA-C----------CCTCT-CG-GC-ATGGATCAACCGCCGCCGC----CAAG----CCCTTGCTAACGCG-TTTTCTCCC-AGGCTTCCGGCAACAAGTATGTGCCCCGCGCCGTCCTCGTCGATCTCGAGCCCGGTACCATGGACGCCGTT

**References for Supporting Information**

1. Crous PW, Wingfield MJ, Schumacher RK et al. 2014. Fungal Planet description sheets: 281–319. Persoonia 33:212–89. https://doi.org/10.3767/003158514X685680

2. Dissanayake AJ, Camporesi E, Hyde KD, Zhang W, Yan JY, Li XH. 2017. Molecular phylogenetic analysis reveals seven new *Diaporthe* species from Italy. Mycosphere 8:853–877. https://doi.org/10.5943/mycosphere/8/5/4

3. Yang Q, Fan X-L, Guarnaccia V, Tian C-M. 2018. High diversity of *Diaporthe* species associated with dieback diseases in China, with twelve new species described. MycoKeys 39:97–149. https://doi.org/10.3897/mycokeys.39.26914

4. Gomes RR, Glienke C, Videira SIR, Lombard L, Groenewald JZ, Crous PW. 2013. *Diaporthe*: a genus of endophytic, saprobic and plant pathogenic fungi. Persoonia 31:1–41. https://doi.org/10.3767/003158513X666844

5. Li WJ, McKenzie EHC, Liu JK, D Bhat J, Dai D-Q, Camporesi E, Tian Q, Maharachchikumbura SSN, Luo Z-L, Shang Q-J, Zhang J-F, Tangthirasunun N, Karunarathna SC, Xu J-C, Hyde KD. 2020. Taxonomy and phylogeny of hyaline-spored coelomycetes. Fungal Divers 100:279–801. https://doi.org/10.1007/s13225-020-00440-y

6. Thompson SM, Tan YP, Shivas RG, Neate SM, Morin L, Bissett A, Aitken EAB. 2015. Green and brown bridges between weeds and crops reveal novel *Diaporthe* species in Australia. Persoonia 35:39–49. https://doi.org/10.3767/003158515X687506

7. Huang F, Udayanga D, Wang X, Hou X, Mei X, Fu Y, Hyde KD, Li H. 2015. Endophytic *Diaporthe* associated with *Citrus*, a phylogenetic reassessment with seven new species from China. Fungal Biol 119:331–347. https://doi.org/10.1016/j.funbio.2015.02.006

8. Matio Kemkuignou B, Schweizer L, Lambert C, Anoumedem EGM, Kouam SF, Stadler M, Marin-Felix Y. 2022. New polyketides from the liquid culture of *Diaporthe breyniae* sp. nov. (Diaporthales, Diaporthaceae). MycoKeys 90:85–118. https://doi.org/10.3897/mycokeys.90.82871

9. Hyde KD, Dong Y, Phookamsak R et al. 2020. Fungal Diversity Notes 1151–1276: taxonomic and phylogenetic contributions on genera and species of fungal taxa. Fungal Divers 100:5–277. https://doi.org/10.1007/s13225-020-00439-5

10. Tennakoon DS, Kuo CH, Maharachchikumbura SSN, Thambugala KM, Gentekaki E, Phillips AJL, Bhat DJ, Wanasinghe DN, de Silva NI, Promputtha I, Hyde KD. 2021. Taxonomic and phylogenetic contributions to *Celtis formosana*, *Ficus ampelas*, *F. septica*, *Macaranga tanarius* and *Morus australis* leaf litter inhabiting microfungi. Fungal Divers 108:1–215. https://doi.org/10.1007/s13225-021-00474-w

11. Iantas J, Savi DC, Schibelbein RdS, Noriler SA, Assad BM, Dilarri G, Ferreira H, Rohr J, Thorson JS, Shaaban KA, Glienke C. 2021. Endophytes of Brazilian medicinal plants with activity against phytopathogens. Front Microbiol 12:714750. https://doi.org/10.3389/fmicb.2021.714750

12. Chang CQ, Cheng YH, Xiang MM, Jiang ZD. 2005. New species of *Phomopsis* on woody plants in Fujian Province. Mycosystema 24:6–11.

13. de Silva NI, Maharachchikumbura SSN, Thambugala KM, Bhat DJ, Karunarathna SC, Tennakoon DS, Phookamsak R, Jayawardena RS, Lumyong S, Hyde KD. 2021. Morphomolecular taxonomic studies reveal a high number of endophytic fungi from *Magnolia candolli* and *M. garrettii* in China and Thailand. Mycosphere 11:163–237. https://doi.org/10.5943/mycosphere/12/1/3

14. Mapook A, Hyde KD, McKenzie EHC, Jones EBG, Bhat DJ, Jeewon R, Stadler M, Samarakoon MC, Malaithong M, Tanunchai B, Buscot F, Wubet T, Purahong W. 2020. Taxonomic and phylogenetic contributions to fungi associated with the invasive weed *Chromolaena odorata* (Siam weed). Fungal Divers 101:1–175. https://doi.org/10.1007/s13225-020-00444-8

15. Huang F, Hou X, Dewdney MM, Fu Y, Chen G, Hyde KD, Li H. 2013. *Diaporthe* species occurring on citrus in China. Fungal Divers 61:237–250. https://doi.org/10.1007/s13225-013-0245-6

16. Gao Y, Liu F, Cai L. 2016. Unravelling *Diaporthe* species associated with *Camellia*. Syst Biodivers 14:102–117. https://doi.org/10.1080/14772000.2015.1101027

17. 10. Udayanga D, Castlebury LA, Rossman AY, Chukeatirote E, Hyde KD. 2015. The *Diaporthe sojae* species complex, phylogenetic re-assessment of pathogens associated with soybean, cucurbits and other field crops. Fungal Biol 119:383–407. https://doi.org/10.1016/j.funbio.2014.10.009

18. Van Rensburg JCJ, Lamprecht SC, Groenewald JZ, Castlebury LA, Crous PW. 2006. Characterization of *Phomopsis* spp. associated with die-back of rooibos (*Aspalathus linearis*) in South Africa. Stud Mycol 55:65–74. https://doi.org/10.3114/sim.55.1.65

19. Crous PW, Wingfield MJ, Chooi Y-H et al. 2020. Fungal Planet description sheets: 1042–1111. Persoonia 44:301–459. https://doi.org/10.3767/persoonia.2020.44.11

20. Udayanga D, Castlebury LA, Rossman AY et al. 2014. Insights into the genus *Diaporthe*: phylogenetic species delimitation in the *D. eres* species complex. Fungal Divers 67:203–229. https://doi.org/10.1007/s13225-014-0297-2

21. Crous PW, Carnegie AJ, Wingfield MJ et al. 2019. Fungal Planet description sheets: 868–950. Persoonia 42:291–473. https://doi.org/10.3767/persoonia.2019.42.11

22. Dong Z, Manawasinghe IS, Huang Y, Shu Y, Phillips AJL, Dissanayake AJ, Hyde KD, Xiang M, Luo M. 2021. Endophytic *Diaporthe* associated with *Citrus grandis* cv. *tomentosa* in China. Front Microbiol 11:3621. https://doi.org/10.3389/fmicb.2020.609387

23. Dissanayake AJ, Chen Y-Y, Liu J-K. 2020. Unravelling *Diaporthe* species associated with woody hosts from karst formations (Guizhou) in China. J Fungi 6:251. https://doi.org/10.3390/jof6040251

24. Sun W, Huang S, Xia J, Zhang X, Li Z. 2021. Morphological and molecular identification of *Diaporthe* species in south-western China, with description of eight new species. MycoKeys 77:65–95. https://doi.org/10.3897/mycokeys.77.59852

25. Manawasinghe IS, Dissanayake AJ, Li X, Liu M, Wanasinghe DN, Xu J, Zhao W, Zhang W, Zhou Y, Hyde KD, Brooks S, Yan J. 2019. High genetic diversity and species complexity of *Diaporthe* associated with grapevine dieback in China. Front Microbiol 10:1936. https://doi.org/10.3389/fmicb.2019.01936

26. Guarnaccia V, Crous PW. 2017. Emerging citrus diseases in Europe caused by *Diaporthe* spp. IMA Fungus 8:317–334. https://doi.org/10.5598/imafungus.2017.08.02.07

27. Crous PW, Summerell BA, Shivas RG et al. 2011. Fungal Planet description sheets: 92–106. Persoonia 27:130–162. https://doi.org/10.3767/003158511X617561

28. Huang S, Xia J, Zhang X, Sun W. 2021. Morphological and phylogenetic analyses reveal three new species of *Diaporthe* from Yunnan, China. MycoKeys 78:49–77. https://doi.org/10.3897/mycokeys.78.60878

29. da Silva RMF, Soares AM, Pádua APSL, Firmino AL, Souza-Motta CM, da Silva GA, Plautz HL, Jr Bezerra JDP, Paiva LM, Ryvarden L, Oliani LC, de Mélo MAC, Magalhães OMC, Pereira OL, Oliveira RJV, Gibertoni TB, Oliveira TGS, Svedese VM, Fan XL. 2019. Mycological Diversity Description II. Acta Bot Bras 33:163–173. https://doi.org/10.1590/0102-33062018abb0411

30. Doilom M, Dissanayake AJ, Wanasinghe DN, Boonmee S, Liu J-K, Bhat DJ, Taylor JE, Bahkali AH, McKenzie EHC, Hyde KD. 2017. Microfungi on *Tectona grandis* (teak) in Northern Thailand. Fungal Divers 82:107–182

31. Milagres CA, Belisário R, Silva MA, Lisboa DO, Pinho DB, Furtado GQ. 2018. A novel species of *Diaporthe* causing leaf spot in *Pachira glabra*. Trop Plant Pathol 43:460–467. https://doi.org/10.1007/s40858-018-0242-0

32. Crous PW, Wingfield MJ, Richardson DM et al. 2016. Fungal Planet description sheets: 400–468. Persoonia 36:316–458. https://doi.org/10.3767/003158516X692185

33. Petrović K, Riccioni L, Dordević V, Tubic SB, Miladinović J, Ceran M, Rajković D. 2018. *Diaporthe pseudolongicolla* - the new pathogen on soybean seed in Serbia. Ratarstvo i povrtarstvo 55:103–109. https://doi.org/10.5937/RATPOV55-18582

34. Santos L, Phillips AJL, Crous PW. 2017. *Diaporthe* species on Rosaceae with descriptions of *D. pyracanthae* sp. nov. and *D. malorum* sp. nov. Mycosphere 8:485–511. https://doi.org/10.5943/mycosphere/8/5/2

35. Marin-Felix Y, Hernandez-Restrepo M, Wingfield MJ et al. 2019. Genera of phytopathogenic fungi: GOPHY 2. Stud Mycol 92:47–133. https://doi.org/10.1016/j.simyco.2018.04.002

36. Wanasinghe DN, Phukhamsakda C, Hyde KD et al. 2018. Fungal diversity notes 709–839: taxonomic and phylogenetic contributions to fungal taxa with an emphasis on fungi on Rosaceae. Fungal Divers 89:1–236. https://doi.org/10.1007/s13225-018-0395-7

37. Pereira C, Ferreira B, Aucique-Perez C, Barreto R. 2021. *Diaporthe rosiphthora* sp. nov.: Yet another rose dieback fungus. Crop Prot 139:105365. https://doi.org/10.1016/j.cropro.2020.105365

38. Hilário S, Amaral IA, Gonçalves MFM, Lopes A, Santos L, Alves A. 2020. *Diaporthe* species associated with twig blight and dieback of *Vaccinium corymbosum* in Portugal, with description of four new species. Mycologia 112:293–308. https://doi.org/10.1080/00275514.2019.1698926.

39. Udayanga D, Liu XZ, Mckenzie EHC, Chukeatirote E, Hyde KD. 2012. Multi-locus phylogeny reveals three new species of *Diaporthe* from Thailand. Cryptogamie Mycologie 33:295–309. https://doi.org/10.7872/crym.v33.iss3.2012.295

40. Feng XX, Chen JJ, Wang GR, Cao TT, Zheng YL, Zhang CL. 2019. *Diaporthe sinensis*, a new fungus from *Amaranthus* sp. in China. Phytotaxa 425:259–268. https://doi.org/10.11646/phytotaxa.425.5.1

41. Santos JM, Correia VG, Phillips AJL. 2010. Primers for mating-type diagnosis in *Diaporthe* and *Phomopsis*, their use in teleomorph induction in vitro and biological species definition. Fungal Biol 114:255–270. https://doi.org/10.1016/j.funbio.2010.01.007

42. Hyde KD, Chaiwan N, Norphanphoun C et al. 2018. Mycosphere Notes 169–224. Mycosphere 9:271–430. https://doi.org/10.5943/mycosphere/9/2/8

43. Liu JK, Hyde KD, Jones EBG et al. 2015. Fungal Diversity Notes 1–110: taxonomic and phylogenetic contributions to fungal species. Fungal Divers 72:1–197. https://doi.org/10.1007/s13225-015-0324-y

44. Crous PW, Wingfield MJ, Le Roux JJ et al. 2015. Fungal Planet description sheets: 371–399. Persoonia 35:264–327. https://doi.org/10.3767/003158515X690269

45. Mostert L, Crous PW, Kang J-C, Phillips AJL. 2001. Species of *Phomopsis* and a *Libertella* sp. occurring on grapevines with specific reference to South Africa: morphological, cultural, molecular and pathological characterization. Mycologia 93:146–167. https://doi.org/10.1080/00275514.2001.12061286

46. Noriler SA, Savi DC, Ponomareva L, Rodrigues R, Rohr J, Thorson JS, Glienke C, Shaaban KA. 2019. Vochysiamides A and B: Two new bioactive carboxamides produced by the new species *Diaporthe vochysiae*. Fitoterapia 138:104–273. https://doi.org/10.1016/j.fitote.2019.104273

47. Gao YH, Liu F, Duan W, Crous PW, Cai L. 2017. *Diaporthe* is paraphyletic. IMA Fungus 8:153–187. https://doi.org/10.5598/imafungus.2017.08.01.11
